# International consensus on fasting terminology

**DOI:** 10.1016/j.cmet.2024.06.013

**Published:** 2024-07-25

**Authors:** Daniela A. Koppold, Carolin Breinlinger, Etienne Hanslian, Christian Kessler, Holger Cramer, Anika Rajput Khokhar, Courtney M. Peterson, Grant Tinsley, Claudio Vernieri, Richard J. Bloomer, Michael Boschmann, Nicola L. Bragazzi, Sebastian Brandhorst, Kelsey Gabel, Alan C. Goldhamer, Martin M. Grajower, Michelle Harvie, Leonie Heilbronn, Benjamin D. Horne, Spyridon N. Karras, Jost Langhorst, Eva Lischka, Frank Madeo, Sarah J. Mitchell, Ioannis-Eleemon Papagiannopoulos-Vatopaidinos, Maria Papagiannopoulou, Hanno Pijl, Eric Ravussin, Martha Ritzmann-Widderich, Krista Varady, Lilian Adamidou, Melika Chihaoui, Rafael de Cabo, Mohamed Hassanein, Nader Lessan, Valter Longo, Emily N.C. Manoogian, Mark P. Mattson, J. Brent Muhlestein, Satchidananda Panda, Sousana K. Papadopoulou, Nikolaos E. Rodopaios, Rainer Stange, Andreas Michalsen

**Affiliations:** 1 Institute of Social Medicine, Epidemiology and Health Economics, Charité - Universitätsmedizin Berlin, Corporate Member of Freie Universität Berlin and Humboldt-Universität zu Berlin, 10117 Berlin, Germany; 2 Department of Internal Medicine and Nature-Based Therapies, Immanuel Hospital Berlin, 14109 Berlin, Germany; 3 Charité Competence Center for Traditional and Integrative Medicine (CCCTIM), Charité – Universitätsmedizin Berlin, Corporate Member of Freie Universität Berlin, Humboldt-Universität zu Berlin and Berlin Institute of Health, Berlin, Germany; 4 Institute for General Practice and Interprofessional Care, University Hospital Tübingen, Tübingen, Germany; 5 Robert Bosch Center for Integrative Medicine and Health, Bosch Health Campus, Stuttgart, Germany; 6 Department of Dermatology, Venereology and Allergology, Charité - Universitätsmedizin Berlin, Corporate Member of Freie Universität Berlin and Humboldt-Universität zu Berlin, Berlin, Germany; 7 Department of Nutrition Sciences, University of Alabama at Birmingham, Birmingham, AL, USA; 8 Department of Kinesiology & Sport Management, Texas Tech University, Lubbock, TX 79409, USA; 9 Medical Oncology Department, Fondazione IRCCS Istituto Nazionale dei Tumori, Milan, Italy; 10 IFOM ETS, the AIRC Institute of Molecular Oncology, Milan, Italy; 11 College of Health Sciences, The University of Memphis, Memphis, TN 38152, USA; 12 Experimental & Clinical Research Center - A joint co-operation between Charité Universitätsmedizin und Max-Delbrück Center for Molecular Medicine in the Helmholtz Association, Clinical Research Unit, Berlin, Germany; 13 Department of Mathematics and Statistics, Laboratory for Industrial and Applied Mathematics (LIAM), York University, Toronto, ON, Canada; 14 Longevity Institute, Davis School of Gerontology, University of Southern California, Los Angeles, CA, USA; 15 Department of Kinesiology and Nutrition, University of Illinois at Chicago, 1919 West Taylor Street, Chicago, IL 60612, USA; 16 TrueNorth Health Foundation, Santa Rosa, CA 95404, USA; 17 TrueNorth Health Center, Santa Rosa, CA 95404, USA; 18 Division of Endocrinology, Albert Einstein College of Medicine, Bronx, New York, NY, USA; 19 Prevent Breast Cancer Research Unit, The Nightingale Centre, Manchester University NHS Foundation Trust, Manchester, England; 20 Division of Cancer Sciences, The University of Manchester, Manchester, England; 21 Adelaide Medical School, Faculty of Health and Medical Sciences, The University of Adelaide, Adelaide, SA, Australia; 22 Nutrition, Metabolism & Gut Health, Lifelong Health Theme, South Australian Health and Medical Research Institute (SAHMRI), Adelaide, SA, Australia; 23 Intermountain Medical Center Heart Institute, Salt Lake City, UT, USA; 24 Division of Cardiovascular Medicine, Department of Medicine, Stanford University, Stanford, CA, USA; 25 Laboratory of Biological Chemistry, Medical School, Aristotle University, 54636 Thessaloniki, Greece; 26 Department for Internal and Integrative Medicine, Sozialstiftung Bamberg Hospital, Bamberg, Germany; 27 Department for Integrative Medicine, University of Duisburg-Essen, Medical Faculty, Bamberg, Germany; 28 Klinik Buchinger Wilhelmi, Überlingen, Germany; 29 BioTechMed Graz, Graz, Austria; 30 Institute of Molecular Biosciences, University of Graz, Graz, Austria; 31 Field of Excellence BioHealth, University of Graz, Graz, Austria; 32 Ludwig Princeton Branch, Ludwig Institute for Cancer Research, Princeton University, Princeton, NJ, USA; 33 Vatopaidi Monastery Hospital, 63086 Mount Athos, Greece; 34 Medical Office for Fasting Therapy, Danaedon 24A, Chalandri, 15232 Athens, Greece; 35 Department of Internal Medicine, Leiden University Medical Center, Leiden, the Netherlands; 36 Pennington Biomedical Research Center, Baton Rouge, LA, USA; 37 Praxis für Ernährungsmedizin und Prävention in Rottweil, Hochbrücktorstraße 22, 78628 Rottweil, Germany; 38 Department of Dietetics and Nutrition, AHEPA University Hospital, Thessaloniki, Greece; 39 Department of Endocrinology, University Hospital La Rabta, Faculty of medicine of Tunis, University Tunis El Manar, Tunis, Tunisia; 40 Experimental Gerontology Section, Translational Gerontology Branch, National Institute on Aging, Baltimore, MD, USA; 41 Department of Endocrinology and Diabetes, Dubai Hospital, Dubai Academic Health Cooperation, United Arab Emirates; 42 The Research Institute, Imperial College London Diabetes Centre, Abu Dhabi, United Arab Emirates; 43 Regulatory Biology Department, Salk Institute for Biological Studies, La Jolla, CA, USA; 44 Department of Neuroscience, Johns Hopkins University School of Medicine, Baltimore, MD 21205, USA; 45 The Intermountain Medical Center, Murray, UT, USA; 46 Department of Nutritional Sciences and Dietetics, School of Health Sciences, International Hellenic University, 57001 Thessaloniki, Greece; 47 Department of Social Medicine, Preventive Medicine and Nutrition Clinic, School of Medicine, University of Crete, Voutes, 71003 Iraklion, Greece; 48 Lead contact

## Abstract

Although fasting is increasingly applied for disease prevention and treatment, consensus on terminology is lacking. Using Delphi methodology, an international, multidisciplinary panel of researchers and clinicians standardized definitions of various fasting approaches in humans. Five online surveys and a live online conference were conducted with 38 experts, 25 of whom completed all 5 surveys. Consensus was achieved for the following terms: “fasting” (voluntary abstinence from some or all foods or foods and beverages), “modified fasting” (restriction of energy intake to max. 25% of energy needs), “fluid-only fasting,” “alternate-day fasting,” “short-term fasting” (lasting 2–3 days), “prolonged fasting” (≥4 consecutive days), and “religious fasting.” “Intermittent fasting” (repetitive fasting periods lasting ≤48 h), “time-restricted eating,” and “fasting-mimicking diet” were discussed most. This study provides expert recommendations on fasting terminology for future research and clinical applications, facilitating communication and cross-referencing in the field.

## In brief

Although fasting is being studied extensively around the world, there was no common definition of basic terms until now. This publication presents an international consensus process on such terms, including fasting, intermittent fasting, time-restricted eating, long-term and short-term fasting, as well as fasting-mimicking diets.

## Graphical Abstract

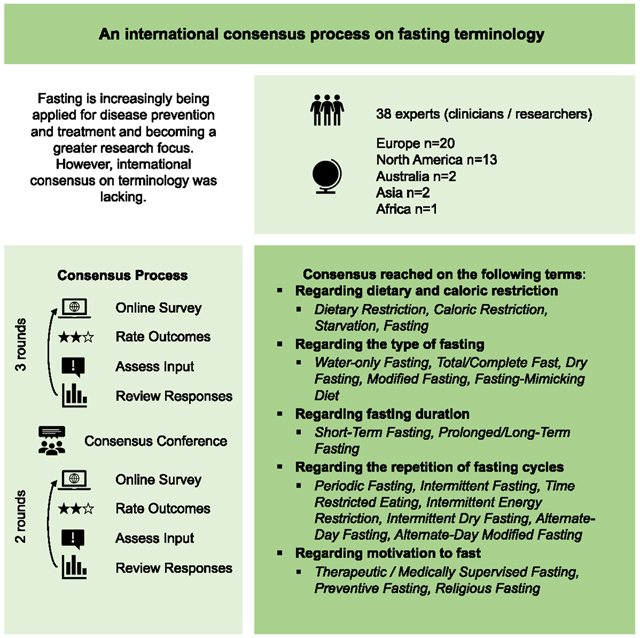

## INTRODUCTION

Various types of fasting have seen a substantial rise in scientific publications over the past two decades, including intermittent fasting (IF), time-restricted eating (TRE), longer fasting periods, short-term fasting (STF), and diverse religious forms of fasting. There is abundant evidence from animal studies showing that fasting can prevent and potentially treat a broad spectrum of chronic diseases.^1,2^ Notably, fasting interventions slow cellular aging processes by affecting key hallmarks of aging, thereby extending life span from yeast to mammalian species.^3,4^ Alongside these findings, the potential of fasting interventions for the treatment of clinical conditions gained attention, with indications ranging from cardiometabolic,^5–7^ inflammatory,^8,9^ and autoimmune diseases^10^ as well as adjunct interventions during chemotherapy in cancer.^11–14^ Recently, the impact of fasting on neurodegenerative diseases via neurogenesis, brain-derived neurotrophic factor (BDNF) signaling, and synaptic plasticity has become another focus of active research in the field.^15^

The main physiological mechanisms of fasting, constituting the so-called “metabolic switch,” are well described. They include an initial systemic stress response leading to a depletion of glycogen storages, followed by enhanced lipolysis and release of free fatty acids, gluconeogenic substrates, and ketone bodies to replace glucose as the main energy supply. Biochemically, fasting modulates central metabolic signaling pathways: among others, fasting downregulates mammalian target of rapamycin (mTOR), insulin, and insulin-like growth factors while upregulating 5′ adenosine monophosphate-activated protein kinase (AMPK) and triggering repair mechanisms. Downstream effects involve epigenetic modification through histone acetyltransferases and the activation of sirtuin pathways enhancing DNA repair, class O of forkhead box transcription factors (FOXO)-mediated stress resistance, and mitochondrial biogenesis.^2,3^ Processes such as autophagy, protein kinase A (PKA)-associated optimization of glucose homeostasis, modulation of microbiota, NRF2-mediated antioxidative effects, as well as stem cell regeneration have also been linked to the fasting state.^2,16,17^

In addition to increasing preclinical understanding of the mechanisms through which fasting may contribute to the delay or treatment of different diseases, preliminary evidence from clinical trials also indicates that fasting approaches may be used for the prevention or treatment of cardiovascular, cerebrovascular, inflammatory, and oncological diseases.^16,18^ These data seem to confirm that the molecular mechanisms that mediate the therapeutic effects of fasting in preclinical models, such as an impact on systemic metabolism and immunity, also apply to humans.

Qualitative and behavioral aspects of the subjective fasting experience represent a growing focus of current research as well. Studies on various religious fasting traditions^19–22^ often include clinical^23–26^ and psychosocial parameters^27,28^ as well as related aspects of self-efficacy, religiosity, and spirituality.^29,30^

Although the physiological mechanisms and biochemical pathways involved in diverse forms of fasting and caloric restriction are being progressively clarified, the concomitant clinical terminology has remained heterogeneous and often confusing, with similar terms being used to define different fasting regimens. This variety arises for several reasons, reflecting the manifold contexts in which fasting is practiced. The growing public interest in fasting has mainly been driven by the impressive results of basic and animal research conducted over the last few decades. Although caloric restriction was the initial focus of research, more and more fasting protocols were tested, once it became clear that at least some of the effects of caloric restriction were not due to chronic energy restriction per se. These studies concluded that intervals characterized by severe restriction of caloric intake or specific nutrients may play a major role in the positive outcomes of caloric restriction.^31,32^ Thus, especially STF and IF approaches have captured scientific and medical interest over the last few years. On the other hand, clinical applications of longer fasting regimens as a treatment strategy have a long-standing history worldwide and have thus been molded by diverse settings, necessities, and practitioner experiences. Especially in Germany and Austria, medically supervised fasting in clinics, hospitals, and outpatient settings looks back on a history of more than 150 years. This historical past has shaped practice and terminology, having led to a German consensus process in 2002, with an update in 2013.^33^ At the same time, religious forms of fasting have been practiced by a great percentage of the world’s population for centuries or even millennia, shaping traditions and being shaped by history, beliefs, and geographical circumstances. In contrast to the therapeutic setting, fasting as a religious practice aims at strengthening certain virtues or expressing devotion through its ascetic context and thus usually exempts the sick from the obligation to follow it. These different frameworks in which fasting is studied, applied, and practiced result in great diversification of fasting methods and terminology. The associated translational, international, and disciplinary challenges are obvious. The practical implications of existing inconsistencies in definitions translate into difficulties in literature search, review generation, meta-analysis, and cross-referencing. In consistencies in definitions also lead to challenges for practitioners, dietitians, and physicians when designing and conducting clinical trials, as well as when applying the available evidence in clinical practice.

Consensus processes, particularly the Delphi method, are increasingly being applied to develop expert consensus, define current positions, and merge expert recommendations. The Delphi method is defined by four main features, including the participation of experts, anonymity, “iterations” (for example, a series of questionnaire rounds) with controlled feedback, and statistical aggregation of individual responses, also referred to as “statistical group response.”^34^

Here, we present the results of the first international consensus process on fasting terminology. In a historical period characterized by a growing use of fasting approaches in clinical research and practice for the prevention or treatment of human diseases, the results of this project will promote clarity, rigor, and comparability of definitions and will help foster cooperation and cross-referencing in interrelated scientific fields.

## RESULTS

### Panelists

**Figure 1.**
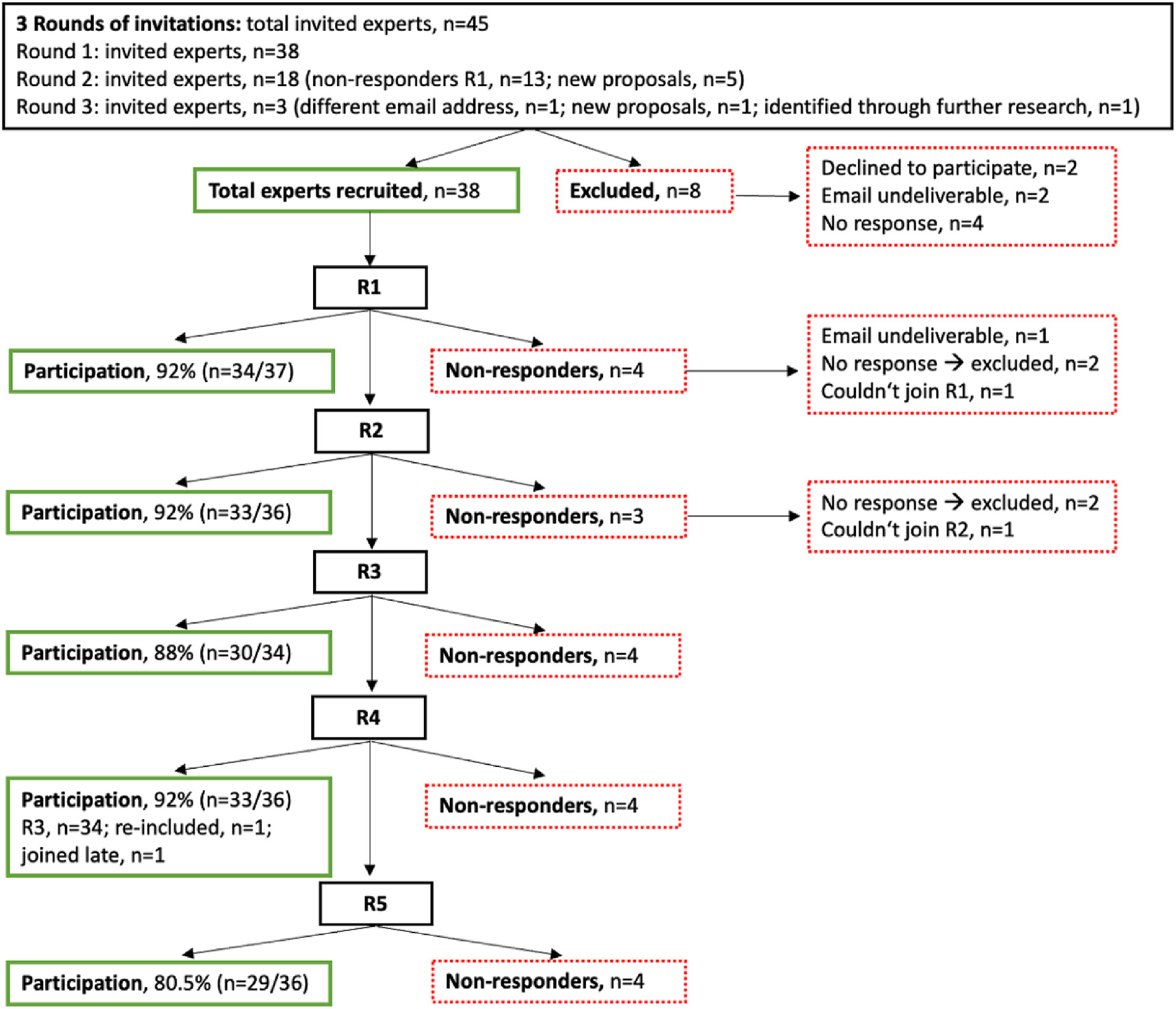
Flowchart of the recruitment process and participation in the study

Figure 1 shows a flowchart of the recruitment process and participation during the Delphi process. Of 45 fasting experts contacted in three rounds of invitations, 38 agreed to participate in this consensus process. Those participants who did not finish the first or second survey and did not provide a justification to the steering committee (SC) were excluded from further rounds of the survey. Of 38 experts who agreed to participate in the study, only 37 people received the invitation to the first round of questionnaires due to a technical error. Of these 37 individuals, 34 completed the first round. Two panel members were excluded after this round. The person who had not received the link to the first survey was offered to participate in the second round. Thus, 36 experts were contacted for the second round, 33 of whom completed the questionnaire. Two individuals were not considered for the third round, as they did not respond to the second survey, resulting in a total of 34 experts invited to participate in the third survey. Of these, 30 returned the third questionnaire. One person was made aware of the consensus process after the third round by a participating expert. This person contacted the SC and was subsequently offered to participate in the last two rounds of the survey. Thus, for the fourth and fifth rounds, 36 experts were invited. In addition to the person joining the panel late, another expert who had already been invited for the first two rounds but did not participate was re-included in the panel at his own request. Thirty-three experts completed the fourth round, and 29 completed the final round. A total of 38 fasting experts (Europe [*n* = 16], North America [*n* = 18], Australia [*n* = 1], Asia [*n* = 2], and Africa [*n* = 1]) completed at least one questionnaire, and 25 experts (Europe [*n* = 12], North America [*n* = 12], and Australia [*n* = 1]) responded to all five surveys. Participation in the online consensus conference was not mandatory, but 18 experts participated in this event. A total of 26 fasting experts took part in a short survey to clarify a few remaining issues that emerged during the peer review process. Table 1 provides demographic data on the 38 panel members who participated in one or more rounds of this consensus process.

### Elements of fasting terminology and evaluation

**Figure 2.**
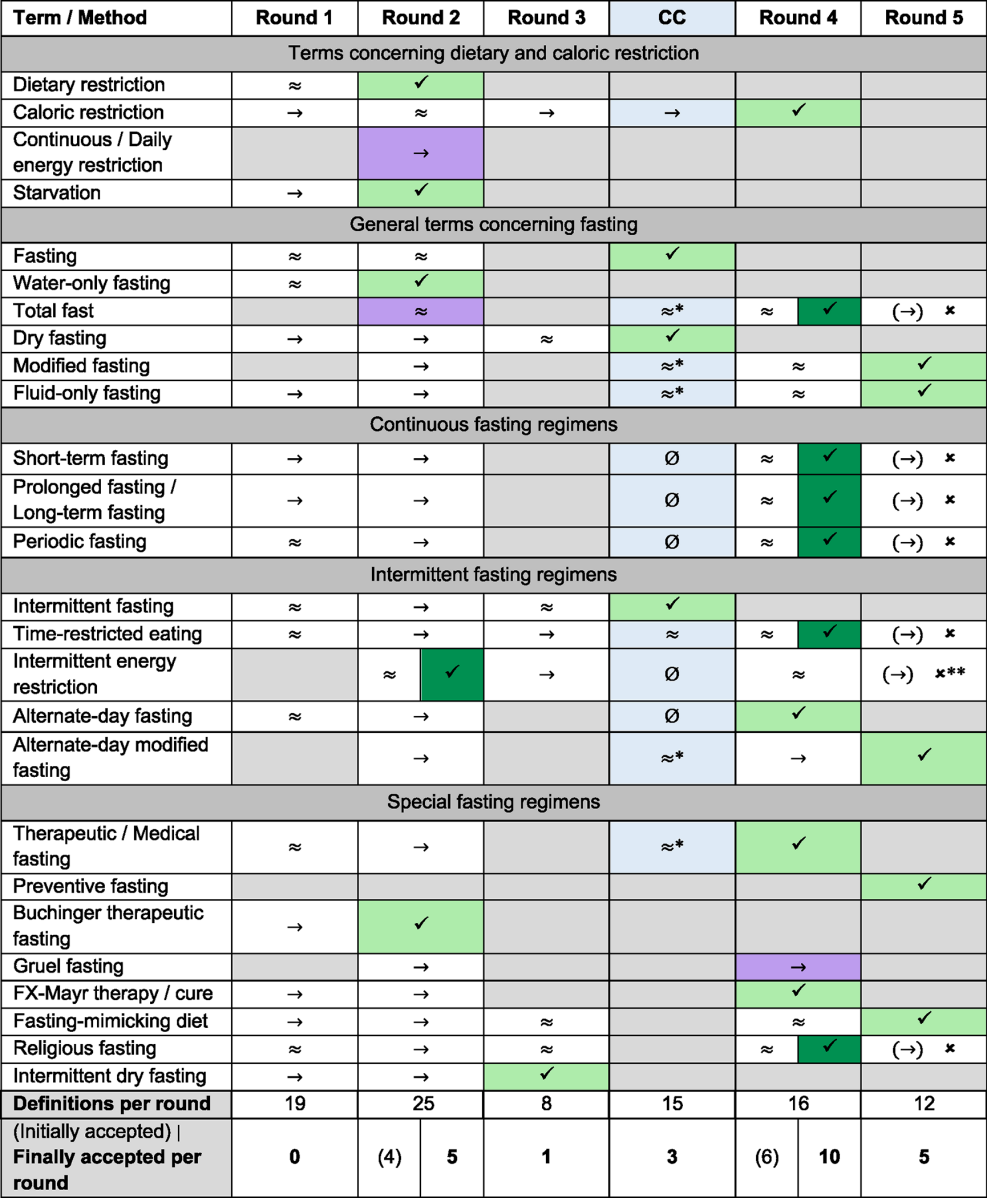
Overview of the consensus process for all terms CC, consensus conference. Gray field: term does not occur in this round; violet field: term that is not given its own definition but will be combined with another term in the next round; dark green: term on which no consensus was reached in final round, so last consensus reached is counted (exception: IER); ✔: final consensus in this round; ≈: ≥70% agreement, but changes required; ≈*: agreement in CC, but new assessment in round 4 required; Ø: not discussed in the CC or not discussed in detail due to time constraints; → and (→): <70% agreement; ✘: no consensus at the end of final round, last consensus reached is used; ✘**: exception: consensus from round 2 is used.

In five rounds of questionnaires, as well as one online consensus conference and a short survey on some open questions that arose during the peer review process, consensus was reached for all proposed 24 definitions. The final definitions for the final 24 terms can be found in Table 2. The process of consensus building for all terms is visualized in Figure 2. Those terms for which consensus building proved difficult are also discussed in detail below. The supplement contains a comprehensive result table with all proposed changes to definitions across all five rounds and the online consensus conference and includes the distribution of votes for each definition (Figure S1). Figure S2 outlines the questions we asked the panel during the peer review process along with the corresponding results.

In the first round of the survey, some definitions included references to animals. Expert comments indicated that separate definitions are required for animal models since optimal timing of fasting and caloric intake varies by species. Additionally, differences between starvation, hunger, and fasting are more difficult to define in animals than in humans. Due to these differences and to reduce complexity of the surveys, the SC decided to limit the terminology to definitions relevant to humans and exclude all references to animals from round 2 onward.

### Terms most discussed

#### Caloric restriction

It took longer than expected to achieve a consensus on the definition of caloric restriction. In the initial rounds, possible definitions included various thresholds constituting the minimum and/or maximal daily caloric intake needed for caloric restriction while also distinguishing it from starvation. However, after a few rounds, panelists decided to drop any quantitative criteria and define caloric restriction more broadly as a reduction in energy intake below the total amount of calories that would be needed to maintain a person’s current body weight, without causing malnutrition.

#### Fasting

Despite reaching a consensus of 88% and 85% in the first and second rounds, many comments were made on the definition of fasting. The SC initially chose a broad definition in order to present the term fasting as a generic term for all possible forms of fasting. Many experts agreed with this broad definition. Panelists wished to mention motivations for fasting, such as “preventive,” “religious,” “cultural,” and “other reasons” to make the definition even more comprehensive. However, experts were against the inclusion of “political reasons,” since in this context, the line between fasting and starvation is not always clear. On the other hand, several experts indicated that they considered fasting to only be the complete abstention from food and caloric beverages. Ultimately, fasting was defined as “a voluntary abstinence from some or all foods or foods and beverages for preventive, therapeutic, religious, cultural, or other reasons.”

#### Modified fasting

Modified fasting, like the term fasting, is meant to serve as a generic term; in this case, it refers to all fasting regimens that limit energy intake to typically up to 25% of energy needs on modified fasting days. To support the clinical application of modified fasting regimens, we supplement the definition with a table indicating the amount of calories that correspond to percent energy requirements based on age, body composition, and activity level (see Figure S3).

#### Fluid-only fasting

The SC suggested this definition to distinguish fasting methods allowing for caloric intake via fluids only, a practice that is common in Germany, from the Buchinger therapeutic fasting, which has a defined intake of fluids and a specific clinical setup. Some experts noted that no calories should be consumed during a fluid-only fasting regimen or that the consumption of 500 kcal/day could not be called “fasting” but should rather be referred to as “caloric restriction” or “modified fasting.” At the same time, two experts even advocated a higher caloric intake of up to 800 kcal/day based on the common definition of very-low-calorie diets (VLCDs), which are popular in the USA. As in the consensus conference, the definition of modified fasting was adopted, the concept of fluid-only fasting was included as a subtype of modified fasting, and the upper calorie limit was set at 500 kcal/day. This value also corresponds to the upper limit specified in the guidelines for therapeutic fasting.^33^ Thus, in the final definition, fluid-only fasting refers to a modified fasting regimen where only beverages are consumed for a certain period of time. Water and unsweetened herbal tea may be consumed *ad libitum*. Clear vegetable broth, vegetable, and/or fruit juices may be consumed up to a total of 500 kcal/day. Ultra-processed fluids should not be consumed.

#### Dry fasting

In the first and second rounds, some experts indicated their concerns about the safety of this method. Since they were not among those experienced in practice or research of dry fasting and because this consensus process was only intended to serve as a collection of existing fasting methods, the SC decided to retain the term dry fasting. In fact, dry fasting is used in therapeutic and cultural/religious contexts for example in the form of intermittent dry fasting.^22,25,26^ In the first and second rounds, several other experts pointed out that total/complete fasting could be equated with dry fasting. For this reason, the definition of total/complete fasting was deleted in the third round and the terms total/complete fasting were listed under dry fasting. This equation was criticized by four experts, which is why both terms were discussed in the consensus conference. In the final definition, dry fasting corresponds to “a fasting regimen during which a voluntary abstinence from all foods and beverages, including water, is practiced for a certain period of time.”

#### Total/complete fasting

A total/complete fast was ultimately decided to refer only to those fasting regimens “where no calories are consumed for a certain period of time.” Participants concluded that total/complete fasts may be equated with water-only fasting but not with dry fasting.

#### FMD

In the consensus conference, it was initially decided that the fasting-mimicking diet (FMD) should be categorized as a modified fasting regimen. Ultimately, FMDs were instead classified under “specific fasting regimens” for the following reasons: it was agreed to set the threshold for maximum caloric intake on modified fasting days at up to 25% of energy requirements, and FMDs may allow an intake of ≥50% (≥1,000 kcal) of energy needs on some “fasting” days. If the caloric threshold for modified fasting was set at such a high value, the definition of modified fasting would have been similar to the definition of caloric restriction.

### IF regimens

IF, TRE, and intermittent energy restriction (IER) were the most discussed terms in this category.

#### IER

A major point of discussion for IF, TRE, and IER was the relationship among the three terms. IER, presented in the second and third surveys as a superordinate category to IF and TRE, included “periods of caloric restriction alternating with periods of *ad libitum* eating.” In the second round, this definition reached an agreement of 81%. However, in both surveys, two experts disagreed with subsuming TRE under IER without providing a conclusive explanation for their decision. Given the discrepancies among participants regarding the hierarchy of IF regimens, IER was one of the terms discussed in the consensus conference. Only one panelist expressed an opinion on this term in the consensus conference. Subsequently, in the fourth round, a definition of IER was offered, which included “periods of modified fasting alternating with periods of *ad libitum* energy intake.” According to this definition, IF was presented as including the subcategories of IER and TRE. Although this definition received 74% agreement, several participants disagreed with it. Two experts said that the wording “caloric restriction” would be more appropriate than “modified fasting” because IER programs often allow energy intakes that are above the energy intake threshold of modified fasting. Three individuals were in favor of changing the hierarchy of terms. According to two of them, IER should be presented as an upper category because IER would be a broader concept than IF. Thus, in the fifth round, the SC offered a definition of IER with similar wording as in the second round (=IF as a subcategory of IER regimens) and the definition from the fourth round (=IER as a subcategory of IF) for consideration. Both wordings received over 70% agreement in the second and fourth rounds, respectively, but in the fifth round, consensus could not be reached for either definition. According to the evaluation rules, the last consensus reached (round 4) should have been adopted as the final definition. Nevertheless, the SC decided to use the definition that found consensus in the second round instead, for the following reasons: this definition yielded a higher level of agreement (81%) than the round 4 definition (74%); it is consistent with the accepted definition for IF, and the wording “caloric restriction” fits the IER definition better than “modified fasting,” as in this way, regimens with smaller energy deficits are also included. Thus, IER was ultimately defined as alternating periods of caloric restriction with periods of *ad libitum* eating, and IF is therefore a subcategory of IER.

#### IF

The final definition of IF, which refers to repetitive fasting periods lasting up to 48 hours each, includes TRE and was finally accepted in the consensus conference. However, the inclusion of TRE under the umbrella of IF was repeatedly opposed by a few participants. The main argument was that TRE is more a chronobiological intervention than a “fasting” intervention, as it does not necessarily include a change in diet or caloric intake and as such does not fit into the concept of IF and IER.

#### TRE

TRE was finally defined as a dietary regimen with a fasting window of “at least 14 h” per day in humans and no explicit limit on energy intake during eating hours. Three leading experts in TRE research would have preferred a fasting window of “at least 12 h” drawing from their clinical experience. More precisely, two experts on TRE and chronobiology proposed the following definition during the last survey: “TRE is a dietary regimen in which all calorie intake is restricted to a consistent period of time during the 24-h day, resulting in a daily fasting window of 12–18 h. There is no explicit limit on energy intake during eating hours.” We do, however, note that studies had already appeared in the literature testing TRE interventions with fasting windows longer than 18 hours in both animals and humans.^35^

### Continuous fasting regimens

The main discussion in this category concerned the distinction between the concepts STF and prolonged fasting/long-term fasting (PF/LTF) and the formulation of the definition of periodic fasting.

#### STF and PF

When discussing the durations for STF and PF/LTF, experts in all rounds suggested different time periods, ranging from “16–72 h” to “1–7 days” for STF and “≥3 days” to “≥14 days” for LTF. A duration of “1–3 days” for STF and a duration of “≥4 days” was offered for LTF in the fourth round, reaching an agreement of 81%. Some participants suggested changes in the wording and duration of both regimens. These changes were incorporated and presented for re-evaluation in the fifth round, as well as an additional definition by the SC for STF of “2–3 days,” since the final definition of IF already included fasting practices of up to 48 h. Consensus could not be reached in the fifth survey; hence, the previously mentioned durations from the fourth survey were to be used as final definitions of STF and LTF. Regarding the actual dietary regimen followed during the fasting hours, some participants argued during round 4 that water-only fasting should be included alongside modified fasting. However, no consensus was reached on this issue in round 5. To make the definitions of STF and LTF more coherent, both definitions were changed in the round of the peer review process to include any fasting regimen and not just fluid-only fasting regimens. This decision was agreed upon by 96% of the participating experts in this new survey round. Additionally, 76% of the participating experts in this survey round agreed to adjust the duration in the definition of STF from “1–3 days” to “2–3 days” to distinguish STF from IF regimens, which last up to 48 h. Thus, the final definitions are as follows: “STF refers to fasting regimens with a duration of 2–3 days” and “PF, also called LTF, refers to fasting regimens lasting ≥4 consecutive days.”

#### Periodic fasting

In the first round, 71% of the experts agreed that the term periodic fasting means fasting at regular intervals, e.g., daily. The disagreeing experts considered fasting programs that are performed “daily,” “every other day,” or “once or several times a week” as IF, and periodic fasting should define fasts of at least 2 days in length. In the second round, these proposed changes were incorporated into an alternative definition. Neither this nor the initial definition reached consensus. In the fourth round, the SC offered the definition with the majority of votes from the second round (the original definition) for re-evaluation. This definition reached an agreement of 81%. However, some experts objected to this proposal again, demanding a distinction between the definition of periodic fasting and frequent IF regimens. One expert requested the intervals in periodic fasting to be regular, whereas another one said they should be defined “without any particular interval,” since “periodic fasting should refer to any form of PF whether it is at regular intervals or not.” In the fifth round, an alternative definition of periodic fasting was presented, defined as a fasting regimen that is prolonged and repeated at intervals. The intervals were not further specified. Consensus could not be reached for this definition. Therefore, the definition from round 4 was accepted. It should be noted that this accepted definition is in line with the first published uses of the term, such as those found in a publication by Horne et al..^36^ In some later works, the term referred to a fast “lasting several days or longer every 2 or more weeks.”^37^ Ultimately, in this consensus process, periodic fasting was defined as “any fasting regimen that is repeated at regular intervals (periods), such as every day, every week, or every several months.” Thus, IF would be considered a subcategory of periodic fasting.

## DISCUSSION

This consensus process aimed to unify terminology in clinical fasting research and practice by developing an expert consensus. It also served to identify which terms or aspects of definitions need further discussion or scientific research. The various fasting regimens discussed in this publication suggest the potential for positive health effects in humans while having only few side effects including no negative effects on aspects of planetary health. The authors hope that this Delphi process will facilitate high-quality fasting research on a global scale by creating a common international terminological basis on which fasting programs can be systematically examined and applied.

Thirty-eight experts in the field participated in at least one and up to five rounds of surveys and one online consensus conference, finally reaching consensus on 24 definitions. A few more questions arose during the peer review process, which we asked the expert panel to clarify through a brief survey. In the future, we recommend using these definitions in clinical research and practice in order to achieve comparability of methods and study results. This work is meant to serve as a starting point, as definitions should and will be further refined and revised in the future as new clinical data become available. A reassessment of these definitions is planned in 5 years’ time at the latest. Creating possible new forms of fasting or evaluating evidence, safety, and feasibility of existing fasting regimens were not part of this study.

### Implications of certain definitions in view of existing literature

#### Caloric restriction

Caloric restriction has been defined in various publications, with restrictions varying between 20% and 40% of *ad libitum* intake,^38–40^ to 20% and 50% of needs,^41^ to decreasing energy intake by 15% and 60% of baseline needs,^42^ or to 10% and 25% of (usual) caloric intake.^43^ Elsewhere, it has been defined as “a state in which energy intake is sufficiently low to achieve or maintain a low-normal body weight status.”^44^ Trying to unify the terminology proved challenging, as different studies and clinical circumstances may require adaptations of the degree of calorie reduction. Discussing these points in the consensus conference, panelists decided to leave out the definition of any specification of caloric intake. Instead, it was decided to use “the amount of calories that would be needed to maintain a person’s current body weight” as the baseline from which to define caloric restriction. Panelists agreed that the terms continuous energy restriction (CER) and daily energy restriction (DER) were synonymous and can be used interchangeably for longer periods of caloric restriction.

#### Fasting

Fasting has been used historically in diverse contexts and cultures for millennia. As a result, in the first two rounds, consensus was difficult to obtain. One of the central debates was whether fasting permits any calorie intake. During the consensus conference, it was agreed to use fasting as an umbrella term, giving it the broadest possible context. Therefore, any reference to minimal duration (as in TRE), amount of global calorie intake (as in medical fasting), abstinence from certain foods (as in Christian orthodox fasting), or specific nutrients or fluids (as in fluid-only fasting or dry fasting) was omitted.

#### Modified fasting

Giving fasting this broad definition made it clear during the consensus conference that modified fasting needed to be more precisely defined to suit clinical application. During the following survey rounds, agreement was reached for modified fasting to typically mean a limitation of energy intake to a maximum of 25% of energy needs. During the consensus conference, it was decided to add a table to support clinicians and researchers in determining the necessary amount of calories to approximate this degree of energy restriction according to body mass, age, and sex (see Table S3). Panelists decided against counting FMDs as a modified fasting regimen, as the caloric intake in FMDs might be higher than 25% of energy needs in some clinical contexts.

#### Total/complete fasting

The broad definition of fasting also led to deliberations around the concepts of total/complete fasting. In the consensus conference, it was proposed to use these terms interchangeably, making it clear that they both meant a fast that did not allow for any caloric intake from food or fluids.

#### STF vs. PF

The terms STF and PF were not easy to differentiate, as there are very limited data on the differential effects of fasts between 2 and 5 days. This also made it difficult to delimit IF from STF. The terms PF/LTF have been used to describe different fasting durations in scientific literature, ranging from 2 to 5 days^17,45^ to >5 days^46,47^ to 2 to 21 days or more.^48^ Of note, PF is often also used in surgical literature for fasting durations of 2–6 h.^49,50^ By comparison, the term STF has been used for fasting durations ranging between 48 h^45,51,52^ and 7 days^53^ in literature. Panelists selected “1–3 days” of fluid-only fasting as the definition of STF and “≥4 days” of fluid-only fasting as the definition for PF in round 4. The 3-day threshold to differentiate these two terms is arbitrary, but it was considered by the experts as a reasonable threshold for the activation of specific metabolic adaptation processes in terms of systemic carbohydrate, protein, and lipid metabolism during fasting regimens. The definitions agreed on during this consensus process are hoped to systematize efforts exploring different physiological, biochemical, and, potentially, therapeutical effects of fasts of a shorter duration of up to 3 days to those of a longer duration, lasting at least 4 days. It needs to be remarked, however, that STF has never been used for fasts lasting only 24 h. This point underlines the need to distinguish between STF and IF regimens, as was done in the survey round during the peer review process by setting a timeframe of 2–3 days for STF. Nevertheless, it is recommended to explicitly define the exact fasting duration studied in each research project for a better comparison of fasting regimens of a given duration. Further research is needed to assess physiological mechanisms active in shorter versus longer fasting periods and investigate individual variations depending on body mass index (BMI), level of physical activity, and age. It would also be commendable to add a maximum length to the definition of LTF allowing for adaptations to individual conditions. This could include physiological rationales explicitly limiting the duration to a timeframe in which adipose tissue stores are not depleted.

#### Periodic fasting

Another term that has been used with some variance in published literature is periodic fasting.^16,36,37,54,55^ Some past publications have used the term for a fasting regimen “lasting several days or longer every 2 or more weeks.”^37^ In the consensus process, most experts voted for it to mean repetitive fasting intervals, including IF regimens. This discrepancy should be considered in future research and especially in reviews examining the effects of longer fasting periods.

#### IER vs. IF

The terms IF and IER were discussed throughout all rounds. Panelists disagreed on whether IF is a subcategory of IER or IER is a subcategory of IF. The crux of the matter was whether IER should be defined as periodically practicing (1) caloric restriction (i.e., any form of energy restriction) or (2) modified fasting (≲25% of energy needs). Ultimately, based on both usage in the literature and the round with the greatest agreement, it was decided that IER should be more broadly defined as alternating periods of caloric restriction and *ad libitum* eating. This means that IER can range from hours or days to weeks of caloric restriction followed by phases of *ad libitum* eating. Thus, IF is a subcategory of IER.

#### TRE

The term TRE was also discussed throughout all rounds. Disagreements mainly centered on the question of whether TRE can be seen as a form of IF. On the one hand, it was argued that TRE is mostly a chronobiological intervention and does not necessarily involve caloric restriction.^56^ Thus, it should not be considered a form of fasting. On the other hand, several other panelists mentioned that the prolonged overnight abstention from calories constitutes a bona fide fast, as glycogen stores are depleted toward the end of an extended overnight fast and fat oxidation is upregulated. Additionally, TRE studies have shown that followers restrict their caloric intake naturally by up to 25%^57,58^ when following the regimen.^59^ Moreover, a knockout study in animals found that TRE improves cardiometabolic health in rodents independent of core circadian clock machinery, suggesting that the effects of TRE are mediated through both fasting- and circadian-related mechanisms.^60^

The second major area of contention was the duration of the “fasting” period. A couple of leading experts in the field favored 12 h as the minimum length of the “fasting” period, whereas others favored 14 h as the minimum, which corresponds to a ≤10-h eating window. In the fifth round, two leading TRE experts proposed a broader definition for TRE, emphasizing the importance of a “consistent period during a 24-h day” for the eating and fasting window. The latter, according to the experts, should be individualized to a person’s schedule and bedtime, with a suggested fasting period of 12–18 h per day. However, the panel opted for a 14-h definition for a combination of reasons, including data on the physiology of fasting and epidemiologic data, suggesting that the median length of the overnight fasting period in humans is currently 12 h.^61^ The panel acknowledged that this definition was specific for humans only and that a 12-h window may count as TRE in rodents and other species. The importance of a consistent eating window during TRE, i.e., maintaining the same mealtimes every day as a means of regulating the circadian clock, is confirmed by other clinical studies^62–64^ and should therefore be included in the definition of the term in the future. Regarding the optimal “fasting” duration and timing of TRE, there are no conclusive data available yet.^63^ The definition chosen for TRE in the end does not adequately reflect these deliberations. We hope that more studies on the chronobiological and nutritional implications of TRE will shed light on these issues.

#### FMD

The FMD was also a subject of deliberations, especially because this term employs a functional definition that is based on the capability of this approach to “mimic the metabolic effects of fasting” (Table 2). Since fasting entails several metabolic effects, and these may vary according to the specific clinical context, the available literature did not allow the experts to be more precise in terms of calorie context, macronutrient, and micronutrient composition of FMDs. Also, the lower caloric threshold differentiating most therapeutic fasting regimens from FMDs as well as the maximum caloric threshold for these diets was difficult to establish, as studies on these topics are presently ongoing.

#### Cultural and religious forms of fasting

Obtaining a consensus on the terms for certain traditional forms of fasting proved difficult, including the terms dry fasting, Buchinger therapeutic fasting, and the FX-Mayr therapy/cure. Dry fasting was critically viewed by a few experts, whereas those familiar with religious fasting traditions like Ramadan fasting, Christian orthodox, Bahá’í fasting, or fasting in Judaism as in Yom Kippur were clear about the importance of including this term in the consensus process. The dissent was resolved by the clarification that this form of fasting has historical and geographical extensions and that the present consensus process did not aim at evaluating therapeutic effectiveness or safety for any term defined. In particular, Buchinger therapeutic fasting and the FX-Mayr therapy are traditional fasting regimens in German-speaking countries that evolved during the last century and have medical applications as well as clinics following their provisions.^33^ Internationally, these types of fasting are not widely known and scarcely applied—a fact that was reflected in the opinions of panelists on these terms. Since they are widely used in German-speaking countries, the SC decided to keep them in the list of definitions and ask the fasting experts to vote on the corresponding definitions only if they were familiar with these regimens, which was 23 (for Buchinger therapeutic fasting) and 22 (for FX-Mayr therapy) of fasting.

### Missing supplementary instructions

Fasting, especially in its traditional forms, is often associated with certain supplementary or supportive measures or is accompanied by nutritional advice for non-fasting periods. Portraying some of these details was attempted in the first two rounds of this survey, where issues such as bowel cleansing procedures in fluid-only fasting, the consumption of black coffee during fasting times, or the type of water consumed (mineralized or distilled) in water-only fasting were raised. The complexity and diversity of the answers provided by the panel amply illustrated that the written form was not adequate to do justice to the lack of scientific data on these topics on the one hand, and the broad experience of the participating clinicians on the other. On this account, these topics were omitted in the following rounds and are left for future research to be clarified.

### Strengths and limitations

The main strength of this consensus process lies in the experts themselves and their many years of research and clinical experience. Many of the most cited and highest publishing experts in different fields concerning fasting, namely IF, TRE, alternate-day fasting (ADF), FMD, and Ramadan fasting, were part of the panel, as can be seen in respective bibliometric reviews.^65–69^ The size of the panel was close to optimal, as relevant sources describe groups between 15 and 30 as rendering the best results.^70^ Also, the diversity of the panel is remarkable, ranging from experienced clinicians to translational researchers, encompassing experts on medically supervised fasting and different religious fasting traditions, as well as specialists on topics concerning nutrition, chronobiology, or aging. The length of the process, although strenuous, was helpful to iteratively refine definitions. Adding an online consensus conference to the process on the one hand reduced anonymity, but it also gave a precious opportunity to discuss details difficult to capture in the written form. The flexibility of the SC in adapting rules during the process to match the dynamics of the communication evolving during the rounds, proved crucial in supporting the achievement of consensus on terms that showed a great diversity of opinions. A high response rate of over 80% was achieved for all surveys.

Although carefully selected, expert panels like the one gathered for this work always engender the possible limitation of not including all relevant stakeholders, especially in fields where no bibliometric reviews are at hand. More bibliometric reviews on different fasting regimens could possibly help future panels to be even more representative than ours. Also, diversity in our case did not include geographic considerations, so that the panel consisted mostly of experts based in Europe and the United States of America. Another limitation that lies in the methodology of this process is that online questionnaires naturally only allow for a certain number of questions to be considered. This meant that detailed questions on practical aspects of traditional elements that have been historically associated with certain fasting regimens could not be fully considered. Similarly, this holds true for aspects that might enhance the clinical impact of the specific fasting regimen. For example, the bowel cleansing procedures during Buchinger therapeutic fasting might support the observed microbiota changes by reducing the microbiota at the beginning of the fast.^71,72^ Also, nutritional aspects, often part of counseling that goes alongside fasting recommendations, were not included in the deliberations during this consensus process. The fact that some aspects of the final definitions are based on opinions only is a characteristic of consensus processes, but it also marks one more limitation of the results. There are insufficient data to clarify the threshold between STF and LTF/PF. Similarly, the differences between TRE and IF, as well as between periodic fasting and FMD, are not fully understood. Additionally, the optimal timing or duration of the “fasting” window for TRE has yet to be determined. These gaps highlight how much more exploration is needed in future research. Even the questions of whether there is a certain caloric threshold provoking fasting responses and whether macronutrient composition makes the difference are not yet clear between experts. Furthermore, metabolically inflexible individuals may need to fast for longer to get the same benefits as metabolically flexible individuals; similarly, age, sex, and other factors may play a role. Finally, basic research and translational studies often use clinical fasting terminology for animals, although there might be substantial differences. These differences could result from an altered stress response of animals, as they are involuntarily exposed to food scarcity and hunger. Also, the metabolism of different species varies according to chronobiological features, lifespan, and activity patterns. This consensus process did not adequately address this issue and it is left for future research to clarify terms in this regard.

### Conclusions

This consensus process has documented the necessity of discussing and unifying the scientific terminology on PF and IF and different types of calorie-restricted and fasting-like approaches. The standardized set of 24 definitions presented here fortifies conceptual frameworks, enhances the comparability of results, and reveals open questions. We recommend employing the definitions published in this work for future publications and addressing missing details or unclear aspects in upcoming research. This approach is expected to further develop, revise, and refine fasting terminology in the years to come.

## STAR★METHODS

Detailed methods are provided in the online version of this paper and include the following:

### RESOURCE AVAILABILITY

#### Lead contact

Further information and requests for resources and data should be directed to the corresponding author, Dr. med. Daniela A. Koppold (daniela.koppold@charite.de).

#### Materials availability

This study did not generate new unique reagents.

#### Data and code availability

All data necessary to assess the conclusions of the paper are included in the article or in the supplemental information. All responses to the questionnaires as well as the transcription of the online consensus conference are archived as a pseudonymized dataset on local servers of the Charité Universitätsmedizin Berlin for 10 years. International researchers may be granted access to the data upon justified request.

### EXPERIMENTAL MODEL AND SUBJECT DETAILS

#### Overview

The purpose of this consensus-based modified Delphi study was to achieve a more uniform terminology of fasting terms and regimens. Experts in the field of fasting were invited to participate in a maximum of five rounds (R) of an online survey (R1 in March 2022, R2 in May 2022, R3 in July 2022, R4 in September 2022, and R5 in November 2022) in which definitions of various fasting terms were assessed and amended. Following the peer review process, a short additional survey round was offered in March 2024, in which 26 experts participated to clarify some open questions that had arisen during the peer review process. The questionnaires conformed to the main features of the Delphi technique. A panel of experts was selected according to specific criteria. During the survey (pseudo) anonymity was guaranteed. Feedback was provided consisting of the results of the statistical analysis on each item from the previous rounds and anonymized comments, so that the experts could revise their opinions if necessary.^34^ Terms where consensus seemed difficult to achieve were discussed via a 3-hour online consensus conference following the third round.

#### Steering Committee

A 7-member Steering Committee (SC) (D.A.K.; C.B.; E.H.; C.K.; A.R.K.; H.C.; A.M.) from the Department Internal and Nature-Based Medicine at the Immanuel Hospital Berlin, the Charité - Universitätsmedizin Berlin, and the University Hospital Tübingen/Bosch Health Campus Stuttgart, was responsible for the design and implementation of the study. The joint team of the Immanuel Hospital Berlin and the Charité - Universitätsmedizin Berlin forms a center of competence for the scientifically-based clinical practice of fasting, together with the University Hospital Tübingen/Bosch Health Campus Stuttgart. Various fasting methods and their influence on health and disease are one of the research focuses of the Department for Internal and Nature-based Therapies at the Immanuel Hospital Berlin/Charité - Universitätsmedizin Berlin. Among other aspects, teaching and research is conducted on IF and therapeutic fasting for medical conditions such as high blood pressure, diabetes, breast cancer, rheumatoid arthritis, and osteoarthritis. SC members for this study also included board members of the German Medical Association for Therapeutic Fasting and Nutrition (Ärztegesellschaft Heilfasten & Ernährung e.V., ÄGHE), founders and staff of the Academy for Integrative Fasting (Akademie für Integratives Fasten, AIF), and physicians with further training in fasting. A core team of the SC was responsible for preparing the draft of the final article.

#### Panelists

The SC established a list of known experts in the field of fasting. This list was expanded after reviewing relevant publications on PubMed to ensure that experts in all fasting methods to be defined were represented. The identified experts were contacted via their publicly available email addresses. Snowball sampling was additionally used, whereby the initially identified and invited experts had the opportunity to propose another expert from their research group or to refer the study to peers in the field who they felt also met the inclusion criteria.

Currently, there is no standard sample size for Delphi studies, nor are there general guidelines for the minimum or maximum number of experts on a panel. The size of the panel may depend on the rigor of the inclusion criteria established, the topic being studied, and the number of individuals with expertise in that area.^34,73^ A systematic review by Boulkedid et al., conducted to provide guidance for future Delphi studies on healthcare quality indicators, examined 70 Delphi studies and found that panel sizes ranged from 3 to 418 participants.^74^ The minimum panel size for this study was set at 25 members, with no upper limit.

#### Inclusion Criteria

- Clinicians or scientists with at least five peer-reviewed papers in fasting or;
- Clinicians or scientists with at least one peer-reviewed paper and 5 years of clinical experience in fasting

#### Exclusion Criteria

- Not more than two experts from one institution were allowed to participate

At the beginning of the first questionnaire, panelists had to agree to the privacy statement. Subsequently, panelist eligibility was checked again. If the expert met these criteria, s/he could begin the questionnaire. Delphi panelists received no compensation for their participation in the study, but all participants are credited as co-authors of this publication, if desired. Study participants could withdraw from their participation at any time without giving a reason.

### METHOD DETAILS

#### Study Registration

The study protocol was approved by the institutional review board of Charité - Universitätsmedizin Berlin (Charitéplatz 1, 10117 Berlin) in February 2022 (ID: EA4/028/22). The study was registered with ClinicalTrials.gov (ClinicalTrials ID: NCT05668156) and conducted according to the standards of the Declaration of Helsinki. Written informed consent was obtained from all participants prior to study entry.

#### Delphi Procedure

Preparations for this Delphi study began in December 2021. Delphi panelists were invited to participate in the Delphi study in February 2022. The first of five survey rounds began in March 2022, with the end of the study scheduled for August 2022. An additional short survey was conducted in response to a few questions that were raised during the peer review process. Originally, one month was planned per round, with 2 weeks of response time for the participants and 2 weeks for the analysis of the results and creating the next round’s survey. An online consensus conference was held between Rounds 3 and 4, as consensus on certain terms was difficult to reach and it was thought that verbal discussion may increase the chances of a consensus being achieved. Evaluation rules were adjusted or added in the course of the survey to facilitate the process (see below). A core team of the SC was responsible for identifying the main fasting terms and developing and pre-formulating their definitions for Round 1. A literature search was then conducted on PubMed to review the various definitions in circulation for these terms. These definitions were subsequently compared with the SC’s draft, adjusted as needed and finalized for the first round of the survey. Table S1 contrasts the definitions drafted by the SC for the first round with corresponding definitions from published literature. During each round, participants were asked to evaluate and modify each definition based on their expertise and professional opinion until either consensus was reached or the fifth round was completed. Participants could also suggest terms to be excluded from the consensus process or added throughout the process. The results and comments of each survey determined the design of the next questionnaire. Whenever consensus had not yet been achieved and changes or alternative definitions were proposed, the wording of the definitions was modified for the subsequent round based on the comments of the Delphi panelists.

After each round, as well as before and after the online consensus conference, a document containing all fasting terminology with the previously proposed definitions, the percentages of agreement and all expert comments, was created and made available to participants upon request in an anonymized form. These documents are displayed in Figure S4. For this purpose, each expert was assigned a fixed pseudonym with a “P” and a number.

No feedback was provided to participants between rounds, but all major findings for each term were presented in each subsequent questionnaire prior to a term’s re-evaluation. For this reason, all virtual surveys included the previously proposed definitions and the corresponding group responses. To keep the surveys as short as possible, only those expert comments that resulted in changes to the definitions were presented.

#### Survey Content and Scoring

The first page of each survey listed the evaluation rules. In the first survey, the subsequent five pages presented the original 19 fasting terms and their definitions formulated by the core team of the SC. The SC grouped the fasting terms into five categories, which were maintained throughout the process. The first category encompassed “terms concerning dietary and caloric restriction” and included the definitions *dietary restriction*, *starvation*, and *caloric restriction*. The second category covered “general terms concerning fasting” and contained the terms *fasting*, *water-only fasting*, *fluid-only fasting* and *dry fasting*. Category 3 was named “continuous fasting regimens” and incorporated *STF, PF/LTF* and *periodic fasting*. The fourth category was named “intermittent fasting regimens” and included proposed definitions for *IF, TRE* and *ADF*. The fifth and last category was called “specific fasting regimens” and included the definitions for *therapeutic/medically supervised fasting, Buchinger therapeutic fasting, FX-Mayr therapy/cure, FMD, religious fasting*, and *intermittent dry fasting*. On the last page of the first questionnaire, participants were asked to select from a list of terms those that they thought should be defined additionally in the next round of the questionnaire: *IER, CER, DER, alternate-day modified fasting (ADMF), sub-total fasting, zero-calorie diet* and *modified fasting*. If desired, a comment or definition proposal could be made under each of these terms. In addition, a maximum of two further terms could be suggested that were not included in the above list.

All subsequent questionnaires began with information relevant to the new round, e.g., references to adjustments in the evaluation rules or regarding the consensus conference. This section was followed, if applicable, by a section of terms on which consensus was reached; a section with terms on which consensus was reached in the last survey but which needed to be reassessed based on comments from participants; and a section with terms on which consensus had not yet been reached and which needed to be reassessed. The last questionnaire additionally asked for demographic data and conflicts of interest.

All rounds of questionnaires collected both quantitative and qualitative data. Proposed definitions were rated on an extended 6-point Likert scale (1 =“strongly agree”; 2 =“agree”; 3 =“neutral”; 4 =“disagree”; and 5 =“strongly disagree”; 6 =“This definition is irrelevant and should be excluded from the consensus process”). The sixth response option was offered to allow experts to directly point out definitions that they felt should not be part of the consensus process. If survey participants selected a response option from “3” to “5”, they were asked to provide their suggestions for improvement or an alternative definition. If “1”, “2” or “6” was selected, txhe experts could optionally justify their choice. The surveys also included detailed questions about some fasting terms and methods, which were intended to help further refine the corresponding definitions during the subsequent rounds. The answers to these questions were therefore specific to the respective fasting method and could not be answered using the Likert scale; instead, they included a selection of predetermined answer options or required a free-text response. An example of such a question is “Would you recommend the use of bowel/colon cleansing during *fluid-only fasting*?” with the response options: “No preference”; “No, I do not recommend bowel/colon cleansing”; “Yes, with sodium sulfate (Glauber’s salt) or magnesium sulfate (Epsom salt, bitter salts)”; “Yes, with colonic irrigation or enema”; “Yes, with other means, namely …”. All five rounds of surveys with all questions and answer options, the agenda and the written summary of the online consensus conference can be found in the supplement (Figure S1).

#### Evaluation Rules as Presented to the Expert Panel

1. A definition is accepted in case of agreement of ≥70% of participants (“strongly agree” or “agree”).
2. A definition is removed if ≥50% of participants choose “This definition is irrelevant and should be excluded from the consensus process”.
3. Suggested changes or new terms to be defined are taken into consideration for the next survey when they have been suggested by at least two participants.

Evaluation rule implemented from the second round onwards:

- In case you feel incapable of voting on one or more terms / methods because they don’t fall within your area of expertise, please choose the answer option: “*Due to my specialization, I am not familiar with this term / this method and prefer not to vote on it*”.
  ▶Only the votes for the answers “strongly agree - agree - neutral - disagree - strongly disagree - this definition is irrelevant and should be excluded from this consensus process” will be counted.

#### Evaluation rules implemented from the third round onwards

- For definitions to which the first and third rule apply, private emails are sent to the commenting participants to clarify details. If the modifications are still relevant to at least two participants after the email exchange, a definition with the desired modifications is created and offered for re-evaluation in the next questionnaire together with the initial definition.
- For definitions to which the first three rules do not apply, individual comments from participants are selected and adopted as modifications to the original definition. These modified definitions will then be presented for re-evaluation.
- Whenever two definitions had been proposed for the same term in the last questionnaire, but no clear leading definition emerged, all comments from all participants are taken into account, and an appropriately modified definition is offered for re-evaluation in the next round.
- For definitions for which no consensus seems foreseeable in the next round and / or for which the participants did not make any target suggestions for change, the SC proposes its own changes to the definitions which are presented for evaluation in the next round.
- Minor additions to definitions for which no consensus seems foreseeable in the next survey (e.g. “what type of water should be consumed in water-only fasting?”) are not included in the final definition of the term but will be mentioned in the explanations of the term in the subsequent publication.
- Terms that have so far been defined separately, but which, according to the participants, can be subsumed under one definition, are grouped together (e.g., “continuous energy restriction /daily energy restriction” and “caloric restriction”).
- If no consensus is reached for a term by the end of the fifth round, the last version that reached consensus will be used. If there was no previous consensus, this result will be described in the publication.

#### Evaluation rule for the online consensus conference

If there was no consensus on the definition before the discussion, a consensus of ≥70% had to be reached in the discussion to adopt the proposed definition. If there was already consensus on the definition in the written surveys but at least two participants proposed the same changes, these terms and proposed changes were discussed. Subsequently, if a consensus of ≥50% was reached in the discussion group, the definition (with or without the changes) was accepted.

### Data Collection, Management, and Analysis

The questionnaires were hosted on the online survey platform LimeSurvey (https://www.limesurvey.org/en/). Invitations with a personalized link to access each survey were sent via the survey website. Each questionnaire was checked for any technical problems and clarity of wording before launch. Reminder emails were sent to non-responders on Day 7, Day 13, and one week after the scheduled end of each round. A digital survey was chosen to ensure that experts from around the world could participate in the study.

Quantitative and qualitative data analysis was conducted at the end of each survey round. Only fully completed questionnaires were included in the evaluation of the individual rounds. Based on the evaluation rules, the terms were then either accepted or adapted for re-evaluation. Quantitative analysis was first conducted using the integrated statistical program of the survey platform. Ratings for all definitions and response options to additional questions were tabulated as percentage of agreement among respondents in each survey round. The additional answer option “Due to my area of expertise, I am not familiar with this term/method and prefer not to vote on it”, which was added in the second round and applicable to all subsequent rounds, was reported similarly as a percentage. All votes for this response option were subtracted from the denominator in Excel, so the final results for all quantitative outcomes were adjusted for these votes.

### Proposed systematization of terminology

When setting up the questionnaire for the survey, the SC predetermined five categories for a clearer arrangement of the questionnaire. These were the following: 1. “Terms concerning dietary and caloric restriction”, 2. “General terms concerning fasting”, 3. “Continuous fasting regimens”, 4. “Intermittent fasting regimens” and 5. “Special fasting regimens”. While evaluating the results, it became clear that these categories did not adequately fit the final definitions. For this reason, we decided to rearrange the categories according to the main characteristics delineated in the final definitions. This brought about the following five groups: 1. “General terms concerning nutritional restriction”; 2. “Fasting regimens categorized by type”; 3. “Fasting regimens categorized by duration”; 4. “Fasting regimens categorized by the aspect of repetition”; and 5. “Fasting regimens categorized by motivation”. The former and current categories, including the respective definitions, are shown in Table 3.

**Figure 3.**
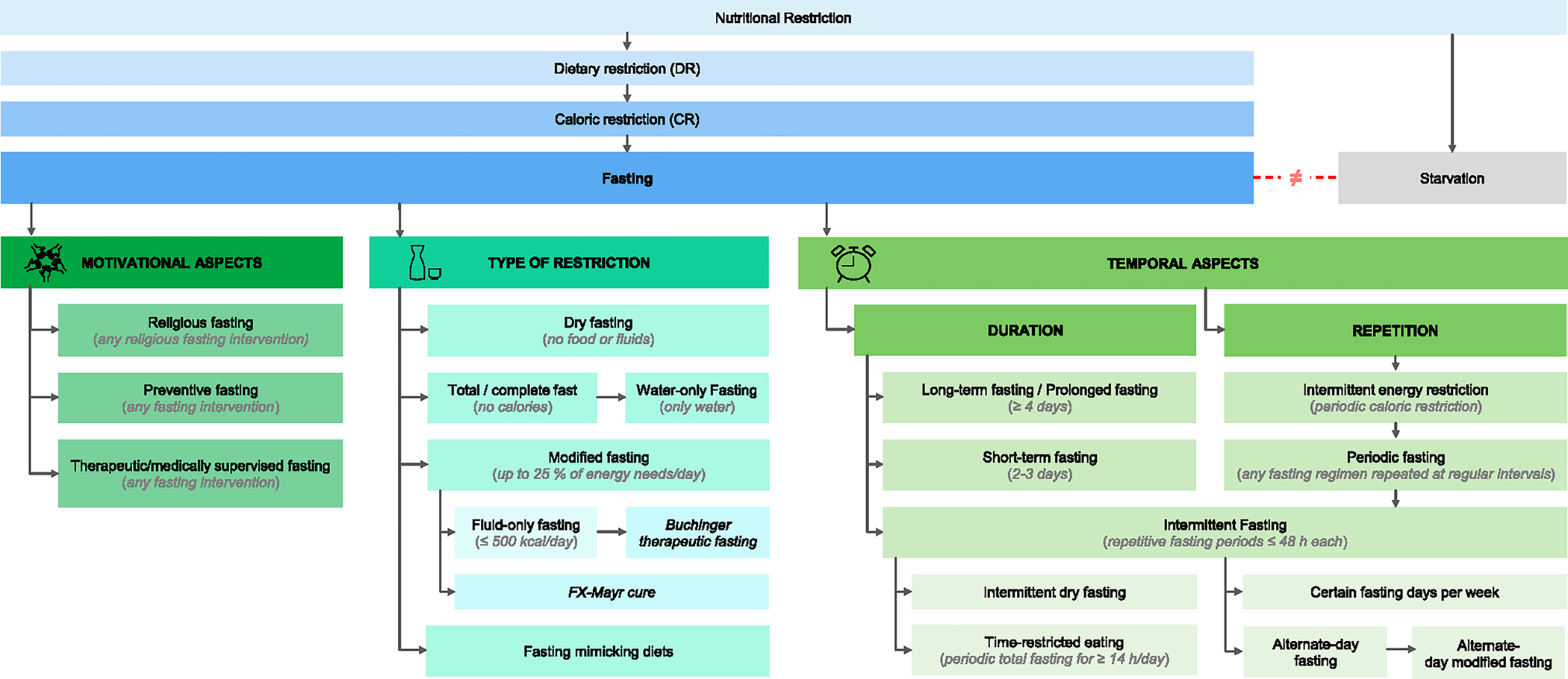
Classification of terminology All defined terms are listed here in distinct categories.

As illustrated in Table 3, *fasting* was moved to the more general terms, as it was defined as an umbrella term by the end of the process. The categories 2 to 5 were completely revised to represent the main characteristic of the fasting definitions they contain, focusing on the dietary intake during the fasting timeframe, the duration of the fast, whether it is meant to be repetitive, and for what reasons it is followed or prescribed. Figure 3 visualizes the classification of all defined terms in these categories.

## Supplementary Material

Supplementary materials

## Figures and Tables

**Table 1.** Demographics of the 38 panelists

	*N*	%

*n*	38	100
Age (mean [SD])	53	(12)

Gender		

Female	13	34
Male	24	63
Non-binary	1	3

Highest degree^a^		

Medicine	19	50
Nutritional sciences	23	60
Health sciences	6	6
Biology	6	6
Molecular medicine	2	5
Molecular biology	2	5
Other/additional^b^	18	47

Primary occupation		

Researcher	19	50
Clinician	8	21
Clinician scientist	10	26
Other	1	3

Is fasting the main focus of your work?		

Yes	21	55
No	17	45

Years working on the subject of fasting		

1–5	1	3
5–10	10	26
10–15	8	21
15 or more	18	47
Other	1	3

Continent of origin		

Asia	2	5
Australia	2	5
Europe	20	53
North America	13	34
Africa	1	3

Continent of employment		

Asia	2	5
Australia	1	3
Europe	16	42
North America	18	47
Africa	1	3

*N*, population; *n*, sample size; SD, standard deviation.

a More than one option could be selected.

b Nutritional or genetic epidemiology, physiological chemistry, medical oncology, endocrinology, circadian biology, (exercise) physiology, kinesiology, chiropractic/osteopathy, neuroscience/chronobiology, theology, and physics.

**Table 2.** Fasting terms generated in the consensus process

Definitions	Survey	Cons. (%)	*p*/N*

Terms concerning dietary and caloric restriction			

Dietary restriction (DR) comprises of continuous or intermittent restrictions in caloric intake and/or specific macronutrients and/or restraints of food or food and fluid intake within a specified time frame. DR thus includes: all types of caloric restriction; fasting regimens such as short-term, long-term, and periodic fasting, intermittent fasting, time-restricted eating, water- and fluid-only fasting, therapeutic fasting and fasting-mimicking diets; and diets with restrictions of specific macronutrients namely proteins, carbohydrates, or fats.	2	78	25/32
Caloric restriction (CR) describes a reduction in energy intake below the total amount of calories that would be needed to maintain a person's current body weight, without causing malnutrition. If CR is done daily, it can also be referred to as continuous energy restriction (CER) or daily energy restriction (DER). *Comment: the degree of caloric restriction should be decided individually depending on the person’s age, sex, body composition, activity level, occupation, goal, and planned duration of CR.*	4	73	24/33
Starvation describes a catabolic process that occurs when the body's reserves are exhausted after a prolonged period of insufficient energy and nutrient supply. Starvation can lead to serious health impairments, organ failure, and death.	2	94	31/33
General terms concerning fasting			
Fasting refers to a voluntary abstinence from some or all foods or foods and beverages for preventive, therapeutic, religious, cultural, or other reasons.	2	85	28/33
Water-only fasting refers to a fasting regimen where only water is consumed for a certain period of time.	2	72	23/32
The term total fast, or complete fast, refers to a fasting regimen where no calories are consumed for a certain period of time. *Comment: total/complete fasting can be equated with water-only fasting, but it may additionally include tea or other non-caloric beverages*.^a^	4	94	31/33
Dry fasting refers to a fasting regimen during which a voluntary abstinence from all foods and beverages, including water, is practiced for a certain period of time.	2	88	28/32
The term modified fasting refers to limiting energy intake to typically up to 25% of energy needs on modified fasting days. *Comment: modified fasting regimens can be adapted to specific clinical settings and indications, allowing for different complementary or supportive therapeutic interventions. Examples of modified fasting regimens are alternate-day modified fasting, fasting practiced on 2 separate or consecutive days per week and fluid-only fasting.*	5	86	25/29
Fluid-only fasting refers to a modified fasting regimen whereby only beverages are consumed for a certain period of time. water and unsweetened herbal tea may be consumed *ad libitum.* Clear vegetable broth, vegetable, and/or fruit juices can be consumed up to a total of 500 kcal/day. Ultra-processed fluids should not be consumed. *Comment: this fasting regimen includes traditional fasting regimens that use various broths, gruel, or decoctions, such as the traditional German gruel fasting.*	5	71	20/28

Continuous fasting regimens			

Short-term fasting (STF) refers to fasting regimens with a duration of 2–3 days.^a^	4	81	26/32
Prolonged fasting (PF), also called long-term fasting (LTF), refers to fasting regimens lasting ≥4 consecutive days.^a^	4	81	26/32
Periodic fasting refers to any fasting regimen that is repeated at regular intervals (periods), such as every day, every week, or every several months. *Comment: according to this definition, periodic fasting would include intermittent fasting regimens.*	4	81	26/32

Intermittent fasting regimens			

Intermittent energy restriction (IER) includes periods of caloric restriction alternating with periods of *ad libitum* eating. as such, IER includes fasting regimens like intermittent fasting (IF) and time-restricted eating (TRE).	2	81	26/32
Intermittent fasting (IF) refers to repetitive fasting periods lasting up to 48 h each. IF includes fasting regimens of 1 fasting day per week, 2 separate or consecutive fasting days per week, alternate-day fasting (ADF), and time-restricted eating (TRE).	3	87	26/30
Time-restricted eating (TRE) is a dietary regimen in which food intake and the consumption of caloric beverages is restricted to a specific period of time during the day, resulting in a daily fasting window of at least 14 hours. There is no explicit limit on energy intake during eating hours.	4	88	28/32
Alternate-day fasting (ADF) refers to alternating a day of eating *ad libitum* and a day of water-only fasting.	4	94	31/33
Alternate-day modified fasting (ADMF) refers to alternating a day of eating *ad libitum* and a day of modified fasting.	5	96	27/28

Special fasting regimens			

Therapeutic fasting refers to any fasting regimen that is applied as a therapeutic intervention. *Comment: therapeutic fasting interventions are individually tailored to a person’s age, sex, body composition, physical activity level, occupation, goal, and planned duration of fasting.* Medically supervised fasting refers to any fasting regimen that is applied as a therapeutic intervention by a trained physician or similar credentialed healthcare provider.	4	91	29/32
Preventive fasting refers to any fasting regimen that is applied as a preventive intervention.	5	82	23/28
Buchinger therapeutic fasting is a fluid-only fasting regimen, allowing for a maximum of 500 kcal/day and lasting at least 5 days, practiced for the prevention or treatment of diseases as well as to support one's individual health, taking into account a person's medical, psychosocial, and spiritual dimensions. It is usually accompanied by bowel/colon cleansing procedures and preceded and followed by a few days of a calorie-restricted, easily digestible diet.	2	91	21/23
FX Mayr therapy or FX-Mayr cure refers to a regimen containing elements of water-only fasting, a very low-calorie diet with a training of “proper chewing” in order to help individuals (re-)gain their sense of satiety, and an easily digestible diet toward the end of the treatment. The dietary intervention is accompanied by bowel cleansing procedures and manual treatments focusing on the abdominal region.	4	100	22/22
A fasting-mimicking diet (FMD) specifies any diet specifically composed to induce the metabolic effects of fasting while allowing for a potentially higher caloric intake, including solid foods. It usually refers to a plant-based, calorie-restricted diet with a maximum of approximately 1,000 kcal/day that lasts 3–7 days. FMDs are usually relatively low in refined sugars and starch, low in protein, and high in plant-based fats. *Comment: the exact amount of calories, macronutrient composition, duration, and frequency of use needs to be decided individually. FMD meals can consist of packaged products or be freshly prepared.*	5	75	21/28
Religious fasting refers to any fasting regimen that is undertaken as part of a religious practice. *Comment: religious fasting thus involves practices such as dry fasting on specific days of the year up to 25 h at a time (E.G., Jewish Tradition, The Church of Jesus Christ of Latter-Day Saints); intermittent dry fasting (E.G. Ramadan fasting, Bahá’í Fasting); time-restricted eating (E.G., Buddhism); and diets restricting certain foods (E.G., Christian Orthodox Traditions, Daniel Fast) if more broadly defined. typically, religious fasting includes spiritual activities to improve cognitive function and well-being.*	4	94	30/32
Intermittent dry fasting (IDF) refers to intermittent fasting regimens that involve abstaining from food and fluid intake during fasting hours. Most commonly, they range from 9 to 20 h.	3	85	23/27

Cons., consensus; p, number of people who voted “strongly agree” and “agree” during the round in which consensus was achieved; N*, total number of people who voted on this term in the round in which consensus was achieved.

a Definitions adapted during the peer review process.

**Table 3.** Proposed systematization of terminology

Prior to the start of the study	After the study

Terms concerning dietary and caloric restriction	General terms concerning nutritional restriction

Dietary restriction	dietary restriction
Caloric restriction	caloric restriction
Starvation	starvation
-	fasting

General terms concerning fasting	Fasting regimens categorized by type

Fasting	water-only fasting
Water-only fasting	total/complete fast
Total/complete fast	dry fasting
Fluid-only fasting	modified fasting fluid-only fasting Buchinger therapeutic fasting FX Mayr therapy/cure
Dry fasting	fasting-mimicking diet
Modified fasting	-

Continuous fasting regimens	Fasting regimens categorized by the aspect of duration

Short-term fasting	short-term fasting
Prolonged/long-term fasting	prolonged/long-term fasting
Periodic fasting	-

Intermittent fasting regimens	Fasting regimens categorized by the aspect of repetition

Intermittent fasting	periodic fasting
Time-restricted eating	intermittent fasting
Intermittent energy restriction	time-restricted eating
Alternate-day fasting	intermittent energy restriction
Alternate-day modified fasting	intermittent dry fasting
-	alternate-day fasting
-	alternate-day modified fasting

	Fasting regimens categorized by motivation

-	therapeutic/medically
-	supervised fasting
-	preventive fasting
-	religious fasting

Special fasting regimens	

Therapeutic/medically supervised	-
Fasting	-
Preventive fasting	-
Buchinger therapeutic fasting	-
FX Mayr therapy/cure	-
Fasting-mimicking diet	-
Religious fasting	-
Intermittent dry fasting	-

## Highlights

- Thirty-eight panelists from five continents participated in this consensus process
- First panel uniting experimental and clinical experts in medical and religious fasts
- Twenty-four terms were defined in five online surveys and one live conference
- Fasting defined as voluntary abstinence from some or all foods or foods and beverages

## Abbreviations

ADF: Alternate-day fasting
ADMF: Alternate-day modified fasting
AMPK: 5’ Adenosine Monophosphate-activated Protein Kinase
BDNF: Brain-derived neurotrophic factor
CER: Continuous Energy Restriction
DER: Daily Energy Restriction
FOXO: Class O of forkhead box transcription factors
FMD: Fasting-Mimicking Diet
IER: Intermittent Energy Restriction
IF: Intermittent Fasting
LTF: Long-Term Fasting
mTOR: mammalian Target Of Rapamycin
NRF2: nuclear factor erythroid 2-related factor 2
PF: Prolonged Fasting
PKA: Protein kinase A
STF: Short-Term Fasting
TRE: Time-Restricted Eating
