# Supplementary material for: International consensus on fasting terminology
Source: Cell Metab. Author manuscript; Available in PMC 2024 Oct 25. (PMC11504329; doi:10.1016/j.cmet.2024.06.013)
Supplement: Supplementary materials [file NIHMS2019353-supplement-Supplementary_materials.pdf]

Cell Metabolism, Volume *36*

Supplemental information

International consensus on fasting terminology

Daniela A. Koppold, Carolin Breinlinger, Etienne Hanslian, Christian Kessler, Holger Cramer, Anika Rajput Khokhar, Courtney M. Peterson, Grant Tinsley, Claudio Vernieri, Richard J. Bloomer, Michael Boschmann, Nicola L. Bragazzi, Sebastian Brandhorst, Kelsey Gabel, Alan C. Goldhamer, Martin M. Grajower, Michelle Harvie, Leonie Heilbronn, Benjamin D. Horne, Spyridon N. Karras, Jost Langhorst, Eva Lischka, Frank Madeo, Sarah J. Mitchell, Ioannis-Eleemon Papagiannopoulos-Vatopaidinos, Maria Papagiannopoulou, Hanno Pijl, Eric Ravussin, Martha Ritzmann-Widderich, Krista Varady, Lilian Adamidou, Melika Chihaoui, Rafael de Cabo, Mohamed Hassanein, Nader Lessan, Valter Longo, Emily N.C. Manoogian, Mark P. Mattson, J. Brent Muhlestein, Satchidananda Panda, Sousana K. Papadopoulou, Nikolaos E. Rodopaios, Rainer Stange, and Andreas Michalsen

Table S1 Collection of key fasting terms with their pre-formulated definitions for Round 1 compared to definitions found through a literature search in PubMed. Related to STAR Methods.

Term	Proposed definitions – First draft	Definitions in the literature
Dietary Restriction	Dietary restriction implies a restriction in caloric intake and / or specific micronutrients and / or restraints in time frames of food intake. Thus, it includes all forms of caloric restriction (CR), Water-only fasting (WF), fasting-mimicking diets (FMD), restriction of specific macro-nutrients such as proteins, fats or carbohydrates, ketogenic diets (KD), short- and long-term fasting (STF/LTF), prolonged fasting (PF), periodic fasting (PF), Intermittent Fasting (IF) and Time Restricted Eating (TRE)/ Time restricted Feeding (TRF).	**Katewa & Kapahi, 2010**: Dietary restriction (DR) is defined as a reduction of particular or total nutrient intake without causing malnutrition. Dietary restriction in this broad sense includes caloric restriction (CR), in which total food intake is reduced, as well as studies involving the restriction of major dietary components (protein, lipid or carbohydrates) or temporal variations of food intake (intermittent fasting).<br>**Trepanowski et al., 2011:** restriction of one or more components of intake (typically macronutrients) with minimal to no reduction in total caloric intake - is another alternative to CR<br>**Lee & Longo, 2016:** DR represents a broader scope of dietary interventions that encompass those with specific macronutrient and feeding pattern restriction including short-term starvation, periodic fasting, fasting-mimicking diets, intermittent fasting, normo-caloric diets with planned deficiencies (in particular: macronutrients: proteins, carbohydrates, fats) and time-restricted feeding<br>**Choi, Lee & Longo, 2017:** The major DR regimens include caloric restriction, intermittent fasting, time-restricted feeding, restriction of specific macronutrients, ketogenic diets, and periodic fasting or fasting-mimicking diets.<br>**Frieling, J. & Roeder, T., 2020:** DR is a generic term encompassing any dietary regimen that differs from a conventional diet in at least one major respect. “DR” can signify a very broad range of dietary regimens that differ from conventional diets with respect to at least one major component. CR can be understood as a particular subset of DR. Because a plethora of different nutritional regimens can be summarized under the umbrella term “DR,” its definition is fairly vague, and uncertainty in the meaning of the term is further increased by differing understandings of what a conventional, normal, or control diet is. DR includes starvation, caloric and macronutrient restriction and time restricted food intake<br>**Fontana et al., 2021:** DR is a chronic or intermittent reduction of food intake without malnutrition.DR can be achieved by chronically reducing food intake or by changing meal frequency and timing.
Caloric restriction (CR)	Caloric restriction refers to 20-40% restriction of calories below *ad libitum* levels	**Hursting, 2003:** CR is also referred to as undernutrition without malnutrition. The CR dietary regimen provides essential nutrients and vitamins but limits the total energy intake of the animal (usually by 20%–40% relative to Ad Libitum-fed controls). In

		actuality, adequate nutriture is designed into CR regimens to avoid the confounding effects of malnutrition, and modest calorie decreases of 20%–30% relative to an AL diet can be equated to a normal, healthy level of intake<br>**Mair & Dillin, 2008:** DR is designed to induce "undernutrition without malnutrition" and the food intake this represents is usually 30-40% less than ad libitum levels.<br>**Canto & Auwerx, 2009:** CR is usually defined as a moderate (normally, 20-40%) reduction in caloric intake as compared with an ad libitum diet, without compromising the maintenance of all essential nutrients<br>**Varady, 2011:** decreasing energy intake by 15-60% of baseline needs every day<br>**Omedei / Fontana, 2011:** CR is often loosely used to describe any reduction in energy intake, even if the baseline energy intake is excessive (i.e., overweight/obese individuals) and it is being reduced to lower levels. In the context of the aging/ longevity studies the term CR should refer only to a state in which energy intake is sufficiently low to achieve or maintain a low-normal body weight status (i.e., body mass index < 21 kg/m2) without causing malnutrition (i.e., adequate intake of proteins and micronutrients)<br>**Redman & Ravussin, 2011:** Calorie restriction (CR), a dietary intervention that is low in calories but maintains proper nutrition<br>**Longo & Mattson, 2014:** Daily caloric intake is reduced chronically by 20-40 % but meal frequency is maintained<br>**Barnosky et al., 2014:** Reducing energy intake every day by 20-50% of needs (citing Omedei & Fontana – die machen aber in ihrem Review keine Angabe mit %)<br>**Anton et al., 2018:** continuous reduction in caloric intake without malnutrition<br>**Di Francesco et al., 2018:** CR without malnutrition can be accomplished by chronically reducing energy intake by 15 to 40% from AL conditions, while maintaining adequate intake of vitamins and minerals.<br>**Frieling, J. & Roeder, T., 2020:** CR involves a lower energy intake compared with ad libitum feeding.<br>**Pifferi et al., 2019:** Caloric restriction (CR) is a nutritional intervention consisting in eating less without malnutrition<br>→ most scientists seem to agree on the fact that CR is restriction **without** causing malnutrition (as starvation would)
Continous /Daily Energy Restriction (CER)/(DER)		**Harvie et al., 2013:** DER = overall 25 % daily energy restriction.<br>**Rynders et al., 2019** CER **=** daily energy deficit of 500 or 700kcal, or a 30% restriction from baseline energy requirements – for obesity treatment (= alternative to IER!)<br>→ according to **Cioffi et al., 2018**: CER = conventional weight-loss diets

Starvation	Starvation refers to a state of unvoluntary and/or unconscious abstinence from foods and/or beverages. When used in the context of animal studies, the term fasting usually refers to starvation. We propose starvation as the more exact wording in this context.	**Longo & Mattson, 2014:** a chronic nutritional insufficiency that is commonly used as a substitute for the word fasting, particularly in lower eukaryotes, but that is also used to define extreme forms of fasting, which can result in degeneration and death.<br>**Phillips, 2019:** a state of chronic nutritional insufficiency which is neither voluntary nor controlled, and which may culminate in organ failure and death
Fasting	Fasting refers to a voluntary and conscious abstinence from some or all foods and/or beverages for therapeutic, spiritual, or political reasons *for a certain length of time.*	**Wilhelmi de Toledo et al., 2002 - updated guidelines on Fasting Therapy:**<br>• Fasting is the voluntary abstinence from solid food and stimulants (caffeine, nicotine) for a limited period of time.<br>• The process of fasting involves the person in all its dimensions: body, soul and spirit<br>• the ability to meet the body's requirements for macro- and micronutrients during a limited period of either shortage or absence of food, by making use of the body's energy reserves, without endangering health<br>• Fasting as biological necessity has evolved into voluntary fasting traditions with specific ethno-cultural modifications. It was further developed either in religious / spiritual contexts or for medical therapeutic purposes<br>**Longo & Mattson, 2014:** in humans fasting is achieved by ingesting no or minimal amounts of food and caloric beverages for periods that typically range from 12 hrs to three weeks.<br>**Phillips, 2019:** Fasting may be defined as a voluntary abstinence from food and drink for specified, recurring periods of time, with the fasting periods typically ranging from 12 hrs to 3 weeks in humans. (Citing Mattson, 2018, Longo & Mattson, 2014, etc.)
Sub-total fasting	Sub-total fasting refers to a fasting regimen that allows max. 500 kcal in the form of beverages only. As one main idea is to decelerate digestive processes this fasting regimen explicitly excludes solid foods and even intake of dietary fibers is to be avoided.	**Huber et al., 2005:** bezieht sich mit sub-total fasting auf Buchinger-Heilfasten: 7.5 days of fasting (180 h). Individuals are permitted to drink vegetable or fruit juices without additives, containing 15-50 kcal per 100 ml rom carbohydrates with a maximum daily intake of <300 kcal/d; water intake is unlimited. Normal daily activity, as before fasting, is maintained but individuals are discouraged from extreme physical exercise.
Water-only fasting	Water-only fasting refers to a fasting regime, where only water (and sometimes tea) is consumed for a certain period of time, usually ≥ 2 days..	**Goldhamer et al., 2001:** Water-only fasting is the complete abstinence from all substances – food, tea, juice, noncaloric beverages, etc. with the sole exception of distilled water ad libitum (with a minimum of 40 ounces daily). Patients' activities are restricted, because even moderate activity during a water-only fast can double energy use.<br>**Goldhammer, 2002:** only distilled water ad libitum is allowed (minimum intake 40 ounces / day = 1200 ml

		**Finnell et al., 2018:** Zero-calorie-diet, initially used for weight reduction in the 1960s/1970s. Complete abstinence of substances except for pure(distilled) water for at least 2 consecutive days.<br>**Phillips, 2019:** Water-only fasts omit all calorie intake but provide adequate hydration and can therefore be extended out to several days, weeks, or even months, provided that adequate salt and other micronutrients are maintained.<br>Fluid-only fasts additionally permit calorie-free fluids, such as tea and black coffee, which can help maintain energy and suppress the transient waves of hunger that may occur in some people. Both types of fasts should aim for a minimum of 2–2.5 L of water or fluid intake per day, and a multivitamin may be added to provide micronutrients.<br>**Oglodek, 2021b:** Water-only fasting (WF) is an absolute cessation of food consumption while consuming water at libitum. When undertaking WF, it is recommended that an individual drink 2-3 liters of fluid daily, especially in the form of mineralized water. This partially covers the body's needs to keep the balance of particular minerals.<br>**Letkiewicz et al., 2021:** individuals drink ad libitum moderately mineralized water
Dry fasting	Dry fasting refers to a fasting regimen, where an abstinence from all foods and beverages is practiced for a certain period of time. *In publications until now this has been described as feasible for young, healthy individuals for up to 5 days.*	**Papagiannopoulos-Vatopaidinos et al., 2020:** Dry fasting (DF), also called food and water deprivation, is defined as the abstinence from any food or hydration.
Zero-Calorie-Diet		https://www.ugb.de/richtig-fasten/bedeutung-fastens-in-behandlung-von-uebergewicht/: „Die größte Definitionsverwirrung entstand in der medizinischen Welt in den 70er Jahren, als die stationäre Nulldiät (bis 249 Tage!) zur Bekämpfung von Übergewicht praktiziert wurde - damals ohne Bewegung, Schulung oder Verhaltenstherapie. Wegen hoher Rückfallquoten und einiger Zwischenfälle wurde sie aufgegeben. Es entstand danach das ambulante, proteinmodifizierte Formulafasten. Wegen einer offenbar verkehrten Komposition forderte die *Liquid Protein Diet* den Tod durch akute Herzarhythmie bei 17-58 Adipösen, die dieses ambulante Formula-Fasten mehr als drei Monate in eigener Regie durchgeführt hatten. Seitdem wurde das Fasten mit der Nulldiät und mit der *Liquid Protein Diet* verwechselt.<br>Wenn jemand aufhört, feste Nahrung zu sich zu nehmen, ohne Betreuung, im alltäglichen Stress, isoliert, ohne Bewegung, Darmhygiene und psychotherapeutische Betreuung, wenn diese Person keine Phase der Nahrungswiederzufuhr durchführt, dann hat sie tatsächlich eine Crash-Diät und kein Fasten gemacht, und Crash-Diäten sind in der Therapie der Adipositas obsolet.“<br>**Stange & Leitzmann Buch “Ernährung und Fasten als Therapie“, S. 184:**

		Vollständige Unterbrechung der Nahrungszufuhr (teilweise über Wochen bis Monate) mit dem Zweck der Gewichtsreduktion bei massiver Adipositas. Stationär, ohne Hilfsmethoden, Schulungsprogramme oder Nachbetreuung, zeigt sich nach Beendigung eine hohe Rückfallquote bei hohen Kosten. Sie wird daher im Krankenhaus nicht mehr praktiziert, obwohl die Ergebnisse teilweise positiv waren.<br>**2002 Leitlinien zur Fastentherapie:** Unterbrechung der Nahrungszufuhr (Null Nahrungsenergie über Wochen bis Monate) mit dem Zweck der Gewichtsreduktion bei massiver Adipositas (Liebermeister et al., 1989). Sie wurde stationär im Krankenhaus (hohe Kosten), ohne Hilfsmethoden und Schulungsprogramme durchgeführt. Sie wird heute nicht mehr praktiziert
Short- term fasting (STF)	Short-term fasting refers to any fasting or fasting-mimicking regimen that has a duration between 2-4 days.	**Michalsen et al., 2003:** 7-10 days modified fast.<br>**Safdie et al., 2009:** 48 h fast<br>**Browning et al., 2012:** 48 h fast – zero calories consumed<br>**Bauersfeld et al., 2018:** 60 h fast – zero calories consumed<br>**Liebscher et al., 2020.:** we use the term STF in our study to define a subtotal caloric restriction of 60–72 h, as we did in a previous publication
Prolonged fasting (PF) and Long-term fasting (LTF)	Fasting durations for ≥ 5 days are referred to as prolonged fasting or long-term fasting. These terms can be used interchangeably.	**Safdie et al, 2009:** Caste study reports:<br>• 180 hrs fast – consuming only water and vitamins (fast for 140 hrs pre-chemotherapy and 40 hrs after chemotherapy); same patient later on did a 144 hrs combined with another chemotherapy cycle.<br>• 72 hrs prior to chemo-, 51 h post-chemotherapy;<br>• 48 hrs prior, 56 hrs post-chemotherapy<br>**Cheng et al., 2014:** fasting lasting 48-120 h (2-5 days)<br>**Li et al., 2017:** Prolonged Fasting: fast > 5 days – study did Buchinger method: 2 pre-fasting days with moderate caloric restriction (1200 kcal and low salt), followed by 7 modified fasting days (300 kcal: unlimited amounts of water, herbal tea (no black or green tea), 200 ml fruit juice and small standardized quantities of light vegetable soup)) and stepwise re-introduction of ordinary food items over 3 days.<br>**Bak et al., 2018:** 72 h fasting - zero calories consumed<br>**WIlhelmi de Toledo et al., 2020:** Long-term Fasting: food abstinence from 2-21 days or more during which no or minimal amounts of calories, up to 200-250 kcal/days are given within appropriate schedule
Periodic fasting (PF)	Periodic fasting refers to any fasting or fasting-mimicking regimen of two or more consecutive days of fasting that is done periodically; this can range from every day or week to every several months.	**Longo & Mattson, 2014:** Periodic fasting (PF) lasting three days or longer every 2 or more weeks.

		**Mattson et al., 2017 / Rynders et al.2019:** periods of fasting or fasting mimicking diets lasting from 2 to as many as 21 or more days.<br>Examples of PF:<br>- Mattson et al. (2018): e.g. 5 day diet providing 750-1100 kcal/d<br>- Brandhorst et al. (2015): 4-5 day FMD / Choi et al. (2016): 7 days of FMD<br>- Safdie et al. (2009): 2-5 days of water only fasting with chemotherapy cycles<br>**Anton et al., 2018:** Fasting only 1 or 2 days / week ad consuming food ad libitum on 5 or 6 days per week<br>**Phillips, 2019:** typically refers to extended fasting periods lasting from 2 days to 3 weeks in duration.<br>**Yang et al.,2021 citing Cioffi et al, 2018:** (though Cioffi's paper is a meta-analysis and doesn't give any extra definition of PF!): A circular weekly eating pattern that consists of fasting 1 to 2 days a week (burning 25% or less of the calories required) and eating freely the rest of the week on a 6:1 or 5:2 scale
Intermittent Energy Restriction (IER)/ Intermittent Calorie Restriction		**Varady, 2011:** Intermittent CR regimens generally involve a 'feed day', where food is consumed ad libitum over a 24-h period, alternated with a 'fast day', where food intake is either completely restricted or partially reduced over 24 h. This form of intermittent CR is also referred to as alternate day fasting. Other forms of intermittent CR, which involve 2 to 4 d of fasting alternated with 2 to 4 d of ad libitum feeding, have also been implemented.<br>**Davis et al., 2015**: IER involves a period of energy restriction alternated with a period of unrestricted or minimally restricted dietary intake. Various terms are currently used for this style of diet, including 'alternate-day fasting'5 and '5:2 diet'. For the purposes of this review the dietary strategy is referred to as IER. The design of IER protocols vary, with some studies employing an alternate-day method and others implementing an alternating set of days, such as 5 'feed' days followed by 2 'fast' days. The core concept of IER is that energy restriction is alternated with minimal dietary intervention, making IER different from the usual ongoing DER.<br>**Harvie & Howell, 2016:** IER comprises periods of marked ER or total fasting interspersed with periods of normal eating<br>**Harvey, Howell & Harvie, 2018:** IER consists of periods of a marked energy restriction, typically either 60%–75% reduction below predicted energy requirements for 2 days each week, with 5 days of baseline intake or alternating days of 75% energy restriction below predicted energy requirements and normal eating<br>**Antoni et al., 2018**: Short period of substantial (>70%) energy restriction interspersed with normal eating. → used 5:2 diet as IER pattern for their study

		**Rynders et al., 2019**: IER is one alternative dietary weight-loss strategy to Continuous Energy Restriction and can be defined as prolonged fasting interval between meals. Rynders defines 2 categories (reasoning see below in the definitions for IF and TRF):<br>1. **Intermittent Fasting (IMF)**<br>2. **Time Restricted Feeding (TRF)**
Intermittent Fasting (IF)	Intermittent fasting refers to relatively brief, repetitive fasting periods. It includes fasting regimens of one day per week (1:6), two days per week (5:2)- be they separate or in a row-, alternate day fasting (ADF) and time-restricted eating (TRE).	**Rynderes et al., 2019 via Mattson et al., 2017:** IF is used as the umbrella term to define "eating patterns in which individuals go extended time periods (e.g. 16-48 h) with little or no energy intake, with intervening periods of normal food intake on a recurring basis": They further "distinguish studies of short-term frequent fasting periods from studies of less frequent but longer fasting periods" by using the term periodic fasting (PF) to refer to IMF regimens with periods of fasting lasting from 2 to as many as 21 or more days. The term time-restricted feeding (TRF) is used as a subcategory of IMF to describe "an eating pattern in which food intake is restricted to a time window of 8 h or less every day".<br>→ according to **Rynders et al.** (2019) putting TRF into IMF category is problematic bc TRF is different from a complete day of fasting or modified fast day (= individuals consume up to 25% of daily energy needs). In TRF energy intake is ad libitum!<br>→ Rynders suggests to better use umbrella term IER (with 2 distinct categories): TRF & Intermittent Fasting<br>**Anton et al., 2018:** variety of eating patterns in which no or few calories are consumed for time periods that can range from 12 h to several days on a recurring basis.<br>**Intermittent Fasting (IMF) – Rynders et al., 2019:** IMF diets include eating patterns with extended time periods (e.g. 16-48 h) with little or no energy intake (60-100% energy reduction), with intervening periods of ad libitum intake, on a recurring basis.<br>Variations include Alternate Day Fasting (ADF) – zero calorie intake on fast days, Alternate day modified fasting (ADMF) – (> 60% energy restriction on fast days), Fasting or modified fasting on 2 days per week (2DW), and Periodic Fasting (PF) – periods of fasting lasting 2 to as many as 21 or more days<br>**Phillips, 2019:** Fasting periods lasting longer than a day are often grouped under the broadly-used term "IF", the definition of which often varies depending on the source. it is probably best to reserve the term "intermittent fasting" for fasting regimes containing recurring fasting periods lasting 24-48 hours in duration. (most common IF regimes are ADF and 2 days per week fasting)
<u>Subcategory of IF:</u><br>***Alternate day fasting (ADF)***	Alternate day fasting refers to the alternation of a day of eating *ad libitum* and a day of either water-only fasting or a diet very low in calories.	**Barnosky et al., 2014:** Consists of a "fast day" (75% energy restriction) alternating with a "feed day" (adlibitum food consumption) → should rather be named AMDF)

	(most thoroughly studied IMF protocol in lab. Rodents)	**Patterson et al., 2015**: (Complete) Alternate day fasting involves “fasting days” in which no energy-containing foods or -beverages are consumed alternating with days where foods and beverages are consumed ad libitum.<br>**Difrancesco et al., 2018:** 24-h water fast without solid food followed by a normal feeding period of 24 h.<br>**Yang et al, 2021:** A circular diet requires fasting for a day (consumption of no calories) and then eating freely for a day
<u>Subcategory of IF:</u><br>***Alternate-modified-day fasting (AMDF)***		**Modified Fasting Regimes – Patterson et al., 2015:** Modified regimens allow for the consumption of 20–25% of energy needs on scheduled fasting days. This regimen is the basis for the popular 5:2 diet (by Spencer), which involves severe energy restriction for 2 non-consecutive days a week and ad libitum eating the other 5 days.<br>**Difrancesco et al., 2018:** Participants consume very few calories one day (e.g., 25 % of usual intake) followed by a day without restrictions.<br>**Yang et al., 2021:** circular feeding pattern that requires fasting (consumption of 20-25% of energy needs) for a day, and then eating freely for a day
<u>Subcategory of IF (?):</u><br>***Time Restricted Eating (TRE)/ Time restricted Feeding (TRF)***	Time-restricted eating or, when animals are meant, time-restricted feeding, is a dietary regimen where food intake is restricted to a certain time frame per day (usually between 4-10 h daily), whereas there is no explicit limit to energy intake during eating / feeding hours.	**Rothshild et al., 2014:** TRF allows individuals to consume ad libitum energy intake within a set window of time (3-4, 7-9 or 10-12 h), which induces a fasting window of 12-21 h per day<br>**Mattson et al., 2015:** food intake is restricted to a time window of 8 h or less every day.<br>**Tinsley & La Bounty, 2015:** involves following the same eating routine each day, with a certain number of hours designated as the fasting window and the remaining hours as the feeding window<br><u>(info:</u> **Patterson et al., 2017:** time-restricted feeding research in animals highlights potential importance of synchronizing intermittent fasting regimes with daily circadian rhythms → mice can develop T2D and obesity with high-fat-diet eaten throughout night and day)<br>**Patterson et al., 2018:** Allows **ad libitum** energy intake within specific time frames, inducing regular, extended fasting intervals<br>**Difrancesco, 2018:** Refers to daily limitations in the timing of food intake, spanning from 3 to 12 hours, without reduction in caloric intake.<br>**Rynders et al, 2019.:** Energy intake is restricted to a time window of 8-10 h or less every day of the week. Variations include Early (eating time window ends before 6 pm) and mid-day TRF (eating time window starts after noon and ends after 6 pm).<br>→ timing of food intake may modify metabolic response to TRF intervention as observational studies have shown that later eating behaviours are linked to obesity and there appears to be reduced weight loss effectiveness in dieters who consume main

		meal later in the day (Quelle 56 von Rynders); though early TRF regimen (= no dinner) might be more difficult to adopt bc it's less aligned with social schedule<br>TRF does **not necessarily have to involve caloric restriction.** TRF is distinct from IMF bc it involves an element of timing optimally aligned to the biological day.<br>→ according to Rynders, TRF is an extra category of IEF not a subcategory of IMF.<br>**Phillips, 2019:** consists of daily fasting periods lasting 12–20 hours, alternating with a daily four-to-twelve hour "eating window" (citing Patterson 2017, Mattson & Longo 2017, Mattson 2018, etc.)<br>**Yang et al., 2021:** Complete fast (no calories) for at least 12 h a day, and eating freely the rest of the time; the 16:8 fasting pattern currently prevails -for their definition Yang cited Cioffi 2018: Cioffi cited studies that apparently defined TRF as 12-21 h fast (Rothshild 2014, Patterson 2015, Hutchlson 2016)
<u>Subcategory of IF:</u><br>***Intermittent dry Fasting (IDF)***	Intermittent dry fasting refers to fasting regimens that involve diurnal or nocturnal restrictions of food and beverage intake. The fasting intervals in these regimens can be shorter or even longer in daily duration than usually defined for intermittent fasting, so they can range from 9 h to 20 h daily. This is due to their usually being part of religious practices and depending on religious rules including duration of daylight.	
Religious Fasting	Religious fasting refers to any fasting regimen that is undertaken as part of a religious practice. Religious fasting thus involves practices such as dry fasting at certain intervals for 24 hours (incl. Jewish traditions, Church of latter-day saints), intermittent dry fasting (incl. Ramadan, Bahá'í Fasting), time-restricted eating (incl. Buddhism) and, if broader defined, diets restricting certain foods (incl. Christian orthodox traditions, Daniel fast).	**Azizi, 2010**: Ramadan is the holiest month in the Islamic calendar, during which Muslims all over the world abstain from eating, drinking, conjugal relationships and smoking from sunrise till sunset as a sign of restraint and introspection in the ninth month of the lunar Islamic year for a period of 29–30 days. Depending on the season and the geographical location of the country, day fasting (= intermittent fasting) varies from 11 to 18 h; being longer in the summer and in temperate regions<br>**Mattson & Longo, 2014:** Many religious groups incorporate periods of fasting into their rituals including Muslims, who fast from dawn until dusk during the month of Ramadan, and Christians, Jews, Buddhists, and Hindus, who traditionally fast on designated days of the week or calendar year<br>**Patterson et al., 2015 - Ramadan:** fast from dawn to sunset during the holy month of Ramadan. In addition to food, fluid intake, cigarette smoking, and medications are forbidden. Depending on the season and the geographical location of the country, day fasting can vary from 11-22 hours. Islamic Fasting during Ramadan does **not** require energy restricition. The most common dietary practice is to consume one large meal

		after sunset and one lighter meal before dawn. Therefore the feast and fast periods of Ramadam are appr. 12 hours in length.<br>→ *caution: this feeding pattern is in biologic opposition to human circadian rhytms, therefore unlikely to be pursued as desirable weight loss intervention*<br>**Latter day Saints:** routinely abstain from food and drink for extended periods of time. Consume last of two daily meals in the afternoon, which results in a long nighttime fasting period that may be biologically important. This meal pattern is typically chronic and sometimes lifelong.
Therapeutic/ Medical fasting	By therapeutic or medical fasting is any fasting or fasting-mimicking regimen that is used by a trained physician as a therapeutic intervention.	**Michalsen et al., 2002:** By definition, medical fasting differs from total food derivation, uncontrolled underfeeding or starvation because of its deliberate character and the accompanying procedures, i.e. relaxation, exercise, lifestyle advice and initial ingestion of laxative salts. In its modern approach ("modified fasting"), fasting includes an intake of 2.5-3l of fluids and 200-400 kcal/day with juice, soup, tea and water (via Farhner, 1991).<br>**Michalsen et al., 2002:** Das therapeutische Fasten ist eine unter stationären Bedingungen durchgeführte Behandlungsmethode, die in zahlreichen spezialisierten Fasten- und naturheilkundlichen Klinikgen standardisiert erfolgt.<br>**Fond et al., 2013:** → Buchinger method: 2 days of 800 kcal/day diet in the form of fruit or rice or potatoes. Patient then receives an oral laxative. Patient then fasts for 1-3 weeks (= 300-500 kcal/day) and ends fast with reintroduction of foods (gradually).<br>**Furmli et al., 2018:** Therapeutic fasting is defined as the controlled and voluntary abstinence from all calorie-containing food and drinks from a specified period of time.
Fasting Mimicking diets (FMD)	A fasting-mimicking diet is a caloric-restricted plant-based diet containing low proteins, low sugar, and high fats. Fasting-mimicking diets usually involve the consumption of solid foods or at least foods containing fibers and can range from 1200 kcal-200 kcal.<br>*5 days per month with 720 – 1100 kcal, vegetarian diet (Fasshauer VL)*	**Pro Lon meal kit from Longo:** 5 day-diet. 1st day: 1100 kcal with plant-based protein, healthy fats and low carbohydrate food sources (includes vegetable soups, bars, crackers, olives and herbal teas). The 5-day-diet is intended to be done once a month for a minimum of 3 months. Days 2-5 contain similar foods as day 1 but in smaller quantities (725 kcal).<br>**Brandhorst et al., 2015:** The human fasting mimicking diet (FMD) program is a plant-based diet program designed to attain fasting-like effects while providing micronutrient nourishment (vitamins, minerals etc.) to minimize the burden of fasting. The human FMD diet consists of a 5 day regimen: day 1 diet of the diet supplies ~1090 kcal (10% protein, 56% fat, 34% carbohydrate), day 2–5 are identical in formulation and provide 725 kcal (9% protein, 44% fat, 47% carbohydrate).<br>**Wei et al.,2017:** 5 day diet. Day 1: 1100 kcal (11% protein, 46% fat, 43 % carbs), days 2-5 provide 717 kcal % protein, 44% fat, 47% arbs) → fat and complex carbs are major source of calories in FMD

		**Difrancesco et al., 2018:** Reduced caloric intake (appr. 30% of energy needs) for 5 consecutive days (50% fat, 40 % carbs, 10 % protein) before returning to normal eating cycles of FMD (30% fat, 55% carbs, 15% protein) once a month or every 3-4 months per year<br>**Vernieri et al. (2019**): an every 3 weeks, 5-days, plant-based, calorie-restricted (600 kcal on day 1; 300 kcal on days 2,3,4; 450 kcal on day 6), sucrose-free, low-carbohydrate, low-protein dietary scheme that is repeated every 3 weeks<br>**De Groot et al. (2020):** FMD is a 4-day plant-based low amino-acid substitution diet, consisting of soups, broths, liquids and tea. Calorie content declined from day 1 (~1200 kcal), to days 2–4 (~200 kcal). Moreover, the carbohydrates/proteins/fats energy ratio was approximately 3.5/1/2 on the first day, while complex carbohydrates were the main macronutrient (>80 energy%) the other subsequent 3 days. Patients were allowed to eat the diet components at any time of the designated day.<br>**Fanti, Longo & Brandhorst, 2021:** FMD is low in protein and sugar, but relatively high in fat content. FMD was developed to be used in periodic cycles from every 2 weeks to every several months and to last from 4-7 days. The 5-day human FMD provides appr. 55% of the recommended daily calorie intake on day 1 and 35% on the subsequent days 2-5.
Buchinger<br><br><br><br>***FX Mayr***		**Wilhelmi De Toledo et al. (2013): Therapeutic Fasting according to Buchinger:** a medically supervised, inpatient multidisciplinary fasting regimen that can be used for prevention or as therapy. Buchinger fasting is based on daily intake of vegetable broth (1/4l), fruit or vegetable juices (1/4l), and honey (30 g) as well as 2–2.5 l of fluid intake by herbal teas and water<br>**FX Mayr**: a regimen of fasting and calorie restriction in 3 steps: The first step consists of tea and water-only fasting, the second step of a hypocaloric milk-bread diet and the third one of a ‘mild intestinal diet, poor in fibers’.

Figure S1 Comprehensive result table for all definitions across the consensus process. Related to Results and Table 2.

Terms concerning dietary and caloric restriction

DIETARY RESTRICTION		Answers (N)	1 (%)	2 (%)	3 (%)	4 (%)	5 (%)	6 (%)
1. round	**Dietary restriction (DR) comprises of continuous or intermittent** restrictions in caloric intake and/or specific macronutrients and/or restraints of food or food and fluid intake within a specified time frame, without malnutrition. DR thus includes: all types of caloric restriction; fasting regimens such as short-term, long-term, and periodic fasting, intermittent fasting, time-restricted eating or feeding, water-only fasting, therapeutic fasting and fasting-mimicking diets; ketogenic diets; and diets with restrictions of specific macronutrients namely proteins, carbohydrates, or fats.	34	**55.88**	**23.53**	11.76	5.88	0.00	2.94

		Answers (N)	A1 (%)	A2 (%)	Neither (%)
2.	**A1: see round 1**<br>**A2: Dietary restriction (DR)** comprises of continuous ~~chronic~~ or intermittent restrictions in caloric intake and / or specific macronutrients and / or restraints of food or food and fluid intake within a specified time frame~~,~~ ~~without malnutrition~~. DR thus includes: all types of caloric restriction; fasting regimens such as short-term, long-term, and periodic fasting, intermittent fasting, time-restricted eating, water- and fluid-only fasting, therapeutic fasting and fasting-mimicking diets; ~~ketogenic diets;~~ and diets with restrictions of specific macronutrients namely proteins, carbohydrates, or fats.	32/33	15.63	78.13	6.25

CONTINUOUS / DAILY ENERGY RESTRICTION*		Answers (N)	1 (%)	2 (%)	3 (%)	4 (%)	5 (%)	6 (%)
2. round	**Continuous Energy Restriction (CER)**, also called **Daily Energy Restriction (DER)**, refers to a daily caloric restriction of about 25-30% of the daily amount of calories required for weight maintenance.	31/33	41.94	22.58	12.90	16.13	3.23	3.23
Note	In the second round, no consensus was reached for the definition of CER / DER. Some experts noted that CER / DER should be defined in the context of caloric restriction instead of alone. For this reason, the term CER/DER was included in the definition of caloric restriction.							

CALORIC RESTRITION		Answers (N)	A1 (%)	A2 (%)	Neither (%)
1. round	**A1**: Caloric restriction (CR) describes a reduction in energy intake below ad libitum levels without malnutrition. (An individual's total energy intake during caloric restriction by this definition could still exceed his / her daily caloric requirements, as is the case with people with overweight and obesity.)<br>**A2:** Caloric restriction (CR) describes a reduction in energy intake below the total caloric intake that would be needed to maintain a healthy body weight, without causing malnutrition. (Total caloric intake here refers to the amount of calories required to maintain a person's "healthy" BMI – depending on their height and age.)	34	41.18	58.82	0.00

		Answers (N)	A1 (%)	A2 (%)	A3 (%)	A4 (%)	A5 (%)	A6 (%)
1.	What range of reduction in daily caloric intake should be included in defining **caloric restriction**?<br>**A1:** 10-25% **A2:** 15-40% **A3:** 20-40% **A4:** 30-40% **A5:** 20-50% **A6:** Other	34	8.82	29.41	8.82	0.00	11.76	41.18

		Answers (N)	1 (%)	2 (%)	3 (%)	4 (%)	5 (%)	6 (%)
2.	**Caloric restriction (CR)** describes a reduction in energy intake below the total ~~caloric intake~~ amount of calories that would be needed to maintain a ~~healthy~~ person's current body weight, without causing malnutrition. ~~(Total caloric intake here refers to the amount of calories required to maintain a person's "healthy" BMI – depending on their height and age.)~~ CR may also be used to achieve a healthy body weight over time.	33/33	**57.58**	**30.30**	0.00	9.09	3.03	0.00

		Answers (N)	A1 (%)	A2 (%)	A3 (%)	A4 (%)	A5 (%)	A6 (%)	A7 (%)	A8 (%)
2.	What range of reduction in daily caloric intake should be included in defining **caloric restriction**?<br>**A1:** 10-25% **A2:** 10-40% **A3:** 10-50% **A4:** 15-40% **A5:** 20-40% **A6:** 20-50% **A7**: Other<br>**A8:** The range can't be generalized (The degree of caloric restriction should be decided individually depending on the person's age, sex, current body weight, occupation, goal & planned duration of CR)	33/33	15.15	27.27	12.12	03.03	03.03	03.03	06.06	30.30

		Answers (N)	A1 (%)	A2 (%)	A3 (%)	6 (%)
3.	**A1: Caloric restriction (CR)** describes a reduction* in energy intake below the total amount of calories that would be needed to maintain a person's current body weight, without causing malnutrition. ~~CR may also be used to achieve a healthy body weight over time.~~ If **CR** is done daily, it can also be referred to as **continuous energy restriction (CER)** or **daily energy restriction (DER).**<br>*Note: amount of reduction is not specified in this definition.<br>**A2: Caloric restriction (CR)** describes a ≥ 10% reduction in energy intake below the total amount of calories that would be needed to maintain a person's current body weight, without causing malnutrition. ~~CR may also be used to achieve a healthy body weight over time.~~ If **CR** is done daily, it can also be referred to as **continuous energy restriction (CER)** or **daily energy restriction (DER).**<br>**A3: Caloric restriction (CR)** describes a reduction in energy intake below the total amount of calories that would be needed to maintain a person's current body weight, without causing malnutrition. The degree of caloric restriction should be decided individually depending on the person's age, sex, current body weight, occupation, goal & planned duration of CR. If **CR** is done daily, it can also be referred to as **continuous energy restriction (CER)** or **daily energy restriction (DER).**	30/30	33.33	13.33	53.33	0.00

		Answers (N)	A1 (%)	A2 (%)	A3 (%)	6 (%)
4.	**A1: Caloric restriction (CR)** describes a reduction in energy intake below the total amount of calories that would be needed to maintain a person's current body weight, without causing malnutrition. If **CR** is done daily, it can also be referred to as **continuous energy restriction (CER)** or **daily energy restriction (DER).**<br>**A2: Caloric restriction (CR)** describes a reduction in energy intake below the total amount of calories that would be needed to maintain a person's current body weight, without causing malnutrition. If **CR** is done daily, it can also be referred to as **continuous energy restriction (CER)** or **daily energy restriction (DER).** *Comment:* The degree of caloric restriction should be decided individually depending on the person's age, sex, body composition, activity level, occupation, goal & planned duration of CR.	33/33	27.27	**72.73**	0.00	0.00

**STARVATION**	Answers (N)	1 (%)	2 (%)	3 (%)	4 (%)	5 (%)	6 (%)

1. round	**Starvation** describes an insufficient nutrient supply based on the abstinence from foods and / or beverages, which is neither voluntary nor controlled. Prolonged starvation can lead to organ failure and / or death.<br>When used in the context of animal studies, the term fasting usually refers to starvation. We propose starvation as the more exact wording in the animal context.	34	47.06	20.59	8.82	14.71	5.88	2.94
2.	**Starvation** describes a catabolic process that occurs when the body's reserves are exhausted after a prolonged period of ~~an~~ insufficient energy and nutrient supply ~~based on the abstinence from foods and / or beverages~~.~~,~~ ~~which is neither voluntary nor controlled. Prolonged~~ Starvation can lead to serious health impairments, organ failure and ~~/ or~~ death.<br>~~When used in the context of animal studies, the term fasting usually refers to starvation. We propose starvation as the more exact wording in the animal context.~~	33/33	66.67	27.27	0.00	3.03	3.03	0.00
**General terms concerning fasting**								
FASTING		Answers (N)	1 (%)	2 (%)	3 (%)	4 (%)	5 (%)	6 (%)
1.	**Fasting** refers to a voluntary abstinence from some or all foods and / or beverages for therapeutic, spiritual, or political reasons.	34	**55.88**	**32.35**	5.88	2.94	2.94	0.00
		Answers (N)	A1 (%)		A2 (%)		Neither (%)	
2.	**A1: see round 1**<br>**A2: Fasting** refers to a voluntary abstinence from some or all foods ~~and /~~ or foods and beverages for preventive, therapeutic, ~~spiritual~~ religious, cultural, or ~~political~~ other reasons.	33/33	6.06		84.85		9.09	
3.	In evaluating the results and comments from the second round, it became clear that it would be very difficult to reach a consensus on the nomenclature of the terms "fasting" and "modified fasting" in a written format. Therefore, it was decided to exclude the term "fasting" in the third round and to discuss it in the LOD instead.							
		Answers (N)	A1 (%)		A2 (%)		Abstained (%)	
LOD	**A1: Fasting** refers to a voluntary abstinence from some or all foods ~~and /~~ or foods and beverages for preventive, therapeutic, ~~spiritual~~ religious, cultural, or ~~political~~ other reasons.<br>**A2:** a more specific definition (e.g. with max. kcal amount & minimum fasting duration)	14/16	68.78		18.75		12.50	
WATER-ONLY FASTING		Answers (N)	1 (%)	2 (%)	3 (%)	4 (%)	5 (%)	6 (%)
1. round	**Water-only fasting** refers to a fasting regimen, where only water (and sometimes tea) is consumed for a certain period of time, usually ≥ 2 days.	34	**50.00**	**29.41**	8.82	11.76	0.00	0.00
		Answers (N)	A1 (%)		A2 (%)		Neither (%)	
2.	**A1: see round 1**<br>**A2: Water-only fasting** refers to a fasting regimen, where only water ~~(and sometimes tea)~~ is consumed for a certain period of time~~, usually ≥ 2 days~~.	32/33	25.00		71.88		3.13	

TOTAL FAST*/**		Answers (N)	1 (%)	2 (%)	3 (%)	4 (%)	5 (%)	6 (%)
**2. round**	The term **total fast**, or **complete fast**, refers to a fasting regimen, where only calorie-free beverages, including water and unsweetened tea, are consumed *ad libitum* for a certain period of time. Historically, prolonged total fasts were used for the therapy of people with obesity under the term **zero-calorie diet***.<br>**see footnote at end of table*	33/33	**69.70**	**15.15**	6.06	6.06	3.03	0.00
**3.**	Despite the consensus reached in round 2, based on some comments, it was decided to delete the above definition and list the terms „total/complete fasting" under "dry fasting" in the third round. This decision was criticized by more than 2 participants and thus the question was included in the LOD.							
**LOD**	Participants in the LOD voted to include "total/complete fasting" as a separate definition in the consensus process for use in studies in which no calories are consumed during the fasting period. Inclusion seemed necessary because it was decided to define "fasting" broadly. Dry fasting experts participating in the LOD supported a distinction between total/complete fasting and dry fasting.							
**4.**	The term **total fast**, or **complete fast**, refers to a fasting regimen, where no calories are consumed for a certain period of time.<br>*Comment*: Total / complete fasting can be equated with water-only fasting.	33/33	39.29	54.55	0.00	6.06	0.00	0.00
		Answers (N)	A1 (%)	A2 (%)	Neither (%)	6 (%)		
**5.**	**A1: see round 4**<br>**A2:** The term **total fast**, or **complete fast**, refers to a fasting regimen, where no calories are consumed for ~~a certain period of time~~ a minimum of 24 hours.<br>*Comment:* Total / complete fasting can be equated with water-only fasting.	29/29	44.83	55.17	0.00	0.00		
**DRY FASTING**		**Answers (N)**	**1 (%)**	**2 (%)**	**3 (%)**	**4 (%)**	**5 (%)**	**6 (%)**
**1. round**	**Dry Fasting** refers to a fasting regimen, during which an abstinence from all foods and beverages is practiced for a limited period of time.	34	61.76	2.94	14.71	2.94	2.94	14.71
**2.**	**Dry Fasting** refers to a fasting regimen, during which a voluntary abstinence from all foods and beverages, including water, is practiced for a certain period of time.	32/33	75.00	12.50	3.13	3.13	0.00	6.25
**3.**	**Dry Fasting**, also called **total fasting / complete fasting**, refers to a fasting regimen, during which a voluntary abstinence from all foods and beverages, including water, is practiced for a certain period of time	27/30	**48.15**	**25.93**	0.00	14.81	11.11	0.00
**LOD**	The decision to equate total/complete fasting with dry fasting was debated in the LOD. Participants decided that dry fasting should not be synonymous with total/complete fasting. Therefore, the terms total/complete fasting are used for a different definition and are not included as synonyms in the final definition of dry fasting. The final definition of dry fasting will be that of the second survey (see above).							
**MODIFIED FASTING***		**Answers (N)**	**1 (%)**	**2 (%)**	**3 (%)**	**4 (%)**	**5 (%)**	**6 (%)**
**2.**	The term **modified fasting** refers to limiting energy intake to 20-25% of energy needs on fasting days. Modified fasting regimens are often adapted to specific clinical settings and indications, allowing for different complementary or supportive therapeutic interventions.	33/33	**63.64**	**12.12**	6.06	12.12	3.03	3.03

<table>
<tr><td>3.</td><td colspan="8">In evaluating the results and comments from the second round, it became clear that it would be very difficult to reach a consensus on the nomenclature of the terms "fasting" and “modified fasting” in a written format. Therefore, it was decided to exclude the term "modified fasting" in the third round and to discuss it in the LOD instead.</td></tr>
<tr><td>LOD</td><td colspan="8">It was decided to define modified fasting broadly (similar to the term “fasting”) to include all types of modified fasting regimens (such as FMD, diets with two separate or consecutive fasting days per week, ADMF).</td></tr>
<tr><td></td><td></td><td>Answers (N)</td><td colspan="2">A1 (%)</td><td colspan="2">A2 (%)</td><td>Neither (%)</td><td>6 (%)</td></tr>
<tr><td rowspan="3">4.</td><td>Please choose which threshold value for the allowed percentage of energy requirements (= kcal intake) on modified fasting days you prefer:
A1: Maximum energy intake of 25% of energy requirements
A2: Maximum energy intake of 35% of energy requirements</td><td>31/33</td><td colspan="2">61.29</td><td colspan="2">19.35</td><td>9.68</td><td>9.68</td></tr>
<tr><td></td><td>Answers (N)</td><td>1 (%)</td><td>2 (%)</td><td>3 (%)</td><td>4 (%)</td><td>5 (%)</td><td>6 (%)</td></tr>
<tr><td>The term modified fasting refers to limiting energy intake, typically up to (…)* % of energy needs on fasting days. Modified fasting regimens encompass alternate-day modified fasting, fasting practiced on 2 separate or consecutive days per week and the fasting-mimicking diet. These regimens are often adapted to specific clinical settings and indications, allowing for different complementary or supportive therapeutic interventions.
*Note that “(…)” will be replaced by the threshold value selected above.</td><td>32/33</td><td>18.75</td><td>62.50</td><td>3.13</td><td>9.38</td><td>3.13</td><td>3.13</td></tr>
<tr><td>5.</td><td>The term modified fasting refers to limiting energy intake to typically up to 25% of energy needs on modified fasting days.
Comment: Modified fasting regimens can be adapted to specific clinical settings and indications, allowing for different complementary or supportive therapeutic interventions. Examples of modified fasting regimens are alternate-day modified fasting, fasting practiced on 2 separate or consecutive days per week and fluid-only fasting ~~and the fasting-mimicking diet~~.*</td><td>29/29</td><td>55.17</td><td>31.03</td><td>0.00</td><td>10.34</td><td>0.00</td><td>3.45</td></tr>
<tr><td colspan="2">FLUID-ONLY FASTING</td><td>Answers (N)</td><td>1 (%)</td><td>2 (%)</td><td>3 (%)</td><td>4 (%)</td><td>5 (%)</td><td>6 (%)</td></tr>
<tr><td>1. round</td><td>Fluid-only fasting refers to a fasting regimen, whereby only non-caloric to low-caloric beverages are consumed for a limited period of time. Water and herbal tea may be consumed ad libitum. In addition, clear vegetable broth as well as vegetable- and / or fruit juice may be consumed up to a maximum of 500 kcal per day in total.</td><td>34</td><td>38.24</td><td>26.47</td><td>14.71</td><td>8.82</td><td>2.94</td><td>8.82</td></tr>
<tr><td></td><td></td><td>Answers (N)</td><td colspan="2">A1 (%)</td><td colspan="2">A2 (%)</td><td colspan="2">Neither (%)</td></tr>
<tr><td>2.</td><td>A1: Fluid-only fasting refers to a fasting regimen, whereby only ~~non-caloric to low-caloric~~ beverages are consumed for a ~~limited~~ certain period of time. Water and unsweetened herbal tea may be consumed ad libitum. In addition, clear vegetable broth, vegetable- and / or fruit juice, as well as up to 2 cups of plain coffee and 2 teaspoons of honey may be consumed ~~up to a maximum of 500 kcal per day in total~~ per day, not exceeding a total of 800 kcal (consistent with the definition of a very low-calorie diet).
A2: Fluid-only fasting refers to a fasting regimen, whereby only ~~non-caloric to low-caloric~~ beverages are consumed for a ~~limited~~ certain period of time. Water and unsweetened herbal tea may be consumed ad libitum. In addition, clear vegetable broth, vegetable- and / or fruit juice, as well as up to 2 cups of plain coffee and 2</td><td>31/33</td><td colspan="2">29.03</td><td colspan="2">38.71</td><td colspan="2">32.26</td></tr>
</table>

	teaspoons of honey may be consumed ~~up to a maximum of 500 kcal per day in total~~ per day, not exceeding a total of 250 kcal.							
3.	As this term could potentially be classified under "modified fasting," it was excluded in the third round and included in the LOD instead.							
LOD	It was decided that the definition of fluid-only fasting should allow only "non-ultra-processed liquids," e.g., water, tea, and juices with no added sugar.							
		**Answers (N)**	**1 (%)**	**2 (%)**	**3 (%)**	**4 (%)**	**5 (%)**	**6 (%)**
4.	**Fluid-only fasting** refers to a fasting regimen, whereby only beverages are consumed for a certain period of time. Water and unsweetened herbal tea may be consumed *ad libitum*. Clear vegetable broth, vegetable and/or fruit juices can be consumed up to a total of 500 kcal per day*. Ultra-processed fluids should not be consumed.<br>*based on the Expert Panel Update of the 2002 Consensus Guidelines for Fasting Therapy from 2013.<br>*Comment*: This fasting regimen includes traditional fasting regimens that use various broths, gruel, or decoctions, such as the traditional German gruel fasting.	32/33	**31.25**	**46.88**	6.25	9.38	0.00	6.25
		**Answers (N)**	**A1 (%)**	**A2 (%)**	**Neither (%)**	**6 (%)**		
5.	**A1:** see round 4<br>**A2: Fluid-only fasting** refers to a modified fasting regimen, whereby only beverages are consumed for a certain period of time. Water and unsweetened herbal tea may be consumed *ad libitum*. Clear vegetable broth, vegetable and/or fruit juices can be consumed up to a total of 500 kcal per day*. Ultra-processed fluids should not be consumed.<br>*based on the Expert Panel Update of the 2002 Consensus Guidelines for Fasting Therapy from 2013. *Comment:* This fasting regimen includes traditional fasting regimens that use various broths, gruel, or decoctions, such as the traditional German gruel fasting.	28/29	25.00	71.43	3.57	0.00		
	**Continuous Fasting Regimens**							
	**SHORT-TERM FASTING**	**Answers (N)**	**1 (%)**	**2 (%)**	**3 (%)**	**4 (%)**	**5 (%)**	**6 (%)**
1. round	**Short-term fasting (STF** also called **short-term caloric restriction,** or, when referring to animals, **short-term starvation (STS)**, is any fasting regimen with a duration of 48 to 72 hours.	34	38.24	11.76	8.82	29.41	5.88	5.88
		**Answers (N)**	**A1 (%)**	**A2 (%)**	**A3 (%)**	**A4 (%)**	**A5 (%)**	
2.	**Short-term fasting (STF)** refers to a fluid-only fasting regimen with a duration of:<br>**A1:** 16 to 72 hours. **A2:** 24 to 48 hours. **A3:** 48 to 72 hours. **A4:** 1- 4 days. **A5:** 1 to 5 days.	33/33	30.30	21.21	27.27	15.15	6.06	
3.	In evaluating the results and comments from the second round, it became clear that it would be difficult to reach a consensus on the duration of “short-term fasting” in a written format. Therefore, the term was excluded in the third round and included in the LOD instead.							
LOD	Due to insufficient time, this term could not be addressed in the LOD.							
		**Answers (N)**	**1 (%)**	**2 (%)**	**3 (%)**	**4 (%)**	**5 (%)**	**6 (%)**

**4.**	**Short-term fasting (STF)** refers to a fluid-only fasting regimen with a duration of 1-3 days.	32/33	31.25	50.00	6.25	12.50	0.00	0.00
		**Answers (N)**	**A1 (%)**	**A2 (%)**	**A3 (%)**	**A4 (%)**	**None (%)**	**6 (%)**
**5.**	**A1: see round 4**<br>**A2: Short-term fasting (STF)** refers to total and modified fasting regimens with a duration of **2-3 days.**<br>**A3: Short-term fasting (STF)** refers to total and modified fasting regimens with a duration of **1-2 days.**<br>**A4: Short-term fasting (STF)** refers to total and modified fasting regimens with a duration of 1-3 days.	29/29	17.24	48.28	6.90	27.59	0.00	0.00
**PROLONGED / LONG-TERM FASTING**		**Answers (N)**	**1 (%)**	**2 (%)**	**3 (%)**	**4 (%)**	**5 (%)**	**6 (%)**
**1. round**	**Prolonged fasting (PF)**, also called **long-term fasting (LTF)**, refers to any fasting regimen lasting ≥ 5 days.	34	52.94	14.71	14.71	14.71	0.00	2.94
		**Answers (N)**	**A1 (%)**	**A2 (%)**	**A3 (%)**	**A4 (%)**		
**2.**	**Prolonged fasting (PF)**, also called **long-term fasting (LTF)**, refers to any fluid-only fasting regimen lasting<br>**A1:** ≥ 3 consecutive days. **A2:** ≥ 4 consecutive days. **A3:** ≥ 5 consecutive days. **A4:** ≥ 7 consecutive days.	32/33	40.63	28.13	28.13	3.13		
**3.**	In evaluating the results and comments from the second round, it became clear that it would be difficult to reach a consensus on the duration of "long-term fasting" in a written format. Therefore, the term was excluded in the third round and included in the LOD instead.							
**LOD**	Due to insufficient time, this term could not be addressed in the LOD.							
		**Answers (N)**	**1 (%)**	**2 (%)**	**3 (%)**	**4 (%)**	**5 (%)**	**6 (%)**
**4.**	**Prolonged fasting (PF)**, also called **long-term fasting (LTF)**, refers to any fluid-only fasting regimen lasting ≥ 4 consecutive days.	32/33	**31.25**	**50.00**	3.13	15.63	0.00	0.00
		**Answers (N)**	**A1 (%)**	**A2 (%)**	**A3 (%)**	**None (%)**	**6 (%)**	
**5.**	**A1**: see round 4<br>**A2: Prolonged fasting (PF),** also called **long-term fasting (LTF)**, refers to ~~fluid-only~~ total and modified fasting regimens with a duration of ≥ 4 consecutive days.<br>**A3: Prolonged fasting (PF),** also called **long-term fasting (LTF)**, refers to ~~fluid-only~~ total and modified fasting regimens with a duration of ≥ 3 consecutive days.	29/29	27.59	51.72	20.69	0.00	0.00	
**PERIODIC FASTING**		**Answers (N)**	**1 (%)**	**2 (%)**	**3 (%)**	**4 (%)**	**5 (%)**	**6 (%)**
**1. round**	**Periodic fasting (PF)** refers to any fasting regimen that is repeated at regular intervals (periods), such as every day, every week, or every several months.	34	**52.94**	**17.65**	2.94	17.65	8.82	0.00
		**Answers (N)**	**A1 (%)**	**A2 (%)**	**Neither (%)**			
**2.**	**A1: s.** Runde 1 (According to this definition, periodic fasting would include intermittent fasting regimens.)	33/33	51.52	42.42	6.06			

	**A2: Periodic fasting (PF)** refers to any fasting regimen lasting ≥ 48 hours that is repeated at regular intervals (periods), such as ~~every day,~~ every several weeks or ~~every several~~ months.							
3.	In evaluating the results and comments from the second round, it became clear that it would be difficult to reach a consensus on this term in a written format. Therefore, the term was excluded in the third round and included in the LOD instead.							
LOD	Due to insufficient time, this term could not be addressed in the LOD.							
		**Answers (N)**	**1 (%)**	**2 (%)**	**3 (%)**	**4 (%)**	**5 (%)**	**6 (%)**
4.	**Periodic fasting (PF)** refers to any fasting regimen that is repeated at regular intervals (periods), such as every day, every week, or every several months.<br>*Comment*: According to this definition, periodic fasting would include intermittent fasting regimens.	32/33	34.38	46.88	3.13	15.63	0.00	0.00
		**Answers (N)**	**A1 (%)**	**A2 (%)**	**Neither (%)**	**6 (%)**		
5.	**A1:** see round 4<br>**A2: Periodic fasting (PF)** refers to ~~any~~ prolonged fasting regimens that are repeated at ~~is repeated at regular~~ intervals (periods)~~, such as every day, every week, or every several months~~.	28/29	53.57	46.43	0.00	0.00		

Intermittent Fasting Regimens

**INTERMITTENT FASTING**		**Answers (N)**	**1 (%)**	**2 (%)**	**3 (%)**	**4 (%)**	**5 (%)**	**6 (%)**
1. round	**Intermittent fasting (IF)** refers to repetitive fasting periods of up to 48 h. IF includes fasting regimens of 1 day per week (6:1), 2 separate or consecutive days per week (5:2), alternate day fasting (ADF) and time-restricted eating (TRE).	34	**70.59**	**17.65**	0.00	8.82	2.94	0.00
		**Answers (N)**	**A1 (%)**		**A2 (%)**		**Neither (%)**	
2.	**A1:** see round 1<br>**A2: Intermittent fasting (IF)** refers to repetitive fasting periods lasting up to 48 h each. IF includes fasting regimens of 1 fasting day per week ~~(6:1),~~ 2 separate or consecutive fasting days per week ~~(5:2)~~ and alternate day fasting (ADF) ~~and time-restricted eating (TRE)~~.	32/33	40.63		50.00		9.38	
		**Answers (N)**	**1 (%)**	**2 (%)**	**3 (%)**	**4 (%)**	**5 (%)**	**6 (%)**
3.	**Intermittent fasting (IF)** refers to repetitive fasting periods lasting up to 48 h each. IF includes fasting regimens of 1 fasting day per week, 2 separate or consecutive fasting days per week, alternate-day fasting (ADF), and time-restricted eating (TRE).	30/30	50.00	36.67	0.00	10.00	3.33	0.00
**TIME-RESTRICTED EATING**		**Answers (N)**	**1 (%)**	**2 (%)**	**3 (%)**	**4 (%)**	**5 (%)**	**6 (%)**
1. round	**Time-restricted eating (TRE)** or, when referring to animals, **time-restricted feeding (TRF)**, is a dietary regimen in which food intake is restricted to a specific period of time (usually 4 to 10 hours) during the day, resulting in a daily fasting window of 14 to 20 hours. There is no explicit limit on energy intake during eating or feeding hours.	34	**70.59**	**20.59**	5.88	2.94	0.00	0.00

<table>
<tr><th></th><th></th><th>Answers (N)</th><th colspan="3">A1 (%)</th><th colspan="3">A2 (%)</th><th colspan="3">A3 (%)</th><th colspan="3">None of them (%)</th></tr>
<tr><td>2.</td><td>A1: see round 1
A2: Time-restricted eating (TRE) ~~or, when referring to animals, **time-restricted feeding (TRF)**,~~ is a dietary regimen in which food intake and the consumption of caloric beverages is restricted to a specific period of time (usually 4 to 10 hours) during the day, resulting in a daily fasting window of 14 to 20 hours. There is no explicit limit on energy intake during eating ~~or feeding~~ hours.
A3: Time-restricted eating (TRE) ~~or, when referring to animals, **time-restricted feeding (TRF)**,~~ is a dietary regimen in which food intake and the consumption of caloric beverages is restricted to a specific period of time (usually 1 to 12 hours) during the day, resulting in a daily fasting window of 12 to 23 hours. There is no explicit limit on energy intake during eating ~~or feeding~~ hours.</td><td>32/33</td><td colspan="3">18.75</td><td colspan="3">59.38</td><td colspan="3">15.63</td><td colspan="3">6.25</td></tr>
<tr><th></th><th></th><th>Answers (N)</th><th colspan="4">A1 (%)</th><th colspan="4">A2 (%)</th><th colspan="4">Neither (%)</th></tr>
<tr><td>3.</td><td>A1: Time-restricted eating (TRE) is a dietary regimen in which food intake and the consumption of caloric beverages is restricted to a specific period of time (usually 1 to 10 hours) during the day, resulting in a daily fasting window of at least 14 hours. There is no explicit limit on energy intake during eating hours.*
*Note: Low frequency meal patterns such as the "one meal a day diet (OMAD)" can be regarded as part of TRE according to this definition.
A2: Time-restricted eating (TRE) is a dietary regimen in which food intake and the consumption of caloric beverages is restricted to a specific period of time (usually 4 to 10 hours) during the day, resulting in a daily fasting window of 14 to 20 hours. There is no explicit limit on energy intake during eating hours.</td><td>30/30</td><td colspan="4">53.33</td><td colspan="4">43.33</td><td colspan="4">3.33</td></tr>
<tr><td>LOD</td><td colspan="14">Since no consensus could be reached on the duration of the fasting and eating window in TRE, this issue was raised in the LOD. Participants decided that it would be simplest not to define the overall fasting or eating window, but to define only the minimum fasting duration. In this way, the one-meal-a-day diet (OMAD) could also be considered as part of TRE.</td></tr>
<tr><th></th><th></th><th>Answers (N)</th><th colspan="2">1 (%)</th><th colspan="2">2 (%)</th><th colspan="2">3 (%)</th><th colspan="2">4 (%)</th><th colspan="2">5 (%)</th><th colspan="2">6 (%)</th></tr>
<tr><td>4.</td><td>Time-restricted eating (TRE) is a dietary regimen in which food intake and the consumption of caloric beverages is restricted to a specific period of time ~~(usually 1 to 10 hours)~~ during the day, resulting in a daily fasting window of at least 14 hours. There is no explicit limit on energy intake during eating hours.</td><td>32/33</td><td colspan="2">50.00</td><td colspan="2">37.50</td><td colspan="2">3.13</td><td colspan="2">3.13</td><td colspan="2">6.25</td><td colspan="2">0.00</td></tr>
<tr><th></th><th></th><th>Answers (N)</th><th colspan="3">A1 (%)</th><th colspan="3">A2 (%)</th><th colspan="3">Neither (%)</th><th colspan="3">6 (%)</th></tr>
<tr><td>5.</td><td>A1: see round 4
A2: Time-restricted eating (TRE) is a dietary regimen in which food intake and the consumption of caloric beverages is restricted to a specific period of time during the day, resulting in a daily fasting window of at least 12 hours. There is no explicit limit on energy intake during eating hours.</td><td>29/29</td><td colspan="3">62.07</td><td colspan="3">31.03</td><td colspan="3">6.90</td><td colspan="3">0.00</td></tr>
</table>

INTERMITTENT ENERGY RESTRICTION*		Answers (N)	1 (%)	2 (%)	3 (%)	4 (%)	5 (%)	6 (%)
2. round	**Intermittent Energy Restriction (IER)** includes periods of caloric restriction alternating with periods of *ad libitum* eating. As such, IER includes fasting regimens like intermittent fasting (IF) and time-restricted eating (TRE).	32/33	78.13	3.13	0.00	9.38	6.25	3.13
		Answers (N)	A1 (%)		A2 (%)		Neither (%)	
3.	**A1:** see round 1<br>**A2: Intermittent Energy Restriction (IER)** includes periods of caloric restriction alternating with periods of *ad libitum* ~~eating~~ energy intake. ~~As such, IER includes fasting regimens like intermittent fasting (IF) and time-restricted eating (TRE).~~	30/30	56.67		43.33		0.00	
		Answers (N)	1 (%)	2 (%)	3 (%)	4 (%)	5 (%)	6 (%)
4.	**Intermittent Energy Restriction (IER)** includes periods of modified fasting alternating with periods of *ad libitum* energy intake.<br>*Comment*: IER is a subcategory of intermittent fasting (IF) and includes regimens such as alternate-day modified fasting (ADMF), fasting on 2 separate or consecutive days per week, weeks of caloric restriction followed by weeks of eating in energy balance, and the fasting-mimicking diet.	31/33	**29.03**	**45.16**	0.00	16.13	6.45	3.23
		Answers (N)	A1 (%)	A2 (%)	Neither (%)	6 (%)		
5.	**A1: Intermittent Energy Restriction (IER)** or intermittent caloric restriction, includes periods of caloric restriction alternating with periods of *ad libitum* eating.<br>*Comment:* IER ~~is a subcategory of intermittent fasting (IF) and~~ includes all intermittent fasting regimens~~, fasting-mimicking diets~~ and weeks of caloric restriction followed by weeks of *ad libitum* eating.<br>**A2:** see round 4	28/29	46.43	39.29	14.29	0.00		
**ALTERNATE-DAY FASTING**		Answers (N)	1 (%)	2 (%)	3 (%)	4 (%)	5 (%)	6 (%)
1. round	**Alternate day fasting (ADF),** or in animals, **every-other-day feeding (EOD),** refers to alternating a day of eating *ad libitum* and a day of either water-only fasting or a diet very low in calories*.<br>**In this case, alternate-day modified fasting would fall under the definition of ADF.*	34	**52.94**	**29.41**	2.94	2.94	8.82	2.94
		Answers (N)	A1 (%)		A2 (%)		Neither (%)	
2.	**A1:** see round 1<br>**A2: Alternate day fasting (ADF)** ~~or in animals, **every-other-day feeding (EOD),**~~ refers to alternating a day of eating *ad libitum* and a day of ~~either~~ water-only fasting ~~or a diet very low in calories~~.	31/33	41.94		54.84		3.23	
3.	As this term could potentially be classified under "fasting," it was excluded in the third round and included in the LOD instead.							
LOD	It was decided to create two separate definitions for alternate-day fasting and alternate-day modified fasting.							
		Answers (N)	1 (%)	2 (%)	3 (%)	4 (%)	5 (%)	6 (%)
4.	**Alternate day fasting (ADF)** refers to alternating a day of eating *ad libitum* and a day of water-only fasting.	33/33	39.39	54.55	0.00	6.06	0.00	0.00

ALTERNATE-DAY MODIFIED FASTING*		Answers (N)	A1 (%)	A2 (%)	A3 (%)	A4 (%)	A5 (%)
2. round	**Alternate-day modified fasting (ADMF)** refers to alternating a day of eating *ad libitum* and a day of eating a low-calorie diet with up to:<br>**A1:** 400 kcal.<br>**A2:** 500 kcal.<br>**A3:** 600 kcal.<br>**A4:** 800 kcal.<br>**A5:** 20 to 25 % of the usual energy intake.	33/33	24.24	12.12	9.09	9.09	45.45
3.	As this term could potentially be classified under "modified fasting," it was excluded in the third round and included in the LOD instead.						
LOD	It was decided to align the definition of alternate-day modified fasting with the broad definition of modified fasting.						

		Answers (N)	A1 (%)	A2 (%)	Neither (%)	6 (%)
4.	**A1: Alternate-day modified fasting (ADMF)** refers to alternating a day of eating *ad libitum* and a day of modified fasting.<br>**A2: Alternate-day modified fasting (ADMF)** refers to alternating a day of eating ad libitum and a day of modified fasting with limited caloric intake at lunch or dinner.	32/33	62.50	28.13	6.25	3.13

		Answers (N)	1 (%)	2 (%)	3 (%)	4 (%)	5 (%)	6 (%)
5.	**A1:** see A1, round 4	28/29	64.29	32.14	0.00	0.00	0.00	3.57

Special Fasting Regimens

THERAPEUTIC / MEDICAL FASTING		Answers (N)	1 (%)	2 (%)	3 (%)	4 (%)	5 (%)	6 (%)
1. round	**Therapeutic fasting**, also called **medical fasting**, refers to any fasting regimen that is applied as a therapeutic intervention by a trained physician.	34	**70.59**	**11.76**	8.82	2.94	0.00	5.88

		Answers (N)	A1 (%)	A2 (%)	Neither (%)
2.	**A1:** see round 1<br>**A2: Therapeutic fasting** refers to any fasting regimen that is applied as a therapeutic intervention. **Medically supervised fasting** refers to any fasting regimen that is applied as a therapeutic intervention by a trained physician or similar credentialed healthcare provider.	31/33	32.26	67.74	0.00
3.	As this term could potentially be classified under "fasting" as well as "modified fasting," it was excluded in the third round and included in the LOD instead.				
LOD	It was decided to use a broad and brief definition of fasting, but to include the aspect of individualization of the chosen fasting intervention in the definition of therapeutic fasting.				

		Answers (N)	1 (%)	2 (%)	3 (%)	4 (%)	5 (%)	6 (%)
4.	**Therapeutic fasting** refers to any fasting regimen that is applied as a therapeutic intervention.<br>*Comment:* Therapeutic fasting interventions are individually tailored to a person's age, sex, body composition, physical activity level, occupation, goal & planned duration of fasting.<br>**Medically supervised fasting** refers to any fasting regimen that is applied as a therapeutic intervention by a trained physician or similar credentialed healthcare provider.	32/33	34.38	56.25	3.13	0.00	3.13	3,.13
PREVENTIVE FASTING***		Answers (N)	1 (%)	2 (%)	3 (%)	4 (%)	5 (%)	6 (%)
5.	**Preventive fasting** refers to any fasting regimen that is applied as a preventive intervention.	28/29	50.00	32.14	0.00	3.57	0.00	14.29
Note	In the context of the discussion on fasting used as a therapeutic intervention, it was decided to propose an additional definition for the term preventive fasting.							
BUCHINGER THERAPEUTIC FASTING		Answers (N)	1 (%)	2 (%)	3 (%)	4 (%)	5 (%)	6 (%)
1. round	**BUCHINGER therapeutic fasting** is a fluid-only fasting regimen, allowing for a maximum of 500 kcal per day and lasting at least 5 days, practiced for the prevention or treatment of diseases as well as to support one's individual health, taking into account a person's medical, psychosocial and spiritual dimensions. It is usually accompanied by bowel / colon cleansing procedures and preceded and followed by a few days of a calorie restricted, easily digestible diet.	34	52.94	5.88	20.59	2.94	2.94	14.71
2.	**BUCHINGER therapeutic fasting** is a fluid-only fasting regimen, allowing for a maximum of 500 kcal per day and lasting at least 5 days, practiced for the prevention or treatment of diseases as well as to support one's individual health, taking into account a person's medical, psychosocial and spiritual dimensions. It is usually accompanied by bowel / colon cleansing procedures and preceded and followed by a few days of a calorie restricted, easily digestible diet.	23/33	86.96	4.35	0.00	0.00	0.00	8.70
GRUEL FASTING		Answers (N)	1 (%)	2 (%)	3 (%)	4 (%)	5 (%)	6 (%)
2. round	**Gruel fasting** refers to a fasting regimen in which 400 ml of oat or rice gruel is given in three portions per day. Water and unsweetened herbal tea may be consumed *ad libitum*. Gruel fasting provides about 200 kcal and 45 g of carbohydrates per day.	26/33	46.16	3.85	0.00	3.85	3.85	42.31
Note	Gruel fasting is a traditional form of fasting practiced in Germany. Since neither consensus nor exclusion was reached for this definition in round 2, and since gruel fasting is technically a fluid-only fast, this term is included in a comment on the definition of fluid-only fasting.							
FX MAYR-THERAPY		Answers (N)	1 (%)	2 (%)	3 (%)	4 (%)	5 (%)	6 (%)
1. round	**FX-Mayr-Therapy** or **FX-Mayr Cure** refers to a 3-phase fasting regimen containing elements of water-only fasting, a very low-calorie diet with a training of "proper chewing", in order to help individuals (re-)gain their sense of satiety and an easily digestible diet towards the end of the treatment. The dietary intervention is accompanied by manual treatments focusing on the abdominal region.	34	44.12	14.71	20.59	0.00	0.00	20.59

2.	**FX-Mayr-Therapy** or **FX-Mayr Cure** refers to a 3-phase fasting regimen containing elements of water-only fasting, a very low-calorie diet with a training of "proper chewing", in order to help individuals (re-)gain their sense of satiety and an easily digestible diet towards the end of the treatment. The dietary intervention is accompanied by manual treatments focusing on the abdominal region.	22/33	77.27	0.00	0.00	4.55	0.00	18.18
3.	Despite the consensus reached in round 2, one expert on FX-Mayr-Therapy suggested further changes to the definition to ensure that it was up to date. Detailed information on the necessary changes was not obtained by the start of the third survey, so the newly adapted definition was submitted for re-evaluation in the fourth round.							
4.	**FX-Mayr-Therapy** or **FX-Mayr Cure** refers to a ~~3-phase fasting~~ regimen containing elements of water-only fasting, a very low-calorie diet with a training of "proper chewing", in order to help individuals (re-)gain their sense of satiety and an easily digestible diet towards the end of the treatment. The dietary intervention is accompanied by bowel cleansing procedures and manual treatments focusing on the abdominal region.	22/33	31.82	68.18	0.00	0.00	0.00	0.00
**FASTING-MIMICKING DIET**		**Answers (N)**	**1 (%)**	**2 (%)**	**3 (%)**	**4 (%)**	**5 (%)**	**6 (%)**
1. round	A **fasting-mimicking diet (FMD)** specifies any diet that aims to induce metabolic effects of fasting. It usually refers to a plant-based, calorie-restricted diet that lasts 3 to 5 days and is followed periodically (e.g. once a month). **FMDs** are usually free of refined sugars and low in protein but high in unsaturated fatty acids and complex carbohydrates.	34	52.94	14.71	20.59	2.94	0.00	8.82
Note	Some experts questioned whether the fasting-mimicking diet should be included in this consensus process. This question was returned to the panel in round 2. All participants who voted "yes" (= 68.75%) were asked two additional questions to further define the term.							
		**Answers (N)**	**A1 (%)**			**A2 (%)**		
2.	Please indicate whether you consider the inclusion of the term **Fasting-Mimicking Diet** important for this consensus process.<br>**A1:** Yes, FMD should be included. **A2:** No, FMD should not be included.	**32**/33	68.75 = 22 participants			31.25 = 10 participants		
		**Answers (N)**	**A1 (%)**			**A2 (%)**		
	How many calories should be consumed as part of the **Fasting-Mimicking Diet**:<br>**A1:** 600-1000 kcal per day **A2:** a maximum of 600 kcal per day	22/**32**	50.00			50.00		
		**Answers (N)**	**A1 (%)**		**A2 (%)**	**A3 (%)**		**A4 (%)**
	Should a **Fasting-Mimicking Diet** per definition include (more than one answer option may be chosen):<br>**A1:** A low carbohydrate intake **A2:** A low protein intake **A3:** A low intake of fatty acids<br>**A4:** Only a restricted intake of refined sugars and calories	22/**32**	17.95		35.90	12.82		33.33
		**Answers (N)**	**1 (%)**	**2 (%)**	**3 (%)**	**4 (%)**	**5 (%)**	**6 (%)**
3.	A **Fasting-Mimicking Diet (FMD)** specifies any diet that is specifically composed to induce the metabolic effects of fasting while allowing for a potentially higher caloric intake. It usually refers to a plant-based, calorie restricted diet with a maximum of 1000 kcal per day with solid food components that lasts 3 to 5 days and is followed periodically (e.g. once a month). **FMDs** are usually free of refined sugars and starch and low in protein.	30/30	**29.63**	**40.74**	3.70	7.41	7.41	11.11

Round	Definition	Answers (N)	1 (%)	2 (%)	3 (%)	4 (%)	5 (%)	6 (%)
4.	A **Fasting-Mimicking Diet (FMD)** specifies any diet that is specifically composed to induce the metabolic effects of fasting while allowing for a potentially higher caloric intake, including solid foods. It usually refers to a plant-based, calorie restricted diet with a maximum of 1400 kcal per day ~~with solid food components~~ that lasts 3 to 7 days ~~and is followed periodically (e.g. once a month)~~. FMDs are usually relatively low in refined sugars and starch, low in protein and high in plant-based fats.<br>*Comment*: It is recommended to follow the FMD once a month to every four months.	33/33	**36.36**	**42.42**	0.00	6.06	9.09	6.06
		**Answers (N)**	**A1 (%)**	**A2 (%)**	**Neither (%)**	**6 (%)**		
5.	**A1:** see round 4<br>**A2:** A **Fasting-Mimicking Diet (FMD)** specifies any diet that is specifically composed to induce the metabolic effects of fasting while allowing for a potentially higher caloric intake, including solid foods. It usually refers to a plant-based, calorie restricted diet with a maximum of approximately 1000 kcal per day that lasts 3 to 7 days. FMDs are usually relatively low in refined sugars and starch, low in protein and high in plant-based fats.<br>~~Note: It is recommended to follow the FMD once a month to every four months.~~<br>*Comment*: The exact amount of calories, macronutrient composition, duration and frequency of use needs to be decided individually. FMD meals can consist of packaged products or be freshly prepared.	28/29	10.71	75.00	7.14	7.14		
**RELIGIOUS FASTING**		**Answers (N)**	**1 (%)**	**2 (%)**	**3 (%)**	**4 (%)**	**5 (%)**	**6 (%)**
1. round	**Religious fasting** refers to any fasting regimen that is undertaken as part of a religious practice. Religious fasting thus involves practices such as: dry fasting at certain intervals over 24 hours (e.g. Jewish traditions, The Church of Jesus Christ of Latter-day Saints); intermittent dry fasting (e.g. Ramadan fasting, Bahá'í fasting); time-restricted eating (e.g. Buddhism); and diets restricting certain foods (e.g. Christian orthodox traditions, Daniel fast) if more broadly defined.	34	**76.47**	**17.65**	5.88	0.00	0.00	0.00
		**Answers (N)**	**A1 (%)**	**A2 (%)**	**Neither (%)**			
2.	**A1:** see round 1<br>**A2: Religious fasting** refers to any fasting regimen that is undertaken as part of a religious practice. Religious fasting thus involves practices such as: dry fasting ~~at certain intervals over~~ on specific days of the year up to 25 hours at a time (e.g. Jewish tradition, The Church of Jesus Christ of Latter-day Saints); intermittent dry fasting (e.g. Ramadan fasting, Bahá'í fasting); time-restricted eating (e.g. Buddhism); and diets restricting certain foods (e.g. Christian orthodox traditions, Daniel fast) if more broadly defined. Typically, religious fasting also includes spiritual activities to improve cognitive function and well-being.	31/33	38.71	51.61	9.68			
		**Answers (N)**	**1 (%)**	**2 (%)**	**3 (%)**	**4 (%)**	**5 (%)**	**6 (%)**
3.	**Religious fasting** refers to any fasting regimen that is undertaken as part of a religious practice. Religious fasting thus involves practices such as: dry fasting (= total / complete fasting) on specific days of the year up to 25 hours at a time (e.g. Jewish tradition, The Church of Jesus Christ of Latter-day Saints); intermittent dry fasting (e.g. Ramadan fasting, Bahá'í fasting); time-restricted eating (e.g. Buddhism); and diets restricting certain foods (e.g. Christian orthodox traditions, Daniel fast) if more broadly defined. Typically, religious fasting also includes spiritual activities to improve cognitive function and well-being.	29/30	**31.03**	**48.28**	3.45	10.34	6.90	0.00

**4.**	**Religious fasting** refers to any fasting regimen that is undertaken as part of a religious practice.<br>*Comment:* Religious fasting thus involves practices such as: dry fasting on specific days of the year up to 25 hours at a time (e.g. Jewish tradition, The Church of Jesus Christ of Latter-day Saints); intermittent dry fasting (e.g. Ramadan fasting, Bahá'í fasting); time-restricted eating (e.g. Buddhism); and diets restricting certain foods (e.g. Christian orthodox traditions, Daniel fast) if more broadly defined. Typically, religious fasting ~~also~~ includes spiritual activities to improve cognitive function and well-being.	32/33	34.38	59.38	3.13	0.00	0.00	3.13
		**Answers (N)**	**A1 (%)**		**A2 (%)**		**Neither (%)**	**6 (%)**
**5.**	**A1:** see round 4<br>**A2: Religious fasting** refers to any fasting regimen that is undertaken as part of a religious practice.<br>*Comment*: Religious fasting thus involves practices such as: dry fasting on specific days of the year up to 25 hours (e.g. Jewish tradition, The Church of Jesus Christ of Latter-day Saints) or up to 60 hours at a time (e.g. Christian orthodox tradition); intermittent dry fasting (e.g. Ramadan fasting, Bahá'í fasting); time-restricted eating (e.g. Buddhism); and diets restricting certain foods (e.g. Christian orthodox traditions, Daniel fast) if more broadly defined. Typically, religious fasting includes spiritual activities aimed at improving cognitive function and well-being.	27/29	29.63		66.67		3.70	0.00
**INTERMITTENT DRY FASTING**		**Answers (N)**	**1 (%)**	**2 (%)**	**3 (%)**	**4 (%)**	**5 (%)**	**6 (%)**
**1. round**	**Intermittent dry fasting (IDF)** refers to intermittent fasting regimens that also involve restrictions of fluid intake during fasting hours. Daily fasting interval time frames may fall outside those usually defined for intermittent fasting, for instance ranging from 9 to 20 hours daily as defined by religious rules depending on daylight hours. Diurnal intermittent dry fasting, as practiced in religious contexts, does not require any defined energy restriction during the nocturnal eating window.	34	64.71	2.94	5.88	11.76	2.94	11.76
		**Answers (N)**	**A1 (%)**		**A2 (%)**		**Neither (%)**	
**2.**	**A1: Intermittent dry fasting (IDF)** refers to intermittent fasting regimens that ~~also~~ involve abstaining from food and fluid intake during fasting hours. As daily fasting intervals generally depend on daylight hours, they may range from 9 to 20 hours. Diurnal intermittent dry fasting, as practiced in religious contexts, does not require any defined energy restriction during the nocturnal eating window.<br>**A2: Diurnal dry fasting**, as practiced in religious contexts, refers to a dietary regimen that involves abstaining from food and fluid intake during daylight hours for a certain period every year. The daily fasting window may range from 9 to 20 hours. There are no requirements on energy restriction during the nocturnal eating window.	28/33	50.00		42.86		7.14	
		**Answers (N)**	**1 (%)**	**2 (%)**	**3 (%)**	**4 (%)**	**5 (%)**	**6 (%)**
**3.**	**Intermittent dry fasting (IDF)** refers to intermittent fasting regimens that involve abstaining from food and fluid intake during fasting hours. Most commonly, they range from 9 to 20 hours.	27/30	29.63	55.56	0.00	7.41	0.00	7.41

LOD = Live Online Discussion; **1** = strongly agree; **2** = agree; **3** = neutral; **4** = disagree; **5** = strongly disagree; **6** = This detail / definition is irrelevant and should be excluded from the consensus process.

→ To determine the level of agreement, the percentages of 1 and 2 are added together; **A1 / A2 / A3 / A4 / A5**: different definitions for one term

* = these terms appear for the first time in the second round, as they were the answer to the question "Are there any other relevant fasting terms?"

** = this term appears for the first time in the second round, as several experts made use of this term in the first round. It was therefore considered relevant for inclusion in the consensus process by the study team

*** = this term appears for the first time in the fifth round as it was considered relevant for inclusion in the consensus process by the study team

Figure S2 Remaining questions discussed during the peer review process. Related to Results and Table 2.

	SHORT-TERM FASTING and LONG-TERM FASTING	Answers (N)	Y (%)	N (%)	A (%)
Peer review prcoess	*According to the accepted definitions, strict water-only fasting regimens as well as all regimens that are not purely fluid-based, such as an FMD or the FX-Mayr cure wouldn't be counted as short-term or long-term fasting, as they do not fall under the definition of fluid-only fasting.*<br><br>**Do you agree / disagree with replacing "fluid-only fasting" with "fasting regimens" in the definitions of STF and LTF?**	26	92.31	3.85	3.85
	To distinguish short-term fasting from intermittent fasting (= repetitive fasting regimens lasting up to 48 hours (= 2 days)), we would propose STF to be defined as a fasting regimen lasting 2-3 days.<br><br>**Do you agree / disagree with changing the minimal duration of STF from "24 hours" to "48 hours (= 2 days)"?**	26	73.08	23.08	3.85
Final definitions	**Short-term fasting (STF)** refers to ~~a **fluid-only**~~ fasting regimen<u>s</u> with a <u>duration of **2-3 days.**</u><br>**Prolonged fasting (PF)**, also called **long-term fasting (LTF)**, refers to ~~**any fluid-only**~~ fasting regimen<u>s</u> lasting ≥ 4 consecutive days.				
	**TOTAL/COMPLETE FASTING**	**Answers (N)**	**Y (%)**	**N (%)**	**A (%)**
Peer review prcoess	*If only water is consumed, total fasting and water-only fasting can be equate. These terms have been listed separately, so that the consumption of unsweetened tea or other calorie-free beverages would theoretically also be permitted during a total/complete fast. To make this distinction clearer, we asked the panel if they agree / disagree with the following adaptation of the comment under the term total/complete fasting.*<br><br>***Comment**: Total/complete fasting can be equated with water-only fasting<u>, but it may additionally include tea or other non-caloric beverages.</u>*	26	100	0	0
Final definition	The term **total fast**, or **complete fast**, refers to a fasting regimen where no calories are consumed for a certain period of time.<br>Comment: *Total/complete fasting can be equated with water-only fasting<u>, but it may additionally include tea or other non-caloric beverages.</u>*				
	**MODIFIED FASTING**	**Answers (N)**	**Y (%)**	**N (%)**	**A (%)**
Peer review prcoess	*The threshold of "up to 25%" for the definition of "modified fasting" was set based on the experts opinion, not on clinical data. Unfortunately, there is still no clinical data to justify this decision. This is yet another research gap that we feel should be addressed in the future. In the opinion of our expert panel, modified fasting should definitely be adapted to the individual due to differences in body composition, activity levels, gender and age. For this reason, the definition contains a percentage of energy requirements rather than a fixed caloric threshold. Next to the question of caloric intake, also the question of physical performance during fasting should be addressed.*<br>*Therefore, we asked our panel of experts two questions on the example of a male person who weighs 100 kg and has an activity level of 2.4.*<br>*According to the calculations of our expert who prepared the tables S4, 25% of the energy requirement for such a person aged 30 would correspond to 1175 kcal. For a person aged 50 with the same general conditions, 25% of the energy requirement would correspond to 1115 kcal.*<br><br>**In your opinion, is a physical activity level of 2.4 in line with fasting or should fasters be advised to reduce their activity level during fasting?**	26	61.54	26.92	11.54
	**Would you consider an intake of 1175 kcal (=25% of total energy for a person with 100 kg body weight and an activity level of 2.4) as (modified) fasting?**	26	53.85	26.92	19.23
	As can be seen, the expert opinions differ on both questions so that no definitive answer can be given at this point.				

Y = Agree; N = Disagree; A = Abstention

Figure S3 Energy requirement calculations to ease the clinical application of modified fasting regimens. Related to Results and Table 2.

For illustration purposes and as a general reference guide, weight-maintenance needs were estimated using the Mifflin-St. Jeor equation (73, 74) and a wide range of body mass, activity factor, and age values. For these calculations, height values were specified as the 50th percentile values reported by the World Health Organization (I.e., 163.2 cm for females and 176.5 cm for males).

A 25% of Estimated Weight-Maintenance Needs for a 30-Year-Old.

		Activity Factor							
**Body Mass (kg)**	**Sex**	**1,0**	**1,2**	**1,4**	**1,6**	**1,8**	**2,0**	**2,2**	**2,4**
40	F	277	333	388	444	499	555	610	665
	M	340	407	475	543	611	679	747	815
50	F	302	363	423	484	544	605	665	725
	M	365	437	510	583	656	729	802	875
60	F	327	393	458	524	589	655	720	785
	M	390	467	545	623	701	779	857	935
70	F	352	423	493	564	634	705	775	845
	M	415	497	580	663	746	829	912	995
80	F	377	453	528	604	679	755	830	905
	M	440	527	615	703	791	879	967	1055
90	F	402	483	563	644	724	805	885	965
	M	465	557	650	743	836	929	1022	1115
100	F	427	513	598	684	769	855	940	1025
	M	490	587	685	783	881	979	1077	1175
110	F	452	543	633	724	814	905	995	1085
	M	515	617	720	823	926	1029	1132	1235
120	F	477	573	668	764	859	955	1050	1145
	M	540	647	755	863	971	1079	1187	1295
130	F	502	603	703	804	904	1005	1105	1205
	M	565	677	790	903	1016	1129	1242	1355
140	F	527	633	738	844	949	1055	1160	1265
	M	590	707	825	943	1061	1179	1297	1415
150	F	552	663	773	884	994	1105	1215	1325
	M	615	737	860	983	1106	1229	1352	1475
160	F	577	693	808	924	1039	1155	1270	1385
	M	640	767	895	1023	1151	1279	1407	1535
170	F	602	723	843	964	1084	1205	1325	1445
	M	665	797	930	1063	1196	1329	1462	1595
180	F	627	753	878	1004	1129	1255	1380	1505
	M	690	827	965	1103	1241	1379	1517	1655

B 25% of Estimated Weight-Maintenance Needs for a 50-Year-Old.

		Activity Factor							
**Body Mass (kg)**	**Sex**	**1,0**	**1,2**	**1,4**	**1,6**	**1,8**	**2,0**	**2,2**	**2,4**
40	F	252	303	353	404	454	505	555	605
	M	315	377	440	503	566	629	692	755
50	F	277	333	388	444	499	555	610	665
	M	340	407	475	543	611	679	747	815
60	F	302	363	423	484	544	605	665	725
	M	365	437	510	583	656	729	802	875
70	F	327	393	458	524	589	655	720	785
	M	390	467	545	623	701	779	857	935
80	F	352	423	493	564	634	705	775	845
	M	415	497	580	663	746	829	912	995
90	F	377	453	528	604	679	755	830	905
	M	440	527	615	703	791	879	967	1055
100	F	402	483	563	644	724	805	885	965
	M	465	557	650	743	836	929	1022	1115
110	F	427	513	598	684	769	855	940	1025
	M	490	587	685	783	881	979	1077	1175
120	F	452	543	633	724	814	905	995	1085
	M	515	617	720	823	926	1029	1132	1235
130	F	477	573	668	764	859	955	1050	1145
	M	540	647	755	863	971	1079	1187	1295
140	F	502	603	703	804	904	1005	1105	1205
	M	565	677	790	903	1016	1129	1242	1355
150	F	527	633	738	844	949	1055	1160	1265
	M	590	707	825	943	1061	1179	1297	1415
160	F	552	663	773	884	994	1105	1215	1325
	M	615	737	860	983	1106	1229	1352	1475
170	F	577	693	808	924	1039	1155	1270	1385
	M	640	767	895	1023	1151	1279	1407	1535
180	F	602	723	843	964	1084	1205	1325	1445
	M	665	797	930	1063	1196	1329	1462	1595

C 25% of Estimated Weight-Maintenance Needs for a 70-year-old

		Activity Factor							
**Body Mass (kg)**	**Sex**	**1,0**	**1,2**	**1,4**	**1,6**	**1,8**	**2,0**	**2,2**	**2,4**
40	F	227	273	318	364	409	455	500	545
	M	290	347	405	463	521	579	637	695
50	F	252	303	353	404	454	505	555	605
	M	315	377	440	503	566	629	692	755
60	F	277	333	388	444	499	555	610	665
	M	340	407	475	543	611	679	747	815

70	F	302	363	423	484	544	605	665	725
	M	365	437	510	583	656	729	802	875
80	F	327	393	458	524	589	655	720	785
	M	390	467	545	623	701	779	857	935
90	F	352	423	493	564	634	705	775	845
	M	415	497	580	663	746	829	912	995
100	F	377	453	528	604	679	755	830	905
	M	440	527	615	703	791	879	967	1055
110	F	402	483	563	644	724	805	885	965
	M	465	557	650	743	836	929	1022	1115
120	F	427	513	598	684	769	855	940	1025
	M	490	587	685	783	881	979	1077	1175
130	F	452	543	633	724	814	905	995	1085
	M	515	617	720	823	926	1029	1132	1235
140	F	477	573	668	764	859	955	1050	1145
	M	540	647	755	863	971	1079	1187	1295
150	F	502	603	703	804	904	1005	1105	1205
	M	565	677	790	903	1016	1129	1242	1355
160	F	527	633	738	844	949	1055	1160	1265
	M	590	707	825	943	1061	1179	1297	1415
170	F	552	663	773	884	994	1105	1215	1325
	M	615	737	860	983	1106	1229	1352	1475
180	F	577	693	808	924	1039	1155	1270	1385
	M	640	767	895	1023	1151	1279	1407	1535

D Kcal Values for Weight Maintenance Needs for a 30-Year Old

		**Activity Factor**							
**Body Mass (kg)**	**Sex**	**1,0**	**1,2**	**1,4**	**1,6**	**1,8**	**2,0**	**2,2**	**2,4**
40	F	1109	1331	1553	1774	1996	2218	2440	2662
	M	1358	1630	1901	2173	2445	2716	2988	3260
50	F	1209	1451	1693	1934	2176	2418	2660	2902
	M	1458	1750	2041	2333	2625	2916	3208	3500
60	F	1309	1571	1833	2094	2356	2618	2880	3142
	M	1558	1870	2181	2493	2805	3116	3428	3740
70	F	1409	1691	1973	2254	2536	2818	3100	3382
	M	1658	1990	2321	2653	2985	3316	3648	3980
80	F	1509	1811	2113	2414	2716	3018	3320	3622
	M	1758	2110	2461	2813	3165	3516	3868	4220
90	F	1609	1931	2253	2574	2896	3218	3540	3862
	M	1858	2230	2601	2973	3345	3716	4088	4460
100	F	1709	2051	2393	2734	3076	3418	3760	4102
	M	1958	2350	2741	3133	3525	3916	4308	4700
110	F	1809	2171	2533	2894	3256	3618	3980	4342

	M	2058	2470	2881	3293	3705	4116	4528	4940
120	F	1909	2291	2673	3054	3436	3818	4200	4582
	M	2158	2590	3021	3453	3885	4316	4748	5180
130	F	2009	2411	2813	3214	3616	4018	4420	4822
	M	2258	2710	3161	3613	4065	4516	4968	5420
140	F	2109	2531	2953	3374	3796	4218	4640	5062
	M	2358	2830	3301	3773	4245	4716	5188	5660
150	F	2209	2651	3093	3534	3976	4418	4860	5302
	M	2458	2950	3441	3933	4425	4916	5408	5900
160	F	2309	2771	3233	3694	4156	4618	5080	5542
	M	2558	3070	3581	4093	4605	5116	5628	6140
170	F	2409	2891	3373	3854	4336	4818	5300	5782
	M	2658	3190	3721	4253	4785	5316	5848	6380
180	F	2509	3011	3513	4014	4516	5018	5520	6022
	M	2758	3310	3861	4413	4965	5516	6068	6620

E Kcal Values for Weight Maintenance Needs for a 50-Year Old.

		Activity Factor							
**Body Mass (kg)**	**Sex**	**1,0**	**1,2**	**1,4**	**1,6**	**1,8**	**2,0**	**2,2**	**2,4**
40	F	1009	1211	1413	1614	1816	2018	2220	2422
	M	1258	1510	1761	2013	2265	2516	2768	3020
50	F	1109	1331	1553	1774	1996	2218	2440	2662
	M	1358	1630	1901	2173	2445	2716	2988	3260
60	F	1209	1451	1693	1934	2176	2418	2660	2902
	M	1458	1750	2041	2333	2625	2916	3208	3500
70	F	1309	1571	1833	2094	2356	2618	2880	3142
	M	1558	1870	2181	2493	2805	3116	3428	3740
80	F	1409	1691	1973	2254	2536	2818	3100	3382
	M	1658	1990	2321	2653	2985	3316	3648	3980
90	F	1509	1811	2113	2414	2716	3018	3320	3622
	M	1758	2110	2461	2813	3165	3516	3868	4220
100	F	1609	1931	2253	2574	2896	3218	3540	3862
	M	1858	2230	2601	2973	3345	3716	4088	4460
110	F	1709	2051	2393	2734	3076	3418	3760	4102
	M	1958	2350	2741	3133	3525	3916	4308	4700
120	F	1809	2171	2533	2894	3256	3618	3980	4342
	M	2058	2470	2881	3293	3705	4116	4528	4940
130	F	1909	2291	2673	3054	3436	3818	4200	4582
	M	2158	2590	3021	3453	3885	4316	4748	5180
140	F	2009	2411	2813	3214	3616	4018	4420	4822
	M	2258	2710	3161	3613	4065	4516	4968	5420
150	F	2109	2531	2953	3374	3796	4218	4640	5062
	M	2358	2830	3301	3773	4245	4716	5188	5660

160	F	2209	2651	3093	3534	3976	4418	4860	5302
	M	2458	2950	3441	3933	4425	4916	5408	5900
170	F	2309	2771	3233	3694	4156	4618	5080	5542
	M	2558	3070	3581	4093	4605	5116	5628	6140
180	F	2409	2891	3373	3854	4336	4818	5300	5782
	M	2658	3190	3721	4253	4785	5316	5848	6380

F Kcal Values for Weight Maintenance Needs for a 70-Year Old.

		Activity Factor							
**Body Mass (kg)**	**Sex**	**1,0**	**1,2**	**1,4**	**1,6**	**1,8**	**2,0**	**2,2**	**2,4**
40	F	909	1091	1273	1454	1636	1818	2000	2182
	M	1158	1390	1621	1853	2085	2316	2548	2780
50	F	1009	1211	1413	1614	1816	2018	2220	2422
	M	1258	1510	1761	2013	2265	2516	2768	3020
60	F	1109	1331	1553	1774	1996	2218	2440	2662
	M	1358	1630	1901	2173	2445	2716	2988	3260
70	F	1209	1451	1693	1934	2176	2418	2660	2902
	M	1458	1750	2041	2333	2625	2916	3208	3500
80	F	1309	1571	1833	2094	2356	2618	2880	3142
	M	1558	1870	2181	2493	2805	3116	3428	3740
90	F	1409	1691	1973	2254	2536	2818	3100	3382
	M	1658	1990	2321	2653	2985	3316	3648	3980
100	F	1509	1811	2113	2414	2716	3018	3320	3622
	M	1758	2110	2461	2813	3165	3516	3868	4220
110	F	1609	1931	2253	2574	2896	3218	3540	3862
	M	1858	2230	2601	2973	3345	3716	4088	4460
120	F	1709	2051	2393	2734	3076	3418	3760	4102
	M	1958	2350	2741	3133	3525	3916	4308	4700
130	F	1809	2171	2533	2894	3256	3618	3980	4342
	M	2058	2470	2881	3293	3705	4116	4528	4940
140	F	1909	2291	2673	3054	3436	3818	4200	4582
	M	2158	2590	3021	3453	3885	4316	4748	5180
150	F	2009	2411	2813	3214	3616	4018	4420	4822
	M	2258	2710	3161	3613	4065	4516	4968	5420
160	F	2109	2531	2953	3374	3796	4218	4640	5062
	M	2358	2830	3301	3773	4245	4716	5188	5660
170	F	2209	2651	3093	3534	3976	4418	4860	5302
	M	2458	2950	3441	3933	4425	4916	5408	5900
180	F	2309	2771	3233	3694	4156	4618	5080	5542
	M	2558	3070	3581	4093	4605	5116	5628	6140

Figure S4 Questionnaires 1-5 and transcript of the consensus conference. Related to STAR Methods.

First Questionnaire

Defining Fasting: Finding Common Ground Using the Delphi Method - Round 1

Inclusion criteria for this consensus process

I am a clinician or scientist with ...

1. at least 5 peer-reviewed publications on fasting
2. at least 1 peer-reviewed publication on fasting + 5 years of clinical experience with fasting
3. neither of the above

Answer	n	%
at least 5 peer-reviewed publications on fasting (AO01)	29	85.29%
at least 1 peer-reviewed publication on fasting + 5 years of clinical experience with fasting (AO02)	5	14.71%
neither of the above (AO03)	0	0.00%

Evaluation rules

1. A definition will be accepted in case of agreement of ≥ 70% of participants ("strongly agree" or "agree").
2. A definition will be removed from the list, if ≥ 50% of participants choose "This definition is irrelevant and should be excluded from the consensus process.
3. Suggested changes or new terms to be defined will be taken into consideration for the next survey round when they have been suggested by at least 2 participants.

Definitions

Before each definition is: To what extend do you agree or disagree with this definition?

Selection options: Strongly agree – Agree – Neutral – Disagree – Strongly disagree – This definition is irrelevant and should be excluded from the consensus process

When you click agree or strongly disagree, a comment box opens with the note: Please provide your suggested modifications or **alternative definition** here.

If you click strongly agree or this definition is irrelevant and should be excluded from the consensus process, a comment box opens with the note:
If you have any further thoughts on the given definition that you would like to share, or if you want to explain your choice, please comment here.

Guide for the colors & italics in the comments

- Comments that occur more than once are marked in: orange/light blue/purple/mustard color
- Red writing: when referring to animals
- Red background: important comments regarding the use of the respective definitions
- Purple background: Alternative names for described definitions
- Terms highlighted in green: reached an agreement ≥ 70%
- *Written in italics:* Alternative proposed definition

---

1. Terms concerning dietary and caloric restriction

**DIETARY RESTRICTION (DR)**	**Dietary restriction (DR) comprises of continuous or intermittent** restrictions in caloric intake and/or specific macronutrients and/or restraints of food or food and fluid intake within a specified time frame, without malnutrition. DR thus includes: all types of caloric restriction; fasting regimens such as short-term, long-term, and periodic fasting, intermittent fasting, time-restricted eating or feeding, water-only fasting, therapeutic fasting and fasting-mimicking diets; ketogenic diets; and diets with restrictions of specific macronutrients namely proteins, carbohydrates, or fats.

Answer	n	%
Strongly Agree (AO01)	19	55.88%
Agree (AO02)	8	23.53%
Neutral (AO03)	4	11.76%
Disagree (AO04)	2	5.88%
Strongly Disagree (AO05)	0	0.00%
This definition is irrelevant and should be excluded from the consensus process (AO06)	1	2.94%

STRONGLY AGREE: Further thoughts on given definition / Explanation of choice

P19:	It is important that "without malnutrition" is always emphasized (which it is here).
P13:	I wonder if the word "continuous" would be an alternative to "chronic" in the first sentence of the current definition. To me, continuous and intermittent are opposite terms (I.e., they may match better here), whereas I would think of chronic (long-term) as an opposite term to "acute" (short-term). However, I consider the importance of this wording change relatively minor.
P05:	I think this is a good definition. I have no major objections. Would a vegetarian or vegan diet also be included here? If so, I would mention it by name.
P33:	This s a restriction which is advised and selected by people and not a result of an inability to eat

AGREE: Suggested modifications / alternative definition

P06:	Just a comment on this point: long-term fasting (e.g., 10-day water only fasting every 3 weeks), especially if repeated cyclically in cancer patients, is highly likely to cause malnutrition. Therefore, since i totally agree on the definition of dietary restriction suggested here (chronic or intermittent restrictions in caloric intake and / or specific macronutrients and / or restraints of food intake within a specified time frame, without malnutrition), I would suggest to remove, among the potential CR regimens, long-term fasting.
P22:	The term "without malnutrition" is very hard to define and evaluate, close to impossible to be precise here
P38:	some types of fasting includes fluid/water restriction. So the caloric restriction is not enough. Please add fluid intake
P03:	sound very technical - very dense and a bit confusing - rewording might be necessary
P17:	I would either skip 'without malnutrition' or maybe formulate: *'while to the best of present knowledge excluding bionegative long-term effects of short-term undernutrition*' Long-term fasting regimes are definitely and on purpose designed as a form of undernutrition, one cannot deny that. On the other hand, we intend biopositive effects inside and outside metabolism in its nutritional sense. Inside: carbohydrate and lipid metabolism, outside e.g. metabolism of inflammation. In the European clinical tradition this has been a controversy for a long time: recommend water-fasting as the strongest restriction and therefore maybe with highest effectiveness or modifications like Buchinger fasting with some electrolytres, carbohydrates, some appearance, taste and so forth to facilitate the procedure.
P02:	Fine as above
P24:	The definition should be specifically inclusive of some forms of religious fasting including Ramadan fasting which is practiced by millions of individuals globally.
P32:	I think there is some discrepancy here because TRE may not actually have any dietary restriction.

NEUTRAL: Suggested modifications / alternative definition

P20:	DR is fine to reflect all of these but not sure what point in defining such as vast concept is.
P18:	*Dietary restriction (DR) is the acute, chronic, or intermittent restriction in caloric or caloric and fluid intake, which leads to the therapy of a disease and/or improvement in health.*
P09:	*Dietary restriction (DR)is the acute, chronic, or intermittent restriction in caloric or caloric and fluid intake, with the aim of curing a disease and/or improving one's health.*
P31:	It is OK as is

DISAGREE: Suggested modifications / alternative definition

P21:	Remove "ketogenic diets" from the definition. Including ketogenic diets opens up the possibility of including any diet (mediterranean, vegetarian, etc., etc...) under the umbrella definition of DR
P28:	I DISAGREE WITH "without malnutrition". In fact, diets with restrictions of specific macronutrients can be responsible of malnutrition.

CALORIC RESTRICTION (CR)	Which of these two definitions of **caloric restriction** do you prefer?

Which of these two definitions of **caloric restriction** do you prefer?

- **A001**: Caloric restriction (CR) describes a reduction in energy intake below ad libitum levels without malnutrition. (An individual's total energy intake during caloric restriction by this definition could still exceed his / her daily caloric requirements, as is the case with overweight and obese individuals.)
- **A002**: Caloric restriction (CR) describes a reduction in energy intake below the total caloric intake that would be needed to maintain a healthy body weight, without causing malnutrition. (Total caloric intake here refers to the amount of calories required to maintain a person's "healthy" BMI – depending on their height and age.)

Answer	n	%
Caloric restriction (CR) describes a reduction in energy intake below ad libitum levels without malnutrition. (An individual's total energy intake during caloric restriction by this definition could still exceed his / her daily caloric requirements, as is the case with overweight and obese individuals.) (AO01)	14	41.18%
Caloric restriction (CR) describes a reduction in energy intake below the total caloric intake that would be needed to maintain a healthy body weight, without causing malnutrition. (Total caloric intake here refers to the amount of calories required to maintain a person's "healthy" BMI - depending on their height and age.) (AO02)	20	58.82%

To what extent do you agree or disagree with the definition of **caloric restriction** chosen above?

A001:

Answers	n
Strongly Agree (AO01)	11
Agree (AO02)	2
Neutral (AO03)	0
Disagree (AO04)	1
Strongly Disagree (AO05)	0
This definition is irrelevant and should be excluded from the consensus process (AO06)	0

A002:

Answers	n
Strongly Agree (AO01)	9
Agree (AO02)	9
Neutral (AO03)	2
Disagree (AO04)	0
Strongly Disagree (AO05)	0
This definition is irrelevant and should be excluded from the consensus process (AO06)	0

STRONGLY AGREE: Further thoughts on given definition / Explanation of choice

P19: **A001:** It is not accepted anymore to use the term "obese people/person" but rather "person with obesity/overweight" Caloric restriction is restriction on AL levels regardless of the persons starting bodyweight.

P20: **A001:** calorie restriction is the long term norm, but it really should be energy restriction for global acceptance. That being said I usually use CR as it has historical importance.

AGREE: Suggested modifications / alternative definition

P06: **A002**: Globally i agree with the selected definition of calorie restriction ("a reduction in energy intake below the total caloric intake that would be needed to maintain a healthy body weight, without causing malnutrition"). To make this definition more complete, I suggest to include a functional parameter in the definition, and in particular to state that this "reduction of energy intake" should also result in measurable metabolic effects, including a reduction of blood glucose/growth factor levels and/or an increase in blood/urinary ketone bodies.

P13: **A001**: I see merits to both options provided in the previous question. While I see some potential difficulties with establishing someone's true ad libitum intake, I saw greater challenges with the "healthy body weight" component of the second definition (which is one reason why I chose the first). If the second definition is chosen by consensus, I may have further feedback, particularly a suggestion to revise "a healthy body weight" to another term like "current body weight" or "initial body weight."

P03: **A002:** Sounds technical

P16:

A001: I would like to see some comments regarding CR in animals vs humans. The first definition is probably the reality for most human trials, whereas rodents tend to stop eating when they are full (i.e. they are generally closer to their healthy BMI). /

	(I think this needs to include a statement for animals as well as humans. CR in mice is probably closer to the 2nd definition (healthy BMI) vs. CR in humans is more likely to be described by the first definition.)
P30:	**A002:** The aim of CR is not only to maintain but also to achieve a healthy body weight.
P17:	**A002:** again , malnutrition is on the Agenda, see above. Also, it might make sense to set a Level of caloric restriction, let's say Caloric restriction (CR) describes a reduction in average energy intake below 70% of the total caloric intake that would be needed to maintain a healthy body weight, without causing malnutrition. (Total caloric intake here refers to the amount of calories required to maintain a person's "healthy" BMI – depending on their height and age.) If you want to include IF and esp long-term fasting in one definition, which is charming, you cannot set a range as below. You avoid this by setting a lower level (water fasting means zero energy absolutely and relatively, no matter from where you start)
P31:	**A002:** The second option is OK
P32:	**A002:** I would remove the "healthy BMI" section. Just for weight maintenance is enough.
P34:	There is no good/proper data from a sustained human clinical trial to fully define. The longest trial was the CALERIE study and participants showed benefits at low levels of sustained restriction (less than 14%)
P35:	**A002:** BMI may not be an effective measurement of healthy weight.
**NEUTRAL: Suggested modifications / alternative** definition	
P22:	**A002:** The problem is the term "healthy body weight without malnutrition". We do know that long term caloric restriction often causes immune dysfunction (for example, those people often have to take antibiotics for months in order to get rid of infections).
P05:	**A001:** I am torn on whether CR should be defined relative to (A) maintaining one's current body weight versus (B) maintaining a healthy body weight. I think I lean towards the former and would instead define it as "Caloric restriction (CR) describes a reduction in energy intake below the total caloric intake that would be needed to maintain one's current body weight." Second, the group could also consider whether a minimum threshold needs to be achieved, such as restricting energy intake by 10% or more (relative to eucaloric requirements) is CR and restricting by less than 10% is not.
**DISAGREE: Suggested modifications / alternative** definition	
P21:	**A001:** The definition of CR should not include reductions in caloric intake above that needed to maintain one's current body weight.
**CALORIC RESTRICTION (CR)**	What range of reduction in daily caloric intake should be included in defining **caloric restriction**? 10-25%; 15-40%; 20-40%; 30-40%; 20-50%, other (Freitext)

Answer	n	%
10-25% (AO01)	3	8.82%
15-40% (AO02)	10	29.41%
20-40% (AO03)	3	8.82%
30-40% (AO04)	0	0.00%
20-50% (AO05)	4	11.76%
Other: …	14	41.18%

**Further thoughts / Explanation of choice**	
P19:	**Other**: 10-60%: When we talk about animals, this can be 10-60% food restriction, when we talk about humans this is another story entirely because there is no "one size fits all". Animal studies show 10% food restriction extends lifespan compared to ad libitum, and 60% also extends lifespan. But it is also context dependent ie there are sex and genetic background effects where CR doesn't "work" the same for everyone.
P20:	**Other**: 10-40%: After 40% severe CR should be used.
P06:	**Other**: 20-100%: depending on the duration (number of days) of calorie restriction and the time interval between subsequent calorie restriction cycles
P22:	10-25%: I do not know if you want to discuss this but there is going evidence that extreme fasting/long term CR can give rise to viral infections
P12:	20-50%: Worunter fällt unsere 800 kcal Diät, wenn Bedarf zB 2000 pro Tag?
P13:	**Other**: 10-40%: I think it makes sense for the range to be quite broad so as to be inclusive of varying degrees of caloric restriction. A minimum of 10% seems reasonable, although some could argue for lower. I think the high end of the range is challenging since some individuals with extremely high ad libitum intakes could sustain a large % caloric restriction without malnutrition. I was undecided regarding 40 or 50% for the upper end.
P14:	**Other**: > 20
P18:	**Other**: None: The degree of caloric restriction should be decided individually depending on the treated person's needs and stamina (physical and psychologic)
P09:	**Other**: None: The degree of caloric restriction should be decided individually, depending on the treated person's needs and psychologic and/or physical stamina.
P03:	**Other**: 500 kcal
P15:	**Other**: 10-50%

P30:	15-40%: Caloric intake should be reduced down to the estimated resting metabolic rate. So, the magnitude is also dependent on the profession of the participant.
P11:	**Other**: 10-50%: is there an official definition of CR?
P05:	**Other**: Greater than Either 10% or 15% with the upper limit defined by the level that corresponds to major impairment in humans. – comment: I think the upper limit should be defined as that which corresponds to major health impairments. In the absence of clear evidence on what would constitute the upper limit in humans, I would prefer either 10-40% or 15-40%. The definition should be species-specific, so we should clarify that this is in humans. The best people to ask are Eric Ravussin and those involved in the CALERIE trials in the United States. They should set the definition for the committee.
P17:	**Other**: see above more than 30% - Here I would avoid daily. If you have alternate fasting let's say 5:2 you have to average one week, for some people that chose 10:4 for whatever reason, it's two weeks and so on. I would define the period of an IF method as an important measure very early in the discourse. E.g. the HELENA trial defined this very early and quite easy to understand comparability between the reduction groups in their M&M. By dividing the whole energy uptake in the period by the number of days you get the average daily reduction of an IF.
P33:	**Other:** dont think you can specify – you can't specify would need to say a x% calorie restriction need to decide if want to use the correct terms of enegy restriction rather than the colloquial term calorie restriction
P34:	**Other:** for humans 10-30%, animals 10-50% - For humans. there are no strong data to define what is possible/feasible in the long-term. It would be important to start thinking on long vs short term uses of CR in humans for rodents it is becoming crystal clear that sex, diet, strain and age of onset should define the % of restriction tolerated......i would expect the same for higher mamals

STARVATION

Starvation describes an insufficient nutrient supply based on the abstinence from foods and / or beverages, which is neither voluntary nor controlled. Prolonged starvation can lead to organ failure and / or death.

When used in the context of animal studies, the term fasting usually refers to starvation. We propose starvation as the more exact wording in the animal context.

Answer	n	%
Strongly Agree (AO01)	16	47.06%
Agree (AO02)	7	20.59%
Neutral (AO03)	3	8.82%
Disagree (AO04)	5	14.71%
Strongly Disagree (AO05)	2	5.88%
This definition is irrelevant and should be excluded from the consensus process (AO06)	1	2.94%

AGREE: Suggested modifications / alternative definition

P13:	I recommend rewording the initial portion of the definition to: "*Starvation describes an insufficient nutrient supply due to the abstinence...*" (I.e., replacing "based on" with "due to").
P23:	I do not understand what you mean by "controlled". Starvation is rarely voluntary, but it is usually very well controlled in animal studies, isn't it?
P03:	We should discuss, if information about animals should be covered in a separate chapter
P26:	*Starvation describes a severe, insufficient nutrient supply based on the abstinence from foods and/or beverages, which is neither voluntary nor controlled. Prolonged starvation can lead to malnuturition, organ failure and /or death.*
P16:	I would change this to *caloric beverages. Water might be considered a beverage but is still offered in most starvation experiments. /<br>(Starvation describes an insufficient nutrient supply based on the abstinence from foods and / or caloric beverages, which is neither voluntary nor controlled. Prolonged starvation can lead to organ failure and / or death. I suggest including "caloric beverages" since almost all starvation/fasting studies supply water to the animals.)
P15:	I agree with this definition for animals. For humans, I would say that it can either be voluntary or involuntary and can be controlled or not controlled.
P31:	You should probably include some modifier like short-term or temporary so people don't think we are starving animals to death.

NEUTRAL: Suggested modifications / alternative definition

P20:	Agree if >24h
P11:	These are two different statements: 1. Starvation: strongly agree, 2. Animals: disagree
P35:	*Starvation is the destructive process that occurs after the labile reserves of the body have been depleted and damage and eventually death ensue. Prior to this depletion, fasting would be more descriptive.*

DISAGREE: Suggested modifications / alternative definition

P19:	This is difficult as animal welfare people will not like "starvation" due to the negative connotations associated with it in humans. Also, in animal research, you don't starve them until death. Endpoints are predefined, so in this case starvation is not appropriate, and fasting should be used.
P22:	"neither voluntary nor controlled. " Well, you know people to water fasting (avoidance o drinking), which is probably bad but voluntary. "When used in the context of animal studies, the term fasting usually refers to starvation. " No. There is a plethora of animal studies where fasting prolongs lifespan: This is therefore not detrimental (which insinuates the word starvation)

P28:	the term “abstinence” indicates a voluntary action.
P05:	You can voluntarily starve, so I would remove this wording. Second, starvation implies impairment of function to distinguish it from caloric restriction. It is important to state this in the definition. So I would suggest the following: “*Starvation describes insufficient energy and/or nutrient intake that leads to significant and serious impairment of health. Prolonged starvation can lead to organ failure and/or death*.”
P01:	Fasting in animal studies is of course not voluntary but may be not severe enough to name it as starvation the second paragraph should be : “... *the term fasting frequently refers more to starvation. Here, we propose starvation as the more exact wording in the animal context*.
**STRONGLY DISAGREE: Suggested modifications / alternative definition**	
P21:	As stated this definition of ‘starvation’ implies that caloric restriction studies in animals are ‘starvation studies’ and that prolonged caloric restriction/starvation leads to death! In fact, prolonged caloric restriction improves and health and extends lifespan in animals
P34:	in most animal studies, the creatures are provided water ad libitum are the periods of forced fasting are typically below the threshold that will cause them organ failure and / or death. Most ACUC's review boards in research centers will not approve protocols that can harm animals.
**IRRELEVANT DEFINITION: Further thoughts on given definition / Explanation of choice**	
P25:	starvation can be voluntary as in hunger protest

General terms concerning fasting

FASTING

Fasting refers to a voluntary abstinence from some or all foods and / or beverages for therapeutic, spiritual, or political reasons.

Answer	n	%
Strongly Agree (AO01)	19	55.88%
Agree (AO02)	11	32.35%
Neutral (AO03)	2	5.88%
Disagree (AO04)	1	2.94%
Strongly Disagree (AO05)	1	2.94%
This definition is irrelevant and should be excluded from the consensus process (AO06)	0	0.00%

**STRONGLY AGREE: Further thoughts on given definition / Explanation of choice**	
P15:	The one caveat I suggest is that if fasting is used for some foods or beverages that the specific items or classes of items should be noted along with the use of the word fasting. If the term is used alone then it should generally be assumed that it is total or complete fasting from caloric intake, abstaining voluntarily from all foods and beverages (except water or non-caloric fluids, although I tend to say water-only fasting when water is allowed or consumed).
P33:	could use the term total fast or partial fast
**AGREE Suggested modifications / alternative definition**	
P20:	none
P13:	I would recommend including a reference to “other reasons” to be as inclusive as possible. For example: “...for therapeutic, spiritual, or other reasons.” This way, we would not only be limited to certain categories of reasons for someone performing fasting.
P14:	Remove political reasons
P23:	it can also be done for preventive reasons; the and/or is not correct, as I wouldn’t call abstinence from beverages alone fasting.
P28:	“religious reason” can be added
P30:	I would not include 24h abstinence from water intake in the definition of fasting.
P11:	Solid food instead of some
P17:	omit political: quite a few of the ‘political fasters’ ended with death or serious health damage. There is no way to comply with our definition before. If the political faster does not get closer to his goal, he will continue in most cases, while we would advice a patient or a healthy person to stop. Extend to ‘new experiences with their body and mind’, for those who do not intend spiritual effects. Many like the ‘ease’ of the body, inhanced creativity, better sleep, whatever. The majority of the maybe 5.000 successful fasters I have seen in my professional life would object to intending spiritual experiences. They might have had them , but not consciously and as intended. At the moment this is an agreeableable association, but still for a minority at least in Western countries.
P24:	There should be specific mention of religious and cultural reasons.
P34:	voluntary for humans, the rest of organism that our field uses it will be imposed
**NEUTRAL: Suggested modifications / alternative definition**	
P05:	The definition needs to allow water to be consumed and should say all foods (not just some foods). I also suggest a time limit. I suggest that the definition be revised to: “*Fasting refers to a voluntary abstinence from all food and calorie-containing beverages for at least 14 hours*.”

P35:	*Fasting is the complete abstinence of off substances, except pure water.*
**DISAGREE: Suggested modifications / alternative definition**	
P31:	*Fasting refers to a voluntary abstinence from all foods and / or beverages for therapeutic, spiritual, or political reasons.*
**STRONGLY DISAGREE: Suggested modifications / alternative definition**	
P21:	Definition should read: *Fasting refers to voluntary abstinence from energy intake for therapeutic, spiritual, or political reasons.*
**WATER-ONLY FASTING**	**Water-only fasting** refers to a fasting regimen, where only water (and sometimes tea) is consumed for a certain period of time, usually ≥ 2 days.

Answer	n	%
Strongly Agree (AO01)	17	50.00%
Agree (AO02)	10	29.41%
Neutral (AO03)	3	8.82%
Disagree (AO04)	4	11.76%
Strongly Disagree (AO05)	0	0.00%
This definition is irrelevant and should be excluded from the consensus process (AO06)	0	0.00%

**AGREE Suggested modifications / alternative definition**	
P19:	Need to include that it is "non caloric" beverages
P06:	In addition to water and tea, I would also include in the definition the use of non-caloric beverages
P08:	Sometimes for shorter timeframes. I am suggesting that water-only fasting could be applied for periods of 24-48 hours, according to BMI, metabolic targets and co-morbidities (i.e. diabetes)
P22:	add coffee
P21:	remove "sometimes tea' from the definition
P25:	zero-calorie water-only fasting
P38:	No suggestions
(P16:	I would remove the "> 2 days" from this statement because people may also choose to just consume water during IF regiments for 24 hours.)
P15:	I would remove the period of time and just say *"...where only water (and sometimes tea) is consumed."*
P05:	I'm not sure what I prefer here yet. This seems reasonable enough. What about instead calling this "prolonged fasting"?
P32:	calorie free beverages instead of tea
P35:	*Water-only fasting is the complete abstinence from all substances except pure water.*
**NEUTRAL: Suggested modifications / alternative definition**	
P26:	I would remove the sometimes tea parenthesis
P28:	Is the duration of the fasting (≥ 2 days) necessary in the definition?
P33:	assume other drink are allowed not just water / tea
**DISAGREE: Suggested modifications / alternative definition**	
P20:	Shouldn't need it. If you eat anything except water its not fasting. That being said I except black tea/coffee for compliance in my studies
P13:	I think the reference to tea and the duration should be removed. This would yield a more general definition, like: "*Water-only fasting refers to a fasting regimen, where only water is consumed for a certain period of time*." The addition of tea is confusing since it seems to contradict the term and also introduces herbal/botanical compounds with biological activity. The timeframe is better removed so not to limit the definition unnecessarily.
P31:	I propose you make it >=24 hours, not 2 days.
P34:	Water only, should be water only. If not we will have to go down the road of define what type of tea, how often and why not other types of coffees or herbal infusions
**WATER-ONLY FASTING**	What kind of water should be consumed in **water-only fasting**?<br>• distilled water<br>• mineralized water<br>• there should be no specific requirements for the consumed water

Answer	n	%
distilled water (011)	2	5.88%
mineralized water (012)	10	29.41%
there should be no specific requirements for the consumed water (013)	22	64.71%

**Further thoughts / Explanation of choice**	
P22:	***Mineralized water*** – Distilled water can kill you

P12:	***Mineralized water*** – Gefahr der Hyponatriämie, Herzrhytmusstörungen
P25:	***No specific*** – there are flavored waters, sports drink waters, carbonated waters. Should clarify if all these are included
P16:	***No specific*** – Unfamiliar if there are studies that show that distilled vs mineralized water is better. There might be also socio-cultural factors for water availability/choice / (I think which water is consumed is very culture/country-specific and I would allow both.)
P30:	***Mineralized water*** – Tap water could also be fine. It depends on the source of tap water.
P28:	***Mineralized water*** – Distilled water can be dangerous. Minerals and electrolytes are necessary.
P32:	***No specific*** – black coffee, tea, diet soda should all be included.
P33:	***No specific*** – water is water- sounds very unscientific to state types of water
P34:	***Mineralized water*** – tap water
P35:	***Distilled water*** - There are advantages to supplemented fasting in that known rate limited micronutrients can be used as markers for depletion. Potassium and sodium are examples. In supplemented fasting these known and easily monitored nutrients are sensitive and reliable markers of depletion. If you supplement these you may experience unrecognized depletion of other less sensitive and unmonitored nutrients. We have the experience of over 21,000 supplemented water-only fasting in human subjects using distilled water only. (see fasting safety study Is Fasting Safe)
**FLUID-ONLY FASTING**	**Fluid-only fasting** refers to a fasting regimen, whereby only non-caloric to low-caloric beverages are consumed for a limited period of time. Water and herbal tea may be consumed *ad libitum*. In addition, clear vegetable broth as well as vegetable- and / or fruit juice may be consumed up to a maximum of 500 kcal per day in total.

Answer	n	%
Strongly Agree (AO01)	13	38.24%
Agree (AO02)	9	26.47%
Neutral (AO03)	5	14.71%
Disagree (AO04)	3	8.82%
Strongly Disagree (AO05)	1	2.94%
This definition is irrelevant and should be excluded from the consensus process (AO06)	3	8.82%

AGREE: Suggested modifications / alternative definition

P19:	Suggest including black coffee (no milk/sugar/sweetner)
P22:	Add coffee
P14:	Perhaps remove kcal containing beverages
P26:	I would add strained vegetable/fruit juice and avoidance of dairy products.
P16:	500kcal (i.e ¼ of recommended average caloric consumption) seems high for a "fast" / (I would not include fruit juices since carbohydrates are usually very high.)
P05:	The only thing I would change is to replace "may be consumed up to a maximum of 500 kcal per day in total" with "*may be consumed up to a maximum of 800 kcal per day in total*", to be consistent with the definition of a very-low-calorie diet (VLCD).
P31:	It is OK. Perhaps you should limit the maximum kcal to 250 per day total.
P02:	Fine as above
P01:	The 500kcal limit is not scientifically defined. There are for example liquid diets as used in the DIRECT study that have higher calorie intake. I´m not sure how to label these

NEUTRAL: Suggested modifications / alternative definition

P18:	*Fluid-only fasting refers to a fasting regimen, whereby only non-caloric to low-caloric beverages are consumed for a limited period of time up to regulated quantity. In addition, clear vegetable broth as well as vegetable- and / or fruit juice may be consumed up to a maximum of 500 kcal per day in total.*
P09:	*Fluid-only fasting refers to a fasting regimen, whereby only non-caloric to low-caloric beverages are consumed up to a regulated quantity and for a limited period of time. In addition, clear vegetable broth as well as vegetable- and / or fruit juice may be consumed up to a maximum of 500 kcal per day in total.*
P38:	I'm not certain about this
P28:	Sugar free tea
P33:	No comment

DISAGREE: Suggested modifications / alternative definition

P20:	*I use it.*
P13:	I mostly agree with the first sentence, although I think "a limited period of time" could be revised to "a certain period of time" to match the phrasing of the water-only fasting definition. I would also consider revising "herbal tea" to "unsweetened herbal tea" and adding plain coffee. For the second sentence, I think it is important to separate the consumption of calorie-containing broths and juices up to 500 kcal from strict fluid-only fasting and call this something like "modified fluid-only fasting." An example of my recommendation is shown below: *Fluid-only fasting refers to a fasting regimen, whereby only non-caloric or very low-caloric beverages are consumed for a certain period of time. Water,*

	unsweetened herbal tea, and plain coffee may be consumed ad libitum. Modified fluid-only fasting also allows consumption of clear vegetable broth as well as vegetable- and / or fruit juice up to a maximum of 500 kcal per day in total.
P25:	500 cal is high for fluids only; if it's for a diabetic and is pure sugar it would be unhealthy. I think fluid only fasting should be 0 calories

STRONGLY DISAGREE: Suggested modifications / alternative definition

P23:	I wouldn't call the consumption of 500 kcal/day 'fasting'. I would say *fluid only fasting entails the consumption of water/tea/coffee or zero calorie soft drinks only.*

IRRELEVANT DEFINITION: Further thoughts on given definition / Explanation of choice

P35:	Calling calorie restricted processes fasting only creates confusion.

FLUID-ONLY FASTING

Would you recommend or tolerate the consumption of the following contents during fluid-only fasting?

- Coffee / caffeine

Answer	n	%
Yes (AO01)	24	70.59%
No (AO02)	8	23.53%
No preference (AO03)	2	5.88%

- Buttermilk

**Yes (AO01)**	**8**	**23.53%**
**No (AO02)**	**24**	**70.59%**
**No preference (AO03)**	**2**	**5.88%**

- Honey

**Yes (AO01)**	**9**	**26.47%**
**No (AO02)**	**21**	**61.76%**
**No preference (AO03)**	**4**	**11.76%**

Further thoughts / Explanation of choice

P06:	For all 3: Provided that a maximum of 500 Kcal per day (including all beverages) is consumed
P08:	Coffee: 2-3 cups per day
P12:	Coffee: Wenn verträglich tolerable schwarz ohne Milch
P13:	Buttermilk & Honey: This would be fine for modified fluid-only fasting, per the comments above.
P14:	Coffee: black coffee only
P03:	Buttermilk: 2 x 100ml; Honey: small amount!
P16:	Coffee: black, no creamer, no sugar; Honey: low amounts
P17:	Coffee: defined amount, e.g. max 2 cups, high quality<br>Buttermilk: defined amount, e.g. 1L/d (classical Northern Swiss 41ecision<br>Honey: defined amount like 2 teaspoon measure

FLUID-ONLY FASTING

Would you recommend the use of bowel / colon cleansing during fluid-only fasting?

- Yes, with sodium sulfate (Glauber's salt) or magnesium sulfate (Epsom salt, bitter salts)
- Yes, with colonic irrigation or enema
- Yes, with other means: ……………………….
- No, I do not recommend bowel / colon cleansing
- No preference

Answer	n	%
**No preference (AO01)**	**10**	**29.41%**
**No, I do not recommend bowel / colon cleansing (AO02)**	**16**	**47.06%**
**Yes, with sodium sulfate (Glauber's salt) or magnesium sulfate (Epsom salt, bitter salts) (AO03)**	**5**	**14.71%**
**Yes, with colonic irrigation or enema (AO04)**	**1**	**2.94%**
**Yes, with other means: …**	**2**	**5.88%**

Further thoughts / Explanation of choice

P30:	***Yes, with colonic irrigation or enema –*** I think this should be practiced during a fasting period of at least 7 days.
P11:	***Yes, with other means:*** either or or combination of methods

P05:	**No, - I do not recommend bowel / colon cleansing:** We should be very careful about recommending colonic irrigations, as they have the potential for serious risks.
P17:	**Yes, with other means –** Either sodium sulfate or appr. 2 L of an endoscopy solution, esp. PEG. If 1 L suffices – fine.
P01:	**No preference** – I would use/recommend bowel cleansing in case of previous good experience with it, in case of obstipation and in some cases of irritable bowl syndrome. Otherwise I would leave the decision to the patient after information about the pro´s and cons. In extension to the cited methods I would also list castor oil and polyethylenglykol
P33:	**No preference** – what is the purpose of bowel cleansing - surely this would happen anyway what is this trying to achieve?
P35:	**No, I do not recommend bowel / colon cleansing:** Proper dietary preparation will eliminate the need for bowel stimulation in over 99% of patients undergoing fasting. The use of bowel stimulation during fasting is contraindicated.
**DRY FASTING (DF)**	**Dry Fasting** refers to a fasting regimen, during which an abstinence from all foods and beverages is practiced for a limited period of time.

Answer	n	%
Strongly Agree (AO01)	21	61.76%
Agree (AO02)	1	2.94%
Neutral (AO03)	5	14.71%
Disagree (AO04)	1	2.94%
Strongly Disagree (AO05)	1	2.94%
This definition is irrelevant and should be excluded from the consensus process (A006)	5	14.71%

STRONGLY AGREE: Further thoughts on given definition / Explanation of choice

P13:	I recommend consistency across definitions regarding the period of time. I personally like the phrasing used by the water-only fasting ("for a certain period of time").
P22:	There should be a clear statement against dry fasting
(P16:	I would never recommend this.)
P35:	I would argue that dry fasting is not a physiologically sound procedure. Without adequate water intake the body is unable to effectively mobilize and eliminate the intermediary products of metabolism in the blood stream for elimination in the urine. Dehydration and kidney failure will become problematic. There is no safety study published on dry fasting.

NEUTRAL: Suggested modifi cations / alternative definition

P23:	I was not familiar with the term 'dry fasting'. I would call abstinence from all foods and beverages 'complete fasting'.
P26:	I woud add "a limited period of time" referring to hours and /or days
P28:	I agree
P05:	I recommend using nearly identical language to the language used for regular fasting. Here is my recommendation for the wording for a definition: *"Dry Fasting refers to voluntary abstinence from all foods and beverages, including water, for several hours."*
P02:	I disagree with such a practice
P33:	could be a total diet & fluid fast

DISAGREE: Suggested modifications / alternative definition

P08:	Excluding water and tea

STRONGLY DISAGREE: Suggested modifications / alternative definition

P25:	don't like the term. Would rather use "total fasting"

IRRELEVANT DEFINITION: Further thoughts on given definition / Explanation of choice

P03:	please put in perspective: "starvation diet" – potentially harmfull
P32:	Not sure this is relevant. Just call it Ramadan Fasting

Continuous Fasting Regimens

**SHORT-TERM FASTING (STF)**	**Short-term fasting (STF** also called **short-term caloric restriction,** or, when referring to animals, **short-term starvation (STS)**, is any fasting regimen with a duration of 48 to 72 hours.

Answer	n	%
Strongly Agree (AO01)	13	38.24%
Agree (AO02)	4	11.76%
Neutral (AO03)	3	8.82%
Disagree (AO04)	10	29.41%
Strongly Disagree (AO05)	2	5.88%
This definition is irrelevant and should be excluded from the consensus process (AO06)	2	5.88%

AGREE: Suggested modifications / alternative definition

P19l:	Starvation in animals will have negative connotations and possibly give difficulties when applying for animal licenses/protocols.

P03:	We should discuss, if information about animals should be covered in an separate chapter
P11:	So how do we call 4-5 day fasting?
P34:	Starvation is clearly wrong, it is always associated with extreme suffering or death, due to the lack of food.
**NEUTRAL: Suggested modifications / alternative definition**	
P13:	I recommend removing the phrase “also called short-term caloric restriction.” To me, this would add confusion to the definition. The range of 48 to 72 hours seems fairly small. As mentioned in the next comment box, I think the range of days for short-term fasting and long-term fasting should include all possible durations. With the current definition, the periods from 3 to 5 days are undefined (I.e., neither short-term nor long-term fasting). So, either the duration for short-term fasting should be increased past 72 hours, the duration for long-term fasting should be shortened to >3 days, or there should be another category (intermediate duration fasting, or something similar).
P25:	oppose term starvation for animals; it is politically motivated to imply cruelty to animals. Prefer caloric restriction for animals also
P38:	Not sure
**DISAGREE: Suggested modifications / alternative definition**	
P08:	The timeframe is much shorter from 24-48 hours
P22:	48h is already super long for a mouse
P14:	Time should be 24-72 hours
P23:	I would extend the timeframe to 1-5 days
P16:	I do not agree that STF/STS should be called short-term CR. STCR seems to be more appropriate for short periods of moderate caloric consumption vs. a fast / (STF/STS are to the best of my knowledge approaches that do not allow food consumption for 36-72 hours. Therefore, STF can not be called short-term caloric restriction (which would imply a “restriction” and not deprivation.)
P28:	is this part “also called short-term caloric restriction” necessary? As it can be a fasting from a type of nutrient without caloric restriction.
P15:	I consider fasting for 8 hours to 48 hours to also be short-term fasting. I could agree if it were 16 hours to 72 hours.
P05:	I think the definition needs to be extended to 4 days, should not have separate wording for different species, and should pertain only to water-only fasting. I also don’t like calling it short-term caloric restriction. That means something else to me, as caloric restriction is different from fasting. Here is what I recommend instead: “*Short-term fasting (STF) is any water-only fasting regimen that lasts from 48 hours to 4 days*.” I do not recommend having separate wording in animals; it becomes confusing over time. We should use the same terminology in animals and humans.
P31:	It should be >=24, not 48 hours.
P02:	24-48 heure
**STRONGLY DISAGREE: Suggested modifications / alternative definition**	
P20:	Acute fasting
P21:	2-3 days is long-term fasting in animals. In fact mice often die within 3 days of continuous fasting.
**IRRELEVANT DEFINITION: Further thoughts on given definition / Explanation of choice**	
P33:	these sound like v different things
P35:	Short-term starvation is a misnomer unless you are dealing with a depleted subject.

PROLONGED FASTING (PF)

Prolonged fasting (PF), also called **long-term fasting (LTF)**, refers to any fasting regimen lasting ≥ 5 days.

Answer	n	%
Strongly Agree (AO01)	18	52.94%
Agree (AO02)	5	14.71%
Neutral (AO03)	5	14.71%
Disagree (AO04)	5	14.71%
Strongly Disagree (AO05)	0	0.00%
This definition is irrelevant and should be excluded from the consensus process (AO06)	1	2.94%

**STRONGLY AGREE: Further thoughts on given definition / Explanation of choice**	
P06:	I agree with the definitions of short-term fasting and prolonged fasting. However, these definitions do not saturate all the possible fasting choices, because fasting between 72 and 120 hours is basically excluded from these definitions. For such cases, a new category, such as “intermediate fasting”, could be used.
P34:	For animals is anything longer than 36 hours, for humans anything longer than 48/72 hours
**AGREE: Suggested modifications / alternative definition**	
P13:	I think the range of days for short-term fasting and long-term fasting should include all possible durations. With the current definition, the periods from 3 to 5 days are undefined (I.e., neither short-term nor long-term fasting). So, either the duration for short-term fasting should be increased past 72 hours, the duration for long-term fasting should be shortened to >3 days, or there should be another category (intermediate duration fasting, or something similar).
P26:	I would recommend >4 days

P30:	I would prefer a fasting regimen lasting > 7 days, although a regimen of > or = 5 days should better fit in todays circumstances of life.
P31:	It is OK.
**NEUTRAL: Suggested modifications / alternative definition**	
P21:	Why 5 days? This seems an arbitrary number with no scientific rationale
P38:	No suggestion
P28:	No alternative
P11:	No modification proposed
P35:	While the adaptations to fasting may be prominent by 5 days, for some patients, a 3-4 day fast may qualify as prolonged.
**DISAGREE: Suggested modifications / alternative definition**	
P08:	The timeframe is much shorter from 2-5 days
P22:	Look at Keton bodies; They are already high in most humans after 3 days.
P14:	>72 hours
P25:	if short term is up to 72 hours, long term should be >72 hours
P16:	I would probably say > 3 or 4 days / (I would say lasting more >3 consecutive days.)
**IRRELEVANT DEFINITION: Further thoughts on given definition / Explanation of choice**	
P33:	if fasting can include a low cal diet-or any diet restriction can include any diet if want to say total fast that would be different

PERIODIC FASTING (PF)

Periodic fasting (PF) refers to any fasting regimen that is repeated at regular intervals (periods), such as every day, every week, or every several months.

Answer	n	%
Strongly Agree (AO01)	18	52.94%
Agree (AO02)	6	17.65%
Neutral (AO03)	1	2.94%
Disagree (AO04)	6	17.65%
Strongly Disagree (AO05)	3	8.82%
This definition is irrelevant and should be excluded from the consensus process (AO06)	0	0.00%

**STRONGLY AGREE: Further thoughts on given definition / Explanation of choice**	
P13:	It would be helpful to have a flow chart demonstrating the relationship between different fasting terms (I.e., which terms are grouped under other terms). I wonder if this would be possible in the next round of review to help with the big-picture of these terms.
P34:	should also include a definition of allowance of water/beverage consumption.
**AGREE: Suggested modifications / alternative definition**	
P21:	This falls under the definition of intermittent fasting
P22:	I am not sure about several month, maybe exclude
P28:	I agree
P31:	It is OK.
P02:	OK with definition
**NEUTRAL: Suggested modifications / alternative definition**	
P01:	I would use periodic fasting only repeated fasting regimes when each fasting period comprises at least 4 days
**DISAGREE: Suggested modifications / alternative definition**	
P14:	Every other day, every other week, etc.
P23:	To me periodic fasting is fasting for ≳ 3 days at regular intervals (i.e. once every few weeks of months
P38:	what about fasting from 3-5 days
P15:	I think of periodic fasting as being of lower frequency as well as being repeated at regular intervals, so once every few weeks, month, or several months. I consider every day or once or more per week to be intermittent fasting.
P05:	I would add the requirement that the fasting have a minimum length of time, such as 24 hours, to distinguish it from TRE. Second, I think it should refer to water-only fasting regimens. I strongly think we should implement a 24-hour minimum water-only fast. I would define it as "*Periodic fasting (PF) refers to any 24-hour or longer water-only fasting regimen that is repeated at regular intervals (periods), such as every day, every week or every several months.*" For example, periodic fasting could include fasting 1 day per week, alternate-day fasting, etc. Regardless, we need terminology for intermittent fasting regimens that involve water-only fasting for at least 24 hours at a time. I propose that the term be "periodic fasting." However, if you use the term periodic fasting to refer to something else, we still need a term to describe periodically water-fasting for at least 24 hours at a time.
P33:	suggest keep this for periods of > 5 days and weeks on week off so can use the term intermittent fasting for 1-2 days / week
**STRONGLY DISAGREE: Suggested modifications / alternative definition**	

P20: periodic fasting should be limited to when you fast 2-5 days or more in fortnightly or monthly cycle

Varady: The FMD diet constitutes as periodic fasting. I would change the definition to: *Periodic fasting (PF) refers to any fasting regimen that is repeated at regular intervals (periods), such as every several weeks or months.* Fasting that is repeated every other day is termed intermittent fasting.

P32: This is a Valter Longo term and should apply to a monthly fasting pattern only.

Intermittent Fasting Regimens

INTERMITTENT FASTING (IF)

Intermittent fasting (IF) refers to repetitive fasting periods up to 48 h. IF includes fasting regimens of 1 day per week (6:1), 2 separate or consecutive days per week (5:2), alternate day fasting (ADF) and time-restricted eating (TRE).

Answer	n	%
Strongly Agree (AO01)	24	70.59%
Agree (AO02)	6	17.65%
Neutral (AO03)	0	0.00%
Disagree (AO04)	3	8.82%
Strongly Disagree (AO05)	1	2.94%
This definition is irrelevant and should be excluded from the consensus process (AO06)	0	0.00%

STRONGLY AGREE: Further thoughts on given definition / Explanation of choice

P20: TRE should only be used for cyclical fasting within a day not IF. IMO is is a very different mechanism of action

P13: This is a nice definition! I would just recommend revising "for up to 48 hours" to "of up to 48 hours."

P15: The term "5:2" is unfortunately a term that should not be used in academic literature because it promotes specific products. The term "5:2 diet" is the copyrighted name of a diet and the title of a series of weight loss books (https://kate-harrison.com/52-health) that provides income to specific individuals. Also, the "5:2

fast diet" and the term "5:2" as it applies to fasting (including the "5:2 intermittent fasting diet") are not only copyrighted but also trademarked names used as identifiers of a diet described in a series of weight loss books entitled the "Fast Diet" (https://thefastdiet.co.uk/). The first of book for each of these 5:2 weight loss diets were published in 2013 (within a month of each other). The term "5:2 diet" is used commonly today in academic papers but, because of the origination of the term and the copyrighting/trademarking, it promotes these specific products whose trade names utilize the 5:2 terminology. These are like the trade names of medications. In academic literature, generic names should be used so that a manuscript does not promote these individual products or sets of products. Instead of referring to the 5:2 diet, a generic term like "twice-weekly fasting," "twice-per-week fast," or a similar term should be used as a generic term like a generic drug would be referred to.

P34: water should be allowed

AGREE: Suggested modifications / alternative definition

P22: up to 24h would be better

P16: I think TRE deserves to be a bit more separate from IF.

P05: I would add a lower limit and also restrict the definition to water-only fasting. Here is my alternative definition: "*Intermittent fasting (IF) refers to water-only fasting regimes that involve periodically fasting for 14-48 hours at a time. IF includes fasting regimens of 1 day per week (6:1), 2 separate or consecutive days per week (5:2), alternate-day fasting (ADF), and time-restricted eating (TRE)*."

P31: It is OK.

P24: The definition needs to incorporate "some widely practiced forms of religious fasting such as Ramadan fasting".

P35: The way we use this term in practice is to recommend 12-16 hours of fasting every day. (limit the feeding window to 8 hours for those hoping to lose weight and 12 hours for those needing higher caloric intake and in all cases avoiding eating 3-4 hours before sleep.

DISAGREE: Suggested modifications / alternative definition

P06: I believe that the different definitions of fasting regimens should be based not only on the duration of fasting, type of foods/beverages allowed/not allowed and time intervals between fasting periods, but they should also take into account the expected biological modifications induced by these regimens. In this case, I would restrict the definition of *Intermittent fasting (IF)* to "*repetitive fasting periods for up to 48 hours. IF includes fasting regimens of 1 day per week (6:1) or 2 separate or consecutive days per week (5:2)",* which have higher chances to result in specific metabolic effects, such as a reduction of blood glucose and growth factor levels. Alternate-day fasting (ADF) could fit this definition, but it cannot be applied to intermittent fasting, because it would necessarily imply at least a total of 72 hours of fasting per week (e.g., Monday, Wednesday, Friday).

P23: *Intermittend fasting refers to repetitive fasting periods for 24-72 hours at an interval of max 1 week*

P28:

- Ramadan fasting consists on fasting everyday for +/- 14 hours over 24 hours during one month. It can also be considered as an intermittent fasting.
- How is TRE defined?

STRONGLY DISAGREE: Suggested modifications / alternative definition

P25: TRE should be classified apart from intermittent fasting.

**ALTERNATE-DAY FASTING (ADF)**	**Alternate day fasting (ADF),** or in animals, **every-other-day feeding (EOD),** refers to alternating a day of eating *ad libitum* and a day of either water-only fasting or a diet very low in calories.

Answer	n	%
Strongly Agree (AO01)	18	52.94%
Agree (AO02)	10	29.41%
Neutral (AO03)	1	2.94%
Disagree (AO04)	1	2.94%
Strongly Disagree (AO05)	3	8.82%
This definition is irrelevant and should be excluded from the consensus process (AO06)	1	2.94%

STRONGLY AGREE: Further thoughts on given definition / Explanation of choice

P20:	ADF should not be used if any carbs are consumed within middle of the day as it will activate insulin pathways.
P32:	I don't work in animals so not sure if the animal term is used.

AGREE: Suggested modifications / alternative definition

P13:	Some could argue that ADF could either involve ad libitum or prescribed energy intake on the eating day. So, if desired, the definition could be broadened to: *"Alternate-day fasting (ADF) or in animals, every-other-day feeding (EOD), refers to alternating a day of ad libitum or prescribed eating and a day of either water-only fasting or a diet very low in calories."*
P14:	define very low in calories as 600kcal or less
P23:	I would not allow the consumption of anything except water/tea or coffee if I call something 'fasting'
P03:	We should discuss, if information about animals should be covered in a separate chapter
P26:	I would recommend defining very low in calories to
P11:	very low: how many? Below 400 per day?
P31:	It is OK.
P02:	ADF is with total fast whereas ADMF allows for low calorie diet every other day
P33:	think we should specify the cal intake on the fasting days and make clear that free fluids allowed
P34:	only water, no inclusion of low calorie diet

NEUTRAL: Suggested modifications / alternative definition

P28:	*Alternate-day fasting (ADF) or in animals, every-other-day feeding (EOD), refers to alternating a day of eating ad libitum and a fasting day*. The duration of fasting is not mentioned. Is it 24 hours?

DISAGREE: Suggested modifications / alternative definition

P08:	This is not the case in periodic Orthodox fasting ,where ''non-restrictive '' days are not suggested for ad libitum food intake, rather than increasing total caloric intake to 1500-2000 according sex and age<br>ADF (or ALF) in humans usually refers to restricting caloric intake in 2-3 days a week, returning to usual caloric intake (not ad libitum) in the rest of the week, or even setting an upper limit of 2000 -2500 kcal (women/men) in the non-restrictive days. This is the case in Christian Orthodox fasting, a subtype of religious fasting, which is a rather a specific subtype of ADF. My suggestion would be to include a few questions on religious fasting regimens ,in general, which would add to the scientific value of the consensus and personalize according religious beliefs, which is followed by a large part of individuals which practice IF. I would be willing to help on this task, if it is needed. Religious fasting practices, are a bit different by those followed for health reasons (i.e chrononutrition followed in Ramadan, significantly impacts metabolic effects of this pattern, etc.)

STRONGLY DISAGREE: Suggested modifications / alternative definition

P18:	*Alternate-day fasting (ADF) or in animals, every-other-day feeding (EOD), refers to alternating a day of regulated eating and a day of water-only fasting or a diet very low in calories.*
P09:	*Alternate-day fasting (ADF) or in animals, every-other-day feeding (EOD), refers to alternating a day of regulated eating and a day of either water-only fasting or a diet very low in calories.*
P05:	Here is how I would revise the definition: "*Alternate-day fasting (ADF) refers to alternating days of eating ad libitum with days of water-only fasting.*" I strongly feel that we should not include Alternate-Day Modified Fasting (ADMF) in the definition. I would define **ADMF** as: "*Alternate-day modified fasting (ADMF) refers to alternating days of eating ad libitum with days eating a very-low-calorie diet (VLCD).*

**TIME RESTRICTED EATING (TRE)**	**Time-restricted eating (TRE)** or, when referring to animals, **time-restricted feeding (TRF)**, is a dietary regimen in which food intake is restricted to a specific period of time (usually 4 to 10 hours) during the day, resulting in a daily fasting window of 14 to 20 hours. There is no explicit limit on energy intake during eating or feeding hours.

Answer	n	%
Strongly Agree (AO01)	24	70.59%
Agree (AO02)	7	20.59%
Neutral (AO03)	2	5.88%
Disagree (AO04)	1	2.94%
Strongly Disagree (AO05)	0	0.00%

Answer	n	%
This definition is irrelevant and should be excluded from the consensus process (AO06)	0	0.00%

STRONGLY AGREE: Further thoughts on given definition / Explanation of choice

P33: could also have a definition where TRF can also be energy restricted

AGREE: Suggested modifications / alternative definition

P13: I think the durations needs to be expanded. The most dramatic form of TRE, in my opinion, is one meal a day (OMAD), in which a single meal is consumed. In this case, the day could be viewed as 23 hours of fasting and 1 hour of feeding. Based on this, I recommend using the range of 1 to 10 hours of eating, resulting in a daily fasting window of 14 to 23 hours. I also recommend replacing "explicit" with "inherent."

P25: during the "fasting" time, non-calorie drinks are generally permitted. I also prefer time-restricted feeding for humans rather than time-restricted eating.

P03: We should discuss, if information about animals should be covered in an separate chapter

(P16: I would change "during the day" to something like " a 24h period" since mice largely consume food at night. "Day" might get confused with day light hours...)

P28: It is the same as "fasting" when water and all types of nutrients are concerned .

P05: Again, I think we should move to a single term in humans and animals. However, we should recognize that different species need different minimum durations of fasting. Second, the definition needs mention beverages. Here are my slight modifications to the definition. "*Time-restricted eating (TRE) is a dietary regimen in which the consumption of food and calorie-containing beverages is restricted to a specific period of time. In humans, TRE is defined as restricting food intake to a 10-hour daily period or less, followed by at least 14 hours per day of water-only fasting.*

P31: It is OK.

P01: I would extend the fasting time window to 12-22 hours

NEUTRAL: Suggested modifications / alternative definition

P18: The eating/feeding window should be in accordance with each species' biorhythmus and the quantities consumed should be regulated

P35: not something we use.

DISAGREE: Suggested modifications / alternative definition

P09: *Time-restricted eating (TRE) or, when referring to animals, time-restricted feeding (TRF), is a dietary regimen in which food intake is restricted to a specific period of time (usually 4 to 10 hours) during the day, resulting in a daily fasting window of 14 to 20 hours. The eating or feeding hours should be in accordance with each species' biorhythm and the energy intake regulated.*

Specific Fasting Regimens

THERAPEUTIC / MEDICAL FASTING

Therapeutic fasting, also called **medical fasting**, refers to any fasting regimen that is applied as a therapeutic intervention by a trained physician.

Answer	n	%
Strongly Agree (AO01)	24	70.59%
Agree (AO02)	4	11.76%
Neutral (AO03)	3	8.82%
Disagree (AO04)	1	2.94%
Strongly Disagree (AO05)	0	0.00%
This definition is irrelevant and should be excluded from the consensus process (AO06)	2	5.88%

STRONGLY AGREE: Further thoughts on given definition / Explanation of choice

P13: If desired, you could consider broadening the end of the definition to "...by trained medical personnel."

AGREE: Suggested modifications / alternative definition

P06: I would complement this definition by adding that "*Therapeutic fasting, also called medical fasting, refers to any fasting regimen that is applied as a therapeutic intervention by a trained team of physicians and nutritionists with expertise in the management of fasting regimens in oncology*".

P26: I would add for a certain amount of time

P31: It is OK.

P01: Medical fasting: for me also includes preventive fasting

NEUTRAL: Suggested modifications / alternative definition

P20: No opinion

P28: neutral

P33: not my area of expertise

DISAGREE: Suggested modifications / alternative definition

P05: I would have different definitions of therapeutic and medical fasting. I would define **therapeutic fasting** as "*Therapeutic fasting refers to any fasting regimen that is applied as a therapeutic intervention*." I would define **medical fasting** as:

	"Medical fasting refers to any fasting regimen that is applied as a therapeutic intervention by a trained physician or similar credentialed healthcare provider."
**IRRELEVANT DEFINITION: Further thoughts on given definition / Explanation of choice**	
P32:	All other forms of fating can be used as therapeutic fasting.
P35:	The fact that a physician is involved does not imply that fasting (the complete abstinence of all substances except pure water) is being undertaken.
**BUCHINGER THERAPEUTIC FASTING**	*The following brief definition of* **BUCHINGER therapeutic fasting** *is based on the Expert Panel Update of the 2002 Consensus Guidelines for Fasting Therapy from 2013. If you would like to read the authors' detailed definition, please open the following link in a new tab or window:*<br>*https://www.karger.com/Article/FullText/357602:*<br>**BUCHINGER therapeutic fasting** is a fluid-only fasting regimen, allowing for a maximum of 500 kcal per day and lasting at least 5 days, practiced for the prevention or treatment of diseases as well as to support one's individual health, taking into account a person's medical, psychosocial and spiritual dimensions. It is usually accompanied by bowel / colon cleansing procedures and preceded and followed by a few days of a calorie restricted, easily digestible diet.

Answer	n	%
Strongly Agree (AO01)	18	52.94%
Agree (AO02)	2	5.88%
Neutral (AO03)	7	20..59%
Disagree (AO04)	1	2.94%
Strongly Disagree (AO05)	1	2.94%
This definition is irrelevant and should be excluded from the consensus process (AO06)	5	14.71%

**STRONGLY AGREE: Further thoughts on given definition / Explanation of choice**	
P13:	If this is the exact definition that has been agreed on, then I don't see a reason for us to modify it.
**AGREE: Suggested modifications / alternative definition**	
P30:	Therapeutic fasting should be done on a regular basis (twice a year or so) to achieve a therapeutic goal.
P31:	It is OK.
**NEUTRAL: Suggested modifications / alternative definition**	
P20:	None
P38:	Not familiar with it
P16:	Unfamiliar with the exact definitions for this fast; I would leave this to the experts.
P28:	No alternative
P15:	If any of the individuals involved in this consensus process receives income from the BUCHINGER therapeutic fasting method as an inventor of it or holding the rights to its use, it would be important for this to be disclosed to all others involved.
P33:	not my area of expertise
P35:	Perhaps Buchinger therapeutic calorie restriction would be a more accurate definition.
**DISAGREE: Suggested modifications / alternative definition**	
P06:	The reported definition of therapeutic fasting refers to Buchinger definition (https://www.karger.com/Article/FullText/357602☺. Based on my knowledge of the topic and my clinical/research experience, I believe that therapeutic fasting should include any fasting regimen that is applied as a therapeutic intervention, regardless of the total amount of calories allowed per day, of the types of allowed/not allowed foods and beverages, and the intervals between fasting periods. These variables could be disease specific, and completely different fasting regimens might be useful for the treatment of different types of diseases. Therefore, a strict definition of therapeutic fasting (in terms of total Kcalories/day, type of foods/beverages and time interval between consecutive fasting cycles) that suits all clinical conditions is unlikely to exist. For instance, for the treatment of human malignancies more severe or long-lasting calorie restriction might be needed to achieve the type and amount of biological effects (e.g., metabolic effects, such as percent reduction of blood glucose/growth factor concentration as compared to baseline levels, or immunomodulatory effects) that are expected to result in therapeutic effects.
**STRONGLY DISAGREE: Suggested modifications / alternative definition**	
P23:	Again, I wouldn't allow calories to be consumed when a regimen is called 'fasting'. Fasting is abstinence of food/calories. Allowance of 500 kcal per day should be called 'calorie restriction' or 'fasting mimicking', depending on the composition of the food allowed.
**IRRELEVANT DEFINITION: Further thoughts on given definition / Explanation of choice**	
P25:	don't think our expert panel should address any specific named diets; we are here to define general terms. If I were to design the "x" diet, I wouldn't; expect this panel to comment on it.
P32:	This is periodic fasting and the fasting mimicking diet.

P34: I would stay clear of any of the commercial/for-profit ventures, short of endorsing their use/practice. Let's stay focused on science.

FX-MAYR-THERAPY

FX-Mayr-Therapy or **FX-Mayr Cure** refers to a 3-phase fasting regimen containing elements of water-only fasting, a very low-calorie diet with a training of “proper chewing”, in order to help individuals (re-)gain their sense of satiety and an easily digestible diet towards the end of the treatment. The dietary intervention is accompanied by manual treatments focusing on the abdominal region.

Answer	n	%
Strongly Agree (AO01)	15	44.12%
Agree (AO02)	5	14.71%
Neutral (AO03)	7	20.59%
Disagree (AO04)	0	0.00%
Strongly Disagree (AO05)	0	0.00%
This definition is irrelevant and should be excluded from the consensus process (AO06)	7	20.59%

AGREE: Suggested modifications / alternative definition

P22: I think it is also accompanied by Glauber Salz triggered colon cleaning!?

P05: I don't know much about this approach, so I defer to the other experts.

P17: I do not know whether you have an approved Mayr physician in the team. If not, you should communicate maybe with the ex. comm. of their society. To my knowledge, the 3-phase model may be practiced , but not mandatory. The most important feature of the approach is selection as well as change of diet according to what the physician thinks is in accordance with patient's constitution and particular therapeutic goals. In the Mayr clinics, the kitchen is a core unit, and they offer quite a variety of food ('Milde Ableitungs-Diät'). Water/tea fasting is no longer a necessary start, to my knowledge still taught, but rarely practiced.

P31: It is OK.

P01: "proper eating and slow eating"

NEUTRAL: Suggested modifications / alternative definition

P20: No opinion

P21: I have no opinion on this definition

P23: I am not familiar with this regimen

P38: Not sure

P16: Unfamiliar with the exact definitions for this fast; I would leave this to the experts.

P28: No alternative

P15: If any of the individuals involved in this consensus process receives income from the FX-Mayr-Therapy method as an inventor of it or holding the rights to its use, it would be important for this to be disclosed to all others involved.

IRRELEVANT DEFINITION: Further thoughts on given definition / Explanation of choice

P25: don’t think our expert panel should address any specific named diets; we are here to define general terms. If I were to design the “x” diet, I wouldn’t; expect this panel to comment on it.

P33: sounds very speculative ? any evidence of benefits / harms

P34: I would stay clear of any of the commercial/for-profit ventures, short of endorsing their use/practice. Let's stay focused on science.

FASTING-MIMICKING DIET (FMD)

A **fasting-mimicking diet (FMD)** specifies any diet that aims to induce metabolic effects of fasting. It usually refers to a plant-based, calorie-restricted diet that lasts <u>3 to 5 days</u> and is followed periodically (e.g. once a month). **FMDs** are usually free of refined sugars and low in protein but high in unsaturated fatty acids and complex carbohydrates.

Answer	n	%
Strongly Agree (AO01)	18	52.94%
Agree (AO02)	5	14.71%
Neutral (AO03)	7	20.59%
Disagree (AO04)	1	2.94%
Strongly Disagree (AO05)	0	0.00%
This definition is irrelevant and should be excluded from the consensus-process (AO06)	3	8.82%

STRONGLY AGREE: Further thoughts on given definition / Explanation of choice

P32: Periodic fasting

AGREE: Suggested modifications / alternative definition

P13: I would personally vote to remove or revise the statement that these are high in unsaturated fatty acids and complex carbohydrates. They are high in these items as a percentage of kcals, but still low in absolute intakes due to the very low kcal content of the foods.

P01:	I would add: and has usually 600-1000kcal/day ; 4 to 5 days
P33:	think there can be a number of different forms of this type of diet- we need to include core principles in terms of carbohydrate / protein
P34:	at the end of the day is a pretty similar diet to a TRF with CR incorporated...at least for animal studies. and as above
P35:	By creating a low carbohydrate intake, a fasting mimicking effect of hunger blunting is induced. It does not follow that the other benefits of actual fasting follow.
**NEUTRAL: Suggested modifications / alternative definition**	
P25:	not familiar with this term
P20:	I think periodic fasting covers it, but can understand the use of a catchy title
P28:	No alternative
P11:	No comment
P05:	Protein restriction is also supposed to be part of the FMD. Here is what I propose instead: "*A fasting-mimicking diet (FMD) refers to a plant-based, very-low-calorie and low-protein diet that lasts 3 to 5 days, and is followed periodically (e.g. once a month)*." Honestly, I think the group should largely defer to Valter Longo on how he wants to define this. I think Valter should define how he wants to use this term since he invented it.
P17:	I would restrict this definition to the use of ready-made packages that do not exceed let's say 600 kcal/d and omit the time scale. To my knowledge, most of the FMD-supporters include or do not oppose longer periods than 5 days. Also, a repetetive use is not mandatory. Also, composition by macro- and micronutrient is arbitrarily with the exclusion of refined carbohydrates. This is banal, but beyond that it becomes complex. There are some on the market with only amino acids, no protein, and the amount and spectrum of unsaturated FA is completely open. Most of them contain minerals which I think is important, but also here the differences are immense.
**DISAGREE: Suggested modifications / alternative definition**	
P06:	I partially disagree with this definition. I suggest the following one: "*A fasting-mimicking diet (FMD) specifies any diet that aims to induce the <u>same metabolic effects of water-only fasting</u>. It usually refers to plant-based, calorie-restricted diets that lasts 3 to 5 days and that are followed periodically (e.g. once a month). FMDs are usually free of refined sugars and very low in protein, but with a high percent content in unsaturated fatty acids*."
**IRRELEVANT DEFINITION: Further thoughts on given definition / Explanation of choice**	
P15:	It is not clear that a diet should be referred to as fasting-mimicking because of the origin of the term in a for-profit company that uses the term to promote their specific product (L-Nutra), and because there are many physiologic pathways that are influenced by fasting and there appear to be no literature in which it is validated that FMD activates all or a specific subset of those pathways. In my view, FMD is a marketing term and not a statement of a scientific process or physiologic effects. Also, if any of the individuals involved in this consensus process receives income from a fasting-mimicking diet method as an inventor of it or holding the rights to its use, it would be important for this to be disclosed to all others involved.
**RELIGIOUS FASTING**	**Religious fasting** refers to any fasting regimen that is undertaken as part of a religious practice. Religious fasting thus involves practices such as: dry fasting at certain intervals over 24 hours (e.g. Jewish traditions, The Church of Jesus Christ of Latter-day Saints); intermittent dry fasting (e.g. Ramadan fasting, Bahá'í fasting); time-restricted eating (e.g. Buddhism); and diets restricting certain foods (e.g. Christian orthodox traditions, Daniel fast) if more broadly defined.

Answer	n	%
Strongly Agree (AO01)	26	76.47%
Agree (AO02)	6	17.65%
Neutral (AO03)	2	5.88%
Disagree (AO04)	0	0.00%
Strongly Disagree (AO05)	0	0.00%
This definition is irrelevant and should be excluded from the consensus-process (AO06)	0	0.00%

**STRONGLY AGREE: Further thoughts on given definition / Explanation of choice**	
P18:	Christian orthodox tradition includes intermittent dry fasting and restriction of certain food categories.
**AGREE: Suggested modifications / alternative definition**	
P20:	none
P08:	Christian Orthodox fasting is a combination of TRF and ALF (ALF refers to ADF (Alternate day fasting)<br>email: Typically, all fasting periods in Orthodox christianity ,include restrictive and nonrestrictive days.That means although,all days are considered as components of the fasting period ,there are more ''strict'' (restrictive days),usually Wednesday and Friday . This approach,also results in reducing more caloric intake these specific days (ADF).In specific,a nonrestrictive day (NRD) usually during a weekend of Nativity Fast, allows fish, olive oil, cereals, legumes, nuts, vegetables, fruits, and alcohol and a weekday (Wednesday and Friday)during Great Lent, during which olive oil and fish are additionally excluded from the regular nutritional plan, defined as a restrictive day (RD).This is also reflected in the caloric intake of fasters in the context of ADF (please see attached some of our previous work on this issue).
P09:	*"Religious fasting refers to any fasting regimen that is undertaken as part of a religious practice. Religious fasting thus involves practices such as: dry fasting at certain intervals over 24 hours (e.g. christian orthodox tradition, Jewish tradition);*

	intermittent dry fasting (e.g. christian orthodox tradition, Ramadan, Bahá'í Fasting); time-restricted eating (e.g. Buddhism); and diets restricting certain foods (e.g. Christian orthodox tradition, Daniel fast) if more broadly defined.
P30:	But: Religious fasting is not only related to caloric restriction or specific dietary pattern but includes also spiritual activities to improve cognitive function and well-being.
P05:	I wouldn't include the part on "diets restricting certain foods." Here is how I would modify the definition: "*Religious fasting refers to any fasting regimen that is undertaken as part of a religious practice. Religious fasting thus involves practices such as: dry fasting at certain intervals over 24 hours (e.g. Jewish traditions, The Church of Jesus Christ of Latter-day Saints); intermittent dry fasting (e.g. Ramadan, Bahá'í Fasting); and time-restricted eating (e.g. Buddhism)."*
P31:	It is OK.
**NEUTRAL: Suggested modifications / alternative definition**	
P28:	The difference between "dry fasting at certain intervals over 24 hours " and "intermittent dry fasting" is ufnclear.
P33:	not sure of the relevance of the religious element need to describe what the different diets are when mention Religion can be added for context
**INTERMITTENT DRY FASTING:**	**Intermittent dry fasting (IDF)** refers to intermittent fasting regimens that also involve restrictions of fluid intake during fasting hours. Daily fasting interval time frames may fall outside those usually defined for intermittent fasting, for instance ranging from 9 to 20 hours daily as defined by religious rules depending on daylight hours. Diurnal intermittent dry fasting, as practiced in religious contexts, does not require any defined energy restriction during the nocturnal eating window.

Answer	n	%
Strongly Agree (AO01)	22	64.71%
Agree (AO02)	1	2.94%
Neutral (AO03)	2	5.88%
Disagree (AO04)	4	11.76%
Strongly Disagree (AO05)	1	2.94%
This definition is irrelevant and should be excluded from the consensus process (AO06)	4	11.76%

**AGREE: Suggested modifications / alternative definition**	
P22:	Are there data that dry fasting is beneficial? I could hardly imagine, because you need renal clearance during fasting.
**NEUTRAL: Suggested modifications / alternative definition**	
P28:	*"Intermittent dry fasting (IDF) refers to intermittent fasting regimens that also involve restrictions of fluid intake during fasting hours. Daily fasting interval time frames depend on daylight hours in religious fasting. Diurnal intermittent dry fasting, as practiced in religious contexts, does not require any defined energy restriction during the nocturnal eating window.*"
P11:	No comment
**DISAGREE: Suggested modifications / alternative definition**	
P25:	what religion proscribes this regimen?
P23:	to keep definitions consistent and clear, I would define intermittent dry fasting (better: intermittent complete fasting) exactly the same as intermittent fasting, but including abstinence of fluids. As far as I understand the above definition, it entails time restricted eating.
P05:	I think the definition should require no fluid, not just a decrease in fluid intake, and the definition should be simplified. Here is what I would recommend instead: "Intermittent dry fasting (IDF) refers to intermittent fasting regimens that also involve no fluid intake during fasting hours for a period of at least several hours." As a group, we need to decide the minimum number of hours of no fluid intake that constitutes dry fasting.
P24:	The definition is ambiguous. If eating food allowed, and fluid only restricted?
**STRONGLY DISAGREE: Suggested modifications / alternative definition**	
P20:	none
**IRRELEVANT DEFINITION: Further thoughts on given definition / Explanation of choice**	
P13:	To me, it doesn't seem necessary to include this as its own construct. I believe that the other definitions include the essential information from this form of fasting.
P32:	This should just be part of religious fasting
P33:	this is too confusing ?say ramadan this is time restricted eating & drinking not intermittent as this is a a few days week with a previous definition

Are there other relevant fasting terms?

We are aware that there may be more fasting terms you feel are important to this consensus process than the ones we have selected. If you would like to add any terms to the next questionnaire round, please select as many as you wish of the following, and/or enter a maximum of 2 suggestions in the "Other"-field.

If you would like to provide a rough definition of the terms relevant to you, you are welcome to do so in the comment box next to the selected term.

Answer	n	%
No additional definitions necessary	11	32.35%
Intermittent Energy Restriction (IER)	10	29.41%
Continuous Energy Restriction (CER)	12	35.29%
Daily Energy Restriction (DER)	5	14.71%
Modified Fasting	6	17.65%
Alternate-Day Modified Fasting (ADMF)	8	23.53%
Sub-total Fasting (STF)	1	2.94%
Zero-calorie-diet	3	8.82%
Other:	6	17.65%

Term	Rough definition
**Intermittent Energy Restriction (IER)**	*P20:* should be used if moderate CR is undertaken intermittently i.e. week on week off dieting<br>*P12:* zB 800 kcal Diät für begrenzte Zeit<br>*P13*: I think it is **important to define** how the terms IER and CER relate to the fasting terms already included.<br>*P23:* in analogy with intermittent fasting definition, but with 10-90% calories less than ad lib allowed<br>*P15*: I consider this a synonym of intermittent fasting<br>*P30*: **has to be defined**<br>*P05:* Eating a very-low-calorie diet (VCLD) on a periodic basis<br>*P32:* week on week off, intermittent periods of fasting with periods without<br>*P33:* same as intermittent fasting but an accurate description of what it is
**Continuous Energy Restriction (CER)**	*P20:* more accurately reflect the use of CR when comparing to IF but no strong opinion.<br>*P13*: I think it is **important to define** how the terms IER and CER relate to the fasting terms already included.<br>*P23*: continuous restriction calories by 10-90% of calories consumed ad lib<br>*P15:* I consider this a synonym of caloric restriction<br>*P30*: **has to be defined**<br>*P11:* instead of "chronic"<br>*P05:* Restricting energy intake by a consistent amount on a daily basis.<br>P17: Fixed relative energetic restriction to be applied to any meal that a person has been used before. The degree of restriction is not defined, in ma, whihny trials participants were advised to reduce by 30%, which of course is thus identical to the overall CR<br>*P32:* as another term for CR
**Daily Energy Restriction (DER)**	*P20*: ditto<br>*P13:* I think DER would be useful to mention.<br>*P15:* Not needed<br>*P33:* Lay version of CER
**Modified Fasting**	*P20:* Its not a fast if you are eating without control of macs particularly...<br>*P13:* I think it is important to draw the distinction between true fasting (no calorie intake) and modified fasting (some calorie intake allowed).<br>*P23*: This term is used a lot in the literature, usually for severe calorie restriction (e.g. 800 kcal or less per day, but not necessarily fasting mimicking))<br>*P15:* A very-low-calorie diet of fasting for 24 hours with allowance for a 500-600 kcal meal during the fasting period.<br>*P11:* protein supplemented fasting: no fasting in the general sense<br>*P33*: will get across the idea that not a total fat and that its a low cal diet
**Sub-total Fasting (STF)**	*P15:* Not needed
**Alternate-Day Modified Fasting (ADMF)**	*P20*: Ditto<br>*P13*: This could be mentioned within the definition of ADF.<br>*P23*: Modified fasting (according to the above definition) every other day<br>*P15:* Modified alternate-day fasting (MADF): *a very-low-calorie diet of fasting every-other-day for 24 hours with allowance for a 500-600 kcal meal during the fasting period*<br>*P05:* Alternating days of eating a very-low-calorie diet (VCLD) with days of ad libitum eating<br>*P02:* as per my comment above ("ADF is with total fast wheras ADMF allows for low calorie diet every other day")

**Zero-calorie-diet**	*P22*: at least this should be explained<br>*P15:* Not needed<br>*P17:* A medical practice outside natural or complementary medicine that is hardly practiced any longer and used equivalently to water/tea fasting with pronounced intake of sufficient amounts of fluids, mostly 3 L/d distributesd equally over the day. Limitation to roughly 20 d.
**Other suggestions:**	*P14*: **OMAD: – The One Meal A Day** plan is now popular and this likely should be included, as this would be considered a form of TRF<br>*P09*: **Alternate-day dry fasting** – Alternate-day dry fasting (ADDF) is the alternating between days with proper meals and days with dry fasting.<br>*P05:* **(1) 5:2 Diet. (2) Ramadan Fasting. (3) Weekly Intermittent Energy Restriction. (4) 6:1 Diet.** – (1) Defined as eating a very-low-calorie diet (VCLD) 2 days per week.; (2) RF is defined as dry fasting between sunrise and sunset; (3) defined as alternating periods of restricting energy intake for a at least a week with periods of eating in energy balance or ad libitum.; (4) 6:1 Diet.: defined as fasting for 24 hours once a week<br>*P17*: Did you mention **gruel or whey fasting**? When I tried to scroll back, I was kicked out, so I will not try again! - **Gruel fasting**: 400 mL/d of oak or rice gruel given in three portions, appr. 200 kcal/d by 45 g carbohydrates only. Whey fasting: 1L/d of fresh whey from cow milk in small portions, appr. 250 kcal/d by 45 g carbohydrates, 8 g proteins, 2.4 g fat, in both cases water/tea ad lib.<br>*P33:* **Total diet replacement –** continuous low energy diets (850 kcal) for 12 weeks with diet replacement products<br>*P35*: **Medically Supervised Water-only Fasting** - Medical history, physical exam and laboratory monitoring and the complete abstinence of all substances except pure water in an environment of daily monitoring and complete rest. (minimize gluconeogenesis and maximize the effectiveness of fasting.<br>*P08:* I would include apart from Orthodox fasting, **Ramadan** ,**Buddhist** and **21-Daniel fasting**, which have previous data on their health benefits (please see also one of our previous reviews) .

Second Questionnaire

Defining Fasting: Finding Common Ground Using the Delphi Method - Round 2

Please read the following information thoroughly before starting the second questionnaire.

Evaluation rules

1.: A definition will be accepted in case of agreement of ≥ 70% of participants ("strongly agree" or "agree").
2.: A definition will be removed from the list, if ≥ 50% of participants choose "This definition is irrelevant and should be excluded from the consensus process".
3.: Suggested changes or new terms to be defined will be taken into consideration for the next survey round when they have been suggested by at least 2 participants.

New information for the upcoming Delphi rounds

- To reduce complexity, we have limited the selection to fasting definitions in humans and removed all terms related to animals.
- In case you feel incapable of voting on one or more terms / methods because they don't fall within your area of expertise, we have added a new answer option for each question: "*Due to my specialization, I am not familiar with this term / this method and prefer not to vote on it*". ►We will only count the votes for the answers "strongly agree - agree - neutral - disagree - strongly disagree - this definition is irrelevant and should be excluded from this consensus process".
- We modified the fasting terms and definitions according to your suggestions. In the definitions you will see crossed out words in red and newly added words underlined in blue to make the modifications visible. In some cases, we also provide you with single or summarized comments of the panel experts in anonymized form to help you decide.
- After the first Delphi round, a number of definitions already reached consensus (according to evaluation rule 1). However, we decided to slightly modify some of these definitions post hoc based on your valuable comments (according to evaluation rule 3). We will therefore present both,

the orginal wording and the modified definition(s), in this round. Please decide whether you prefer the modified definition or the original one.

- In the final Delphi round, we will ask you to indicate whether you are generating income in any way from one or more of the fasting methods defined in this process, so that we can disclose this information in the subsequent publications, if appropriate.

This questionnaire is divided into two sections:

In the first section, we present the definitions that received an agreement of < 70% and that we have modified for this next round based on your comments. We have also included new terms & definitions suggested by you. Additionally, you will find answers and links with further information to questions we received in the first round.In the second section, we contrast the definitions that have already received an agreement of ≥ 70% in their current form with the definitions that we have slightly modified based on your comments and ask you to indicate which you prefer.

Please note that you can click on the "Previous" button in the left corner at any time if you wish to revise a statement or change your votes.

Guide for the colors & italics in the comments

- Comments that occur more than once are marked in: orange/light blue/purple
- Purple background: Alternative names for described definitions
- Terms highlighted in green: reached an agreement ≥ 70%
- *Written in italics:* Alternative proposed definition

**Terms concerning dietary and caloric restriction -**Definitions that achieved less than 70% agreement + newly added definitions in this category	
**CALORIC RESTRICTION (CR)**	**CALORIC RESTRICTION (CR)** - In the first round we asked you which of the two definitions of CR you prefer:<br>• **A1: Caloric restriction (CR)** describes a reduction in energy intake below ad libitum levels without malnutrition. (An individual's total energy intake during caloric restriction by this definition could still exceed his / her daily caloric requirements, as is the case with overweight and obese individuals.) ►**41.18%** of the panel experts voted in favour of this definition.<br>• **A2: Caloric restriction (CR)** describes a reduction in energy intake below the total caloric intake that would be needed to maintain a healthy body weight, without causing malnutrition. (Total caloric intake here refers to the amount of calories required to maintain a person's "healthy" BMI – depending on their height and age.) ►**58.92%** of the panel experts voted in favour of this definition.<br><br>Based on these votes, we decided to proceed with the **second definition** and modify it according to your suggested changes. To what extent you agree or disagree with the modified definition?<br><br>**Caloric restriction (CR)** describes a reduction* in energy intake below the total ~~caloric intake~~ amount of calories that would be needed to maintain a ~~healthy~~ person's current body weight, without causing malnutrition. ~~(Total caloric intake here refers to the amount of calories required to maintain a person's "healthy" BMI – depending on their height and age.)~~ CR may also be used to achieve a healthy body weight over time.<br><br>(*range of reduction is to be selected in the next question)

Answer	n	%
Strongly Agree (AO01)	19	57.58%
Agree (AO02)	10	30.30%
Neutral (AO03)	0	0.00%
Disagree (AO04)	3	9.09%
Strongly Disagree (AO05)	1	3.03%

	This definition is irrelevant and should be excluded from the consensus process (AO06)	0	0.00%
	Due to my specialisation, I am not familiar with this term / this method and prefer not to vote on it (AO07)	0	0.00%

AGREE: Suggested modifications / alternative definition

P16:	"CR may also be used to achieve a healthy body weight over time." doesn't need to be here in my opinion
P20:	not sure the last addition is needed. even if you drop 5% thats great for health - this should not be the focus. Eg A bmi of 40 is almost never going to get to 25 without surgery or drugs.
P21:	n/a
P05:	I would delete "CR may also be used to achieve a healthy body weight over time", but I don't feel strongly about this.
P11:	The addition of the second sentence doesn't really make sense to me...
P34:	CR definition, in animal models, doesn't have anything to do to maintain particular body weight. In fact, depending on the strain of animal used, the proportion of change in body weight post CR doesn't follow a linear change.
P17:	I do not like the part 'without causing malnutrition'. It is a matter of time scale: if you fast for several days, you definitely undergo malnutrition for a defined interval. But the way you define the interval and/or add supplements, you hopefully are not malnourished in the end or refurnish declined supplies of any important substance fast.
P01:	i have no modifications to suggest
P32:	remove "heathy"

DISAGREE: Suggested modifications / alternative definition

P06:	Unless physical exercise and total energy expenditure is increased, calorie restriction, especially if chronic, is inevitably associated with loss of body weight and BMI reduction, which should remain the range of normal body weight.
P25:	second statement (in blue) is not relevant to the definition of CR. It simply says a reason one might want to calorie restrict. A definition of a term doesn't include the reason. eg: deoderant: a chemical to prevent body odor. Why a person would use it is not part of the definiton.
P28:	I prefer the former definition: A2

STRONGLY DISAGREE: Suggested modifications / alternative definition

P36:	calorie restriction in the field is viewed as a restriction that is species specific. For mice it is usually 20-40% for humans in most cases 10-25%. It is recognized that CR in humans does cause weight loss, and in fact the clinical studies suggest a heavy weight loss below normal, with many men reaching BMI 19 or so

CALORIC RESTRICTION (CR)

Please select again which range of reduction in daily caloric intake you think should be included in the definition of **caloric restriction**. New options were added after the first round of the survey.

- 10-25 %
- 10-40 %
- 10-50 %
- 15-40 %
- 20-40 %
- 20-50 %
- The range can't be generalized (The degree of caloric restriction should be decided individually depending on the person's age, sex, current body weight, occupation, goal & planned duration of CR)
- Due to my specialization, … .

Answer	n	%
10-25% (AO01)	5	15.15%
10-40% (AO02)	9	27.27%
10-50% (AO03)	4	12.12%
15-40% (AO04)	1	3.03%
20-40% (AO05)	1	3.03%
20-50% (AO06)	1	3.03%
Other	2	6.06%
The range can not be generalized (The degree of caloric restriction should be decided individually depending on the person's age, sex, current body weight, occupation, goal & planned duration of CR) (AO07)	10	30.30%
Due to my specialization, I am not familiar with this topic and prefer not to vote on it (AO08)	0	0.00%

10-25% - Comments

P06:	My answer refers to the case of chronic (daily) calorie restriction

10-50% - Comments

P05:	I would define it as >=10% and leave it as that.
P13:	I see the appeal of the option "The range can not be generalized...", and I imagine many respondents may choose this. However, I think the definition will be more useful if it contains a range. This is why I elected to vote for 10-50%. I think

	10-40% would also be appropriate, but I think a broader range better encompasses the variety of scenarios in which caloric restriction may be employed.
**The range can't be generalized – Comments**	
P15:	If caloric restriction includes intermittent fasting, no amount of energy restriction can be specified in the definition of CR.
P25:	The percent is a function of the reason for losing weight and the speed with which one wants to lose. Again, it is not part of the definition of CR. Arguably, a 1% reduction is also CR, albeit a very slow way to lose weight.
P30:	I think, CR should be close to the estimated (better measured) resting metabolic rate.
**Other – Comments**	
P36:	**See above** → calorie restriction in the field is viewed as a restriction that is species specific. For mice it is usually 20-40% for humans in most cases 10-25%. It is recognized that CR in humans does cause weight loss, and in fact the clinical studies suggest a heavy weight loss below normal, with many men reaching BMI 19 or so
P17:	**at least 30%** - if Buchunger fasting is to be included, this is about 80 to 90% restriction. If water fasting is to be included this is 100% restriction.<br>It seems reasonable to me to set an upper but no lower limit. Here In sugguste 30% reduction.

STARVATION

Starvation describes a catabolic process that occurs when the body's reserves are exhausted after a prolonged period of ~~an~~ insufficient energy and nutrient supply ~~due to the abstinence from foods and / or beverages.~~, ~~which is neither voluntary nor controlled. Prolonged~~ Starvation can lead to serious health impairments, organ failure and ~~/ or~~ death.

Answer	n	%
Strongly Agree (AO01)	22	66.67%
Agree (AO02)	9	27.27%
Neutral (AO03)	0	0.00%
Disagree (AO04)	1	3.03%
Strongly Disagree (AO05)	1	3.03%
This definition is irrelevant and should be excluded from the consensus process (AO06)	0	0.00%
Due to my specialization, I am not familiar with this term / this method and prefer not to vote on it (AO07)	0	0.00%

**AGREE: Suggested modifications / alternative definition**	
P13:	**Modified definition to more directly state what starvation is** ("Starvation is" rather than "Starvation describes"): *Starvation is an extreme form of malnutrition characterized by catabolic processes that occur when the body's energy reserves are exhausted after a prolonged period of insufficient energy and nutrient supply. Starvation can lead to serious health impairments, organ failure and death.*
P02:	*Starvation describes a catabolic process that occurs when the body's reserves are exhausted after a prolonged period of insufficient energy and nutrient supplies. Starvation can lead to serious health impairments, organ failure and / or death.*
P20:	nil
P21:	n/a
P06:	I would add that starvation can lead to "serious health impairments" if prolonged
P28:	I agree
P34:	"prolonged" should stay in the second sentence
P33:	None
**DISAGREE: Suggested modifications / alternative definition**	
Lischka:	Hunger ist ein Signal des Körpers zur Nahrungsaufnahme. Unfreiwilliger und langer Nahrungsverzicht kann zu schweren gesundheitlichen Beeinträchtigungen, Organversagen und Tod führen: Verhungern
**STRONGLY DISAGREE: Suggested modifications / alternative definition**	
P36:	again this is species specific. in simple organism starvation is usually referring to total lack of nutrients for extended period. For humans we probably need to have a consensus workshop which included a language expert to make sure that both the scientific part and the language part are respected before making a decision

CONTINUOUS ENERGY RESTRICTION (CER)

Continuous Energy Restriction (CER), also called **Daily Energy Restriction (DER)**, refers to a daily caloric restriction of about 25-30% of the daily amount of calories required for weight maintenance.

Answer	n	%	% w/o AO07
Strongly Agree (AO01)	13	39.39%	41,94%
Agree (AO02)	7	21.21%	22,58%
Neutral (AO03)	4	12.12%	12,90%
Disagree (AO04)	5	15.15%	16,13%
Strongly Disagree (AO05)	1	3.03%	3,23%
This definition is irrelevant and should be excluded from the consensus process (AO06)	1	3.03%	3,23%

	Due to my specialization, I am not familiar with this term / this method and prefer not to vote on it (AO07)	2	6.06%
	Total w/o A007	31	100,00%

AGREE: Suggested modifications / alternative definition

P14:	The range could be larger, perhaps 15-30%
P16:	because this defines the daily use of CR, I would suggest that the % reduction should be adjusted to the % range of reduction in daily caloric intake that has the majority vote
P30:	As I pointed out before, caloric intake should be reduced to the level of resting metabolic rate. Indeed, this could end up in a reduction by 25-30%, depending also on daily physical activity.
P11:	25-30% seems somewhat arbitrary..
P34:	**10-40%**
P09:	Maybe those terms should not be completely identified. The term Continuous Energy Restriction (CER) could means that the caloric restriction is more than 2 consecutive days.

NEUTRAL: Suggested modifications / alternative definition

P13:	To me, the percentage of energy restriction included in this definition should match the range agreed upon for the definition of caloric restriction (I.e., the first question on this page). I feel like this makes sense because CER/DER is essentially caloric restriction employed each day. For example, if the 10-50% range was selected to be included in the definition of caloric restriction, the definition for CER/DER could be: "*Continuous Energy Restriction (CER), also called Daily Energy Restriction (DER), refers to a daily caloric restriction of about 10-50% of the daily amount of calories required for weight maintenance*." If needed, the definition could be modified to acknowledge that the most common range is about 25-30%. For example: "*Continuous Energy Restriction (CER), also called Daily Energy Restriction (DER), refers to a daily caloric restriction of 10-50%, but commonly about 25-30%, of the daily amount of calories required for weight maintenance.*" Overall, giving a narrow range of about 25-30% without further information – particularly if the definition of caloric restriction ends up being broader – may limit the use of the definition.
P02:	*Continuous Energy Restriction (CER), also called Daily Energy Restriction (DER), refers to a daily caloric restriction* ***up to 40%*** *of the daily amount of calories required for weight maintenance.*
P20:	Do you really need it?
P23:	there are many , in my eyes well conducted and I trials that use 50% reduction in CER. So I would say: *e.g. 30% reduction, in some cases even more...*

DISAGREE: Suggested modifications / alternative definition

P15:	I don't think that continuous energy restriction necessarily has a certain amount of energy restriction assigned to it. It just describes that it is occurring at every meal across the day.
P25:	Take the percent out of the first sentence and make it a separate sentence: "*A 25-30% reduction is considered an effective, yet safe, target*."
P05:	I would define it as daily caloric restriction >=10% with no upper limit.
P33:	Cant actually specify the actual calorie. The term applies to spells of low calorie diets i.e. optifast which provide a 65-70% CER for 12 – 20 weeks. Could say *Continuous Energy Restriction (CER), also called Daily Energy Restriction (DER), refers to a period of daily caloric restriction typically between 25-70 % of the daily amount of calories required for weight maintenance.*
P23:	I think the term should not include a percentage reduction of calories. Any reduction of calories (below the requirement for maintenance of bodyweight) on a daily basis can be defined as continuous or daily energy restriction.

STRONGLY DISAGREE: Suggested modifications / alternative definition

P36:	we should just have 1 term CR, adding more terms to say the same is only going to add to confusion

General terms concerning fasting - Definitions that achieved less than 70% agreement + newly added definitions in this category

TOTAL FAST

The term **total fast**, or **complete fast**, refers to a fasting regimen, where only calorie-free beverages, including water and unsweetened tea, are consumed *ad libitum* for a certain period of time. Historically, prolonged total fasts were used for the therapy of people with obesity under the term **zero-calorie diet**.

Answer	n	%
Strongly Agree (AO01)	23	69.70%
Agree (AO02)	5	15.15%
Neutral (AO03)	2	6.06%
Disagree (AO04)	2	6.06%
Strongly Disagree (AO05)	1	3.03%
This definition is irrelevant and should be excluded from the consensus process (AO06)	0	0.00%
Due to my specialization, I am not familiar with this term / this method and prefer not to vote on it (AO07)	0	0.00%

AGREE: Suggested modifications / alternative definition

P13: The only change I would recommend is to remove or modify the last sentence. If retained, I think it could be simplified to: "*Historically, prolonged total fasts were used under the term zero-calorie diet.*" I personally think the other information (about therapy of people with obesity) is unnecessary detail for a definition.

P18: We strongly feel that zero calorie beverages should be excluded of any current definition of fasting since they have been to be harmful for one's health.

P25: would add to the above in parenthesis:. "*When used to describe religious fasting, the term "total fast" can also mean abstaining from liquids also.*"

P23: I would omit the last sentence and define the period as > 24 hours

NEUTRAL: Suggested modifications / alternative definition

P35: This would be a supplemented complete fast.

P05: I would use the term "water-only fasting" or simply "fasting" instead of total fast or complete fast. Also, I would delete the sentence "Historically, prolonged total fasts were used for the therapy of people with obesity under the term zero-calorie diet."

DISAGREE: Suggested modifications / alternative definition

P09: *The term total fast, or complete fast, refers to a fasting regimen, where only water and unsweetened tea, are consumed up to a regulated quantity for a certain period of time. The term zero-calorie diet should not be confused with fasting, since it includes zero calorie beverages, being harmful for one's health.*

P36: I think it is better to call it water only fasting. Is someone wants to replace it with tea then ok, but it is still essentially a water only fast. The risk with other names is that people will think some calorie can be included (buchinger fast etc)

STRONGLY DISAGREE: Suggested modifications / alternative definition

P28: In total fast, food and beverages should not be consumed such as during Ramadan fasting.

WATER-ONLY FASTING

Please select either the original wording, the modified definition, "neither of the above", if you disagree with the given definitions, or "I am not familiar with this term / this method":

- **A1: Water-only fasting** refers to a fasting regimen, where only water* (and sometimes tea) is consumed for a certain period of time, usually ≥ 2 days.
- **A2: Water-only fasting** refers to a fasting regimen, where only water* ~~(and sometimes tea)~~ is consumed for a certain period of time~~, usually ≥ 2 days~~.

(*kind of water is to be selected in the next question)

Answer	n	%	% w/o AO04
A1: original wording (AO01)	8	24.24%	25,00%
A2: modified definition (AO02)	23	69.70%	71,88%
neither of the above (I disagree with both definitions) (AO03)	1	3.03%	3,13%
Due to my specialization, I am not familiar with this term / this method and prefer not to vote on it (AO04)	1	3.03%	
Total w/o AO04	32		100,00%

I agree with A2: modified definition

P13: I think definition A2 is perfect! It avoids unnecessary detail that would limit the definition and also, appropriately, excludes tea to avoid confusion.

P05: My first choice would be "*Water-only fasting refers to a fasting regimen, where only water* (and sometimes tea) is consumed for a certain period of time*." I think tea should be allowed but there should be no time limit.

P23: I would omit 'for a certain period of time' and define fasting as > 24 hours (to distinguish the term from time restricted eating)

Neither of the above (I disagree with both definitions)

P32: (or *calorie* free beverages) instead of (sometimes tea)

WATER-ONLY FASTING

To complete the above definition of **water-only fasting**, please state again what **kind of water** you think should be consumed in water-only fasting? The main arguments for the different types of water selected in the first round are listed below:

- **distilled water**: *There are advantages to supplemented fasting in that known rate limited micronutrients can be used as markers for depletion. Potassium and sodium are examples. In supplemented fasting these known and easily monitored nutrients are sensitive and reliable markers of depletion. If you supplement these, you may experience unrecognized depletion of other less sensitive and unmonitored nutrients.*
- **mineralized water / tap water**: *Distilled water can be dangerous. Minerals and electrolytes are necessary.*
  Tap water could also be fine. It depends on the source of tap water.

- **there should be no specific requirements for the consumed water**: *Unfamiliar if there are studies that show that distilled vs mineralized water is better. There might be also socio-cultural factors for water availability/choice*

Answer	n	%	% w/o A04
distilled water (A01)	1	3.03%	**3,33%**
mineralized water / tap water (A02)	9	27.27%	**30,00%**
there should be no specific requirements for the consumed water (A03)	20	60.61%	**66,67%**
Due to my specialization, I am not familiar with this topic and prefer not to vote on it (A04)	3	9.09%	
Total w/o A04	30		100,00%

Distilled water – Comments

P35: Although any highly purified water might do, steam distillation is the most effective means of purification.

There should be no specific requirements for the consumed water – Comments

P13: The valid points about potential differences in the source of tap water, availability/choice of water, etc. further supports the idea that there should be no specific requirement for the water source (in my opinion).

P36: these can be specified in anyone s particular practice but only the practices that are widely used should have consensus naming

P25: would specifically say in the definition: *"…where only water (of any kind, plain or calorie-free flavored, carbonated or non-carbonated)…"*

FLUID-ONLY FASTING

We modified the original definition with the help of your comments and prepared two versions. Please select one of them, "neither of the above", if you disagree with the given definitions, or "I am not familiar with this term / this method":

- **Fluid-only fasting** refers to a fasting regimen, whereby only ~~non-caloric to low-caloric~~ beverages are consumed for a ~~limited~~ certain period of time. Water and unsweetened herbal tea may be consumed *ad libitum*. In addition, clear vegetable broth, vegetable- and / or fruit juice, as well as up *to 2 cups of plain coffee and 2 teaspoons of honey* may be consumed ~~up to a maximum of 500 kcal per day in total~~ per day, not exceeding a total of 800 kcal (consistent with the definition of a very low-calorie diet).*
- **Fluid-only fasting** refers to a fasting regimen, whereby only ~~non-caloric to low-caloric~~ beverages are consumed for a ~~limited~~ certain period of time. Water and unsweetened herbal tea may be consumed *ad libitum*. In addition, clear vegetable broth, vegetable- and / or fruit juice, as well as up to 2 cups of plain coffee and 2 teaspoons of honey may be consumed ~~up to a maximum of 500 kcal per day in total~~ per day, not exceeding a total of 250 kcal.*

(*whether – and if so, what kind of – bowel / colon cleansing can be used in fluid-only fasting is to be clarified in the next question)

Answer	n	%	% w/o AO04
**A1 (AO01)**	**9**	**27.27%**	**29,03%**
**A2 (AO02)**	**12**	**36.36%**	**38,71%**
**neither of the above (I disagree with both definitions) (AO03)**	**10**	**30.30%**	**32,26%**
**Due to my specialization, I am not familiar with this term / this method and prefer not to vote on it (AO04)**	**2**	**6.06%**	
**Total w/o AO04**	**31**		**100,00%**

A1

P17: ..not very friendly towards Delphi participants: it takes minutes to grasp a difference between A1 and A2. If there is to be another round, please avoid that and list the corrected versions with emphasis on differences.

A2

P11: Up to now we suggested to stay below 400 calories per day. Do we have reliable date proving the advantage of 250 calories?

P33: very low and low calorie formula diets should be described in the CER definition and not as fluid only fasting

Neither of the above (I disagree with both definitions)

P35: Calling these modified programs fasting is often confusing.

P22: I do not get the point of honey during fasting: This is counterintuitive. You should at least explain that Buchinger may do this due to psychological considerations (e.g. to enhanced compliance)

P13: Of the two choices, I think that the 800 kcal limit makes sense because it is grounded in another well-established definition (I.e., a very low-calorie diet). However, for both options, I think that the specific numerical recommendations for the cups of coffee and teaspoons of honey should be removed. It is unclear why there are specific numerical limits for these items but not for vegetable broth and juices. For consistency and clarity, I think that no individual numerical limits should be

	placed on the specific allowed items. Rather, the single numerical recommendation could be in the form of the kcal limit (I.e., 800 or 250 kcal in these definitions). To me, this improves the flexibility and clarity of the definition. So, the example modified definition for A1 would be: “A1: Fluid-only fasting refers to a fasting regimen, whereby only beverages are consumed for a certain period of time. Water, unsweetened herbal tea, clear vegetable broth, vegetable- and / or fruit juice, and plain coffee may be consumed up to a maximum of 800 kcal per day (consistent with the definition of a very low-calorie diet).*”
P03:	No consumption of psychotropic substances (caffeine) in the form of coffee
P09:	*Fluid-only fasting refers to a fasting regimen, whereby only water and herbal teas are consumed up to a regulated quantity for a certain period of time. In addition, clear vegetable broth, vegetable- and / or fruit juice, as well as up to 2 teaspoons of honey may be consumed up to a maximum of 500 kcal per day in total.*
P18:	*Fluid-only fasting refers to a fasting regimen, whereby only water and herbal tea are consumed for a certain period of time, up to a regulated quantity. In addition, clear vegetable broth, vegetable- and / or fruit juice, as well as up to 2 teaspoons of honey may be consumed up to a maximum of 500 kcal per day in total.*
P36:	that would not make any sense. It does not really matter whether someone acts solid food and then drinks water or drinks the food with the water so fluid fasting should not be used because fasting has nothing to do with the solid or liquid form of calories placed in your mouth
P28:	I suggest: *Fluid-only fasting refers to a fasting regimen, whereby only non-caloric beverages are consumed for a limited certain period of time. Water, vegetable cooking water and unsweetened herbal tea may be consumed ad libitum.*
P23:	I think fasting refers to consumption of zero calories. These definitions will therefore confuse people. If calories are consumed in minute amounts, this is better referred to as calorie restriction or modified fasting.
P01:	i would suggest an uper calorie/day limit of 500kcal

FLUID-ONLY FASTING

To complete the above definition of **fluid-only fasting**, please state again whether you would recommend the use of **bowel / colon cleansing** during fluid-only fasting. The main arguments for the different options selected in the first round are listed below:

- **Yes, with sodium sulfate (Glauber’s salt) or magnesium sulfate (Epsom salt, bitter salts)**
  - *No argument*
- **Yes, with colonic irrigation or enema:**
  - *I think this should be practiced during a fasting period of at least 7 days.*
- **Yes, with other means:**
  - *Either sodium sulfate or appr. 2 L of an endoscopy solution, esp. PEG. If 1 L suffices – fine.*
  - *I would also list castor oil and polyethylenglykol*
  - *combination of methods*
- **No, I do not recommend bowel / colon cleansing:**
  - *We should be very careful about recommending colonic irrigations, as they have the potential for serious risks.*
  - *Proper dietary preparation will eliminate the need for bowel stimulation in over 99% of patients undergoing fasting. The use of bowel stimulation during fasting is contraindicated*
- **No preference**:
  - *I would use/recommend bowel cleansing in case of previous good experience with it, in case of obstipation and in some cases of irritable bowel syndrome. Otherwise, I would leave the decision to the patient after information about the pros and cons.*
  - *what is the purpose of bowel cleansing – surely this would happen anyway what is this trying to achieve?*

Answers	n	%	% w/o AO05
No preference (AO01)	5	15.15%	19,23%
No, I do not recommend bowel / colon cleansing (AO02)	18	54.55%	69,23%
Yes, with sodium sulfate (Glauber’s salt) or magnesium sulfate (Epsom salt, bitter salts) (AO03)	2	6.06%	7,69%
Yes, with colonic irrigation or enema (AO04)	1	3.03%	3,85%
Due to my specialization, I am not familiar with this topic and prefer not to vote on it (AO05)	7	21.21%	
Total w/o AO05	26		100,00%

Yes, with sodium sulfate (Glauber’s salt) or magnesium sulfate (Epsom salt, bitter salts)

P11:	I am missing the option: combination of methods. I usually have my participants choose among different methods like laxative salts, enema and/or colonic irrigation. Endoscopy solutions may be used as well, in case patients tolerate the large amount of fluids. Prior to colonoscopy the application of colon cleansing methods are crucial, so why do we question this for fasting (Keyword: Auto-Intoxication….probably no scientific proof..)

Yes, with colonic irrigation or enema

P30:	I think, bowel / colon cleansing could help built up a healthy microbiota. In this case, duration of fasting should be at least 7 days.
**No, I do not recommend bowel / colon cleansing**	
P13:	To me, it would be much better to acknowledge that some individual practitioners may recommend these procedures to their patients – or that some individual patients may request this from their practitioner – rather than to recommend these procedures for everyone undergoing fluid-only fasting.
P09:	Bowel cleansing, preferably with Epsom salt, is necessary in very rare cases.
P18:	Bowel cleansing, preferably with Epsom salts, is necessary in very rare cases.
**No preference**	
P33:	This does not sound evidence based and cant see any potential benefit. Should only be included in the terminology if there is some evidence to support the rational and benefits
P17:	I think this was my suggestion: "Either sodium sulfate or appr. 2 L of an endoscopy solution, esp. PEG. If 1 L suffices – fine."<br>I cannot find it here.<br>"I would also list castor oil and polyethylenglykol" is similar except for the castor oil which i do not like.
**MODIFIED FASTING**	The term **modified fasting** refers to limiting energy intake to 20-25% of energy needs on fasting days. Modified fasting regimens are often adapted to specific clinical settings and indications, allowing for different complementary or supportive therapeutic interventions.

Answer	n	%
Strongly Agree (AO01)	21	63.64%
Agree (AO02)	4	12.12%
Neutral (AO03)	2	6.06%
Disagree (AO04)	4	12.12%
Strongly Disagree (AO05)	1	3.03%
This definition is irrelevant and should be excluded from the consensus process (AO06)	1	3.03%
Due to my specialization, I am not familiar with this term / this method and prefer not to vote on it (AO07)	0	0.00%

**AGREE: Suggested modifications / alternative definition**	
P14:	Since most people do not know their daily kcal needs, using a percentage may be difficult. May want to use an absolute amount, such as 500kcal per day max.
P13:	I would consider a slight modification (broadening) of the first sentence to: "*The term modified fasting refers to limiting energy intake, typically to 20-25% of energy needs, on fasting days*." The second sentence could be retained in its present form.
P25:	would leave out this term
**NEUTRAL: Suggested modifications / alternative definition**	
P02:	*The term modified fasting refers to limiting energy intake to 20-50% of energy needs on fasting days. Modified fasting regimens are often adapted to specific clinical settings and indications, allowing for different complementary or supportive therapeutic interventions.*
P20:	I think carbs and time of fast are important here, but no real comments.
**DISAGREE: Suggested modifications / alternative definition**	
P05:	I think we should define "*modified fasting*" *as a very-low-calorie diet (VLCD), which is typically defined as <= 800 kcal/day*.<br>The problem with using a threshold of 20-25% is that **there are modified fasting studies already published that use up to ~35% of energy needs. So my second choice for a definition would be up to 35-40% of calories**.
P11:	Why don't we refer to daily caloric intake e.g. 700-800 calories?
P33:	don't think we can specify the exact CR on fasting days Should say *The term modified fasting refers to limiting energy intake to a proportion of the energy needs on fasting days, typically 20-25% of energy needs. Modified fasting regimens are often adapted to specific clinical settings and indications, allowing for different complementary or supportive therapeutic interventions.*
P23:	I would define modified fasting arbitrarily as "consumption of < 600 kcal/day". It is not necessarily adapted to specific clinical settings (although it is often used for reduction of bodyweight), so I would omit the second sentence.
**STRONGLY DISAGREE: Suggested modifications / alternative definition**	
P36:	**25% of calories from the wrong source , lets say proteins could completely reverse many of the effects of fasting**, so unless the term fasting mimicking diet is also included I m against this term
**DRY FASTING (DF)**	For information on the **safety** of **dry fasting**, we have listed a few studies below. To access the content of the studies, please open the links in a new tab or window:<br>• **Leiper und Molla**, 2003: *Effects on health of fluid restriction during fasting in Ramadan*

	Link: https://www.nature.com/articles/1601899<br>• **Maughan und Shirrreffs**, 2012: Hydration and performance during Ramadan Link: https://pubmed.ncbi.nlm.nih.gov/22594964/<br>• **Papagiannopoulos-Vatopaidinos et al., 2020**: *Dry Fasting Physiology: Responses to Hypovolemia and Hypertonicity* Link: https://www.karger.com/Article/Pdf/505201<br>• Liebscher et al., 2021: *Effects of Daytime Dry Fasting on Hydration, Glucose Metabolism and Circadian Phase: A Prospective Exploratory Cohort Study in Bahá'í Volunteers* Link: https://www.ncbi.nlm.nih.gov/pmc/articles/PMC8358295/<br><br>**Dry Fasting** refers to a fasting regimen, during which a voluntary abstinence from all foods and beverages, including water, is practiced for a certain period of time.

Answer	n	%	% w/o AO07
Strongly Agree (AO01)	24	72.73%	75,00%
Agree (AO02)	4	12.12%	12,50%
Neutral (AO03)	1	3.03%	3,13%
Disagree (AO04)	1	3.03%	3,13%
Strongly Disagree (AO05)	0	0.00%	0,00%
This definition is irrelevant and should be excluded from the consensus process (AO06)	2	6.06%	6,25%
Due to my specialization, I am not familiar with this term / this method and prefer not to vote on it (AO07)	1	3.03%	
Total w/o AO07	32		100,00%

STRONGLY AGREE: Suggested modifications / alternative definition

P17:	I still miss a remark on the Mayr practice. I made some suggestions whom to address. Apparently this did not work? **I would be willing to make a short description of their appraisal of fasting if you like.**

AGREE: Suggested modifications / alternative definition

P02:	*Dry Fasting refers to a fasting regimen, during which a voluntary abstinence from all foods and beverages, including water, is practiced **for up to a few days**.*
P28:	it corresponds to total fasting.
P23:	I would suggest "*a regimen whereby no food of drinks (including water) are consumed whatsoever*".

NEUTRAL: Suggested modifications / alternative definition

P11:	I am not familiar with this method, thus I am not sure whether to recommend this at all..

DISAGREE: Suggested modifications / alternative definition

P36:	I don't know enough about dry fasting to determine whether it is safe and for whom so I'm not sure it should be accepted until then

This definition is irrelevant and should be excluded from the consensus process – Comments

P25:	not clear how this differs from regular fasting if you're excluding "food and beverages"

Continuous Fasting Regimens - Definitions that achieved less than 70% agreement in this category

SHORT-TERM FASTING (STF)

Original wording: **Short-term fasting (STF)**, also called short-term caloric restriction, or, when referring to animals, short-term starvation (STS), is any fasting regimen with a duration of 48 to 72 hours.
Various **durations** for short-term fasting have been proposed in the last round. Please select one of the following durations to complete the modified definition of **short-term fasting** below:

Short-term fasting (STF) refers to a fluid-only fasting regimen with a duration of:

- 16 to 72 hours
- 24 to 48 hours
- 48 to 72 hours
- 1- 4 days
- 1 to 5 days
- Due to my specialization, … .

Answer	n	%
16 to 72 hours. (AO01)	10	30.30%
24 to 48 hours. (AO02)	7	21.21%
48 to 72 hours. (AO03)	9	27.27%
1 to 4 days. (AO04)	5	15.15%
1 to 5 days. (AO05)	2	6.06%

Due to my specialization, I am not familiar with this term / this method and prefer not to vote on it (AO06)	0	0.00%

16 to 72 hours - Comments

P28: I consider that 72h is too long for a total fast. It should last less than 24hours.

24 to 48 hours - Comments

P20: Does it need to be 24? Why not 18? Or other… what is the physiological basis for 24h, nefa, ketones are up after 18…

P11: This to me seems quite arbitrary…

48 to 72 hours - Comments

P36: again we need to have a species specific discussion and know what else will be used to define other fasting periods so this can be formalized

1 to 4 days - Comments

P05: My first choice would be 2-4 days. My second choice is 1-4 days. don't feel too strongly about this, other than I don't think that less than 24 hours should be called short-term fasting.

P33: I don't understand when this would be used. Assume this is not replacing intermittent fasting *Short-term fasting (STF) refers to any fasting regimen (total fast or calorie restricted fast) with a duration of:* think it is difficult to define what is short and long based on ketones as the level of ketone formation will depend on whether it is a period of total fast or CR

P23: I would define short term fasting as **1-3 days** actually, since I believe ketones bodies more or less plateau after 3 days. Long term could then be defined as ≥ 4 days.

PROLONGED FASTING (PF)

Original wording: **Prolonged fasting (PF)**, also called long-term fasting (LTF), refers to any fasting regimen lasting ≥ 5 days.

Various durations for prolonged / long-term fasting have been proposed in the last round. We listed the main arguments for the proposed durations below. Please select one of the following durations to complete the modified definition of prolonged / long-term fasting below:

- ≥ 3 consecutive days:
  - *While the adaptations to fasting may be prominent by 5 days, for some patients, a 3-4 day fast may qualify as prolonged.*
  - *Look at Keton bodies; They are already high in most humans after 3 days.*
- ≥ 4 consecutive days: *While the adaptations to fasting may be prominent by 5 days, for some patients, a 3-4 day fast may qualify as prolonged.*
- ≥ 5 consecutive days: *I would prefer a fasting regimen lasting > 7 days, although a regimen ≥ 5 days should better fit in today's circumstances of life*.
- ≥ 7 consecutive days

Prolonged fasting **(PF)**, also called long-term fasting (LTF), refers to any fluid-only fasting regimen lasting:

- ≥ 3 consecutive days.
- ≥ 4 consecutive days.
- ≥ 5 consecutive days.
- ≥ 7 consecutive days.

Answer	n	%	% w/o AO05
≥ 3 consecutive days. (AO01)	13	39.39%	40.63%
≥ 4 consecutive days. (AO02)	9	27.27%	28.13%
≥ 5 consecutive days. (AO03)	9	27.27%	28.13%
≥ 7 consecutive days. (AO04)	1	3.03%	3,.13%
Due to my specialization, I am not familiar with this term / this method and prefer not to vote on it (AO05)	1	3.03%	
Total w/o AO05	32		100.00%

≥ 4 days – Comments

P36: again this is species specific, so a workshop would be needed with many of the experts and a separate discussion for at least mice and humans. **I think intermittent fasting should be 12 to 48 hours and prolonged fasting 48 or more hours. I have written several reviews with other experts on this topic (panda, matron, etc) and we did not hear any complaints from any of the readers**

P33: *long -term fasting (STF) refers to any fasting regimen (total fast or calorie restricted fast) with a duration of:* think it is difficult to define what is short and long based on ketones as the level of ketone formation will depend on whether it is a period of total fast or CR

P23: I would define short term fasting as 1-3 days actually, since I believe ketones bodies more or less plateau after 3 days. Long term could then be defined as ≥ 4 days.

**≥ 5 days – Comments**	
P11:	Dito – This to me seems quite arbitrary...

INTERMEDIATE DURATION FASTING:

Depending on the selected duration of the two fasting regimens "**short-term-**" and "**prolonged / long-term fasting**", should a fasting regimen such as "**intermediate duration fasting**" be defined?

- Yes
- No
- No preference
- Due to my specialization, … .

Answer	n	%	% w/o AO04
Yes (AO01)	11	33,33%	34,38%
No (AO02)	21	63,64%	65,63%
No preference (AO03)	0	0,00%	0,00%
Due to my specialization, I am not familiar with this term / this method and prefer not to vote on it (AO04)	1	3.03%	
Total w/o AO04	32		100,00%

**Yes – Comments:**	
P13:	As implied in the question, this would only be necessary if there ends up being undefined periods of time between short-term and long-term fasting (such as 3-4 days of fasting if >5 days is selected for long-term fasting). I think this definition would be useful since it makes more sense to transition from short to intermediate to long rather than directly from short to long.
**No – Comments:**	
P36:	See above (again this is species specific, so a workshop would be needed with many of the experts and a separate discussion for at least mice and humans. I think intermittent fasting should be 12 to 48 hours and prolonged fasting 48 or more hours. I have written several reviews with other experts on this topic (panda, matron, etc) and we did not hear any complaints from any of the readers)
P33:	cant see a use for this

Intermittent Fasting Regimens Newly added definitions in this category

INTERMITTENT ENERGY RESTRICTION (IER)

Intermittent Energy Restriction (IER) includes periods of caloric restriction alternating with periods of *ad libitum* eating. As such, IER includes fasting regimens like intermittent fasting (IF) and time-restricted eating (TRE).

Answer	n	%	% w/o AO07
Strongly Agree (AO01)	25	75.76%	78,13%
Agree (AO02)	1	3.03%	3,13%
Neutral (AO03)	0	0.00%	0,00%
Disagree (AO04)	3	9.09%	9,38%
Strongly Disagree (AO05)	2	6.06%	6,25%
This definition is irrelevant and should be excluded from the consensus process (AO06)	1	3.03%	3,13%
Due to my specialization, I am not familiar with this term / this method and prefer not to vote on it (AO07)	1	3.03%	
Total w/o AO07	32		100,00%

**STRONGLY AGREE: Suggested modifications / alternative definition**	
P32:	maybe include periodic fasting as well
**AGREE: Suggested modifications / alternative definition**	
P15:	TRE is a form of intermittent fasting because the fasting in TRE occurs intermittently (the intermittent occurrence is simply on a daily basis rather than a weekly or monthly basis).
**DISAGREE: Suggested modifications / alternative definition**	
P36:	I think it is better to continue to call it intermittent fasting but it needs to be defined, and maybe IER can be an additional but different term
P21:	*Intermittent Energy Restriction (IER) includes periods of caloric restriction **sufficient to induce ketosis** alternating with periods of ad libitum **energy intake**. As such, IER includes fasting regimens like intermittent fasting (IF) and time-restricted eating (TRE).*
P28:	*Intermittent Energy Restriction (IER) includes periods of caloric restriction alternating with periods of ad libitum **energy intake**. As such, IER includes fasting regimens like **time-restricted eating (TRE).***
**STRONGLY DISAGREE: Suggested modifications / alternative definition**	

P05:	Time-restricted eating (TRE) is definitely not a form of intermittent energy restriction. To me, IER means eating a low-calorie diet some number of days per week or per month. I would change the definition to: "*Intermittent Energy Restriction (IER) includes periods of caloric restriction alternating with periods of ad libitum energy intake*."
P33:	TRE needs to have a separate definition to IER. Many IER regimens will advise a healthy diet on the non -restricted days – also ? **use the term energy throughout the definitions and not calorie as they seem to be used interchangeably.** *Intermittent Energy Restriction (IER) or intermittent fasting includes periods of caloric restriction alternating with periods of ad libitum* ***energy intake*** *, either normal diet or a different prescribed diet (i.e. healthy eating , med diet)*

ONE MEAL A DAY DIET (OMAD)

Should the term **One Meal a Day diet** (a version of time-restricted eating) be defined separately as part of this consensus process?

- Yes
- No
- No preference
- Due to my specialization, … .

Answer	n	%
**Yes (AO01)**	**14**	**42.42%**
**No (AO02)**	**15**	**45.45%**
**No preference (AO03)**	**4**	**12.12%**
**Due to my specialization, I am not familiar with this term / this method and prefer not to vote on it (AO04)**	**0**	**0.00%**

Yes – Comments:

P13:	While it could be included in the definition of time-restricted eating, I think it is also reasonable to provide a separate definition for this. My recommended definition is something like: "*One Meal a Day diet (OMAD) is form of time-restricted eating in which all daily energy intake is consumed in a single eating occasion. The length of the daily eating window is the duration of this single eating occasion.*"

ALTERNATE-DAY MODIFIED FASTING (ADMF)

It has been proposed to add **alternate-day modified fasting (ADMF)** to the list of terms based on the definition of alternate-day fasting. Please select one of the following durations for this definition.

Alternate-day modified fasting (ADMF) refers to alternating a day of eating *ad libitum* and a day of eating a low-calorie diet with up to:

- 400 kcal.
- 500 kcal.
- 600 kcal.
- 800 kcal.
- 20 to 25 % of the usual energy intake.
- Due to my specialization, … .

Answer	n	%
**400 kcal. (AO01)**	**8**	**24.24%**
**500 kcal. (AO02)**	**4**	**12.12%**
**600 kcal. (AO03)**	**3**	**9.09%**
**800 kcal. (AO04)**	**3**	**9.09%**
**20 to 25% of the usual energy intake. (AO05)**	**15**	**45.45%**
**Due to my specialization, I am not familiar with this term / this method and prefer not to vote on it (AO06)**	**0**	**0.00%**

500 kcal:

P03:	Should be part of the background text

800 kcal:

P05:	Equivalently, I would be in favor of up to ~35% of energy intake on the modified fasting days.
P11:	Does this (or this question) make sense at all?

20 to 25% of the usual energy intake:

P13:	**To me, the use of a relative intake (%) makes the definition much more flexible than absolute (kcal) units due to the widely varying energy needs of humans.**
P36:	a review of the literature is necessary to see what constitute. A calorie limit on the fast day
P20:	But also need to specify what and when it is eaten
P30:	My suggestion is based on the assumption that daily caloric intake matches daily caloric needs.
P28:	I disagree. The term fasting can not be used here. It is rather a calories restriction.
P33:	don't think you can specify one need to put a range of possibilities in the definition
P32:	25% of energy needs for weight maintenance

Specific Fasting Regimens - Definitions that achieved less than 70% agreement + newly added definitions in this category

BUCHINGER THERAPEUTIC FASTING

To what extent do you agree or disagree with this definition?

(Please note that this definition has not been modified, however the last answer option "*Due to my specialization, I am not familiar with this term / this method and prefer not to vote on it.*" Has been added. If applicable to you, please choose this option.)

The following brief definition of **BUCHINGER therapeutic fasting** *is based on the Expert Panel Update of the 2002 Consensus Guidelines for Fasting Therapy from 2013. If you would like to read the authors' detailed definition, please open the following link in a new tab or window* https://www.karger.com/Article/FullText/357602*:*

BUCHINGER therapeutic fasting is a fluid-only fasting regimen, allowing for a maximum of 500 kcal per day and lasting at least 5 days, practiced for the prevention or treatment of diseases as well as to support one's individual health, taking into account a person's medical, psychosocial and spiritual dimensions. It is usually accompanied by bowel / colon cleansing procedures and preceded and followed by a few days of a calorie restricted, easily digestible diet.

Answer	n	%	% w/o AO07
Strongly Agree (AO01)	20	60.61%	86,96%
Agree (AO02)	1	3.03%	4,35%
Neutral (AO03)	0	0.00%	0,00%
Disagree (AO04)	0	0.00%	0,00%
Strongly Disagree (AO05)	0	0.00%	0,00%
This definition is irrelevant and should be excluded from the consensus process (AO06)	2	6.06%	8,70%
Due to my specialization, I am not familiar with this term / this method and prefer not to vote on it (AO07)	10	30.30%	
Total w/o A007	23		100,00%

This definition is irrelevant and should be excluded from the consensus process

P23: I'm not sure if this definition adds to all others. It would if it has been shown to have specific effects other than those documented for other regimens which were described before in more general terms (and therefore be specifically applicable for certain conditions).

FX-MAYR-THERAPY

To what extent do you agree or disagree with this definition?

(Please note that this definition has not been modified, however the last answer option "*Due to my specialization, I am not familiar with this term / this method and prefer not to vote on it.*" Has been added. If applicable to you, please choose this option.)

FX-Mayr-Therapy or **FX-Mayr Cure** refers to a 3-phase fasting regimen containing elements of water-only fasting, a very low-calorie diet with a training of "proper chewing", in order to help individuals (re-)gain their sense of satiety and an easily digestible diet towards the end of the treatment. The dietary intervention is accompanied by manual treatments focusing on the abdominal region.

Answer	n	%	% w/o AO07
Strongly Agree (AO01)	17	51.52%	77,27%
Agree (AO02)	0	0.00%	0,00%
Neutral (AO03)	0	0.00%	0,00%
Disagree (AO04)	1	3.03%	4,55%
Strongly Disagree (AO05)	0	0.00%	0,00%
This definition is irrelevant and should be excluded from the consensus process (AO06)	4	12.12%	18,18%
Due to my specialization, I am not familiar with this term / this method and prefer not to vote on it (AO07)	11	33.33%	
Total w/o A007	22		100,00%

STRONGLY AGREE: any further thoughts

P17: I am sorry, I missed that before. It is hard to overlook the questionnaire.

I still miss a remark on the Mayr practice. I made some suggestions whom to address. Apparently this did not work? I would be willing to make a short description of their appraisal of fasting if you like.

(Kommentar von Runde 1 zu FX Mayr: I do not know whether you have an approved Mayr physician in the team. If not, you should communicate maybe with the ex. Comm. Of their society. To my knowledge, the 3-phase model may be

	practiced , but not mandatory. The most important feature of the approach is selection as well as change of diet according to what the physician thinks is in accordance with patient's constitution and particular therapeutic goals. In the Mayr clinics, the kitchen is a core unit, and they offer quite a variety of food ('Milde Ableitungs-Diät'). Water/tea fasting is no longer a necessary start, to my knowledge still taught, but rarely practiced.)
**DISAGREE: Suggested modifications / alternative definition**	
P01:	in the first phase fasting according to Mayr includes tea and water. In the second phase there are "Kautrainer" like old bread, crackers but also broth and light soups resulting in a daily calorie intake in phase 2 of 350-600 kcal, thus differing a bit to very low calorie diet

FASTING-MIMICKING DIET (FMD)

Below you find the slightly modified definition of the term **Fasting-Mimicking Diet (FMD)** from the first round of the survey, in case you need it to answer the following question(s).

A **Fasting-Mimicking Diet (FMD)** specifies any diet that aims to induce the metabolic effects of fasting. It usually refers to a plant-based, calorie-restricted diet with solid food components that lasts 3 to 5 days and is followed periodically (e.g. once a month). **FMDs** are usually free of refined sugars and low in protein but high in unsaturated fatty acids and complex carbohydrates.

Please indicate whether you consider the inclusion of the term **Fasting-Mimicking Diet** important for this consensus process. The main arguments from the first round of the survey are listed below.

- **Yes.**
  - *I think there can be a number of different forms of this type of diet – we need to include core principles in terms of carbohydrate / protein.*
- **No**
  - *[There is no need to define a fasting-mimicking diet (FMD) as] it falls within the definition of periodic fasting.*
  - *By creating a low carbohydrate intake, a fasting mimicking effect of hunger blunting is induced. It does not follow that the other benefits of actual fasting follow.*
  - *The term fasting-mimicking diet was originally created in a for-profit company that uses the term to promote their specific product (L-Nutra). There are many physiologic pathways that are influenced by fasting and there appears to be no literature which shows that FMD activates all, or a specific subset of those same pathways. FMD is a marketing term and not a statement of a scientific process or physiologic effects.*

Answer	n	%	% w/o AO03
Yes, FMD should be included (AO01)	22	66.67%	68,75%
No, FMD should not be included (AO02)	10	30.30%	31,25%
Due to my specialization, I am not familiar with this term / this method and prefer not to vote on it (AO03)	1	3.03%	
Total w/o A003	32		100,00%

If Yes is clicked, the following additional questions will be asked:

3. How many calories should be consumed as part of the **Fasting-Mimicking Diet**:
- 600-1000 kcal per day
- a maximum of 600 kcal per day

4. Should a **Fasting-Mimicking Diet** per definition include (you can choose more than one answer):
- A low carbohydrate intake
- A low protein intake
- A low intake of fatty acids
- Only a restricted intake of refined sugars and calories

**Yes, FMD should be included**	
P13:	While I agree with the comment that this term seems to be used primarily in relation to the company L-Nutra / ProLon, I think it is important to be as comprehensive as possible. Since there is a growing body of research on FMD, it seems important to include so that the publication(s) resulting from this process are as comprehensive as possible.
P16:	FMD is the scientific term that is used in numerous peer-reviewed publications. The complaint that FMD is promoting a commercially available product is wrong; this product is sold as "ProLon" (not FMD) by L-Nutra
P36:	there is absolutely a need to include a FMD since it is not simply a prolonged fasting but a very special type of prolonged fasting that actually allow people to eat while achieving many of the effects of fasting
P06:	I disagree that FMDs are rich in complex carbohydrates. In most FMD regimens, the absolute and relative carbohydrate content is very low (Vernieri C et al. Cancer Discov 2022; de Groot S et al. Nat Commun 2020; Bauersfeld SP et al. BMC Cancer 2018)
P11:	It needs to be emphasized that fasting mimicking isn't comparable to plain fasting because of the much higher caloric intake, plus there is no complete shut-down of the activity of the digestive system

P23:	I agree with the definition above (except that I would omit the words 'is followed periodically' and 'usually'. FMD differs from calorie restriction as it mimics water only fasting better and it obviously differs from water only fasting because calories are consumed.
**No, FMD should not be included**	
P33:	This is a name which has been used for a commercial product as stated. Also it is a low calorie / fasting diet. There may be other versions of fasting mimicking diets in future with different macronutrient compositions. Lots of reasons to leave out

FMD

How many calories should be consumed as part of the **Fasting-Mimicking Diet**:

Answer	n	%
a maximum of 600 kcal per day (AO01)	11	33.33%
600-1000 kcal per day (AO02)	11	33.33%
Due to my specialization, I am not familiar with this term / this method and prefer not to vote on it (AO03)	0	0.00%
Didn't vote, as they didn't want to include FMD	11	33.33%

**A maximum of 600 kcal per day – Comments**	
P06:	In our experience, to be effective in reducing blood glucose, insulin and IGF-1 concentration within 3-5 days, a FMD regimen should contain less than 600 Kcal per day and should be very poor in carbohydrate and protein content
**600-1000 kcal per day – Comments**	
P36:	there should be no limit as a diet could be fasting mimicking even in the absence of CR, although in the great majority of cases it will need to be calorie restricted
P11:	This is actually modified fasting …
P01:	i would give a range of 500-1000 kcal

FMD

Should a **Fasting-Mimicking Diet** per definition include (you can choose more than one answer):

Answer	n	%	% w/o SQ004 & 05
a low carbohydrate intake (SQ001)	7	21.21%	18%
a low protein intake (SQ002)	14	42.42%	36%
a low intake of fatty acids (SQ003)	5	15.15%	13%
only a restricted intake of refined sugars and calories (SQ004)	13	39.39%	33%
Due to my specialization, I am not familiar with this term / this method and prefer not to vote on it (SQ005)	1	3.03%	
Not voted on because they don't want to include FMD in the Delphi (SQ006)	11	33.33%	

**A low protein intake**	
P36:	this is a complex question and answer. Again the type of carbs, or proteins or fat or CR level matter. But yes a minimum could be set up. For example 10 grams of legume proteins could have FMD effects equivalent to those of 3 grams of red meat proteins (mehr Methhionin)
**A low protein and a low carbohydrate intake**	
P06:	In my opinion, *a fasting-mimicking diet regimen should be defined as a low-calorie (max 600 Kcal/day), low-carbohydrate (max 200 Kcal/day), low-protein (max 100 Kcal/day) diet that lasts for 3-5 days and that is repeated cyclically*
**Low protein, low intake of fatty acids and low carbohydrate intake**	
P34:	plant based
**Low protein & only a restricted intake of refined sugars and calories**	
P23:	It is as defined above: it should virtually lack refined sugar and starch, have low protein and therefore primarily comprise complex carbohydrate and (unsaturated) fatty acid.

GRUEL FASTING (traditional german fasting regimen)

Gruel fasting refers to a fasting regimen in which 400 ml of oat or rice gruel is given in three portions per day. Water and unsweetened herbal tea may be consumed *ad libitum*. Gruel fasting provides about 200 kcal and 45 g of carbohydrates per day.

Answer	n	%	% w/o AO07
Strongly Agree (AO01)	12	36.36%	46,15%
Agree (AO02)	1	3.03%	3,85%
Neutral (AO03)	0	0.00%	0,00%
Disagree (AO04)	1	3.03%	3,85%
Strongly Disagree (AO05)	1	3.03%	3,85%
This definition is irrelevant and should be excluded from the consensus process (AO06)	11	33.33%	42,31%

	Due to my specialization, I am not familiar with this term / this method and prefer not to vote on it (AO07)	7	21.21%	
	Total w/o AO07	26		100,00%

AGREE: Suggested modifications / alternative definition

P17: most might use more volume say 3x200 = 600 mL

DISAGREE: Suggested modifications / alternative definition

P01: Isn´t the caloric intake higher ?

STRONGLY DISAGREE: Suggested modifications / alternative definition

P36: I think for these to be taken seriously we need to only include regimens that have strong scientific support and publications. I have never heard of a gruel fasting

INTERMITTENT DRY FASTING /DIURNAL DRY FASTING

Answers to comments from the first round of the survey:

What religion proscribes this regimen?

- Islam
- Bahá'í religion

Are there data that dry fasting is beneficial? I could hardly imagine because you need renal clearance during fasting.

For more information on the benefits of **dry fasting**, we have listed a few studies below. To access the content of the studies, please open the links in a new tab or window:

Alkandari et al., 2012: The implications of Ramadan fasting for human health and well-being
Link: https://pubmed.ncbi.nlm.nih.gov/22742901/
Sarro et al., 2020: The efficacy of fasting regimens on health outcomes: a systematic overview
Link: https://pubmed.ncbi.nlm.nih.gov/32914941/
Liebscher et al., 2021: Effects of Daytime Dry Fasting on Hydration, Glucose Metabolism and Circadian Phase: A Prospective Exploratory Cohort Study in Bahá'í Volunteers
Link: https://www.ncbi.nlm.nih.gov/pmc/articles/PMC8358295/

We have decided to offer two different terms with slightly different definitions for what we called intermittent dry fasting in the last round, according to the feedback we received. Please select one of the following terms with their definition, "neither of the above", if you disagree with the given definitions, or "I am not familiar with this term / this method":

- **A1: Intermittent dry fasting (IDF)** refers to intermittent fasting regimens that involve abstaining from food and fluid intake during fasting hours. As daily fasting intervals generally depend on daylight hours, they may range from 9 to 20 hours. Diurnal intermittent dry fasting, as practiced in religious contexts, does not require any defined energy restriction during the nocturnal eating window.
- **A2: Diurnal dry fasting**, as practiced in religious contexts, refers to a dietary regimen that involves abstaining from food and fluid intake during daylight hours for a certain period every year. The daily fasting window may range from 9 to 20 hours. There are no requirements on energy restriction during the nocturnal eating window.

Answer	n	%	% w/o AO04
A1: Intermittent dry fasting (IDF) (AO01)	14	42.42%	50,00%
A2: Diurnal dry fasting (AO02)	12	36.36%	42,86%
neither of the above (I disagree with both definitions) (AO03)	2	6.06%	7,14%
Due to my specialization, I am not familiar with this term / this method and prefer not to vote on it (AO04)	5	15.15%	
Total w/o A007	28		100,00%

Diurnal Dry Fasting

P20: Just call it Ramadan fasting?

P23: I actually think both definitions are valuable. They essentially specify daily or intermittent fasting regimens to include abstinence of water intake into the intervention.

Neither of the above (I disagree with both definitions)

P05: I would prefer a more simple and generalizable definition: "*Intermittent dry fasting (IDF) refers to intermittent fasting regimens that involve abstaining from food and fluid intake during fasting hours. Most commonly, they range from 9 to 20 hours.*"

As the former definiotoins didn't receive conseunss we are suggesting the definition of one of the experts

Definitions that received ≥ 70% agreement – with slight modifications:

In this second section of the questionnaire, we contrast the definitions that have already received an agreement of ≥ 70% in their current form with the definitions that we have slightly modified based on your comments. Please indicate which you prefer.

Terms concerning dietary and caloric restriction

Please select either the original wording, the modified definition, "neither of the above", if you disagree with the given definitions, or "I am not familiar with this term / this method":

DIETARY RESTRICTION (DR)

A1: (DR) comprises of chronic or intermittent restrictions in caloric intake and / or specific macronutrients and / or restraints of food intake within a specified time frame, without malnutrition. DR thus includes: all types of caloric restriction; fasting regimens such as short-term, long-term, and periodic fasting, intermittent fasting, time-restricted eating or feeding, water-only fasting, therapeutic fasting and fasting-mimicking diets; ketogenic diets; and diets with restrictions of specific macronutrients namely proteins, carbohydrates or fats.

A2: Dietary restriction (DR) comprises of continuous **~~chronic~~ or intermittent** restrictions in caloric intake and/or specific macronutrients and/or restraints of food or food and fluid intake within a specified time frame~~, without malnutrition~~. DR thus includes: all types of caloric restriction; fasting regimens such as short-term, long-term, and periodic fasting, intermittent fasting, time-restricted eating, water- and fluid-only fasting, therapeutic fasting and fasting-mimicking diets; ~~ketogenic diets;~~ and diets with restrictions of specific macronutrients namely proteins, carbohydrates, or fats.

Answer	n	%	% w/o AO04
I agree with A1: original wording (AO01)	5	15.62%	16,13%
I agree with A2: modified definition (AO02)	25	75.00%	77,42%
neither of the above (I disagree with both definitions) (AO03)	2	6.25%	6,45%
Due to my specialization, I am not familiar with this term / this method and prefer not to vote on it (AO04)	1	3.12%	
Total w/o A004	32		100,00%

I agree with A2: modified definition

P19: I agree with A2, but it needs to specify without malnutrition as this is a KEY component of DR.

neither of the above (I disagree with both definitions)

P20: I use DR interchangeably with CR – ie when I cant use 'calorie' as not appropriate as writing for a journal that I allow 'calorie' – when it should be energy (kilojoule) restriction. This is half the world. Or with macronutrient restrictions – never for fasting.

General terms concerning fasting

FASTING

A1: Fasting refers to a voluntary abstinence from some or all foods and / or beverages for therapeutic, spiritual, or political reasons.

A2: Fasting refers to a voluntary abstinence from some or all foods ~~and /~~ or foods and beverages for preventive, therapeutic, ~~spiritual~~ religious, cultural, or ~~political~~ other reasons.

Answer	n	%
I agree with A1: original wording (AO01)	2	6.06%
I agree with A2: modified definition (AO02)	28	84.85%
neither of the above (I disagree with both definitions) (AO03)	3	9.09%
Due to my specialization, I am not familiar with this term / this method and prefer not to vote on it (AO04)	0	0.00%

Neither of the above (I disagree with both definitions)

P16: A3*: Fasting refers to voluntary abstinence from some or all foods and / or beverages for preventive, therapeutic, religious, or political other reasons.*

P05: I have changed my mind. I prefer the definition: "*Fasting refers to a voluntary abstinence **from all foods and calorie-containing beverages**.*"

P23: I think fasting should refer to abstinence from **all (not some) foods or foods and caloric beverages** for preventive, therapeutic, religious or other reasons

Continuous Fasting Regimens

PERIODIC FASTING (PF)

A1: Periodic fasting (PF) refers to any fasting regimen that is repeated at regular intervals (periods), such as every day, every week, or every several months.
(According to this definition, periodic fasting would include intermittent fasting regimens.)

A2: Periodic fasting (PF) refers to any fasting regimen **lasting** ≥ 48 hours that is repeated at regular intervals (periods), such as ~~every day,~~ every **several** week**s** or ~~every several~~ months.

Answer	n	%
**I agree with A1: original wording (AO01)**	**17**	**51.52%**
**I agree with A2: modified definition (AO02)**	**14**	**42.42%**
**neither of the above (I disagree with both definitions) (AO03)**	**2**	**6.06%**
**Due to my specialization, I am not familiar with this term / this method and prefer not to vote on it (AO04)**	**0**	**0.00%**

I agree with A2: modified definition

P05: If we go with definition A2, then we need a term for intermittent fasting approaches such as fasting one day per week.

Neither of the above (I disagree with both definitions)

P15: *Periodic fasting (PF) refers to any fasting regimen that is repeated at regular intervals (periods), such as every several weeks or months.*

P36: we are confusing intermittent fasting with periodic fasting. Periodic fasting should refer to a fasting which **is repeated less than once every 2-4 weeks** and which **could be at regular intervals or not**. For example **someone could do periodic fasting once every year or every 3 months** or **even month** or do it **every month but then change to once in a while when needed**

Intermittent Fasting Regimens

INTERMITTENT FASTING (IF)

A1: Intermittent fasting (IF) refers to repetitive fasting periods of up to 48 hours. IF includes fasting regimens of 1 day per week (6:1), 2 seperate or consecutive days per week (5:2), alternate-day fasting (ADF) and time-restricted eating (TRE).

A2: Intermittent fasting (IF) refers to repetitive fasting periods **lasting** up to 48 h **each**. IF includes fasting regimens of 1 **fasting** day per week ~~(6:1),~~ 2 separate or consecutive **fasting** days per week ~~(5:2)~~ **and** alternate day fasting (ADF).~~and time-restricted eating (TRE).~~

Answer	n	%	% w/o AO04
**I agree with A1: original wording (AO01)**	**13**	**39.39%**	**40,63%**
**I agree with A2: modified definition (AO02)**	**16**	**48.48%**	**50,00%**
**neither of the above (I disagree with both definitions) (AO03)**	**3**	**9.09%**	**9,38%**
**Due to my specialization, I am not familiar with this term / this method and prefer not to vote on it (AO04)**	**1**	**3.03%**	
**Total w/o A004**	**32**		**100,00%**

I agree with A2: modified definition

P25: TRE has a different mechanism of action (restoring circadian rhythms) than IF (body uses ketones for energy) and should therefore not be lumped together.

P33: time restricted eating should have its own definition

Neither of the above (I disagree with both definitions)

P15: *Intermittent fasting (IF) refers to repetitive fasting periods lasting up to 48 h each. IF includes fasting regimens of 1 fasting day per week, 2 separate or consecutive fasting days per week, alternate day fasting (ADF), and time-restricted eating (TRE).*
TRE is a form of intermittent fasting because the fasting in TRE occurs intermittently (the intermittent occurrence is simply on a daily basis rather than a weekly or monthly basis).

P05: I strongly believe that TRE should count as intermittent fasting.
My first choice for wording would be a combination of A1 and A2: *" Intermittent fasting (IF) refers to repetitive fasting periods lasting up to 48 h each. IF includes fasting regimens of 1 fasting day per week (~~6:1 diet~~), 2 separate or consecutive fasting days per week (~~5:2 diet~~), alternate day fasting (ADF), and time-restricted eating (TRE)"*

P28: -IF refers to repetitive periods of fasting (without indicating the duration)
- fasting day: does it mean 24 hours of fasting.

For example: Ramadan fasting is an intermittent fasting and fasting lasts up to 16 hours /24 (diurnal fasting) and is repeated every day during one month (Ramadan). Another type of fasting in Islam is diurnal fasting during two days a week (Mondays and Thursdays) during the whole year

ALTERNATE-DAY FASTING (ADF)

A1: Alternate-day fasting (ADF) or in animals, **every-other-day feeding (EOD),** refers to alternating a day of eating *ad libitum* and a day of either water-only fasting or a diet very low in calories.

A2: Alternate day fasting (ADF) ~~or in animals, **every-other-day feeding (EOD),**~~ refers to alternating a day of eating *ad libitum* and a day of ~~either~~ water-only fasting ~~or a diet very low in calories~~.

Answer	n	%	% w/o AO04
I agree with A1: original wording (AO01)	13	39.39%	41,94%
I agree with A2: modified definition (AO02)	17	51.52%	54,84%
neither of the above (I disagree with both definitions) (AO03)	1	3.03%	3,23%
Due to my specialization, I am not familiar with this term / this method and prefer not to vote on it (AO04)	2	6.06%	
Total w/o A004	31		100,00%

I agree with A2: modified definition

P20: Could specify low carb calories ie not activating insulin signalling pathways if you ate

Neither of the above (I disagree with both definitions)

P33: *Alternate-day fasting (ADF) refers to alternating a day of either water-only fasting or a diet very low in calories and a day of eating ad libitum or a modified diet i.e a healthy Mediteranean diet*

TIME RESTRICTED EATING (TRE)

A1: Time-restricted eating (TRE) or, when referring to animals, **time-restricted feeding (TRF)**, is a dietary regimen in which food intake is restricted to a specific period of time (usually 4 to 10 hours) during the day, resulting in a daily fasting window of 14 to 20 hours. There is no explicit limit on energy intake during eating or feeding hours.

A2: Time-restricted eating (TRE) ~~or, when referring to animals, **time-restricted feeding (TRF)**,~~ is a dietary regimen in which food intake and the consumption of caloric beverages is restricted to a specific period of time (usually 4 to 10 hours) during the day, resulting in a daily fasting window of 14 to 20 hours. There is no explicit limit on energy intake during eating ~~or feeding~~ hours.

A3: Time-restricted eating (TRE) ~~or, when referring to animals, **time-restricted feeding (TRF)**,~~ is a dietary regimen in which food intake and the consumption of caloric beverages is restricted to a specific period of time (usually 1 to 12 hours) during the day, resulting in a daily fasting window of 12 to 23 hours. There is no explicit limit on energy intake during eating ~~or feeding~~ hours.

Answer	n	%	% w/o AO05
I agree with A1: original wording (AO01)	6	18.18%	18,75%
I agree with A2: modified definition (AO02)	19	57.58%	59,38%
I agree with A3: modified definition (AO03)	5	15.15%	15,63%
neither of the above (I disagree with the given definitions) (AO04)	2	6.06%	6,25%
Due to my specialization, I am not familiar with this term / this method and prefer not to vote on it (AO05)	1	3.03%	
Total w/o A005	32		100,00%

I agree with A2: modified definition

P05: I suggest a small modification to definition A2: "*Time-restricted eating (TRE) is a dietary regimen in which food intake and the consumption of caloric beverages is restricted to a specific period of time (usually 1-10 hours) during the day, resulting in a daily fasting window of at least 14 hours. There is no explicit limit on energy intake during eating hours*."

I agree with A3: modified definition

P33: although can also have overall cal restricted TRF diets

Neither of the above (I disagree with the given definitions)

P36: I agree with A3 but the animal variation has to be included

P25: the literature supports definition 3 **BUT range of eating 6-12 hours**; there is no benefit to restricting less than 6 hours (reference available upon request:
"Time-restricted Eating for the Prevention and Management of Metabolic Diseases" 2022 By Panda

Specific Fasting Regimens

THERAPEUTIC / MEDICAL FASTING

A1: Therapeutic fasting, also called **medical fasting**, refers to any fasting regimen that is applied as a therapeutic intervention by a trained physician.

A2: Therapeutic fasting refers to any fasting regimen that is applied as a therapeutic intervention. **Medically supervised fasting** refers to any fasting regimen that is applied as a therapeutic intervention by a trained physician or similar credentialed healthcare provider.

Answer	n	%	% w/o AO04
I agree with A1: original wording (AO01)	10	30.30%	32,26%
I agree with A2: modified definition (AO02)	21	63.64%	67,74%
neither of the above (I disagree with both definitions) (AO03)	0	0.00%	0,00%
Due to my specialization, I am not familiar with this term / this method and prefer not to vote on it (AO04)	2	6.06%	
Total w/o A004	31		100,00%

RELIGIOUS FASTING

A1: A1: Religious fasting refers to any fasting regimen that is undertaken as part of a religious practice. Religious fasting thus involves practices such as: dry fasting at certain intervals over 24 hours (e.g. Jewish traditions, The Church of Jesus Christ of Latter-day Saints); intermittent dry fasting* (e.g. Ramadan fasting, Bahá'í fasting); time-restricted eating (e.g. Buddhism); and diets restricting certain foods (e.g. Christian orthodox traditions, Daniel fast) if more broadly defined.

A2: Religious fasting refers to any fasting regimen that is undertaken as part of a religious practice. Religious fasting thus involves practices such as: dry fasting* ~~at certain intervals over~~ **on specific days of the year up to** 25 hours at a time (e.g. Jewish tradition, The Church of Jesus Christ of Latter-day Saints); intermittent dry fasting (e.g. Ramadan fasting, Bahá'í fasting); time-restricted eating (e.g. Buddhism); and diets restricting certain foods (e.g. Christian orthodox traditions, Daniel fast) if more broadly defined. **Typically, religious fasting also includes spiritual activities to improve cognitive function and well-being.**

(*Please note that the term "intermittent dry fasting" may need to be replaced by "diurnal dry fasting" in this definition depending on the votes for intermittent / diurnal dry fasting).

Answer	n	%	% w/o AO04
I agree with A1: original wording (AO01)	12	36.36%	38,71%
I agree with A2: modified definition (AO02)	16	48.48%	51,61%
neither of the above (I disagree with both definitions) (AO03)	3	9.09%	9,68%
Due to my specialization, I am not familiar with this term / this method and prefer not to vote on it (AO04)	2	6.06%	
Total w/o A004	31		100,00%

I agree with A2: modified definition

P11: In Germany it is very common to use the term: Intervall-fasting referring to the method of 16/8 time-restricted eating

Neither of the above (I disagree with both definitions)

P25: Jewish tradition is TOTAL fasting not dry fasting; otherwise A2 OK

P28: - Ramadan fasting (in Islam) is a dry fasting repeated at 24 hours intervals (diurnal fasting) during the month of Ramadan (lunar month) – another type of fasting in Islam is Mondays and Thursdays diurnal fasting.

P33: *Religious fasting refers to any fasting regimen that is undertaken as part of a religious practice. Religious fasting thus involves practices such as:* ***dry fasting at certain intervals ~~over~~ on specific days of the year up to 25 hours at a time*** *(e.g. Jewish tradition, The Church of Jesus Christ of Latter-day Saints); intermittent dry fasting* (e.g. Ramadan fasting, Bahá'í fasting); time-restricted eating (e.g. Buddhism); and diets restricting certain foods (e.g. Christian orthodox traditions, Daniel fast) if more broadly defined.* (combination of A1 and A2)

Third Questionnaire

Defining Fasting: Finding Common Ground Using the Delphi Method - Round 3

Please read the following information thoroughly before starting the third questionnaire.

In evaluating the results of the second round, we found that opinions on the nomenclature of the terms "**fasting**" and "**modified fasting**" vary widely around the world. Therefore, it was decided to exclude the term "fasting" and all terms that could possibly be classified under "modified fasting" (e.g. therapeutic fasting, alternate-day modified fasting) in this third round. The discussion and decision on these terms will be a central aspect of the **upcoming live online discussion** (see more information on the next page). Furthermore, we would like to inform you that it was necessary to **expand the evaluation rules** again (see below).

As a reminder:

- To reduce complexity, we have limited the selection to fasting definitions in humans and removed all terms related to animals.
- We modified the fasting terms and definitions according to your suggestions. In the definitions you will see crossed out words in red and newly added words underlined in blue to make the modifications visible. We also provide you with single or summarized comments of the panel experts in anonymized form, as well as the distrubtion of votes from the last round, to help you decide. However, in order to make this survey as compact as possible, not all comments are listed. (You will r**eceive a document with all comments on request**).
- In the final Delphi round, we will ask you to indicate whether you are generating income in any way from one or more of the fasting methods defined in this process, so that we can disclose this information in the subsequent publications, if appropriate.

Evaluation rules (new rules are marked in green: 5-10)

1. A definition will be **accepted** in case of **agreement of ≥ 70%** of participants ("strongly agree" or "agree").
2. A definition will be **removed** from the list, if **≥ 50%** of participants choose "**This definition is irrelevant and should be excluded from the consensus process**".
3. Suggested changes or new terms to be defined will be taken into consideration for the next survey round when they have been suggested by at least 2 participants.
4. In case you feel incapable of voting on one or more terms / methods because they don't fall within your area of expertise, please choose the answer option: "*Due to my specialization, I am not familiar with this term / this method and prefer not to vote on it*". ►We will only count the votes for the answers "**strongly agree - agree - neutral - disagree - strongly disagree - this definition is irrelevant and should be excluded from this consensus process**".
5. Consensus was reached on several definitions in the second Delphi round (according to the 1st evaluation rule), but often ≥ 2 people proposed the same changes for these definitions which is why the 3rd evaluation rule comes into play. We therefore sent **private emails to the commenting participants** to clarify any questions of understanding and to clarify the importance of the change to the individual. If the modifications were still relevant to ≥ 2 experts after the email exchange, a definition with the desired modifications was created and offered for re-evaluation in this questionnaire round together with the initial definition.
6. For definitions from which neither reached-consensus (≥ 70% of participants strongly agree + agree), nor exclusion from the process (≥ 50% of participants choose “this definition is irrelevant”) emerged and for which the 3rd evaluation rule was irrelevant, we decided to select individual expert comments and adopt them as modifications to the original definition. These modified definitions will now be presented for re-evaluation.
7. Whenever two definitions had been proposed for the same term, but no clear leading definition emerged, we tried to take into account all experts’ comments and propose an appropriately modified definition for re-evaluation in this round.
8. For definitions for which no consensus seemed to be foreseeable in the next round and / or for which the experts did not make any target suggestions for change, we proposed our own changes to the definitions which will be presented for evaluation in this round.
9. **Minor additions to definitions for which no consensus seemed foreseeable** in the next round (e.g. “what type of water should be consumed in water-only fasting?”) will **not** be **included** in the final definition of the term, but will be mentioned in the explanations of the term in the subsequent publication.
10. Terms that have so far been defined separately but which, according to the experts, can be **subsumed under one definition**, are grouped together (e.g. “continuous energy restriction / daily energy restriction” and “caloric restriction”).

Information regarding the live-online workshop

After this survey round, a **live online discussion** will take place to discuss all terms on which no consensus could be reached up to that point. As the time will be limited (planned duration: 3 hours), we ask you to send us the terms and arguments that are particularly important to you to in advance via e-mail, or alternatively, submit them on the last page of this questionnaire. We will then structure the discussion according to your input and inform you about the planned procedure shortly before the date.

Disclaimer: Please note that anonymity cannot be maintained during the live online discussion.

Guide for the colors & italics in the comments

- Comments that occur more than once are marked in: orange/light blue/purple
- Red background: no consensus
- Terms highlighted in green: reached an agreement ≥ 70%
- *Written in italics:* Alternative proposed

Additions to definitions / definitions that will not be further evaluated

This page is for your information only, so that you understand why we have made certain changes to the definitions.

<table>
<tr><td>CONTINUOUS ENERGY RESTRICITION</td><td>CONTINUOUS ENERGY RESTRICTION – In the last round we asked you to vote on the following definition:

Continuous Energy Restriction (CER), also called Daily Energy Restriction (DER), refers to a daily caloric restriction of about 25-30% of the daily amount of calories required for weight maintenance.

No consensus (agreement votes: 64.52%) was reached, but a few experts commented that CER / DER should be defined together with caloric restriction. For this reason, no additional definition for CER /DER will be included in the publication.</td></tr>
<tr><td>TOTAL FAST</td><td>TOTAL FAST / COMPLETE FAST – definition:

The term total fast, or complete fast, refers to a fasting regimen, where only calorie-free beverages, including water and unsweetened tea, are consumed ad libitum for a certain period of time. Historically, prolonged total fasts were used for the therapy of people with obesity under the term zero-calorie diet.

Although the above definition reached a consensus of 85.85 %, a few dry fasting experts mentioned that the terms better fit the definition of "dry fasting". It was therefore decided to delete the above definition and list the terms "total fasting / complete fasting" under "dry fasting".

Expert comment: In total fast, food and beverages should not be consumed such as during Ramadan fasting.</td></tr>
<tr><td>WATER-ONLY FASTING</td><td>Kind of water in WATER-ONLY FASTING – distribution of votes:
<table><tr><th>Kind of water</th><th>Votes in %</th></tr><tr><td>Distilled water</td><td>3.33</td></tr><tr><td>Mineralized water / tap water</td><td>30.00</td></tr><tr><td>There should be no specific requirements for the consumed water</td><td>66.67</td></tr></table>
No consensus could be reached on the preferred kind of water to be consumed in water-only fasting. Therefore, no recommendations are made in the definition. The results and comments on this question will be described in the publication.

Expert comments (Round 1 & 2):

Distilled water:
There are advantages to supplemented fasting in that known rate limited micronutrients can be used as markers for depletion. Potassium and sodium are examples. In supplemented fasting these known and easily monitored nutrients are sensitive and reliable markers of depletion. If you supplement these, you may experience unrecognized depletion of other less sensitive and unmonitored nutrients.</td></tr>
</table>

	• Although any highly purified water might do, steam distillation is the most effective means of purification.<br><br>**Mineralized water / tap water**:<br><br>• Distilled water can be dangerous. Minerals and electrolytes are necessary.<br>• Tap water could also be fine. It depends on the source of tap water.<br><br>**There should be no specific requirements for the consumed water**:<br><br>• Unfamiliar if there are studies that show that distilled vs mineralized water is better. There might be also socio-cultural factors for water availability/choice<br>• The valid points about potential differences in the source of tap water, availability/choice of water, etc. further supports the idea that there should be no specific requirement for the water source (in my opinion).<br>• these can be specified in anyone's particular practice but only the practices that are widely used should have consensus naming

FLUID-ONLY FASTING

FLUID-ONLY FASTING – To complete the above definition of fluid-only fasting, we asked you whether you would recommend the use of **bowel / colon cleansing** during this fasting regimen.

Distribution of votes**:**

Bowel / colon cleansing	Votes in %
No preference	19.23
No, I do not recommend bowel / colon cleansing	69.23
Yes, with sodium sulfate (Glauber's salt) or magnesium sulfate (Epsom salt, bitter salts)	7.69
Yes, with colonic irrigation or enema	3.85

Expert comments in Round 2:

Yes:

- I am missing the option: combination of methods. I usually have my participants choose among different methods like laxative salts, enema and/or colonic irrigation. Endoscopy solutions may be used as well, in case patients tolerate the large amount of fluids. Prior to colonoscopy the application of colon cleansing methods are crucial, so why do we question this for fasting (Keyword: Auto-Intoxication….probably no scientific proof..)
- I think, bowel / colon cleansing could help built up a healthy microbiota. In this case, duration of fasting should be at least 7 days
- I think this should be practiced during a fasting period of at least 7 days.

No:

- To me, it would be much better to acknowledge that some individual practitioners may recommend these procedures to their patients – or that some individual patients may request this from their practitioner – rather than to recommend these procedures for everyone undergoing fluid-only fasting.
- Bowel cleansing, preferably with Epsom salt, is necessary in very rare cases.
- We should be very careful about recommending colonic irrigations, as they have the potential for serious risks.
- Proper dietary preparation will eliminate the need for bowel stimulation in over 99% of patients undergoing fasting. The use of bowel stimulation during fasting is contraindicated.

No preference:

- This does not sound evidence-based and can't see any potential benefit. Should only be included in the terminology if there is some evidence to support the rational and benefits
- I would use / recommend bowel cleansing in case of previous good experience with it, in case of obstipation and in some cases of irritable bowl syndrome. Otherwise I would leave the decision to the patient after information about the pro´s and cons.

No consensus could be reached on this issue. Therefore, no recommendations regarding bowel cleansing will be included in this definition. The results and comments on this question will be described in the publication.

ONE MEAL A DAY DIET (OMAD)

ONE MEAL A DAY DIET – (a version of time-restricted eating) We asked you if you thought OMAD should be defined separately as part of this consensus process?

Distribution of votes:

Answer	Votes in %
Yes	42.42
No	45.45
No preference	12.12

	No consensus was reached, hence the editors decided to not create a separate definition for this term, but to include the "**one meal a day diet**" in the definition of "**time-restricted eating**" (see option: A1 for time-restricted feeding).

Terms on which consensus has been reached but which need to be re-evaluated due to the evaluation rules

Terms concerning dietary and caloric restriction

CALORIC RESTRICTION (CR)

CALORIC RESTRICTION (CR) – previous definition:

Caloric restriction (CR) describes a reduction in energy intake below the total amount of calories that would be needed to maintain a person's current body weight, without causing malnutrition. CR may also be used to achieve a healthy body weight over time.

This definition reached a consensus of **87.88 %.** Regarding the range of reduction in daily caloric intake no consensus was reached:

Range in %	Votes in %
10-25	15.15
10-40	27.27
15-40	12.12
10-50	3.03
15-40	3.03
20-40	3.03
20-50	6.06
The range can't be generalized	30.30
other	6.06

Although consensus was reached for the above definition, several changes were proposed and since consensus could not be reached on the range of reduction, the editors decided to adapt the above definition and offer two more options for the definition of CR according to the comments and the 7th evaluation rule.

In these newly proposed definitions, the term "**continuous energy restriction**" (CER) / "**daily energy restriction**" (DER) is subsumed under the term "**caloric restriction**", as the following comments on the definition of CER /DER were received:

Expert comments on CER /DER:

- To me, the percentage of energy restriction included in the definition of CER should match the range agreed upon for the definition of caloric restriction.I feel like this makes sense because CER/DER is essentially caloric restriction employed each day.
- because this [CER] defines the daily use of CR, I would suggest that the % reduction should be adjusted to the % range of reduction in daily caloric intake that has the majority vote
- we should just have 1 term (CR), adding more terms to say the same is only going to add to confusion

Please choose the definition of **caloric restriction** that you agree with the most:

Note on all three definitions: Total caloric intake here refers to the amount of calories required to maintain a person's "healthy" BMI – depending on their height and age.

A1: Caloric restriction (CR) describes a reduction* in energy intake below the total amount of calories that would be needed to maintain a person's current body weight, without causing malnutrition. ~~CR may also be used to achieve a healthy body weight over time.~~ If **CR** is done daily, it can also be referred to as **continuous energy restriction (CER)** or **daily energy restriction (DER).**
*Note: amount of reduction is not specified in this definition.

Experts' comments as to why the amount of reduction should not be stated:

- on CR: The percentage is a function of the reason for losing weight and the speed with which one wants to lose [weight]. Again, it is not part of the definition of CR. Arguably, a 1% reduction is also CR, albeit a very slow way to lose weight.
- On CER: I don't think that continuous energy restriction necessarily has a certain amount of energy restriction assigned to it. It just describes that it is occurring at every meal across the day.

- On CER: I think the term should not include a percentage reduction of calories. Any reduction of calories (below the requirement for maintenance of bodyweight) on a daily basis can be defined as continuous or daily energy restriction.

A2: Caloric restriction (CR) describes a ≥ 10%* reduction in energy intake below the total amount of calories that would be needed to maintain a person's current body weight, without causing malnutrition. ~~CR may also be used to achieve a healthy body weight over time.~~ If **CR** is done daily, it can also be referred to as **continuous energy restriction (CER)** or **daily energy restriction (DER).**
*Note: the editors have chosen "≥ 10%" to combine the range options previously offered.

A3: Caloric restriction (CR) describes a reduction in energy intake below the total amount of calories that would be needed to maintain a person's current body weight, without causing malnutrition. The degree of caloric restriction should be decided individually depending on the person's age, sex, current body weight, occupation, goal & planned duration of CR. If **CR** is done daily, it can also be referred to as **continuous energy restriction (CER)** or **daily energy restriction (DER).**

Answer	n	%
I agree with A1 (amount of reduction is not specified in this definition) (AO01)	10	33.33%
I agree with A2 (reduction in energy intake ≥ 10%) (AO02)	4	13.33%
I agree with A3 (The degree of caloric restriction is to be decided individually) (AO03)	16	53.33%
This definition is irrelevant and should be excluded from the consensus process (AO06)	0	0.00%
Due to my specialisation, I am not familiar with this term / this method and prefer not to vote on it (AO07)	0	0.00%

I agree with A1 - comments

P13: To me, the additional sentence of explanation provided in A3 ("The degree of caloric restriction should be decided individually depending on the person's age, sex, current body weight, occupation, goal & planned duration of CR.") could be included in the publication as additional explanation regarding the concept of caloric restriction but is not necessary to include in the initial definition itself. It seems to make the definition longer than necessary.

P33:

- Need to state that energy restriction is estimated according to their requirements for the day as this aligns with energy to maintain body weight. Time restricted feeding does not fit with this.
- No need to state how energy requirements are estimated. The first statement saying below energy requirements would by definition use information on age, gender etc but no need to state. Not sure why need to say anything about daily calorie restriction

I agree with A3 - comments

P25: instead of occupation I would put "degree of daily physical activity"

P10: A data-driven definition is more appropriate

DRY FASTING (DF)

DRY FASTING – definition:

Dry fasting refers to a fasting regimen, during which a voluntary abstinence from all foods and beverages, including water, is practiced for a certain period of time.

The above definition reached a consensus of **87.50 %** as is. However, some dry fasting experts have pointed out that the terms "**total /complete fasting**" is equivalent to "**dry fasting**". The editors therefore propose to include the terms " total /complete fasting" in the definition of dry fasting as follows:

Dry fasting, also called **total fasting / complete fasting**, refers to a fasting regimen, during which a voluntary abstinence from all foods and beverages, including water, is practiced for a certain period of time.

Answer	n	%	% w/o A007
Strongly Agree (AO01)	13	43.33%	48,15%
Agree (AO02)	7	23.33%	25,93%
Neutral (A003)	0	0.00%	0,00%
Disagree (AO04)	4	13.33%	14,81%
Strongly Disagree (AO05)	3	10.00%	11,11%
This definition is irrelevant and should be excluded from the consensus process (AO06)	0	0.00%	0,00%
Due to my specialization, I am not familiar with this term / this method and prefer not to vote on it (AO07)	3	10.00%	

DISAGREE: suggested modifications / alternative definition

P07: **Dry food in the Orthodox Church fasting**, today, means that **plant foods are allowed, but fresh and not cooked**. **Drinking water is allowed**. It usually lasts one day, sometimes up to three days.

P22:	I do mir lieb the addendum in blue, because complete or total fasting to me seems absence of food and drinks, but not from water
P33:	think useful to define whether total food (calorie) fast or food and fluid fast
P01:	Commonly, in these times, The term total is used for zero diet (but with water or tea intake) If we choose the above proposed definition a lot of published studies would be misinterpreted
**STRONGLY DISAGREE: suggested modifications / alternative definition**	
P35:	Dry fasting should not be referred to as total/complete fasting as that will create confusion on the well-established practice of referring to water-only fasting as complete or total fasting.
P06:	I strongly disagree with considering "dry fasting" as equivalent to "total/complete fasting".<br>Indeed, while these types of fasting could be similar in terms of calorie intake restriction, there are huge physiological and therapeutic differences between these two approaches. In particular, while "total/complete fasting" can be safe, well tolerated and, potentially, therapeutically effective even when prolonged (i.e., when lasting for 2 or more days), "dry fasting", which also implies total abstinence from the intake of beverages, including water, is potentially harmful, in that it can cause hypotension, dehydration, damage to the kidneys, blood electrolyte imbalance and other severe side effects.
P32:	*Dry Fasting refers to a fasting regimen, during which a voluntary abstinence from all foods and beverages, including water, is practiced for a certain period of time.*

Intermittent Fasting Regimens

INTERMITTENT ENERGY RESTRICTION (IER)

INTERMITTENT ENERGY RESTRICTION – definition:

Intermittent Energy Restriction (IER) includes periods of caloric restriction alternating with periods of *ad libitum* eating. As such, IER includes fasting regimens like intermittent fasting (IF) and time-restricted eating (TRE).

This definition reached a consensus of **78.79 %**. Although consensus was reached, changes were proposed.

Expert comments:

- TRE is a form of intermittent fasting because the fasting in TRE occurs intermittently (the intermittent occurrence is simply on a daily basis rather than a weekly or monthly basis).
- I think it is better to continue to call it intermittent fasting but it needs to be defined, and maybe IER can be an additional but different term
- Time-restricted eating (TRE) is definitely not a form of intermittent energy restriction. To me, IER means eating a low-calorie diet some number of days per week or per month
- TRE needs to have a separate definition to IER.

Based on the comments received, we decided to offer two options. Please indicate which you prefer:

A1 (initial wording): **Intermittent Energy Restriction (IER)** includes periods of caloric restriction alternating with periods of *ad libitum* eating. As such, IER includes fasting regimens like intermittent fasting (IF) and time-restricted eating (TRE).

A2: Intermittent Energy Restriction (IER) includes periods of caloric restriction alternating with periods of ad libitum energy intake.

Answer	n	%
I agree with A1 (AO01)	17	56.67%
I agree with A2 (AO02)	13	43.33%
This definition is irrelevant and should be excluded from the consensus process (AO03)	0	0.00%
Due to my specialization, I am not familiar with this term / this method and prefer not to vote on it (AO04)	0	0.00%

**I agree with A2 – comments:**	
P06:	I disagree with considering IER, IF and TRE as equivalent terms.<br>**IF an a more extreme version of IER**, and in this respect it **can be incorpotared within IER.** However, **they cannot be considered as equivalent terms**, and a threshold of allowed calorie intake should be fixed to differentiate these two terms. IF and TRE can be considered as equivalent, provided that a minimum duration of fasting is specified for both approaches
P23:	I would define this along the same lines as intermittent fasting (see further)
P13:	I appreciate how straightforward A2 is, which is why it received my vote. However, this definition may leave it up to the reader **if they conceptualize intermittent fasting and time-restricted eating as programs that meet this definition (rather than explicitly stating that they do meet the definition, as in A1**). This could be fine, but I am not sure if that is consistent with the **purpose of the document**, which I think is **to reduce some of the ambiguity and confusion** regarding these terms.
P33:	think time restricted feeding should have its own definition to distinguish it from IER

P01:	none
P28:	in the term 'TRE' there is not the term 'intermittent'

Terms on which consensus has NOT been reached and need to be re-evaluated:

Intermittent Fasting Regimens

INTERMITTENT FASTING (IF)

INTERMITTENT FASTING – In the second round we asked you which of the following two definitions of intermittent fasting you prefer:

A1: Intermittent fasting (IF) refers to repetitive fasting periods of up to 48 hours. IF includes fasting regimens of 1 day per week (6:1), 2 separate or consecutive days per week (5:2), alternate-day fasting (ADF) and time-restricted eating (TRE).

A2: Intermittent fasting (IF) refers to repetitive fasting periods lasting up to 48 h each. IF includes fasting regimens of 1 fasting day per week, 2 separate or consecutive fasting days per week and alternate day fasting (ADF).

Distribution of votes:

A1: ▶**40.63 %** of the panel experts voted in favour of this definition.
A2: ▶**50.00 %** of the panel experts voted in favour of this definition.
A3 (neither of the above): ▶**9.38 %** of the panel experts voted "neither of the above".

Expert comments:

A2:

- TRE has a different mechanism of action (restoring circadian rhythms) than IF (body uses ketones for energy) and should therefore not be lumped together
- time restricted eating should have its own definition

neither of the above:

- I strongly believe that TRE should count as intermittent fasting.

As no consensus was reached, the editors propose to subsume "**time-restricted eating**" under the term "**intermittent fasting**" since TRE is found under IF in common mesh terms This is a stronger argument for the editors than the fact that TRE is slightly different from other IF regimens because of its effect on circadian rhythms rather than ketone body metabolism. Therefore, we propose a combination of definitions A1 and A2, which was suggested by one of the participants:

A3: Intermittent fasting (IF) refers to repetitive fasting periods lasting up to 48 h each. IF includes fasting regimens of 1 fasting day per week, 2 separate or consecutive fasting days per week, alternate-day fasting (ADF), and time-restricted eating (TRE).

Answer	n	%
Strongly Agree (AO01)	15	50.00%
Agree (AO02)	11	36.67%
Neutral (AO03)	0	0.00%
Disagree (AO04)	3	10.00%
Strongly Disagree (AO05)	1	3.33%
This definition is irrelevant and should be excluded from the consensus process (AO06)	0	0.00%
Due to my specialization, I am not familiar with this term / this method and prefer not to vote on it (AO07)	0	0.00%

STRONGLY AGREE: further thoughts

P05:	I disagree with implications of the statement: TRE has a different mechanism of action (restoring circadian rhythms) than IF (body uses ketones for energy) and should therefore not be lumped together. (1) TRE has multiple mechanisms, not just restoring circadian rhythms (too narrow). (2) This statement assumes that the most important feature of fasting is the production of ketones. History, IF has not been defined by the production of ketones. Historically, definitions have focused on the length of the fasting period, not the underlying mechanisms (which are still being understood).
P01:	none

DISAGREE: suggested modifications / alternative definition

P23:	*Intermittent fasting refers to repetitive fasting periods applied **on a per week basis**, alternated with periods of ad libitum food intake.*
P25:	would have preferred to keep them separate BUT if you do combine them, then at least give a brief description of TRE in the definition "(limiting food intake to 6-12 hours per 24 hour period)"

P28:	*Intermittent fasting (IF) refers to repetitive fasting periods. IF includes diurnal fasting regimens of 1 fasting day per week, 2 separate or consecutive fasting days per week,* ***successive- or*** *alternate-day fasting.*

STRONGLY DISAGREE: suggested modifications / alternative definition

P33:	we need to describe the different patterns of the diet time restricted feeding is very different to intermittent fasting and should have its own definition.

TIME RESTRICTED EATING (TRE)

TIME-RESTRICTED EATING – In the second round we asked you which of the following three definitions of time-restricted eating you prefer:

A1: Time-restricted eating (TRE) or, when referring to animals, **time-restricted feeding (TRF)**, is a dietary regimen in which food intake is restricted to a specific period of time (usually 4 to 10 hours) during the day, resulting in a daily fasting window of 14 to 20 hours. There is no explicit limit on energy intake during eating or feeding hours.

A2: Time-restricted eating (TRE) is a dietary regimen in which food intake and the consumption of caloric beverages is restricted to a specific period of time (usually 4 to 10 hours) during the day, resulting in a daily fasting window of 14 to 20 hours. There is no explicit limit on energy intake during eating hours.

A3: Time-restricted eating (TRE) is a dietary regimen in which food intake and the consumption of caloric beverages is restricted to a specific period of time (usually 1 to 12 hours) during the day, resulting in a daily fasting window of 12 to 23 hours. There is no explicit limit on energy intake during eating hours.

Distribution of votes:

A1: ▶ **18.75 %** of the panel experts voted in favour of this definition.
A2: ▶ **59.38 %** of the panel experts voted in favour of this definition.
A3: ▶ **15.63 %** of the panel experts voted in favour of this definition.
A4 (neither of the above): ▶ **6.25 %** of the panel experts voted "neither of the above".

As no clear favorite emerged, we offer a new definition (=A1) proposed by one of the experts and the definition with the majority of votes from the last round (A2). Please indicate which you prefer:

A1: Time-restricted eating (TRE) is a dietary regimen in which food intake and the consumption of caloric beverages is restricted to a specific period of time (usually 1 to 10 hours) during the day, resulting in a daily fasting window of at least 14 hours. There is no explicit limit on energy intake during eating hours.*

*Note: Low frequency meal patterns such as the "**one meal a day diet (OMAD)**" can be regarded as part of TRE according to this definition.

A2: Time-restricted eating (TRE) is a dietary regimen in which food intake and the consumption of caloric beverages is restricted to a specific period of time (usually 4 to 10 hours) during the day, resulting in a daily fasting window of 14 to 20 hours. There is no explicit limit on energy intake during eating hours.

Answer	n	%
I agree with A1: new wording (AO01)	16	53.33%
I agree with A2: initial definition (AO02)	13	43.33%
neither of the above (I disagree with the given definitions) (AO04)	1	3.33%
Due to my specialization, I am not familiar with this term / this method and prefer not to vote on it (AO05)	0	0.00%

I agree with A1: new wording – comments

P13:	As indicated by the note attached to the asterisk (for A1), inclusion of OMAD as part of TRE necessitates that the eating period could be as short as ~1 hour. I think this definition is preferable as **it is broader and encompasses virtually all TRE-style programs**, which I think **should be the goal of the definition**.
P01:	none

I agree with A2: initial definition - comments

P25:	what about the requirement to begin eating at least one hour after arising, and end at least 3 hours before going to sleep? Without that you don't have the full benefit of improving the circadian rhythm.

Neither of the above - comments

P33:	suggest TRF if > fasting period to the average i.e > 12 hours

Specific Fasting Regimens

FASTING-MIMICKING DIET (FMD)

FASTING-MIMICKING DIET (FMD) – In the last round we asked you to indicate whether you consider the inclusion of the term **Fasting-Mimicking Diet** important for this consensus process.

Distribution of votes:

Answer	Votes in %
Yes, FMD should be included.	68.75
No, FMD should not be included.	31.25

Expert comments:

Yes:

- While I agree with the comment that this term seems to be used primarily in relation to the company L-Nutra / ProLon, I think it is important to be as comprehensive as possible. Since there is a growing body of research on FMD, it seems important to include so that the publication(s) resulting from this process are as comprehensive as possible
- FMD is the scientific term that is used in numerous peer-reviewed publications. The complaint that FMD is promoting a commercially available product is wrong; this product is sold as "ProLon" (not FMD) by L-Nutra
- there is absolutely a need to include a FMD since it is not simply a prolonged fasting but a very special type of prolonged fasting that actually allow people to eat while achieving many of the effects of fasting
- It needs to be emphasized that fasting mimicking isn't comparable to plain fasting because of the much higher caloric intake, plus there is no complete shut-down of the activity of the digestive system
- FMD differs from calorie restriction as it mimics water only fasting better and it obviously differs from water only fasting because calories are consumed.

No:

- This is a name which has been used for a commercial product as stated. Also it is a low calorie / fasting diet. There may be other versions of fasting mimicking diets in future with different macronutrient compositions. Lots of reasons to leave out

Although no consensus was reached, the editors propose to include the term FMD because of the growing body of research on this diet.

A **Fasting-Mimicking Diet (FMD)** specifies any diet that is specifically composed to induce the metabolic effects of fasting while allowing for a potentially higher caloric intake. It usually refers to a plant-based, calorie restricted diet with a maximum of 1000 kcal per day with solid food components that lasts 3 to 5 days and is followed periodically (e.g. once a month). **FMDs** are usually free of refined sugars and starch and low in protein.

Answer	n	%	% w/o A007
Strongly Agree (AO01)	8	26.67%	29,63%
Agree (AO02)	11	36.67%	40,74%
Neutral (A003)	1	3.33%	3,70%
Disagree (AO04)	2	6.67%	7,41%
Strongly Disagree (AO05)	2	6.67%	7,41%
This definition is irrelevant and should be excluded from the consensus process (AO06)	3	10.00%	11..11%
Due to my specialization, I am not familiar with this term / this method and prefer not to vote on it (AO07)	3	10.00%	

STRONGLY AGREE: further thoughts

P01: none

AGREE: further thoughts

P06: I propose a modification of this definition as follows:

"*Fasting-Mimicking Diet (FMD) specifies any diet that is specifically composed to induce the metabolic effects of fasting*, ***such as a reduction of blood glucose and growth factors, while allowing for a potentially higher caloric intake than fasting****. It usually refers to a plant-based, calorie restricted diet with a maximum of 1000 kcal per day that lasts 3 to 5 days, and which is followed periodically (e.g. every 2, 3 or 4 weeks, also depending on daily calorie content). FMDs are usually free of refined sugars and starch and low in protein content.* "

P05: I would delete the words "free of refined sugars and starch and" so that the last sentence reads: "FMDs are usually low in protein."

NEUTRAL: suggested modifications / alternative definition

P15: I suggest adding that FMD as a term and as a diet was originally developed by a for-profit company.

DISAGREE: suggested modifications / alternative definition

P23: *A Fasting-Mimicking Diet (FMD) refers to any diet that is specifically composed to induce the (metabolic) effects of fasting while allowing for intake of food. It usually concerns a plant-based, calorie restricted diet, free of refined sugars and starch and low in protein, containing a maximum of 1000 kcal per day, followed periodically for 3-5 consecutive days.*

P32: a very low protein low calorie diet with a maximum of 1000 kcal/day...

STRONGLY DISAGREE: suggested modifications / alternative definition

P33:	cant say It usually refers to a plant-based, calorie restricted diet with a maximum of 1000 kcal per day with solid food components that lasts 3 to 5 days and is followed periodically (e.g. once a month). FMDs are usually free of refined sugars and starch and low in protein.<br>This is one commercial version, there could be others
P11:	FMD is just another method of caloric restriction, because with a daily intake of up to 1000 Kcal you cannot induce the same metabolic effects of traditional fasting which requires far less calories.
**This definition is irrelevant and should be excluded – comments:**	
P17:	I cannot see that you considered FMD as industrial products without limitations eg as to content of minerals, amino acids, vitamins and so forth.<br>Free of refined sugars and vegan origin is too weak to associate it to fasting with natural products.
**RELIGIOUS FASTING**	**RELIGIOUS FASTING –** In the second round we asked you which of the following two definitions of religious fasting you prefer:

A1: A1: Religious fasting refers to any fasting regimen that is undertaken as part of a religious practice. Religious fasting thus involves practices such as: dry fasting at certain intervals over 24 hours (e.g. Jewish traditions, The Church of Jesus Christ of Latter-day Saints); intermittent dry fasting (e.g. Ramadan fasting, Bahá'í fasting); time-restricted eating (e.g. Buddhism); and diets restricting certain foods (e.g. Christian orthodox traditions, Daniel fast) if more broadly defined.

A2: Religious fasting refers to any fasting regimen that is undertaken as part of a religious practice. Religious fasting thus involves practices such as: dry fasting on specific days of the year up to 25 hours at a time (e.g. Jewish tradition, The Church of Jesus Christ of Latter-day Saints); intermittent dry fasting (e.g. Ramadan fasting, Bahá'í fasting); time-restricted eating (e.g. Buddhism); and diets restricting certain foods (e.g. Christian orthodox traditions, Daniel fast) if more broadly defined. Typically, religious fasting also includes spiritual activities to improve cognitive function and well-being.

Distribution of votes:

A1: ►**38.71 %** of the panel experts voted in favour of this definition.
A2: ►**51.61 %** of the panel experts voted in favour of this definition.
A3 (neither of the above): ►**9.68 %** of the panel experts voted "neither of the above".

As no clear favorite emerged, we offer the definition with the majority of votes from the last round (A2) with the additional synonyms of dry fasting (= total / complete fasting).

Religious fasting refers to any fasting regimen that is undertaken as part of a religious practice. Religious fasting thus involves practices such as: dry fasting (= total / complete fasting) on specific days of the year up to 25 hours at a time (e.g. Jewish tradition, The Church of Jesus Christ of Latter-day Saints); intermittent dry fasting (e.g. Ramadan fasting, Bahá'í fasting); time-restricted eating (e.g. Buddhism); and diets restricting certain foods (e.g. Christian orthodox traditions, Daniel fast) if more broadly defined. Typically, religious fasting also includes spiritual activities to improve cognitive function and well-being.

Answer	n	%	% w/o A007
Strongly Agree (AO01)	9	30.00%	31,03%
Agree (AO02)	14	46.67%	48,28%
Neutral (A003)	1	3.33%	3,45%
Disagree (AO04)	3	10.00%	10,34%
Strongly Disagree (AO05)	2	6.67%	6,90%
This definition is irrelevant and should be excluded from the consensus process (AO06)	0	0.00%	0.00%
Due to my specialization, I am not familiar with this term / this method and prefer not to vote on it (AO07)	1	3.33%	

**STRONGLY AGREE: further thoughts**	
P06:	For the reasons specified in the answer to a previous question, I **strongly disagree with considering dry fasting and total fasting as equivalent terms/approaches**. Indeed, while total fasting can be safe, well tolerated and, potentially, therapeutically efficacious in some human diseases even when prolonged (i.e., when it lasts for more than 48 consecutive hours), **dry fasting, which employs abstinence from beverage intake**, can be harmful, because it can lead, to hypotension, cardiac toxicity, blood electrolyte imbalance, dehydration and neurologic toxicities.
P01:	none
**AGREE: further thoughts**	
P14:	Final sentence should be edited to: *Typically, religious fasting also includes spiritual activities to improve cognitive function and well-being, as well as an individual's relationship with God.*
P05:	I don't feel strongly.
P18:	*Religious fasting refers to any fasting regimen that is undertaken as part of a religious practice. Religious fasting thus involves practices such as: dry fasting (= total / complete fasting) on specific days of the year* ***up to 72 hours***

	at a time (e.g. Christian orthodox tradition, Jewish tradition, The Church of Jesus Christ of Latter-day Saints); intermittent dry fasting (e.g. Christian orthodox tradition, Ramadan fasting, Bahá'í fasting); time-restricted eating (e.g. Buddhism); and diets restricting certain foods (e.g. Christian orthodox traditions, Daniel fast) if more broadly defined. Typically, religious fasting also includes spiritual activities to improve cognitive function and well-being.
P09:	*Religious fasting refers to any fasting regimen that is undertaken as part of a religious practice. Religious fasting thus involves practices such as: dry fasting (= total / complete fasting) on specific days of the year* ***up to 72 hours (christian orthodox tradition), 25 hours at a time (e.g. Jewish tradition, The Church of Jesus Christ of Latter-day Saints);*** *intermittent dry fasting (e.g. christian orthodox tradition, Ramadan fasting, Bahá'í fasting); time-restricted eating (e.g. Buddhism); and diets restricting certain foods (e.g. Christian orthodox traditions, Daniel fast) if more broadly defined. Typically, religious fasting also includes spiritual activities to improve cognitive function and well-being.*
**NEUTRAL: suggested modifications / alternative definition**	
P07:	**Dry food in the Christian Orthodox Church fasting**, today, means that **plant foods are allowed, but fresh** and not cooked. **Drinking water is allowed**. It usually lasts one day, sometimes up to three days (as I mentioned earlier).
**DISAGREE: suggested modifications / alternative definition**	
P23:	*Religious fasting refers to any fasting regimen that is undertaken as part of a religious practice.* I prefer short and concise definitions. Why add examples?
P33:	don't think we need to describe the spiritual elements in a nutrition definition
P25:	don't see why the last sentence should be part of the definition; we are defining food intake NOT other religious practices. Otherwise you need to start including things like special dress associated with the fasting, prayers, etc. It doesn't end. Let's stick to just food/liquid intake.
**STRONGLY DISAGREE: suggested modifications / alternative definition**	
P32:	A2
P10:	*Religious fasting refers to any fasting regimen that is undertaken as part of a religious practice. Religious fasting thus involves practices such as: dry fasting (= total / complete fasting) on specific days of the year up to 25 hours at a time (e.g. Jewish tradition, The Church of Jesus Christ of Latter-day Saints); intermittent dry fasting (e.g. Ramadan fasting, Bahá'í fasting); time-restricted eating (e.g. Buddhism); and diets restricting certain foods (e.g. Christian orthodox traditions, Daniel fast) if more broadly defined.* ***~~Typically, religious fasting also includes spiritual activities to improve cognitive function and well-being.~~***
**INTERMITTENT DRY FASTING:**	**INTERMITTENT DRY FASTING** – In the second round we offered two different terms with slightly different definitions:<br><br>**A1: Intermittent dry fasting (IDF)** refers to intermittent fasting regimens that involve abstaining from food and fluid intake during fasting hours. As daily fasting intervals generally depend on daylight hours, they may range from 9 to 20 hours. Diurnal intermittent dry fasting, as practiced in religious contexts, does not require any defined energy restriction during the nocturnal eating window.<br><br>**A2: Diurnal dry fasting**, as practiced in religious contexts, refers to a dietary regimen that involves abstaining from food and fluid intake during daylight hours for a certain period every year. The daily fasting window may range from 9 to 20 hours. There are no requirements on energy restriction during the nocturnal eating window.<br><br>Distribution of votes:<br><br>A1: ►**50.00 %** of the panel experts voted in favour of this definition.<br>A2: ►**42.86 %** of the panel experts voted in favour of this definition.<br>A3 (neither of the above): ►**7.14 %** of the panel experts voted in favour of this definition.<br><br>As no consensus was reached, we offer a definition proposed by one of the experts:<br><br>**Intermittent dry fasting (IDF)** refers to intermittent fasting regimens that involve abstaining from food and fluid intake during fasting hours. Most commonly, they range from 9 to 20 hours.

Answer	n	%	% w/o A007
Strongly Agree (AO01)	8	26.67%	29,63%
Agree (AO02)	15	50.00%	55,56%
Neutral (A003)	0	0.00%	0,00%
Disagree (AO04)	2	6.67%	7,41%
Strongly Disagree (AO05)	0	0.00%	0,00%
This definition is irrelevant and should be excluded from the consensus process (AO06)	2	6.67%	7,41%
Due to my specialization, I am not familiar with this term / this method and prefer not to vote on it (AO07)	3	10.00%	

**AGREE: further thoughts**	
P01:	none

P17:	Why don't you mention the **rules**? 9 -20 h seems arbitrarily which in the cases I know it is not.
DISAGREE: suggested modifications / alternative definition	
P23:	*Intermittent dry fasting (IDF) refers to intermittent fasting regimens that involve abstaining from food and fluid intake during fasting hours.*
P33:	think this should be **part of the religious section** - I very specific and should be called Ramadan needs to reflect this as **otherwise can confuse with the other terms of intermittent and dry fasting**

Terms on which consensus has been reached

This page is for your information only.

Terms concerning dietary and caloric restriction	
**DIETARY RESTRICTION (DR)**	**DIETARY RESTRICTION –** In the second round we asked you which of the following two definitions of dietary restriction you prefer:<br>**A1: Dietary restriction (DR)** comprises of chronic or intermittent restrictions in caloric intake and / or specific macronutrients and / or restraints of food intake within a specified time frame, without malnutrition. DR thus includes: all types of caloric restriction; fasting regimens such as short-term, long-term, and periodic fasting, intermittent fasting, time-restricted eating or feeding, water-only fasting, therapeutic fasting and fasting-mimicking diets; ketogenic diets; and diets with restrictions of specific macronutrients namely proteins, carbohydrates or fats.<br>**A2: Dietary restriction (DR) comprises of ~~chronic~~ continuous or intermittent** restrictions in caloric intake and/or specific macronutrients and/or restraints of food **or food and fluid intake** within a specified time frame. DR thus includes: all types of caloric restriction; fasting regimens such as short-term, long-term, and periodic fasting, intermittent fasting, time-restricted eating, water- and fluid-only fasting, therapeutic fasting and fasting-mimicking diets; and diets with restrictions of specific macronutrients namely proteins, carbohydrates, or fats.<br>Distribution of votes:<br>**A1:** ▶ **16.13 %** of the panel experts voted in favour of this definition.<br>**A2:** ▶ **77.42 %** of the panel experts voted in favour of this definition.<br>**A3** (neither of the above): ▶ **6.45 %** of the panel experts voted “neither of the above”.<br>With the reached consensus of **77.42 %,** A2 will be included in the publication as is.
**STARVATION**	**STARVATION –** The following definition has reached a consensus of **93.94 %** and will thus be included in the publication as is:<br>**Starvation** describes a catabolic process that occurs when the body’s reserves are exhausted after a prolonged period of insufficient energy and nutrient supply. Starvation can lead to serious health impairments, organ failure and death.
**General terms concerning fasting**	
**WATER-ONLY FASTING**	**WATER-ONLY FASTING** – In the second round we asked you which of the following two definitions of water-only fasting you prefer:<br>**A1: Water-only fasting** refers to a fasting regimen, where only water (and sometimes tea) is consumed for a certain period of time, usually ≥ 2 days.<br>**A2: Water-only fasting** refers to a fasting regimen, where only water is consumed for a certain period of time.<br>Distribution of votes:<br>**A1:** ▶ **25.00 %** of the panel experts voted in favour of this definition.<br>**A2:** ▶ **71.88 %** of the panel experts voted in favour of this definition.<br>**A3** (neither of the above): ▶ **3.13 %** of the panel experts voted “neither of the above”.<br>With the reached consensus of **71.88 %, A2** will be included in the publication as is.

Definitions to be discussed in the online consensus conference

In evaluating the results of the second round, we found that opinions on the nomenclature of the terms “**fasting**” and “**modified fasting**” vary widely around the world. Therefore, it was decided to exclude the term “fasting” and all terms that could possibly be classified under “modified fasting” (e.g. therapeutic fasting, alternate-day modified fasting) in this third round. However, the discussion and decision on these terms will be a central aspect of the upcoming live online discussion.

►In this section you can see the distribution of votes for the terms from round 2 to be discussed in the upcoming live online discussion.

General terms concerning fasting	
**FASTING**	**FASTING –** In the second round we asked you which of the following two definitions of fasting you prefer:<br>**A1: Fasting** refers to a voluntary abstinence from some or all foods and / or beverages for therapeutic, spiritual, or political reasons.<br>**A2: Fasting** refers to a voluntary abstinence from some or all foods or foods and beverages for preventive, therapeutic, religious, cultural, or other reasons.<br>Distribution of votes:<br>**A1**: ►**6.06 %** of the panel experts voted in favour of this definition.<br>**A2**: ►**84.85 %** of the panel experts voted in favour of this definition.<br>**A3** (neither of the above): ►**9.09 %** of the panel experts voted "neither of the above".<br>To see the experts' comments we received via email, please click here.<br>• I really think that **fasting is abstinence from all food (not fluid)**. It is confusing to include the consumption of some food into the definition, because you then have to define how many calories you still call fasting. I think Valter did a good job by coining the term "fasting mimicking diet" to describe diets that have effects similar to (water only) fasting.<br>• Historically and **currently** the term **fasting is used for water only fasting** or **low energy diets** which could be 5-600 kcal or other low kcal<br>• Patients think that **fasting means no food** and we have found it to be clearer to distinguish between "fasting" and "modified fasting". Thus, I would add a new concept of "modified fasting references means voluntary abstinence from most foods with only a limited amount of caloric intake (e.g., less than 800 kcal/day)".
**MODIFIED FASTING**	**MODIFIED FASTING –** definition:<br>The term **modified fasting** refers to limiting energy intake to 20-25% of energy needs on fasting days. Modified fasting regimens are often adapted to specific clinical settings and indications, allowing for different complementary or supportive therapeutic interventions.<br>The definition reached a consensus of **75.76%.** Although consensus was reached for this definition, several changes were proposed.<br>Expert comments:<br>• Since most people do not know their daily kcal needs, using a percentage may be difficult. May want to use an absolute amount, such as 500kcal per day max.<br>• I would consider a slight modification (broadening) of the first sentence to: "The term modified fasting refers to limiting energy intake, typically to 20-25% of energy needs, on fasting days." The second sentence could be retained in its present form.<br>• I think we should define "modified fasting" as a very-low-calorie diet (VLCD), which is typically defined as <= 800 kcal/day.<br>• The problem with using a threshold of 20-25% is that there are modified fasting studies already published that use up to ~35% of energy needs. So my second choice for a definition would be up to 35-40% of calories.<br>• I don't think we can specify the exact CR on fasting days → "typically 20-25% of energy needs"<br>• I would define modified fasting arbitrarily as "consumption of < 600 kcal/day". It is not necessarily adapted to specific clinical settings (although it is often used for reduction of bodyweight), so I would omit the second sentence.<br>• 25% of calories from the wrong source, let's say proteins could completely reverse many of the effects of fasting, so unless the term fasting mimicking diet is also included I'm against this term.
**FLUID-ONLY FASTING**	**FLUID-ONLY FASTING** – In the second round we asked you which of the following two definitions of fluid-only fasting you prefer:<br>**A1: Fluid-only fasting** refers to a fasting regimen, whereby only beverages are consumed for a certain period of time. Water and unsweetened herbal tea may be consumed *ad libitum*. In addition, clear vegetable broth, vegetable- and / or fruit juice, as well as *up to 2 cups of plain coffee and 2 teaspoons of honey* may be consumed per day, not exceeding a total of 800 kcal (consistent with the definition of a very low-calorie diet).<br>**A2: Fluid-only fasting** refers to a fasting regimen, whereby only beverages are consumed for a certain period of time. Water and unsweetened herbal tea may be consumed *ad libitum*. In addition, clear vegetable broth, vegetable- and / or fruit juice, as well as *up to 2 cups of plain coffee and 2 teaspoons of honey* may be consumed per day, not exceeding a total of 250 kcal.

	Distribution of votes:<br>**A1**: ►**29.03 %** of the panel experts voted in favour of this definition.<br>**A2**: ►**38.71 %** of the panel experts voted in favour of this definition.<br>**A3** (neither of the above): ►**32.26 %** of the panel experts voted "neither of the above".<br>Expert comments on A3: "**neither of the above**":<br>• I do not get the point of **honey** during fasting: This is counterintuitive. You should at least explain that Buchinger may do this due to psychological considerations (e.g. to enhanced compliance)<br>• Of the two choices, I think that the **800 kcal** limit makes sense because it is grounded in another well-established definition (I.e., a **very low-calorie diet**). However, for both options, I think that the **specific numerical recommendations for the cups of coffee and teaspoons of honey should be removed**. It is unclear why there are specific numerical limits for these items but not for vegetable broth and juices. For consistency and clarity, I think that no individual numerical limits should be placed on the specific allowed items. Rather, the single numerical recommendation could be in the form of the kcal limit (I.e., 800 or 250 kcal in these definitions). To me, this improves the flexibility and clarity of the definition<br>• **No consumption of psychotropic substances** (caffeine) in the form of coffee<br>• that would not make any sense. It does not really matter whether someone eats solid food and then drinks water or drinks the food with the water so fluid fasting should not be used because fasting has nothing to do with the solid or liquid form of calories placed in your mouth<br>• I think fasting refers to **consumption of zero calories**. These definitions will therefore confuse people. If calories are consumed in minute amounts, this is better referred to as calorie restriction or modified fasting.<br>**Please note the following regarding the reasoning of fluid-only fasting:** Drinking liquids such as vegetable broth and juices is permitted in the tradition of fluid-only fasting in Germany and has its background in supporting the fasting person psychologically to abstain from food.
**Continuous Fasting Regimens**	
**SHORT-TERM FASTING (STF)**	**SHORT-TERM FASTING** – In the last round, we asked you to indicate what duration you consider appropriate for the term short-term fasting<br>Distribution of votes:<br>(see table below)<br>Expert comments:<br>• I would **define short-term fasting as 1-3 days**, since I believe ketones bodies more or less plateau after 3 days. Long term could then be defined as ≥ 4 days.<br>• My first choice would be 2-4 days. My second choice is 1-4 days. I don't feel too strongly about this, other than **I don't think that less than 24 hours should be called short-term fasting.**<br>• I don't understand when this would be used. Assume this is not replacing intermittent fasting. I think it is difficult to define what is short and long based on ketones as the level of ketone formation will depend on whether it is a period of total fast or CR
**PROLONGED FASTING (PF) / LONG-TERM FASTING:**	**LONG-TERM FASTING** –In the last round, we asked you to indicate what duration you consider appropriate for the term long-term fasting.<br>**Prolonged fasting (PF)**, also called **long-term fasting (LTF)**, refers to any fluid-only fasting regimen lasting ≥ … days.<br>Distribution of votes:<br>(see table below)
**INTERMEDIATE DURATION FASTING:**	**INTERMEDIATE DURATION FASTING –** In the last round we asked you if you thought a fasting regimen such as "**intermediate duration fasting**" should be defined?<br>Distribution of votes:

Short-term fasting – distribution of votes:

Duration	Votes in %
16-72 hours (1-3 days)	30.30
24-48 hours (1-2 days)	21.21
48-72 hours (2-3 days)	27.27
24-96 hours (1-4 days)	15.15
24-120 hours (1-5 days)	6.06

Long-term fasting – distribution of votes:

Duration	Votes in %
≥ 3 consecutive days	40.63
≥ 4 consecutive days	28.13
≥ 5 consecutive days	28.13
≥ 7 consecutive days	3.13

Answer	Votes in %
Yes	34.38
No	65.63
No proference	0.00

We suggest not to define a term such as “intermediate duration fasting”, as most votes show that it is not necessary and as the proposed definitions of short-term and long-term fasting complement each other.

PERIODIC FASTING (PF)

PERIODIC FASTING – In the second round we asked you which of the following two definitions of periodic fasting you prefer:

A1: Periodic fasting (PF) refers to any fasting regimen that is repeated at regular intervals (periods), such as every day, every week, or every several months.

(Note: According to this definition, periodic fasting would include intermittent fasting regimens.)

A2: Periodic fasting (PF) refers to any fasting regimen lasting ≥ 48 hours that is repeated at regular intervals (periods), such as every several weeks or months.

Distribution of votes:

A1: ►**51.52 %** of the panel experts voted in favour of this definition.
A2: ►**42.42 %** of the panel experts voted in favour of this definition.
A3 (neither of the above): ►**6.06 %** of the panel experts voted “neither of the above”.

Expert comments:

- If we go with definition A2, then we need a term for intermittent fasting approaches such as fasting one day per week.
- we are confusing intermittent fasting with periodic fasting. Periodic fasting should refer to a fasting which **is repeated less than once every 2-4 weeks** and which **could be at regular intervals or not**. For example **someone could do periodic fasting once every year or every 3 months** or **even month** or do it **every month but then change to once in a while when needed**

As no consensus was reached, the editors propose to discuss this term and its definition in the **upcoming live discussion**. Please note that according to the Cambridge Dictionary, the term “periodic” in science refers to events happening repeatedly in a fixed pattern.

Intermittent Fasting Regimens

ALTERNATE-DAY FASTING (ADF)

ALTERNATE-DAY FASTING – In the second round we asked you which of the following two definitions of alternate-day fasting you prefer:

A1: Alternate-day fasting (ADF) or in animals, **every-other-day feeding (EOD),** refers to alternating a day of eating *ad libitum* and a day of either water-only fasting or a diet very low in calories.

A2: Alternate day fasting (ADF) refers to alternating a day of eating *ad libitum* and a day of water-only fasting.

Distribution of votes:

A1: ►**41.94 %** of the panel experts voted in favour of this definition.
A2: ►**54.84 %** of the panel experts voted in favour of this definition.
A3 (neither of the above): ►**3.23 %** of the panel experts voted “neither of the above”.

ALTERNATE-DAY MODIFIED FASTING (ADMF)

ALTERNATE-DAY MODIFIED FASTING (ADMF) – definition:
Alternate-day modified fasting (ADMF) refers to alternating a day of eating *ad libitum* and a day of eating a low-calorie diet with up to (…).
We asked you to select an energy equivalent for the modified fasting days.

Distribution of votes:

Answer	Votes in %
400 kcal	24.24
**500 kcal**	**12.12**
**600 kcal**	**9.09**
800 kcal	9.09
20-25 % of the usual energy intake	45.45

Specific Fasting Regimens

THERAPEUTIC / MEDICAL FASTING

THERAPEUTIC FASTING – In the second round we asked you which of the following two definitions of therapeutic fasting you prefer:

A1: Therapeutic fasting, also called **medical fasting**, refers to any fasting regimen that is applied as a therapeutic intervention by a trained physician.

	**A2: Therapeutic fasting** refers to any fasting regimen that is applied as a therapeutic intervention. **Medically supervised fasting** refers to any fasting regimen that is applied as a therapeutic intervention by a trained physician or similar credentialed healthcare provider.<br>Distribution of votes:<br>**A1**: ►**32.26 %** of the panel experts voted in favour of this definition.<br>**A2**: ►**67.74 %** of the panel experts voted in favour of this definition.<br>**A3** (neither of the above): ►**0.00 %** of the panel experts voted "neither of the above".
**BUCHINGER THERAPEUTIC FASTING**	**BUCHINGER THERAPEUTIC FASTING –** In the last round we asked you to vote on the following definition:<br>**BUCHINGER therapeutic fasting** is a fluid-only fasting regimen, allowing for a maximum of 500 kcal per day and lasting at least 5 days, practiced for the prevention or treatment of diseases as well as to support one's individual health, taking into account a person's medical, psychosocial and spiritual dimensions. It is usually accompanied by bowel / colon cleansing procedures and preceded and followed by a few days of a calorie restricted, easily digestible diet.<br>This definition of **BUCHINGER therapeutic fasting** reached a consensus of **91.31 %**. Depending on the discussions in the live workshop about the modified fasting regimens, the term "Buchinger therapeutic fasting" may need to be adapted.
**FX-MAYR-THERAPY**	The following definition of FX-Mayr Therapy reached a consensus of **77.27%**:<br>**FX-Mayr-Therapy** or **FX-Mayr Cure** refers to a 3-phase fasting regimen containing elements of water-only fasting, a very low-calorie diet with a training of "proper chewing", in order to help individuals (re-)gain their sense of satiety and an easily digestible diet towards the end of the treatment. The dietary intervention is accompanied by manual treatments focusing on the abdominal region.<br>However, an expert on **FX-Mayr therapy** suggested a few more changes to the definition to ensure it is up to date. After the decision-making process in the live online discussion, the final definition will be presented again.
**GRUEL FASTING (traditional german fasting regimen)**	**GRUEL FASTING –** In the last round we asked you to vote on the following definition:<br>**Gruel fasting** refers to a fasting regimen in which 400 ml of oat or rice gruel is given in three portions per day. Water and unsweetened herbal tea may be consumed *ad libitum*. Gruel fasting provides about 200 kcal and 45 g of carbohydrates per day.<br>As neither consensus (**agreement votes: 50.00 %**), nor exclusion (**exclusion votes: 42.31 %)** was reached, the editors suggest to simply include the term gruel fasting with a note in the definition of **fluid-only fasting**.

Wishes of the experts for the online consensus conference

**Workshop preparation**	
P35:	**Medically supervised water-only fasting**. The complete abstinence from all substances except pure water in an environment of rest.<br>There is a growing body of peer-reviewed literature on this specific process with the rest being an important component in safety and clinical outcome. Our recent safety study using DEXA data supports a preservation of lean tissue and bone preservation with this specific protocol.
P15:	None to suggest at this time.
P06:	I found this survey very interesting and comprehensive.<br>Along with the definitions on which we still need to find a shared agreement, in the online discussion session I also propose to discuss the **potential clinical applications/evidence** for **each of these approaches**, or, as an **alternative, to discuss and cite some studies in which these approaches were evaluated in human subjects**. Along with the definitions on which we still need to find a shared agreement,
P05:	I think you covered all the topics that I think we should discuss. I would focus on the following topics:<br>(1) creating an overarching definition of the **minimum duration of fasting** needed for something to constitute **intermittent fasting**,<br>(2) **definition of modified fasting**,<br>(3) the **cut-off for IF vs. long-term fasting**, and<br>(4) definition of **alternate-day fasting**.
P13:	I think the primary items that need to be discussed are clear after this round of the survey, particularly the previous page which outlined the definitions to be discussed. As alluded to on that page, some of the **distinctions between fasting vs. modified fasting, short-term vs. long-term fasting,** etc., may need to be resolved. For these, I think an important point will be to ensure that **no fasting programs are left somewhere "in between" two apparently dichotomous definitions**.

	For example, and as mentioned, there **shouldn't be any durations that fall between short-term and long-term** if those two terms are used without inclusion of something like "intermediate duration fasting." As a general comment, some of the disagreement about definitions could possibly relate to information that is more specific than necessary being included in the definitions. My opinion is that the **definitions should be clear and informative but not overly restrictive**. Then, the **publication text accompanying the definition could provide additional explanations**, thoughts, etc. to further inform the reader. Of course, specific numerical ranges or examples may be needed in some definitions, so this isn't to say that all definitions should be overly broad. I think the current versions of the definitions largely abide by this general recommendation, which is encouraging! If I think of any additional feedback prior to August 15th, I will send this via email. Thank you for facilitating this productive dialogue! I am very excited for these definitions to be finalized and move towards publication/dissemination. I think they will help researchers and practitioners immensely!
P33:	there are too many dates / times in the doodle poll, I can make a meeting sometime in october
P17:	esp continuing **fasting** in discrimination to all kinds of **perdioic fasting**. Some authors tried to introduce periodic fasting for isolated phases of several days of fasting which I think is not a good term. In the European tradition of Natural Medicine, it is also important whether you use natural or industrial products.

Online Consensus Conference

Online Consensus Conference Preparation Document

Aim of the discussion: Defining existing fasting forms - especially forms for which there are publications (Note: there will be no evaluation of existing evidence, safety/feasibility of existing fasting regimens or construction of possible new fasting forms.)

Disclaimer: The conversation will be recorded solely for the purpose of the consensus process. The recording will not be published or distributed on any platform, and it will be deleted after evaluation. Anonymity cannot be kept during the discussion.

Rules for the discussion:

- Raise virtual hand
- You are welcome to post references in the chat where necessary

---

Times in CET	Agenda
**7.00 - 7:30 pm**	Introduction – facilitators and experts: name, workplace, clinic / research, fasting expertise, motivation for consensus process
**7.30 - 8.15 pm**	Fasting / modified fasting / total fast
8.15 **- 8.25 pm**	Alternate-day (modified) fasting
**8.25 - 8.35 pm**	BREAK
**8.35 - 8.55 pm**	Periodic fasting
**8.55 - 9.15 pm**	Durations for short- & long-term fasting
**8.15 - 9.30 pm**	Intermittent energy restriction / Intermittent fasting / Time-restricted eating
**9.30 - 9.45 pm**	Caloric restriction
**9.45 - 10.00 pm**	**CLOSURE**

Guide to layout, colors & italics in the following text

The text below is laid out as follows:

1. **TERM TO BE DISCUSSED**

Current state of the discussion

❖ Questions to be discussed in the workshop

Detailed information	on each term with all relevant comments arranged according to survey round

❖ Comments that occur more than once are highlighted in orange / light blue / purple

❖ Terms highlighted in pink: possible synonyms

❖ Text in italics: alternative definitions for terms

---

1. FASTING - MODIFIED FASTING - TOTAL FAST

In our survey opinions on the nomenclature of the terms **fasting** and **modified fasting** have varied widely. Please take note of the definitions of “fast” (verb and noun) from the Cambridge, Oxford and Merriam Webster`s dictionaries.

“FAST” according to dictionaries:

	Cambridge	Merriam-Webster	Oxford Dictionary of English
**noun**	a period of time when you **eat no food**	the practice of fasting; a time of fasting	an act or period of fasting
**verb**	to eat **no food** for a period of time	to **abstain from food**;<br>to **eat sparingly** or **abstain from some foods**	abstain from **all or some kinds of food or drink**, especially as a religious observance<br>- (be fasted): be deprived of all or some kinds of food, especially for medical or experimental reasons: all patients were fasted before surgery.
**etymology**	**fast (verb):** "abstain from food," Old English fæstan "to fast" (as a religious duty), also "to make firm; establish, confirm, pledge," from Proto-Germanic *fastanan "to hold, guard," extended to the religious act "observe abstinence" (...) (adj.). The original meaning in prehistoric Germanic was "hold firmly," and the sense evolved via "have firm control of oneself," to "hold oneself to observance" (compare Gothic fastan "to keep, observe," also "to fast"). (...)<br>**fast (noun):** "act of fasting," late Old English fæsten "voluntary abstinence from food and drink or from certain kinds of food," especially, but not necessarily, as a religious duty; (...) In earlier Old English fæsten meant "fortress, cloister, enclosure, prison."<br>Source: https://www.etymonline.com/word/fast		

1.1 FASTING

The following definition reached a consensus of 84.85% in the second survey:

> ***Fasting** refers to a voluntary abstinence from some or all foods or foods and beverages for preventive, therapeutic, religious, cultural, or other reasons.*

However, some participants criticized this definition for allowing energy intake during fasting. Therefore, it was decided to include the term in the live discussion instead of presenting it again in the third survey.

QUESTIONS TO BE DISCUSSED:

❖ Could the term **fasting** serve as an umbrella term for all kinds of fasting?

❖ If **fasting** was defined as complete abstention from all foods and calorie-containing beverages (à only water / tea allowed), would **fluid-only fasting** (incl. juice / broth / linseed gruel or porridge) and **religious fasting** have to count as **modified fasting regimens**?

❖ Reminder: We have reached consensus on the term **religious fasting** together, defining it as “any fasting regimen that is undertaken as part of a religious practice”, thus including dry fasting, intermittent dry fasting and diets restricting certain foods as practiced in Christian orthodox traditions.

**First round**	
**FASTING**	**Fasting** refers to a voluntary abstinence from some or all foods and / or beverages for therapeutic, spiritual, or political reasons.<br>**Distribution of votes:**<br>► **88.23 %** of the panel experts voted in favour of this definition.<br>► **5.88 %** of the panel experts disagreed or strongly disagreed.

**STRONGLY AGREE: Further thoughts on given definition / Explanation of choice**	
1.	The one caveat I suggest is that if fasting is used for some foods or beverages that the specific items or classes of items should be noted along with the use of the word fasting. If the term is used alone then it should generally be assumed that it is total or complete fasting from caloric intake, abstaining voluntarily from all foods and beverages (except water or non-caloric fluids, although I tend to say water-only fasting when water is allowed or consumed).
2.	could use the term total fast or partial fast
**AGREE Suggested modifications / alternative definition**	
6.	Solid food instead of some
**NEUTRAL: Suggested modifications / alternative definition**	
8.	The definition needs to allow water to be consumed and should say all foods (not just some foods). I also suggest a time limit. I suggest that the definition be revised to: "*Fasting refers to a voluntary abstinence from all food and calorie-containing beverages for at least 14 hours*."
9.	*Fasting is the complete abstinence of off substances, except pure water.*
**DISAGREE: Suggested modifications / alternative definition**	
10.	*Fasting refers to a voluntary abstinence from ~~some or~~ all foods and / or beverages for therapeutic, spiritual, or political reasons*.
**STRONGLY DISAGREE: Suggested modifications / alternative definition**	
11.	Definition should read: *Fasting refers to voluntary abstinence from **energy intake** for therapeutic, spiritual, or political reasons.*
**Second round**	
**FASTING**	**A1 (original wording): Fasting** refers to a voluntary abstinence from some or all foods and / or beverages for therapeutic, spiritual, or political reasons.<br>**A2 (proposed changes incorporated): Fasting** refers to a voluntary abstinence from some or all foods ~~and /~~ or foods and beverages for preventive, therapeutic, ~~spiritual~~ religious, cultural, or ~~political~~ other reasons.<br>**Distribution of votes:**<br>A1: ►**6.06 %** of the panel experts voted in favour of this definition.<br>A2: ►**84.85 %** of the panel experts voted in favour of this definition.<br>A3 (neither of the above): ►**9.09 %** of the panel experts voted "neither of the above".
**Neither of the above (I disagree with both definitions)**	
1.	A3*: Fasting refers to voluntary abstinence from some or all foods and / or beverages for preventive, therapeutic, religious, or political other reasons.*
2.	I have changed my mind. I prefer the definition: ***"Fasting refers to a voluntary abstinence from all foods and calorie-containing beverages."***
3.	I think **fasting should refer to abstinence from all (not some) foods or foods and caloric beverages** for preventive, therapeutic, ~~spiritual~~ religious, ~~cultural,~~ or other reasons.
**Experts asked via email regarding the orange comments:**	
2.	Patients think that **fasting means no food** and we have found it to be clearer to distinguish between "fasting" and "modified fasting". Thus, I would add a new concept of "modified fasting references means voluntary abstinence from most foods with only a limited amount of caloric intake (e.g., less than 800 kcal/day)".
3.	I really think that **fasting is abstinence from all food (not fluid)**. It is confusing to include the consumption of some food into the definition, because you then have to define how many calories you still call fasting. I think Valter did a good job by coining the term "fasting mimicking diet" to describe diets that have effects similar to (water only) fasting.
	Another expert who was originally asked about alternate-day fasting:<br>Historically and **currently** the term "**fasting" is used for water only fasting** or **low energy diets** which could be 5-600 kcal or other low kcal

1.2 MODIFIED FASTING

At the end of the first survey, we asked you if there were any other terms that you considered important to this consensus process. **Modified fasting** was one of the requested terms and was therefore included in the second round of the survey.

The following definition reached a consensus of **75.76%** in the **second** survey:

> *The term **modified fasting** refers to limiting energy intake to 20-25% of energy needs on fasting days.*
> *Modified fasting regimens are often adapted to specific clinical settings and indications, allowing for different complementary or supportive therapeutic interventions.*

However, some participants suggested several changes for this definition. Therefore, it was decided to include the term in the live discussion instead of presenting it again in the third survey.

QUESTIONS TO BE DISCUSSED:

- How should **modified fasting** be defined?
- Should the term **fasting-mimicking diet** be part of the definition?
- Should the definition have a specific **maximum caloric intake**?
- If so, should the maximum caloric intake be a **range** or an **absolute number** of calories?

Based on the comments in the second survey round, we created two options for discussion / re-evaluation.

A1: The term **modified fasting** refers to limiting energy intake, typically to 20-25% of energy needs on fasting days. Modified fasting regimens are often adapted to specific clinical settings and indications, allowing for different complementary or supportive therapeutic interventions.

A2: The term **modified fasting** refers to limiting energy intake, typically to 20-50% of energy needs on fasting days. Modified fasting regimens are often adapted to specific clinical settings and indications, allowing for different complementary or supportive therapeutic interventions. An optimal composition of modified fasting can be achieved with a fasting-mimicking diet (FMD).

**Second round** (term was included in the second round only)	
**MODIFIED FASTING**	The term **modified fasting** refers to limiting energy intake to 20-25% of energy needs on fasting days. Modified fasting regimens are often adapted to specific clinical settings and indications, allowing for different complementary or supportive therapeutic interventions.<br>The definition reached a consensus of **75.76%.** Although consensus was reached, several changes were proposed. See comments below.
**AGREE: Suggested modifications / alternative definition**	
1.	Since most **people do not know their daily kcal needs**, **using a percentage may be difficult**. May want to use an absolute amount, such as 500kcal per day max.
2.	I would consider a slight modification (broadening) of the first sentence to: "*The term modified fasting refers to limiting energy intake, **typically** to 20-25% of energy needs, on fasting days*." The second sentence could be retained in its present form.
**NEUTRAL: Suggested modifications / alternative definition**	
3.	*The term modified fasting refers to limiting energy intake to 20-**50%** of energy needs on fasting days. Modified fasting regimens are often adapted to specific clinical settings and indications, allowing for different complementary or supportive therapeutic interventions.*
**DISAGREE: Suggested modifications / alternative definition**	
4.	I think we should define "modified fasting" as a **very-low-calorie diet** (VLCD), which is typically defined as <= 800 kcal/day.
5.	The problem with using a threshold of 20-25% is that there are **modified fasting studies already published** that use up **to ~35% of energy needs**. So, my second choice for a definition would be **up to 35-40% of calories.**
6.	Why don't we refer to daily caloric intake e.g. 700-800 calories?
7.	don't think we can specify the exact CR on fasting days, should say: *The term modified fasting refers to limiting energy intake **to a proportion of the energy needs** on fasting days, **typically** 20-25% of energy needs. Modified fasting regimens are often adapted to specific clinical settings and indications, allowing for different complementary or supportive therapeutic interventions.*
8.	I would define modified fasting arbitrarily as "**consumption of < 600 kcal/day**". It is **not necessarily adapted to specific clinical settings** (although it is often used for reduction of bodyweight), so I would omit the second sentence.
**STRONGLY DISAGREE: Suggested modifications / alternative definition**	
9.	**25% of calories from the wrong source, let's say proteins could completely reverse many of the effects of fasting**, so unless the term **fasting mimicking diet** is also included I'm against this term.
**Experts asked via email regarding their comments:**	
4.	I prefer the term "**modified fasting**" to describe **partial fasting** with **a limited amount of caloric intake**". Patients think that fasting means no food and we have found it to be clearer to distinguish between "fasting" and "modified fasting". Thus, I would add a new concept of "modified fasting references means voluntary abstinence from most foods with only a limited amount of caloric intake (e.g., less than 800 kcal/day)". The wording here is not perfect, but I like something along these lines.<br>For instance, using the modified fasting term allows us to distinguish between alternate-day fasting (zero intake every other day) versus alternate-day MODIFIED fasting (limited intake every other day). It also allows us to discuss diets like the 5:2 diet as forms of intermittent fasting that involve "modified fasting." Then the 5:2 diet, ADMF, FMD, Buchinger fasting, etc., can all be classified as modified fasting regimens.

	Another expert who was originally asked about alternate-day fasting:<br>**Modified alternate day fasting** - fast days with 500-600 kcal

1.3 TOTAL FAST

The term **total / complete fast** was included as an additional term in the second survey because some experts had mentioned this term as an alternative to the general term **fasting** or **dry fasting** in the first round of the survey. However, we wanted to define total fasting separately to distinguish it more clearly from similar terms like **fasting**, **water-only fasting,** and **fluid-only fasting.** In our proposed definition we included the historical term **zero-calorie diet**.

The following definition reached a consensus of **85.85 %** in the **second** survey:

> *The term **total fast**, or **complete fast**, refers to a fasting regimen, where only calorie-free beverages, including water and unsweetened tea, are consumed ad libitum for a certain period of time. Historically, prolonged total fasts were used for the therapy of people with obesity under the term **zero-calorie diet**.*

Two participants suggested that this definition should rather be titled **fasting** or **water-only fasting.** Another two participants again pointed out that the term **total fast** would better fit the definition of **dry fasting**. It was therefore decided to delete the above definition and list the terms **total fasting / complete fasting** under **dry fasting** in the third round of the survey. This was criticized by several participants in the third round (see comments under the term **dry fasting**).

QUESTIONS TO BE DISCUSSED:

- Should the term **total / complete fasting** equal the term **fasting / water-only fasting**? OR Should the term **total / complete fast** be omitted altogether?
- Should the term **total / complete fasting** equal the term **dry fasting**?

**Second round** (term was included in the second round only)	
**TOTAL FAST**	The term **total fast**, or **complete fast**, refers to a fasting regimen, where only calorie-free beverages, including water and unsweetened tea, are consumed *ad libitum* for a certain period of time. Historically, prolonged total fasts were used for the therapy of people with obesity under the term **zero-calorie diet**.<br><br>Although the above definition reached a consensus of **85.85 %,** a few dry fasting experts mentioned that the terms better fit the definition of "**dry fasting**". It was therefore decided to **delete** the above definition and list the terms "**total fasting / complete fasting**" under "**dry fasting**" in the third survey.
AGREE: Suggested modifications / alternative definition	
1.	The only change I would recommend is to remove or modify the last sentence. If retained, I think it could be simplified to: "*Historically, prolonged total fasts were used under the term zero-calorie diet.*" I personally think the other information (about therapy of people with obesity) is unnecessary detail for a definition.
2.	We strongly feel that zero calorie beverages should be excluded of any current definition of fasting since they have been to be harmful for one's health.
3.	would add to the above in parenthesis: "*When used to describe religious fasting, the term "**total fast**" can also mean abstaining from liquids also.*"
4.	I would omit the last sentence and define the period as > 24 hours
NEUTRAL: Suggested modifications / alternative definition	
5.	This would be a supplemented complete fast.
6.	I would use the term "water-only fasting" or simply "**fasting**" instead of total fast or complete fast. Also, I would delete the sentence "Historically, prolonged total fasts were used for the therapy of people with obesity under the term zero-calorie diet."
DISAGREE: Suggested modifications / alternative definition	
7.	*The term total fast, or complete fast, refers to a fasting regimen, where only water and unsweetened tea, are consumed **up to a regulated quantity** for a certain period of time.*<br>The term zero-calorie diet should not be confused with fasting, since it includes zero calorie beverages, being harmful for one's health.
8.	I think it is better to call it water only fasting. If someone wants to replace it with tea then ok, but it is still essentially a water only fast. The risk with other names is that people will think some calorie can be included (buchinger fast etc)
STRONGLY DISAGREE: Suggested modifications / alternative definition	
9.	In **total fast**, food and beverages should not be consumed such as during Ramadan fasting.

**Expert asked via email regarding the blue comments (3.):**	
3.	Total/complete fast is abstaining from ALL food and liquid intake including water (eg Yom Kippur)

1.5 DRY FASTING

The following definition of **dry fasting** reached a consensus of **87.50%** already in the **second** round of the survey.

> ***Dry Fasting** refers to a fasting regimen, during which a voluntary abstinence from all foods and beverages, including water, is practiced for a certain period of time.*

However, as mentioned above, two participants pointed out that the term **total fast** would correspond to the definition of **dry fasting**. For this reason, we included the terms **total fasting / complete fasting** in the definition of **dry fasting** in the third round of the survey.
This modified definition then reached a consensus of **74.08 %** in the **third** survey:

> ***Dry Fasting**, also called **total fasting / complete fasting**, refers to a fasting regimen, during which a voluntary abstinence from all foods and beverages, including water, is practiced for a certain period of time.*

Equating dry fasting with total fasting was criticized by several participants in the third round:

QUESTION TO BE DISCUSSED:

❖ Which definition of **dry fasting** is preferred - that of the second or third survey?

**First round**	
**DRY FASTING (DF)**	**Dry Fasting** refers to a fasting regimen, during which an abstinence from all foods and beverages is practiced for a limited period of time.<br>▶ **64.70 %** of the panel experts voted in favour of this definition.<br>▶ **14.71 %** of the panel experts voted neutral.<br>▶ **5.88 %** of the panel experts selected “disagree / strongly disagree”.<br>▶ **14.71 %** of the panel experts voted that this definition is irrelevant.
**NEUTRAL: Suggested modifications / alternative definition**	
1.	I was not familiar with the term ‘dry fasting’. I would call abstinence from all foods and beverages ‘complete fasting’.
2.	I woud add “a limited period of time” referring to hours and /or days
3.	I recommend using nearly identical language to the language used for regular fasting. Here is my recommendation for the wording for a definition: *“Dry Fasting refers to voluntary abstinence from all foods and beverages, including water, for several hours.”*
4.	could be a total diet & fluid fast
**DISAGREE: Suggested modifications / alternative definition**	
4.	Excluding water and tea
**STRONGLY DISAGREE: Suggested modifications / alternative definition**	
5.	don’t like the term. Would rather use “total fasting”
**Second round**	
**DRY FASTING (DF)**	**Dry Fasting** refers to a fasting regimen, during which a voluntary abstinence from all foods and beverages, including water, is practiced for a certain period of time.<br>▶ **87.50 %** of the panel experts voted in favour of this definition.<br>▶ **3.13 %** of the panel experts voted neutral.<br>▶ **3.13 %** of the panel experts selected “disagree / strongly disagree”.<br>▶ **6.25 %** of the panel experts voted that this definition is irrelevant.
**AGREE: Suggested modifications / alternative definition**	
1.	*Dry Fasting refers to a fasting regimen, during which a voluntary abstinence from all foods and beverages, including water, is practiced **for up to a few days**.*
2.	it corresponds to total fasting.
3.	I would suggest "*a regimen whereby no food of drinks (including water) are consumed whatsoever*".
**This definition is irrelevant and should be excluded from the consensus process – Comments**	
4.	not clear how this differs from regular fasting if you’re excluding “food and beverages”
**Third round**	

**DRY FASTING (DF)**	**Dry Fasting**, also called **total fasting / complete fasting**, refers to a fasting regimen, during which a voluntary abstinence from all foods and beverages, including water, is practiced for a certain period of time.<br>►**74.08 %** of the panel experts voted in favour of this definition.<br>►**25.92 %** of the panel experts selected "disagree / strongly disagree".
**DISAGREE: suggested modifications / alternative definition**	
1.	**Dry food in the Orthodox Church fasting**, today, means that **plant foods are allowed, but fresh and not cooked**. **Drinking water is allowed**. It usually lasts one day, sometimes up to three days.
2.	I do not like the addendum in blue, because complete or total fasting to me seems absence of food and drinks, but not from water
3.	think useful to define whether total food (calorie) fast or food and fluid fast
4.	Commonly, in these times, the term total is used for zero diet (but with water or tea intake) If we choose the above proposed definition a lot of published studies would be misinterpreted
**STRONGLY DISAGREE: suggested modifications / alternative definition**	
5.	Dry fasting should not be referred to as total/complete fasting as that will create confusion on the well-established practice of referring to water-only fasting as complete or total fasting.
6.	I strongly disagree with considering "dry fasting" as equivalent to "total/complete fasting".<br>Indeed, while these types of fasting could be similar in terms of calorie intake restriction, there are huge physiological and therapeutic differences between these two approaches. In particular, while "total/complete fasting" can be safe, well tolerated and, potentially, therapeutically effective even when prolonged (i.e., when lasting for 2 or more days), "dry fasting", which also implies total abstinence from the intake of beverages, including water, is potentially harmful, in that it can cause hypotension, dehydration, damage to the kidneys, blood electrolyte imbalance and other severe side effects.<br>*Dry Fasting refers to a fasting regimen, during which a voluntary abstinence from all foods and beverages, including water, is practiced for a certain period of time.*

2. ALTERNATE-DAY (MODIFIED) FASTING

2.1 ALTERNATE-DAY FASTING

The following definition reached a consensus of **82.35 %** in the **first** round of the survey:

> ***Alternate day fasting (ADF)** refers to alternating a day of eating ad libitum and a day of either water-only fasting or a diet very low in calories.*
>
> (Note that "or, in animals, **every-other-day feeding (EOD)**" was removed as it was decided to focus on fasting terms in humans.)

However, some participants requested a distinction between **ADF** and the term **alternate-day modified fasting (ADMF),** which allows some caloric intake on the fasting day. Therefore, in the second survey, we asked you to choose either the ADF definition from above or one that allows only water on the fasting day. In this second round, no clear favorite emerged. Since there were also differing opinions on the definitions of the general terms **fasting** and **modified fasting,** it was decided to include the terms **alternate-day fasting** and **alternate-day modified fasting** in the live discussion as well rather than presenting them again in the third round.

QUESTION TO BE DISCUSSED:

- Should we have two separate definitions for **alternate-day fasting** and **alternate-day modified fasting** or combine the two, keeping the definition of the first round of the survey?

If the definitions for ADF and ADMF should be separate, we propose to choose one of these three definitions of **ADF**:

A1: Alternate-day fasting (ADF) refers to alternating a day of eating *ad libitum* and a day of either water-only fasting or a diet very low in calories.

A2: Alternate day fasting (ADF) refers to alternating a day of eating *ad libitum* and a day of water-only fasting.

A3: Alternate day fasting (ADF) refers to alternating a day of eating *ad libitum* and a fasting day where only water and energy-free beverages are consumed.

First round	
**ALTERNATE-DAY**	**Alternate day fasting (ADF),** or in animals, **every-other-day feeding (EOD),** refers to alternating a day of eating *ad libitum* and a day of either water-only fasting or a diet very low in calories.

**FASTING (ADF)**	► **82.35 %** of the panel experts voted in favour of this definition.<br>► **2.94 %** of the panel experts voted neutral.<br>► **11.76 %** of the panel experts selected "disagree / strongly disagree".<br>► **2.94 %** of the panel experts voted that this definition is irrelevant.
**STRONGLY AGREE: Further thoughts on given definition / Explanation of choice**	
1.	ADF should not be used if any carbs are consumed within middle of the day as it will activate insulin pathways.
**AGREE: Suggested modifications / alternative definition**	
2.	Some could argue that ADF could either involve ad libitum or prescribed energy intake on the eating day. So, if desired, the definition could be broadened to: "*Alternate-day fasting (ADF) or in animals, every-other-day feeding (EOD), refers to alternating a day of ad libitum or prescribed eating and a day of either water-only fasting or a diet very low in calories.*"
3.	define very low in calories as 600kcal or less
4.	I would not allow the consumption of anything except water/tea or coffee if I call something 'fasting'
5.	I would recommend defining very low in calories to
6.	very low: how many? Below 400 per day?
7.	ADF is with total fast whereas ADMF allows for low calorie diet every other day
8.	think we should specify the cal intake on the fasting days and make clear that free fluids allowed
9.	only water, no inclusion of low calorie diet
**NEUTRAL: Suggested modifications / alternative definition**	
10.	*Alternate-day fasting (ADF) or in animals, every-other-day feeding (EOD), refers to alternating a day of eating ad libitum and a fasting day*. The duration of fasting is not mentioned. Is it 24 hours?
**DISAGREE: Suggested modifications / alternative definition**	
11.	This is not the case in periodic Orthodox fasting, where ''non-restrictive '' days are not suggested for ad libitum food intake, rather than increasing total caloric intake to 1500-2000 according sex and age<br>ADF (or ALF) in humans usually refers to restricting caloric intake in 2-3 days a week, returning to usual caloric intake (not ad libitum) in the rest of the week, or even setting an upper limit of 2000 -2500 kcal (women/men) in the non-restrictive days. This is the case in Christian Orthodox fasting, a subtype of religious fasting, which is a rather a specific subtype of ADF. My suggestion would be to include a few questions on religious fasting regimens, in general, which would add to the scientific value of the consensus and personalize according religious beliefs, which is followed by a large part of individuals which practice IF. I would be willing to help on this task, if it is needed. Religious fasting practices, are a bit different by those followed for health reasons (i.e chrononutrition followed in Ramadan, significantly impacts metabolic effects of this pattern, etc.)
**STRONGLY DISAGREE: Suggested modifications / alternative definition**	
12.	*Alternate-day fasting (ADF) or in animals, every-other-day feeding (EOD), refers to alternating a day of regulated eating and a day of water-only fasting or a diet very low in calories.*
13.	*Alternate-day fasting (ADF) or in animals, every-other-day feeding (EOD), refers to alternating a day of regulated eating and a day of either water-only fasting or a diet very low in calories.*
14.	Here is how I would revise the definition: "*Alternate-day fasting (ADF) refers to alternating days of eating ad libitum with days of water-only fasting.*" I strongly feel that we should not include Alternate-Day Modified Fasting (ADMF) in the definition. I would define **ADMF** as: "*Alternate-day modified fasting (ADMF) refers to alternating days of eating ad libitum with days eating a very-low-calorie diet (VLCD).*
**Second round**	
**ALTERNATE -DAY FASTING (ADF)**	**A1: Alternate-day fasting (ADF)** or in animals, **every-other-day feeding (EOD),** refers to alternating a day of eating *ad libitum* and a day of either water-only fasting or a diet very low in calories.<br>**A2: Alternate day fasting (ADF)** ~~or in animals,~~ **~~every-other-day feeding (EOD),~~**refers to alternating a day of eating *ad libitum* and a day of ~~either~~ water-only fasting ~~or a diet very low in calories~~.<br>**Distribution of votes:**<br>A1: ► **41.94 %** of the panel experts voted in favour of this definition.<br>A2: ► **54.84 %** of the panel experts voted in favour of this definition.<br>A3: ► **3.23 %** of the panel experts voted "neither of the above".
**I agree with A2: modified definition**	
1.	Could specify low carb calories i.e. not activating insulin signaling pathways if you ate
**Neither of the above (I disagree with both definitions)**	
2.	*Alternate-day fasting (ADF) refers to alternating a day of either water-only fasting or a diet very low in calories and a day of eating ad libitum or a modified diet i.e. a healthy Mediterranean diet*

2.2 ALTERNATE-DAY MODIFIED FASTING

It was suggested that the term **alternate-day modified fasting**, which allows some caloric intake on the fasting day, be included as an extra term in the **second** round of the survey to distinguish this modified fasting regimen from **alternate-day fasting.** We asked you to select a maximum number of calories for the modified fasting days, but no consensus was reached.

QUESTIONS TO BE DISCUSSED:

- If we define **alternate-day modified fasting** separately from **alternate-day fasting** what maximum number of calories for the modified fasting day do you suggest?

We propose the following definition for **ADMF** based on the responses of the **alternate-day fasting** experts we asked via email (see their answers below):

Alternate-day modified fasting (ADMF) refers to alternating a day of eating *ad libitum* and a day of eating a low-calorie diet with up to 500 - 600 kcal.

**Second round** (term was included in the second round only)	
ALTERNATE -DAY MODIFIED FASTING (ADMF)	**Alternate-day modified fasting (ADMF)** refers to alternating a day of eating *ad libitum* and a day of eating a low-calorie diet with up to (see below).<br>We asked you to select an energy equivalent for the modified fasting days:<br>**Distribution of votes:**

Answer	Votes in %
400 kcal	24.24
500 kcal	12.12
600 kcal	9.09
800 kcal	9.09
20-25 % of the usual energy intake	45.45

**500 kcal:**	
1.	Should be part of the background text
**800 kcal:**	
2.	Equivalently, I would be in favor of up to ~35% of energy intake on the **modified fasting days**.
3.	Does this (or this question) make sense at all?
**20 to 25% of the usual energy intake:**	
4.	**To me, the use of a relative intake (%) makes the definition much more flexible than absolute (kcal) units due to the widely varying energy needs of humans.**
5.	a review of the literature is necessary to see what constitute. A calorie limit on the fast day
6.	But also need to specify what and when it is eaten
7.	My suggestion is based on the assumption that daily caloric intake matches daily caloric needs.
8.	I disagree. The term **fasting can not be used here**. It is rather a calories restriction.
9.	don't think you can specify one need to put a range of possibilities in the definition
10.	25% of energy needs for weight maintenance
**Experts on alternate-day fasting were asked for their opinion via email:**	
Comments:	"Zero-calorie alternate day fasting" - fast days with water or energy free beverages only; "Modified alternate day fasting" - fast days with **500-600 kcal**<br>I would suggest using these terms since they coincide with terminology used in most studies.<br>Historically and currently the term fasting is used for water only fasting or low energy diets which could be **5-600 kcal** or other low kcal; I would personally prefer to say alternate day calorie / energy restriction as not sure what a modified fast actually means. The problem with all these definitions is that we need to acknowledge a body of literature which has used terminology like alternate day modified fasting people will need to use all these terms for lit searches.<br>Alternate day fasting can be either water only or low calorie and this needs to be described.

3. PERIODIC FASTING

The following definition of **periodic fasting** reached a consensus of **70.59 %** already in the **first** round of the survey.

> ***Periodic fasting (PF)*** *refers to any fasting regimen that is repeated at regular intervals (periods), such as every day, every week, or every several months.*

(According to this definition, periodic fasting would include intermittent fasting regimens.)

However, several changes for this definition were suggested: specifying the duration of the fasting period and excluding the interval "every day". Therefore, we offered an additional definition in the second round:

> ***Periodic fasting (PF)*** *refers to any fasting regimen lasting ≥ 48 hours that is repeated at regular intervals (periods), such as ~~every day,~~ every several weeks or ~~every several~~ months.*

In this second round, no clear favorite emerged. Therefore, it was decided to include the term in the live discussion instead of presenting it again in the third survey.

QUESTIONS TO BE DISCUSSED:

- How should we define **periodic fasting?**
- Should the definition include **intermittent fasting regimens**?
- How long should the **minimum fasting period** be?

Please also take note of the definitions of "**periodic**" from the Cambridge, Oxford and Merriam Webster`s dictionaries.

"PERIODIC" according to dictionaries:

	Cambridge	Merriam-Webster	Oxford Dictionary of English
adjective	happening repeatedly over a period of time;<br>*American Cambridge:*<br>(=repeated event) SCIENCE - happening repeatedly in a fixed pattern;<br>(=time) happening repeatedly although not necessarily frequently;<br>PHYSICS - A pattern or other event is periodic if it repeats at periods of time that are equal	occurring or recurring at regular intervals; occurring repeatedly from time to time;<br>consisting of or containing a series of repeated stages, processes, or digits: CYCLIC	Appearing or occurring at intervals

**First round**	
**PERIODIC FASTING (PF)**	**Periodic fasting (PF)** refers to any fasting regimen that is repeated at regular intervals (periods), such as every day, every week, or every several months.<br>► **70.59 %** of the panel experts voted in favour of this definition.<br>► **2.94 %** of the panel experts voted neutral.<br>► **26.47 %** of the panel experts selected "disagree / strongly disagree".
**STRONGLY AGREE: Further thoughts on given definition / Explanation of choice**	
1.	It would be helpful to have a flow chart demonstrating the relationship between different fasting terms (I.e., which terms are grouped under other terms). I wonder if this would be possible in the next round of review to help with the big-picture of these terms.
2.	should also include a definition of allowance of water/beverage consumption.
**AGREE: Suggested modifications / alternative definition**	
3.	This falls under the definition of intermittent fasting
4.	I am not sure about several month, maybe exclude
**NEUTRAL: Suggested modifications / alternative definition**	
5.	I would use periodic fasting only for repeated fasting regimes when each fasting period comprises of at least 4 days
**DISAGREE: Suggested modifications / alternative definition**	
6.	Every other day, every other week, etc.
7.	To me periodic fasting is fasting for ≳ 3 days at regular intervals i.e. once every few weeks of months
8.	I think of periodic fasting as being of lower frequency as well as being repeated at regular intervals, so once every few weeks, month, or several months. I consider every day or once or more per week to be intermittent fasting.
9.	I would add the requirement that the fasting have a minimum length of time, such as 24 hours, to distinguish it from TRE. Second, I think it should refer to water-only fasting regimens. I strongly think we should implement a 24-hour minimum

	water-only fast. I would define it as "*Periodic fasting (PF) refers to any 24-hour or longer water-only fasting regimen that is repeated at regular intervals (periods), such as every day, every week or every several months.*" For example, periodic fasting could include fasting 1 day per week, alternate-day fasting, etc. Regardless, we need terminology for intermittent fasting regimens that involve water-only fasting for at least 24 hours at a time. I propose that the term be "periodic fasting." However, if you use the term periodic fasting to refer to something else, we still need a term to describe periodically water-fasting for at least 24 hours at a time.
10.	suggest keep this for periods of > 5 days and weeks on week off so can use the term intermittent fasting for 1-2 days / week
**STRONGLY DISAGREE: Suggested modifications / alternative definition**	
13.	periodic fasting should be limited to when you fast 2-5 days or more in fortnightly or monthly cycle
14.	The FMD diet constitutes as periodic fasting. I would change the definition to: *Periodic fasting (PF) refers to any fasting regimen that is repeated at regular intervals (periods), such as every several weeks or months.* Fasting that is repeated every other day is termed intermittent fasting.
15.	This is a Valter Longo term and should apply to a monthly fasting pattern only.
**Second round**	
**PERIODIC FASTING (PF)**	**A1: Periodic fasting (PF)** refers to any fasting regimen that is repeated at regular intervals (periods), such as every day, every week, or every several months.<br>(According to this definition, periodic fasting would include intermittent fasting regimens.)<br>**A2: Periodic fasting (PF)** refers to any fasting regimen lasting ≥ 48 hours that is repeated at regular intervals (periods), such as ~~every day,~~ every several weeks or ~~every several~~ months.<br>**Distribution of votes:**<br>A1: ▶ **51.52 %** of the panel experts voted in favour of this definition.<br>A2: ▶ **42.42 %** of the panel experts voted in favour of this definition.<br>A3 (neither of the above): ▶ **6.06 %** of the panel experts voted "neither of the above".
**I agree with A2: modified definition**	
1.	If we go with definition A2, then we need a term for intermittent fasting approaches such as fasting one day per week.
**Neither of the above (I disagree with both definitions)**	
2.	*Periodic fasting (PF) refers to any fasting regimen that is repeated at regular intervals (periods), such as every several weeks or months.*
3.	we are confusing intermittent fasting with periodic fasting. Periodic fasting should refer to a fasting which **is repeated less than once every 2-4 weeks** and which **could be at regular intervals or not**. For example **someone could do periodic fasting once every year or every 3 months** or **even month** or do it **every month but then change to once in a while when needed**

4. DURATIONS FOR SHORT- & LONG-TERM / PROLONGED FASTING

No consensus was reached on the durations for **short-** and **long-term / prolonged fasting.**

QUESTIONS TO BE DISCUSSED:

- What should be the durations for **short-term** and **prolonged / long-term fasting**?
- If the selected durations for **short-term-** and **long-term fasting** are not complementary, it has been suggested in the last rounds that a definition for an **intermediate duration fasting** regimen be established. However, such a fasting regimen has not been used in the literature to date. Do you still think it would be helpful?

Based on the lack of consensus and the comments received, we propose the following definitions:

Short-term fasting (STF) refers to a fluid-only fasting regimen with a duration of 1-3 days.

Prolonged fasting (PF), also called **long-term fasting (LTF)**, refers to any fluid-only fasting regimen lasting ≥ 4 consecutive days.

First round – SHORT-TERM FASTING	
**SHORT-TERM FASTING (STF)**	**Short-term fasting (STF)**, also called short-term caloric restriction, or, when referring to animals, short-term starvation (STS), is any fasting regimen with a duration of 48 to 72 hours.<br>▶ **50.00 %** of the panel experts voted in favour of this definition.<br>▶ **8.82 %** of the panel experts voted neutral.<br>▶ **35.29 %** of the panel experts selected "disagree / strongly disagree".<br>▶ **5.88 %** of the panel experts voted that this definition is irrelevant.

**AGREE: Suggested modifications / alternative definition**	
1.	So how do we call 4-5 day fasting?
**NEUTRAL: Suggested modifications / alternative definition**	
2.	I recommend removing the phrase "also called short-term caloric restriction." To me, this would add confusion to the definition. The range of 48 to 72 hours seems fairly small. As mentioned in the next comment box, I think the range of days for short-term fasting and long-term fasting should include all possible durations. With the current definition, the periods from 3 to 5 days are undefined (I.e., neither short-term nor long-term fasting). So, either the duration for short-term fasting should be increased past 72 hours, the duration for long-term fasting should be shortened to >3 days, or there should be another category (intermediate duration fasting, or something similar).
**DISAGREE: Suggested modifications / alternative definition**	
3.	The timeframe is much shorter from 24-48 hours
4.	Time should be 24-72 hours
5.	I would extend the timeframe to 1-5 days
6.	I do not agree that STF/STS should be called short-term CR. STCR seems to be more appropriate for short periods of moderate caloric consumption vs. a fast /
7.	is this part "also called short-term caloric restriction" necessary? As it can be a fasting from a type of nutrient without caloric restriction.
8.	I consider fasting for 8 hours to 48 hours to also be short-term fasting. I could agree if it were 16 hours to 72 hours.
9.	I think the definition needs to be extended to 4 days and should pertain only to water-only fasting. I also don't like calling it short-term caloric restriction. That means something else to me, as caloric restriction is different from fasting. Here is what I recommend instead: "*Short-term fasting (STF) is any water-only fasting regimen that lasts from 48 hours to 4 days.*"
10.	It should be >=24, not 48 hours.
11.	24-48 heure
**STRONGLY DISAGREE: Suggested modifications / alternative definition**	
12.	Acute fasting
**IRRELEVANT DEFINITION: Further thoughts on given definition / Explanation of choice**	
13.	these sound like v different things
14.	Short-term starvation is a misnomer unless you are dealing with a depleted subject.
**Second round**	
**SHORT-TERM FASTING (STF)**	Original wording: **Short-term fasting (STF)**, also called short-term caloric restriction, or, when referring to animals, short-term starvation (STS), is any fasting regimen with a duration of 48 to 72 hours.<br>Various **durations** for short-term fasting have been proposed in the last round. Please select one of the following durations to complete the modified definition of **short-term fasting** below:<br>**Short-term fasting (STF)** refers to a fluid-only fasting regimen with a duration of:<br>• 16 to 72 hours<br>• 24 to 48 hours<br>• 48 to 72 hours<br>• 1- 4 days<br>• 1 to 5 days<br>**Distribution of votes:**

Duration	Votes in %
16-72 hours (1-3 days)	30.30
24-48 hours (1-2 days)	21.21
48-72 hours (2-3 days)	27.27
24-96 hours (1-4 days)	15.15
24-120 hours (1-5 days)	6.06

**16 to 72 hours - comments**	
1.	I consider that 72h is too long for a total fast. It should last less than 24hours.
**24 to 48 hours - comments**	
2.	Does it need to be 24? Why not 18? Or other… what is the physiological basis for 24h, nefa, ketones are up after 18…
3.	This to me seems quite arbitrary…
**48 to 72 hours - comments**	
4.	again we need to have a species specific discussion and know what else will be used to define other fasting periods so this can be formalized

1 to 4 days - comments	
5.	My first choice would be 2-4 days. My second choice is 1-4 days. don't feel too strongly about this, other than I don't think that less than 24 hours should be called short-term fasting.
6.	I don't understand when this would be used. Assume this is not replacing intermittent fasting *Short-term fasting (STF) refers to any fasting regimen (total fast or calorie restricted fast) with a duration of:* think it is difficult to define what is short and long based on ketones as the level of ketone formation will depend on whether it is a period of total fast or CR
7.	I would define short term fasting as **1-3 days** actually, since I believe ketones bodies more or less plateau after 3 days. Long term could then be defined as ≥ 4 days.

**First round – LONG-TERM FASTING**	
**PROLONGED FASTING (PF)**	**Prolonged fasting (PF)**, also called **long-term fasting (LTF)**, refers to any fasting regimen lasting ≥ 5 days.<br>► **67.65 %** of the panel experts voted in favour of this definition.<br>► **14.71 %** of the panel experts voted neutral.<br>► **14.71 %** of the panel experts selected "disagree / strongly disagree".<br>► **2.94 %** of the panel experts voted that this definition is irrelevant.
**STRONGLY AGREE: Further thoughts on given definition / Explanation of choice**	
1.	I agree with the definitions of short-term fasting and prolonged fasting. However, these definitions do not saturate all the possible fasting choices, because fasting between 72 and 120 hours is basically excluded from these definitions. For such cases, a new category, such as "intermediate fasting", could be used.
**AGREE: Suggested modifications / alternative definition**	
2.	I think the range of days for short-term fasting and long-term fasting should include all possible durations. With the current definition, the periods from 3 to 5 days are undefined (I.e., neither short-term nor long-term fasting). So, either the duration for short-term fasting should be increased past 72 hours, the duration for long-term fasting should be shortened to >3 days, or there should be another category (intermediate duration fasting, or something similar).
3.	I would recommend >4 days
4.	I would prefer a fasting regimen lasting > 7 days, although a regimen of > or = 5 days should better fit in todays circumstances of life.
**NEUTRAL: Suggested modifications / alternative definition**	
5.	Why 5 days? This seems an arbitrary number with no scientific rationale
6.	While the adaptations to fasting may be prominent by 5 days, for some patients, a 3-4 day fast may qualify as prolonged.
**DISAGREE: Suggested modifications / alternative definition**	
7.	The timeframe is much shorter from 2-5 days
8.	Look at Keton bodies; They are already high in most humans after 3 days.
9.	>72 hours
10.	if short term is up to 72 hours, long term should be >72 hours
11.	I would probably say > 3 or 4 days
**IRRELEVANT DEFINITION: Further thoughts on given definition / Explanation of choice**	
12.	if fasting can include a low cal diet-or any diet restriction can include any diet if want to say total fast that would be different
**Second round**	
**PROLONGED FASTING (PF)**	Original wording: **Prolonged fasting (PF)**, also called **long-term fasting (LTF)**, refers to any fasting regimen lasting ≥ 5 days.<br>Various **durations** for prolonged / long-term fasting have been proposed in the last round.<br>**Prolonged fasting (PF)**, also called **long-term fasting (LTF)**, refers to any fluid-only fasting regimen lasting<br>• ≥ 3 consecutive days.<br>• ≥ 4 consecutive days.<br>• ≥ 5 consecutive days.<br>• ≥ 7 consecutive days.<br>**Distribution of votes:**

Duration	Votes in %
≥ 3 consecutive days	40.63
≥ 4 consecutive days	28.13
≥ 5 consecutive days	28.13
≥ 7 consecutive days	3.13

≥ 4 days – Comments	
1.	**I think intermittent fasting should be 12 to 48 hours and prolonged fasting 48 or more hours.**
2.	*long -term fasting (STF) refers to any fasting regimen (total fast or calorie restricted fast) with a duration of:* think it is difficult to define what is short and long based on ketones as the level of ketone formation will depend on whether it is a period of total fast or CR
3.	I would define short-term fasting as 1-3 days actually, since I believe ketones bodies more or less plateau after 3 days. Long term could then be defined as ≥ 4 days.
**≥ 5 days – Comments**	
4.	Dito – This to me seems quite arbitrary...

5. INTERMITTENT ENERGY RESTRICTION - INTERMITTENT FASTING - TIME-RESTRICTED EATING

5.1 INTERMITTENT ENERGY RESTRICTION

At the end of the first survey, we asked you if there were any other terms that you considered important to this consensus process. **Intermittent energy restriction** was one of these requested terms and was therefore included in the second round of the survey.

The following definition reached a consensus of **81.26 %** in the **second** survey:

> ***Intermittent Energy Restriction (IER)*** *includes periods of caloric restriction alternating with periods of ad libitum eating. As such, IER includes fasting regimens like intermittent fasting (IF) and time-restricted eating (TRE).*

However, some participants pointed out that **TRE** should not be part of the definition of **IER**. Therefore, we offered an additional definition in the **third** round:

> ***Intermittent Energy Restriction (IER)*** *includes periods of caloric restriction alternating with periods of ad libitum energy intake.*

In this third round, no clear favorite emerged. Therefore, it was decided to include the term in the live discussion.

QUESTION TO BE DISCUSSED:

- Should **Intermittent Fasting** and **Time-Restricted Eating** be classified under **Intermittent Energy Restriction**?

Please also take note of the definitions of "**intermittent**" from the Cambridge, Oxford and Merriam Webster`s dictionaries.

"Intermittent" according to dictionaries:

	Cambridge	Merriam-Webster	Oxford Dictionary of English
adjective	not happening regularly or continuously; stopping and starting repeatedly or with periods in between<br><br>*American Cambridge:*<br>not happening regularly or continuously;<br>stopping and starting repeatedly or with long periods in between	Coming and going at intervals;<br>not continuous;<br>also: occasional	occurring at irregular intervals; not continuous or steady

**Second round – INTERMITTENT ENERGY RESTRICTION** (term was included in the second round only)	
**INTERMITTENT ENERGY RESTRICTION (IER)**	**Intermittent Energy Restriction (IER)** includes periods of caloric restriction alternating with periods of *ad libitum* eating. As such, IER includes fasting regimens like intermittent fasting (IF) and time-restricted eating (TRE).<br>► **81.26 %** of the panel experts voted in favour of this definition.<br>► **15.63 %** of the panel experts selected "disagree / strongly disagree".<br>► **3.13 %** of the panel experts voted that this definition is irrelevant.
**STRONGLY AGREE: Suggested modifications / alternative definition**	

1.	maybe include periodic fasting as well
**AGREE: Suggested modifications / alternative definition**	
2.	TRE is a form of intermittent fasting because the fasting in TRE occurs intermittently (the intermittent occurrence is simply on a daily basis rather than a weekly or monthly basis).
**DISAGREE: Suggested modifications / alternative definition**	
3.	I think it is better to continue to call it intermittent fasting, but it needs to be defined, and maybe IER can be an additional but different term
4.	*Intermittent Energy Restriction (IER) includes periods of caloric restriction* ***sufficient to induce ketosis*** *alternating with periods of ad libitum energy intake. As such, IER includes fasting regimens like intermittent fasting (IF) and time-restricted eating (TRE).*
5.	*Intermittent Energy Restriction (IER) includes periods of caloric restriction alternating with periods of ad libitum energy intake. As such, IER includes fasting regimens like* ~~**intermittent fasting (IF) and**~~ *time-restricted eating (TRE).*
**STRONGLY DISAGREE: Suggested modifications / alternative definition**	
6.	Time-restricted eating (TRE) is definitely not a form of intermittent energy restriction. To me, IER means eating a low-calorie diet some number of days per week or per month. I would change the definition to: "*Intermittent Energy Restriction (IER) includes periods of caloric restriction alternating with periods of ad libitum* ~~**eating**~~ ***energy intake***. ~~***As such, IER includes fasting regimens like intermittent fasting (IF) and time-restricted eating (TRE)***~~*."*
7.	TRE needs to have a separate definition to IER. Many IER regimens will advise a healthy diet on the non -restricted days – also ? **use the term energy throughout the definitions and not calorie as they seem to be used interchangeably.** *Intermittent Energy Restriction (IER) or intermittent fasting includes periods of caloric restriction alternating with periods of ad libitum* ~~**eating**~~ ***energy intake, either normal diet or a different prescribed diet (i.e. healthy eating , med diet).***
**Third round**	
**INTERMITTENT ENERGY RESTRICTION (IER)**	Although consensus was reached for this definition in the last round, changes were proposed. Based on the comments received, we decided to offer two options.<br>**A1 (initial wording): Intermittent Energy Restriction (IER)** includes periods of caloric restriction alternating with periods of *ad libitum* eating. As such, IER includes fasting regimens like intermittent fasting (IF) and time-restricted eating (TRE).<br>**A2: Intermittent Energy Restriction (IER)** includes periods of caloric restriction alternating with periods of ad libitum energy intake.<br>**Distribution of votes:**<br>A1: ► **56.67 %** of the panel experts voted in favour of this definition.<br>A2: ► **43.33 %** of the panel experts voted in favour of this definition.
**I agree with A2 – comments:**	
1.	I disagree with considering IER, IF and TRE as equivalent terms.<br>**IF an a more extreme version of IER**, and in this respect it **can be incorpotared within IER.** However, **they cannot be considered as equivalent terms**, and a threshold of allowed calorie intake should be fixed to differentiate these two terms. IF and TRE can be considered as equivalent, provided that a minimum duration of fasting is specified for both approaches
2.	I would define this along the same lines as intermittent fasting (see further)
3.	I appreciate how straightforward A2 is, which is why it received my vote. However, this definition may leave it up to the reader **if they conceptualize intermittent fasting and time-restricted eating as programs that meet this definition (rather than explicitly stating that they do meet the definition, as in A1**). This could be fine, but I am not sure if that is consistent with the **purpose of the document**, which I think is **to reduce some of the ambiguity and confusion** regarding these terms.
4.	think time restricted feeding should have its own definition to distinguish it from IER
5.	in the term 'TRE' there is not the term 'intermittent'

5.2 INTERMITTENT FASTING

The following definition of **intermittent fasting** reached a consensus of **88.24 %** already in the **first** round of the survey.

> ***Intermittent fasting (IF)*** *refers to repetitive fasting periods of up to 48 h. IF includes fasting regimens of 1 day per week (6:1), 2 separate or consecutive days per week (5:2), alternate day fasting (ADF) and time-restricted eating (TRE).*

However, some participants voted to exclude **TRE** from this definition. Therefore, we offered an additional definition in the second round:

> ***Intermittent fasting (IF)*** *refers to repetitive fasting periods lasting up to 48 h each. IF includes fasting regimens of 1 fasting day per week ~~(6:1),~~ 2 separate or consecutive fasting days per week ~~(5:2)~~ and alternate-day fasting (ADF). ~~and time-restricted eating (TRE).~~*

In this second round, no clear favorite emerged, hence we proposed the following definition in the **third** round that reached a consensus of **86.67 %.**

> ***Intermittent fasting (IF)*** *refers to repetitive fasting periods lasting up to 48 h each. IF includes fasting regimens of 1 fasting day per week, 2 separate or consecutive fasting days per week, alternate-day fasting (ADF), and time-restricted eating (TRE).*

A few participants still voted to exclude TRE from the definition.

QUESTION TO BE DISCUSSED:

❖ Should **Time-Restricted Eating** be considered a sub-category of **Intermittent Fasting**?

**First round – INTERMITTENT FASTING**	
**INTERMITTENT FASTING (IF)**	**Intermittent fasting (IF)** refers to repetitive fasting periods of up to 48 h. IF includes fasting regimens of 1 day per week (6:1), 2 separate or consecutive days per week (5:2), alternate day fasting (ADF) and time-restricted eating (TRE).<br>**Distribution of votes:**<br>► **88.24 %** of the panel experts voted in favour of this definition.<br>► **11.76 %** of the panel experts selected "disagree / strongly disagree".
**STRONGLY AGREE: Further thoughts on given definition / Explanation of choice**	
1.	TRE should only be used for cyclical fasting within a day not IF. In my opinion IF has a very different mechanism of action
2.	The term "5:2" is unfortunately a term that should not be used in academic literature because it promotes specific products. The term "5:2 diet" is the copyrighted name of a diet and the title of a series of weight loss books (https://kate-harrison.com/52-health) that provides income to specific individuals. Also, the "5:2<br>fast diet" and the term "5:2" as it applies to fasting (including the "5:2 intermittent fasting diet") are not only copyrighted but also trademarked names used as identifiers of a diet described in a series of weight loss books entitled the "Fast Diet" (https://thefastdiet.co.uk/). The first of book for each of these 5:2 weight loss diets were published in 2013 (within a month of each other). The term "5:2 diet" is used commonly today in academic papers but, because of the origination of the term and the copyrighting/trademarking, it promotes these specific products whose trade names utilize the 5:2 terminology. These are like the trade names of medications. In academic literature, generic names should be used so that a manuscript does not promote these individual products or sets of products. Instead of referring to the 5:2 diet, a generic term like "twice-weekly fasting," "twice-per-week fast," or a similar term should be used as a generic term like a generic drug would be referred to.
3.	water should be allowed
**AGREE: Suggested modifications / alternative definition**	
4.	up to 24h would be better
5.	I think TRE deserves to be a bit more separate from IF.
6.	I would add a lower limit and also restrict the definition to water-only fasting. Here is my alternative definition: "*Intermittent fasting (IF) refers to water-only fasting regimes that involve periodically fasting for 14-48 hours at a time. IF includes fasting regimens of 1 day per week (6:1), 2 separate or consecutive days per week (5:2), alternate-day fasting (ADF), and time-restricted eating (TRE).*"
7.	The definition needs to incorporate "some widely practiced forms of religious fasting such as Ramadan fasting".
8.	The way we use this term in practice is to recommend 12-16 hours of fasting every day. (limit the feeding window to 8 hours for those hoping to lose weight and 12 hours for those needing higher caloric intake and in all cases avoiding eating 3-4 hours before sleep.
**DISAGREE: Suggested modifications / alternative definition**	
9.	I believe that the different definitions of fasting regimens should be based not only on the duration of fasting, type of foods/beverages allowed/not allowed and time intervals between fasting periods, but they should also take into account the expected biological modifications induced by these regimens. In this case, I would restrict the definition of *Intermittent fasting (IF)* to "*repetitive fasting periods for up to 48 hours. IF includes fasting regimens of 1 day per week (6:1) or 2 separate or consecutive days per week (5:2)*", which have higher chances to result in specific metabolic effects, such as a reduction of blood glucose and growth factor levels. Alternate-day fasting (ADF) could fit this definition, but it cannot be applied to intermittent fasting, because it would necessarily imply at least a total of 72 hours of fasting per week (e.g., Monday, Wednesday, Friday).
10.	*Intermittend fasting refers to repetitive fasting periods for 24-72 hours at an interval of max 1 week*

11.	• Ramadan fasting consists on fasting everyday for +/- 14 hours over 24 hours during one month. It can also be considered as an intermittent fasting.<br>• How is TRE defined?
**STRONGLY DISAGREE: Suggested modifications / alternative definition**	
12.	TRE should be classified apart from intermittent fasting.
**Second round**	
**INTERMITTENT FASTING (IF)**	**A1: Intermittent fasting (IF)** refers to repetitive fasting periods of up to 48 hours. IF includes fasting regimens of 1 day per week (6:1), 2 separate or consecutive days per week (5:2), alternate-day fasting (ADF) and time-restricted eating (TRE).<br><br>**A2: Intermittent fasting (IF)** refers to repetitive fasting periods lasting up to 48 h each. IF includes fasting regimens of 1 fasting day per week ~~(6:1),~~ 2 separate or consecutive fasting days per week ~~(5:2)~~ and alternate-day fasting (ADF).~~and time-restricted eating (TRE).~~<br>**Distribution of votes:**<br>A1: ▶ **40.63 %** of the panel experts voted in favour of this definition.<br>A2: ▶ **50.00 %** of the panel experts voted in favour of this definition.<br>A3 (neither of the above): ▶ **9.38 %** of the panel experts voted "neither of the above".
**I agree with A2: modified definition**	
1.	TRE has a different mechanism of action (restoring circadian rhythms) than IF (body uses ketones for energy) and should therefore not be lumped together.
2.	time restricted eating should have its own definition
**Neither of the above (I disagree with both definitions)**	
3.	*Intermittent fasting (IF) refers to repetitive fasting periods lasting up to 48 h each. IF includes fasting regimens of 1 fasting day per week, 2 separate or consecutive fasting days per week, alternate day fasting (ADF), and time-restricted eating (TRE).*
4.	I strongly believe that TRE should count as intermittent fasting.<br>My first choice for wording would be a combination of A1 and A2: *"Intermittent fasting (IF) refers to repetitive fasting periods lasting up to 48 h each. IF includes fasting regimens of 1 fasting day per week (6:1 diet), 2 separate or consecutive fasting days per week (5:2 diet), alternate day fasting (ADF), and time-restricted eating (TRE)"*
5.	-IF refers to repetitive periods of fasting (without indicating the duration)<br>- fasting day: does it mean 24 hours of fasting.<br>For example: Ramadan fasting is an intermittent fasting and fasting lasts up to 16 hours /24 (diurnal fasting) and is repeated every day during one month (Ramadan). Another type of fasting in Islam is diurnal fasting during two days a week (Mondays and Thursdays) during the whole year
**Third round**	
**INTERMITTENT FASTING (IF)**	As no consensus was reached in the last round, we propose to subsume "**time-restricted eating**" under the term "**intermittent fasting**" since TRE is found under IF in common mesh terms This is a stronger argument for us than the fact that TRE is slightly different from other IF regimens because of its effect on circadian rhythms rather than ketone body metabolism. Therefore, we proposed a combination of definitions A1 and A2 from round two, which was suggested by one of the participants:<br>**A3: Intermittent fasting (IF)** refers to repetitive fasting periods lasting up to 48 h each. IF includes fasting regimens of 1 fasting day per week, 2 separate or consecutive fasting days per week, alternate-day fasting (ADF), and time-restricted eating (TRE).<br>**Distribution of votes:**<br>▶ **86.67 %** of the panel experts voted in favour of this definition.<br>▶ **13.33 %** of the panel experts selected "disagree / strongly disagree".
**STRONGLY AGREE: further thoughts**	
1.	I disagree with implications of the statement: TRE has a different mechanism of action (restoring circadian rhythms) than IF (body uses ketones for energy) and should therefore not be lumped together. (1) TRE has multiple mechanisms, not just restoring circadian rhythms (too narrow). (2) This statement assumes that the most important feature of fasting is the production of ketones. History, IF has not been defined by the production of ketones. Historically, definitions have focused on the length of the fasting period, not the underlying mechanisms (which are still being understood).
**DISAGREE: suggested modifications / alternative definition**	
2.	*Intermittent fasting refers to repetitive fasting periods applied **on a per week basis**, alternated with periods of ad libitum food intake.*
3.	would have preferred to keep them separate BUT if you do combine them, then at least give a brief description of TRE in the definition "(limiting food intake to 6-12 hours per 24 hour period)"

4.	*Intermittent fasting (IF) refers to repetitive fasting periods. IF includes diurnal fasting regimens of 1 fasting day **per week**, 2 separate or consecutive fasting days **per week**, successive- or alternate-day fasting.*
**STRONGLY DISAGREE: suggested modifications / alternative definition**	
5.	we need to describe the different patterns of the diet time restricted feeding is very different to intermittent fasting and should have its own definition.

5.3 TIME-RESTRICTED EATING

The following definition of **time-restricted eating** reached a consensus of **91.18 %** already in the **first** round of the survey.

> ***Time-restricted eating (TRE)** is a dietary regimen in which food intake is restricted to a specific period of time (usually 4 to 10 hours) during the day, resulting in a daily fasting window of 14 to 20 hours. There is no explicit limit on energy intake during eating or feeding hours.*
> (Note that "*or, when referring to animals, **time-restricted feeding (TRF)**,*" was removed as it was decided to focus on fasting terms in humans.)

However, a few participants wished to expand the fasting window and to include the **one-meal a day diet (OMAD)** under the definition of **TRE**. Therefore, we offered additional definitions to choose from. In the past two rounds, no clear favorite emerged.

QUESTIONS TO BE DISCUSSED:

- How long should the fasting window be for **TRE**?
- Should **OMAD** be included in the definition of **TRE**?

**First round – TIME RESTRICTED EATING**	
**TIME RESTRICTED EATING (TRE):**	**Time-restricted eating (TRE)** or, when referring to animals, **time-restricted feeding (TRF)**, is a dietary regimen in which food intake is restricted to a specific period of time (usually 4 to 10 hours) during the day, resulting in a daily fasting window of 14 to 20 hours. There is no explicit limit on energy intake during eating or feeding hours.<br>**Distribution of votes:**<br>► **91.18 %** of the panel experts voted in favour of this definition.<br>► **5.88 %** of the panel experts voted neutral.<br>► **2.94 %** of the panel experts selected "disagree / strongly disagree".
**STRONGLY AGREE: Further thoughts on given definition / Explanation of choice**	
1.	could also have a definition where TRF can also be energy restricted
**AGREE: Suggested modifications / alternative definition**	
2.	I think the durations needs to be expanded. The most dramatic form of TRE, in my opinion, is one meal a day (OMAD), in which a single meal is consumed. In this case, the day could be viewed as 23 hours of fasting and 1 hour of feeding. Based on this, I recommend using the range of 1 to 10 hours of eating, resulting in a daily fasting window of 14 to 23 hours. I also recommend replacing "explicit" with "inherent."
3.	during the "fasting" time, non-calorie drinks are generally permitted. I also prefer time-restricted feeding for humans rather than time-restricted eating.
4.	It is the same as "fasting" when water and all types of nutrients are concerned .
5.	the definition needs to mention beverages. Here are my slight modifications to the definition. "*Time-restricted eating (TRE) is a dietary regimen in which the consumption of food and calorie-containing beverages is restricted to a specific period of time. In humans, TRE is defined as restricting food intake to a 10-hour daily period or less, followed by at least 14 hours per day of water-only fasting.*
6.	I would extend the fasting time window to 12-22 hours
**DISAGREE: Suggested modifications / alternative definition**	
7.	*Time-restricted eating (TRE) is a dietary regimen in which food intake is restricted to a specific period of time (usually 4 to 10 hours) during the day, resulting in a daily fasting window of 14 to 20 hours. The eating or feeding hours should be in accordance with each species' biorhythm and the energy intake regulated.*
**Second round**	
**TIME RESTRICTED EATING (TRE)**	**A1: Time-restricted eating (TRE)** or, when referring to animals, **time-restricted feeding (TRF)**, is a dietary regimen in which food intake is restricted to a specific period of time (usually 4 to 10 hours) during the day, resulting in a daily fasting window of 14 to 20 hours. There is no explicit limit on energy intake during eating or feeding hours.

	**A2: Time-restricted eating (TRE)** ~~or, when referring to animals, **time-restricted feeding (TRF)**,~~ is a dietary regimen in which food intake and the consumption of caloric beverages is restricted to a specific period of time (usually 4 to 10 hours) during the day, resulting in a daily fasting window of 14 to 20 hours. There is no explicit limit on energy intake during eating ~~or feeding~~ hours.<br>**A3: Time-restricted eating (TRE)** ~~or, when referring to animals, **time-restricted feeding (TRF)**,~~ is a dietary regimen in which food intake and the consumption of caloric beverages is restricted to a specific period of time (usually 1 to 12 hours) during the day, resulting in a daily fasting window of 12 to 23 hours. There is no explicit limit on energy intake during eating ~~or feeding~~ hours.<br>**Distribution of votes:**<br>A1: ▶ **18.75 %** of the panel experts voted in favour of this definition.<br>A2: ▶ **59.38 %** of the panel experts voted in favour of this definition.<br>A3: ▶ **15.63 %** of the panel experts voted in favour of this definition.<br>A4: ▶ **6.25 %** of the panel experts voted "neither of the above".
**I agree with A2: modified definition**	
1.	I suggest a small modification to definition A2: "*Time-restricted eating (TRE) is a dietary regimen in which food intake and the consumption of caloric beverages is restricted to a specific period of time (usually 1-10 hours) during the day, resulting in a daily fasting window of* ***at least 14 hours****. There is no explicit limit on energy intake during eating hours*."
**I agree with A3: modified definition**	
2.	although can also have overall cal restricted TRF diets
**Neither of the above (I disagree with the given definitions)**	
3.	I agree with A3 but the animal variation has to be included
4.	the literature supports definition 3 **BUT range of eating 6-12 hours**; there is no benefit to restricting less than 6 hours (reference available upon request)
**Third round**	
**TIME RESTRICTED EATING (TRE)**	As no clear favorite emerged in the last round, we offered a new definition (=A1) proposed by one of the experts and the definition with the majority of votes from the last round (A2) for re-evaluation.<br>**A1: Time-restricted eating (TRE)** is a dietary regimen in which food intake and the consumption of caloric beverages is restricted to a specific period of time (usually 1 to 10 hours) during the day, resulting in a daily fasting window of at least 14 hours. There is no explicit limit on energy intake during eating hours.*<br>*Note: Low frequency meal patterns such as the "**one meal a day diet (OMAD)**" can be regarded as part of TRE according to this definition.<br>**A2: Time-restricted eating (TRE)** is a dietary regimen in which food intake and the consumption of caloric beverages is restricted to a specific period of time (usually 4 to 10 hours) during the day, resulting in a daily fasting window of 14 to 20 hours. There is no explicit limit on energy intake during eating hours.<br>**Distribution of votes:**<br>A1: ▶ **53.33 %** of the panel experts voted in favour of this definition.<br>A2: ▶ **43.33 %** of the panel experts voted in favour of this definition.<br>A3: ▶ **3.33 %** of the panel experts voted "neither of the above".
**I agree with A1: new wording – comments**	
1.	As indicated by the note attached to the asterisk (for A1), inclusion of OMAD as part of TRE necessitates that the eating period could be as short as ~1 hour. I think this definition is preferable as **it is broader and encompasses virtually all TRE-style programs**, which I think **should be the goal of the definition**.
**I agree with A2: initial definition - comments**	
2.	what about the requirement to begin eating at least one hour after arising, and end at least 3 hours before going to sleep? Without that you don't have the full benefit of improving the circadian rhythm.
**Neither of the above - comments**	
3.	suggest TRF if > fasting period to the average i.e > 12 hours

6. CALORIC RESTRICTION

The following definition of **caloric restriction** reached a consensus of **88.24 %** in the **second** round of the survey.

> ***Caloric restriction (CR)*** *describes a reduction in energy intake below the total amount of calories that would be needed to maintain a person's current body weight, without causing malnutrition. CR may also be used to achieve a healthy body weight over time.*

However, no consensus was reached on the range of reduction in daily caloric intake.

Additionally, it was suggested that the term **continuous energy restriction** (CER) / **daily energy restriction** (DER) be subsumed under the term **caloric restriction.** Thus, the following sentence was added to the definition above and accepted during the third survey round:

> *If **CR** is done daily, it can also be referred to as **continuous energy restriction (CER)** or **daily energy restriction (DER).***

QUESTIONS TO BE DISCUSSED:

- Should a **specific amount / range of reduction** in daily caloric intake be specified in the definition of **caloric restriction**?
- If yes, what amount / range should be specified?

First round	
CALORIC RESTRICTION (CR)	**A1**: **Caloric restriction (CR)** describes a reduction in energy intake below ad libitum levels without malnutrition. (An individual's total energy intake during caloric restriction by this definition could still exceed his / her daily caloric requirements, as is the case with people with overweight and obesity.)<br>**A2**: **Caloric restriction (CR)** describes a reduction in energy intake below the total caloric intake that would be needed to maintain a healthy body weight, without causing malnutrition. (Total caloric intake here refers to the amount of calories required to maintain a person's "healthy" BMI – depending on their height and age.)<br>**Distribution of votes:**<br>A1: ►**41.18 %** of the panel experts voted in favour of this definition.<br>A2: ►**58.92 %** of the panel experts voted in favour of this definition.
**STRONGLY AGREE: Further thoughts on given definition / Explanation of choice**	
1.	**A1:** Caloric restriction is restriction on AL levels regardless of the persons starting bodyweight.
2.	**A1:** calorie restriction is the long term norm, but it really should be energy restriction for global acceptance. That being said I usually use CR as it has historical importance.
**AGREE: Suggested modifications / alternative definition**	
3.	**A2**: Globally i agree with the selected definition of calorie restriction ("a reduction in energy intake below the total caloric intake that would be needed to maintain a healthy body weight, without causing malnutrition"). To make this definition more complete, I suggest to include a functional parameter in the definition, and in particular to state that this "reduction of energy intake" should also result in measurable metabolic effects, including a reduction of blood glucose/growth factor levels and/or an increase in blood/urinary ketone bodies.
4.	**A1**: I see merits to both options provided in the previous question. While I see some potential difficulties with establishing someone's true ad libitum intake, I saw greater challenges with the "healthy body weight" component of the second definition (which is one reason why I chose the first). If the second definition is chosen by consensus, I may have further feedback, particularly a suggestion to revise "a healthy body weight" to another term like "current body weight" or "initial body weight."
5.	**A2:** The aim of CR is not only to maintain but also to achieve a healthy body weight.
6.	**A2:** again, malnutrition is on the Agenda, see above. Also, it might make sense to set a Level of caloric restriction, let's say Caloric restriction (CR) describes a reduction in average energy intake below 70% of the total caloric intake that would be needed to maintain a healthy body weight, without causing malnutrition. (Total caloric intake here refers to the amount of calories required to maintain a person's "healthy" BMI – depending on their height and age.) If you want to include IF and esp long-term fasting in one definition, which is charming, you cannot set a range as below. You avoid this by setting a lower level (water fasting means zero energy absolutely and relatively, no matter from where you start)
7.	I would remove the "healthy BMI" section. Just for weight maintenance is enough.
8:	There is no good/proper data from a sustained human clinical trial to fully define. The longest trial was the CALERIE study and participants showed benefits at low levels of sustained restriction (less than 14%)
9.	**A2:** BMI may not be an effective measurement of healthy weight.
**NEUTRAL: Suggested modifications / alternative definition**	
10.	**A2:** The problem is the term "healthy body weight without malnutrition". We do know hat long term caloric restriction often causes immune dysfunction (for example, those people often have to take antibiotics for months in order to get rid of infections).
11.	I am torn on whether CR should be defined relative to (A) maintaining one's current body weight versus (B) maintaining a healthy body weight. I think I lean towards the former and would instead define it as "Caloric restriction (CR) describes a reduction in energy intake below the total caloric intake that would be needed to maintain one's current body weight." Second, the group could also consider whether a minimum threshold needs to be achieved, such as restricting energy intake by 10% or more (relative to eucaloric requirements) is CR and restricting by less than 10% is not.

DISAGREE: Suggested modifications / alternative definition	
12.	**A1:** The definition of CR should not include reductions in caloric intake above that needed to maintain one's current body weight.
**CALORIC RESTRICTION (CR)**	What range of reduction in daily caloric intake should be included in defining **caloric restriction**?<br>10-25%; 15-40%; 20-40%; 30-40%; 20-50%, other<br>**Distribution of votes:**<br>A1: ▶ **8.82 %** of the panel experts voted in favour of 10-25 %.<br>A2: ▶ **29.41 %** of the panel experts voted in favour of 15-40 %.<br>A3: ▶ **8.82 %** of the panel experts voted in favour of 20-40 %.<br>A4: ▶ **0.00 %** of the panel experts voted in favour of 30-40 %.<br>A5: ▶ **11.76 %** of the panel experts voted in favour of 20-50 %.<br>A6: ▶ **41.18 %** of the panel experts voted in favour of "other" (see comments).
**Further thoughts / Explanation of choice**	
1.	**Other**: when we talk about humans there is no "one size fits all". Animal studies show 10% food restriction extends lifespan compared to ad libitum, and 60% also extends lifespan. But it is also context dependent ie there are sex and genetic background effects where CR doesn't "work" the same for everyone.
2.	**Other**: 10-40%: After 40% severe CR should be used.
3.	**Other**: 20-100%: depending on the duration (number of days) of calorie restriction and the time interval between subsequent calorie restriction cycles
4.	10-25%: I do not know if you want to discuss this but there is going evidence that extreme fasting/long term CR can give rise to viral infections
5.	20-50%: Worunter fällt unsere 800 kcal Diät, wenn Bedarf zB 2000 pro Tag?
6.	**Other**: 10-40%: I think it makes sense for the range to be quite broad so as to be inclusive of varying degrees of caloric restriction. A minimum of 10% seems reasonable, although some could argue for lower. I think the high end of the range is challenging since some individuals with extremely high ad libitum intakes could sustain a large % caloric restriction without malnutrition. I was undecided regarding 40 or 50% for the upper end.
7.	**Other**: > 20
8.	**Other**: None: The degree of caloric restriction should be decided individually depending on the treated person's needs and stamina (physical and psychologic)
9.	**Other**: None: The degree of caloric restriction should be decided individually, depending on the treated person's needs and psychologic and/or physical stamina.
10.	**Other**: 500 kcal
11.	**Other**: 10-50%
12.	15-40%: Caloric intake should be reduced down to the estimated resting metabolic rate. So, the magnitude is also dependent on the profession of the participant.
13.	**Other**: 10-50%: is there an official definition of CR?
14.	**Other**: Greater than Either 10% or 15% with the upper limit defined by the level that corresponds to major impairment in humans. – comment: I think the upper limit should be defined as that which corresponds to major health impairments. In the absence of clear evidence on what would constitute the upper limit in humans, I would prefer either 10-40% or 15-40%. The best people to ask are Eric Ravussin and those involved in the CALERIE trials in the United States. They should set the definition for the committee.
15.	**Other**: see above more than 30% - Here I would avoid daily. If you have alternate fasting let's say 5:2 you have to average one week, for some people that chose 10:4 for whatever reason, it's two weeks and so on. I would define the period of an IF method as an important measure very early in the discourse. E.g. the HELENA trial defined this very early and quite easy to understand comparability between the reduction groups in their M&M. By dividing the whole energy uptake in the period by the number of days you get the average daily reduction of an IF.
16.	**Other:** dont think you can specify – you can't specify would need to say a x% calorie restriction need to decide if want to use the correct terms of enegy restriction rather than the colloquial term calorie restriction
17.	**Other:** for humans 10-30%, for humans there are no strong data to define what is possible/feasible in the long-term. It would be important to start thinking on long vs short term uses of CR in humans for rodents it is becoming crystal clear that sex, diet, strain and age of onset should define the % of restriction tolerated......i would expect the same for higher mamals
**Second round**	
**CALORIC RESTRICTION (CR)**	Based on the votes in the last round, we decided to proceed with the **second definition** and modify it according to your suggested changes:<br>**Caloric restriction (CR)** describes a reduction* in energy intake below the total ~~caloric intake~~ amount of calories that would be needed to maintain a ~~healthy~~ person's current body weight, without causing malnutrition.~~(Total caloric intake here refers to the amount of calories required to maintain a person's "healthy" BMI – depending on their height and age.)~~ CR may also be used to achieve a healthy body weight over time.

	(*range of reduction is to be selected in the next question)<br>**Distribution of votes:**<br>▶ **87.88 %** of the panel experts voted in favour of this definition.<br>▶ **12.12 %** of the panel experts selected “disagree / strongly disagree”.

AGREE: Suggested modifications / alternative definition

1.	"CR may also be used to achieve a healthy body weight over time." doesn't need to be here in my opinion
2.	not sure the last addition is needed. Even if you drop 5% that’s great for health – this should not be the focus. Eg A bmi of 40 is almost never going to get to 25 without surgery or drugs.
3-	I would delete "CR may also be used to achieve a healthy body weight over time", but I don't feel strongly about this.
4.	The addition of the second sentence doesn't really make sense to me...
5.	CR definition, in animal models, doesn't have anything to do to maintain particular body weight. In fact, depending on the strain of animal used, the proportion of change in body weight post CR doesn't follow a linear change.
6.	I do not like the part 'without causing malnutrition'. It is a matter of time scale: if you fast for several days, you definitely undergo malnutrition for a defined interval. But the way you define the interval and/or add supplements, you hopefully are not malnurished in the end or refurnish declined supplies of any important substance fast.
7.	remove "heathy"

DISAGREE: Suggested modifications / alternative definition

8.	Unless physical exercise and total energy expenditure is increased, calorie restriction, especially if chronic, is inevitably associated with loss of body weight and BMI reduction, which should remain the range of normal body weight.
9.	second statement (in blue) is not relevant to the definition of CR. It simply says a reason one might want to calorie restrict. A definition of a term doesn't include the reason. eg: deoderant: a chemical to prevent body odor. Why a person would use it is not part of the definiton.
10.	I prefer the former definition: A2

STRONGLY DISAGREE: Suggested modifications / alternative definition

11.	calorie restriction in the field is viewed as a restriction that is species specific. For mice it is usually 20-40% for humans in most cases 10-25%. It is recognized that CR in humans does cause weight loss, and in fact the clinical studies suggest a heavy weight loss below normal, with many men reaching BMI 19 or so

**CALORIC RESTRICTION (CR)**	Which range of reduction in daily caloric intake should be included in the definition of **caloric restriction**? New options were added after the first round of the survey.<br>**Distribution of votes:**

Range in %	Votes in %
10-25	15.15
10-40	27.27
15-40	12.12
10-50	3.03
15-40	3.03
20-40	3.03
20-50	6.06
The range can’t be generalized	30.30
other	6.06

10-25% - Comments

1.	My answer refers to the case of chronic (daily) calorie restriction

10-50% - Comments

2.	I would define it as >=10% and leave it as that.
3.	I see the appeal of the option “The range can not be generalized...”, and I imagine many respondents may choose this. However, I think the definition will be more useful if it contains a range. This is why I elected to vote for 10-50%. I think 10-40% would also be appropriate, but I think a broader range better encompasses the variety of scenarios in which caloric restriction may be employed.

The range can’t be generalized – Comments

4.	If caloric restriction includes intermittent fasting, no amount of energy restriction can be specified in the definition of CR.
5.	The percent is a function of the reason for losing weight and the speed with which one wants to lose. Again, it is not part of the definition of CR. Arguably, a 1% reduction is also CR, albeit a very slow way to lose weight.
6.	I think, CR should be close to the estimated (better measured) resting metabolic rate.

Other – Comments

7. **See above** à calorie restriction in the field is viewed as a restriction that is species specific. For mice it is usually 20-40% for humans in most cases 10-25%. It is recognized that CR in humans does cause weight loss, and in fact the clinical studies suggest a heavy weight loss below normal, with many men reaching BMI 19 or so

8. **at least 30%** - if Buchunger fasting is to be included, this is about 80 to 90% restriction. If water fasting is to be included this is 100% restriction.

It seems reasonable to me to set an upper but no lower limit. Here In sugguste 30% reduction.

Third round

CALORIC RESTRICTION (CR)

Although consensus was reached for the definition in the last round, several changes were proposed and since consensus could not be reached on the range of reduction, the editors decided to adapt the above definition and offer two more options for the definition of CR according to the comments and the 7th evaluation rule.

In these newly proposed definitions, the term "**continuous energy restriction**" (CER) / "**daily energy restriction**" (DER) is subsumed under the term "**caloric restriction**", as the following comments on the definition of CER /DER were received:

Expert comments on CER /DER:

- To me, the percentage of energy restriction included in the definition of CER should match the range agreed upon for the definition of caloric restriction.I feel like this makes sense because CER/DER is essentially caloric restriction employed each day.
- because this [CER] defines the daily use of CR, I would suggest that the % reduction should be adjusted to the % range of reduction in daily caloric intake that has the majority vote
- we should just have 1 term (CR), adding more terms to say the same is only going to add to confusion

Note on all three definitions: Total caloric intake here refers to the amount of calories required to maintain a person's "healthy" BMI – depending on their height and age.

A1: Caloric restriction (CR) describes a reduction* in energy intake below the total amount of calories that would be needed to maintain a person's current body weight, without causing malnutrition. ~~CR may also be used to achieve a healthy body weight over time.~~ If **CR** is done daily, it can also be referred to as **continuous energy restriction (CER)** or **daily energy restriction (DER).**

*Note: amount of reduction is not specified in this definition.

Experts' comments as to why the amount of reduction should not be stated:

- on CR: The percentage is a function of the reason for losing weight and the speed with which one wants to lose [weight]. Again, it is not part of the definition of CR. Arguably, a 1% reduction is also CR, albeit a very slow way to lose weight.
- On CER: I don't think that continuous energy restriction necessarily has a certain amount of energy restriction assigned to it. It just describes that it is occurring at every meal across the day.
- On CER: I think the term should not include a percentage reduction of calories. Any reduction of calories (below the requirement for maintenance of bodyweight) on a daily basis can be defined as continuous or daily energy restriction.

A2: Caloric restriction (CR) describes a ≥ 10%* reduction in energy intake below the intake amount of calories that would be needed to maintain a person's current body weight, without causing malnutrition. ~~CR may also be used to achieve a healthy body weight over time.~~ If **CR** is done daily, it can also be referred to as **continuous energy restriction (CER)** or **daily energy restriction (DER).**

*Note: the editors have chosen "≥ 10%" to combine the range options previously offered.

A3: Caloric restriction (CR) describes a reduction in energy intake below the total amount of calories that would be needed to maintain a person's current body weight, without causing malnutrition. The degree of caloric restriction should be decided individually depending on the person's age, sex, current body weight, occupation, goal & planned duration of CR. If **CR** is done daily, it can also be referred to as **continuous energy restriction (CER)** or **daily energy restriction (DER).**

Distribution of votes:

A1: ► **33.33 %** of the panel experts voted in favour of 10-25 %.
A2: ► **13.33 %** of the panel experts voted in favour of 15-40 %.
A3: ► **53.33 %** of the panel experts voted in favour of 20-40 %.

I agree with A1 - comments

1. To me, the additional sentence of explanation provided in A3 ("The degree of caloric restriction should be decided individually depending on the person's age, sex, current body weight, occupation, goal & planned duration of CR.") could be included in the publication as additional explanation regarding the concept of caloric restriction but is not necessary to include in the initial definition itself. It seems to make the definition longer than necessary.

2.	○ Need to state that energy restriction is estimated according to their requirements for the day as this aligns with energy to maintain body weight. Time restricted feeding does not fit with this.<br>○ No need to state how energy requirements are estimated. The first statement saying below energy requirements would by definition use information on age, gender etc but no need to state. Not sure why need to say anything about daily calorie restriction
**I agree with A3 - comments**	
3.	instead of occupation I would put "degree of daily physical activity"
4.	A data-driven definition is more appropriate

Online Consensus Conference - Summary

Summary

Rule for the discussion

If there was **no consensus on the definition before the discussion**, a **consensus of ≥ 70% had to be reached** in the discussion to adopt the proposed definition. If there was **already consensus on the definition in the written surveys** but at **least two participants proposed the same changes**, these terms and proposed changes were discussed. Subsequently, if a **consensus of ≥ 50% was reached in the discussion** group, the definition (with or without the changes) was accepted.

---

*18 people participated in the online discussion - some were absent for some time during the session or had to leave early, hence there were only **15 participants** for some polls.*
Note: We had to change the order of the terms discussed in the online discussion from the agenda sent out prior to the discussion, otherwise a key expert on one of the terms would not have been able to participate in the discussion on that term. However, this document maintains the previous order to make it more logical.
*We have tried to transcribe everything that was said by all participants. However, please note that we could not always write down word for word what was said. Sometimes what was said was shortened or summarized by us. To our native English speakers: Please keep in mind that in this written document we only try to **reflect the participants' opinion partly verbatim**. However, since this was a live discussion and all participants are from different countries with different mother tongues, we did not make a point of correcting grammatical errors such as sentence structure, etc.*
*When you see square brackets [ ], it means that we added something in **our own words** to what was said by the speakers: Sometimes we completed incomplete sentences or added something if the participants did not fully express their thoughts. Even though these unspoken additions were certainly understandable for the experts present, we thought it was important to make the speaker's point of view clearer to readers.*

Guide to layout & colours in the following text

The text below is laid out as follows:

1. **DISCUSSED TERM**

   State of the definition BEFORE the live discussion

❖ Questions discussed in the workshop

Dialogues/comments on the questions of the group *in exchange with the moderator*

What was decided by the discussion group on the term / definition

---

THE TERMS THAT HAVE BEEN DISCUSSED

1. FASTING - TOTAL FAST - MODIFIED FASTING (MF)

1.1 FASTING

The following definition reached a consensus of **84.85%** in the **second** survey:

> ***Fasting*** *refers to a voluntary abstinence from some or all foods or foods and beverages for preventive, therapeutic, religious, cultural, or other reasons.*

However, some participants criticized this definition for allowing energy intake during fasting. Therefore, it was decided to include the term in the live discussion instead of presenting it again in the third survey.

DISCUSSED QUESTIONS:

- ❖ If **fasting** was defined as complete abstention from ALL foods and calorie-containing beverages (à only water / tea allowed), would **fluid-only fasting** (incl. juice / broth / linseed gruel or porridge) and **religious fasting** count as **modified fasting regimens**?
- ❖ Reminder: We have reached consensus on the term **religious fasting** together, defining it as "any fasting regimen that is undertaken as part of a religious practice", thus including dry fasting, intermittent dry fasting and diets restricting certain foods as practiced in Christian orthodox traditions.

Dialogues/comments:

Question: How should "fasting" be defined? What about the fasting regimens that do not require zero-calorie intake, but have been called fasting in the past? (This was also discussed in breakout rooms)

P05: Define a term that is **fasting** that is **zero intake of calories** and **a separate term for modified fasting (MF),** then have a sub-debate on what constitutes MF.

P02: What is the reduction you need on the MF days?

P05's answer: in America / Europe there is a kind of move to talk about very-low-calorie-diets (VCLD) and that could provide a nice framework to define MF – of course this is sth. for the group to decide but in general it would be beneficial to have a clear definition of fasting for the public and to say that "fasting" means no caloric intake and it would be good to have a sperate term for MF that mimics effects of fasting but is not zero calorie.

Comments after the breakout rooms – one participant from each room presented the outcome of the small group discussion:

P05: good consensus in our group:

à "fasting" = zero intake of calories
à "modified fasting" = low intake of calories
à have a third term (as a subtype of fasting) = dry fasting;

Reasoning: there are cultural differences based on countries; problem could be degree of confusion that is created when someone says fasting and by definition we allow zero calorie intake AND a certain amount of calories, e.g. 25% of their caloric intake; the public as well as practitioners might get confused with what they (patients) are doing / not doing;

In America there are also protein-sparing diets to mimic effects of fasting while preserving lean mass.

P06: There are **two crucial points** that are **missing in the definition of fasting right now**:

1. **maximum amount of kcal/d –** proposal: max. amount of 200(-500) kcal on fasting days
2. **minimum amount of duration of fasting** – proposal: minimum duration of 20 hrs;

There could be two terms: "total fasting" = zero calories and "fasting" = allowing for some kcal (threshold should be discussed)

P06: P11 explained that "fasting" in Germany allows a very low-calorie intake of up to 400 kcal/d; for me "fast" means zero calorie intake; the problem we don't know the physiological consequences of allowing certain amounts of calories, everybody is different, body size should be taken into account when defining MF.

P14: I agree with P05 à differentiating between **three terms**: "absolute/ complete fast", "modified fast (some kcal allowed)" and "dry fasting"

P09: I favour the given definition in the second survey (= the one presented above):
Fasting refers to a voluntary abstinence from some or all foods ~~and /~~ or foods and beverages for preventive, therapeutic, ~~spiritual~~ religious, cultural, or ~~political~~ other reasons.
reasoning: There is a long tradition of "fasting", the word "fasting" has been used in all the described forms in the definition (cultural, religious, therapeutic, etc.), hence it is perfect as is.

P01: The term "fasting" used to also be used in Germany for zero-calorie-diets BUT the aim was then to not propogate zero-calorie diets to avoid protein catabolism. For this reason, MF was introduced. In general, it is logical to say "fasting" = zero kcal, "modified fasting" = some kcal allowed.

P11: In Germany MF is not [generally] **25% of usual caloric intake** because men and women have different kcal needs, hence we say 200-400 kcals (no matter of gender or leanness) but not more; in Germany there is no zero-kcal fasting today.

Questions: It seems necessary to distinguish certain types of fasting from others. Nevertheless, can these types all be called "fasting" because they have been called fasting in the past? Or is this group proposing to use a different use of language, or should fasting, when used in the medical field, mean something specific?

P05: I suggest doing a poll to see who is in favor of MF and fasting as separate definitions.

P08: We want to do medical guidelines, I can't imagine that fasting allows 300-400 kcal, in my opinion it should be zero kcal (no food and caloric beverages) to allow for ketogenesis to occur - if calories are ingested ketogenesis doesn't happen (unless maybe very low amount of kcal); maybe make a differentiation between total and partial fasting.

Moderator: in the German therapeutic fasting, some kcals are allowed and it has been shown that ketosis is actually achieved.

P06: Most of us are thinking not necessarily in a physiologic sense but in a therapeutic perspective, the most important metabolic change that could induce therapeutic effects is reduction of blood glucose, increasing ketone bodies, changes in amino acids, etc.. Regardless of these, basically we all agree that a strong reduction of kcal intake is the most fundamental aspect, the majority of us agree that zero-kcal beverages can be allowed in those cases where they are necessary to prolong the fasting for the organism to survive.

P05: What we could do now is either "agree to disagree = e.g. have a 60:40 split" or introduce separate terms to describe "total fasting" versus "MF", even if we can't come to a consensus on what "fasting" alone means; if in Germany MF seems to be beneficial, then it is important to have this terminology for patients; we need separate definitions for subtypes of fasting.

Moderator: That was the actual idea we had: to have a broad term for "fasting" and then adapt it to different situations.

P09: When we talk about "fasting" we need to remember that "fasting" is an old word. In the history of fasting various forms have been employed, and these forms have to be included in the definition of "fasting". Zero-kcal diet was never a part of fasting except for in the last decades. 2500 years ago, there

were no zero kcal diets or zero-kcal beverages, they didn't exist, so in my opinion the definition should stay as mentioned above (= A2).
Moderator: One expert that can't be in the discussion was very adamant on the fact that fasting has always been sth. more than just a diet, because historically fasting has a lot of environmental factors that support it: It is an experience of going without certain habits and specific foods to have a specific experience. This is also what we see in the clinical field: Besides losing weight, patients also gain the experience of "not needing so much to be happy", they get a different satiety feeling, they become more self-satisfied.
P10: From a methodological perspective: if we want to define "fasting" – since there are different kinds of practices – the common features and characteristics must be found and listed, so the definition should be somewhat broad. I also agree with the definition A2. Based on this definition, we can then articulate the different types of fasting.
P17: The criteria for the definition of "fasting" are different and all are correct – some have historical criteria, some have present-day criteria, some have functional / metabolic criteria – maybe it would be good if everyone stated what their criteria is; and to have a practical definition with a broad consensus that gives a good message to the public to practice fasting. If we are too narrow, we might miss a large amount of people that would otherwise be open to try fasting. In scientific papers, of course it needs to be stated how many kcals are allowed, but for the public it is not that important.
P06: I agree that the definition should be broad, but as it is now [A2] it might be too broad, the most crucial elements are the **kcal amount allowed per day / per BMI and minimal duration of fasting**, these should not be missed in such a definition.
Moderator: It seems that on the one hand we have the need for very clear definition for publications to set a clear frame, and on the other hand we want to have a broader definition so that people doing research in the field of fasting can define their fasting methods very specifically in their own work.
Question: Who agrees with definition A2:
Fasting refers to a voluntary abstinence from some or all foods ~~and /~~ or foods and beverages for preventive, therapeutic, ~~spiritual~~ religious, cultural, or ~~political~~ other reasons.
Poll: **68.78% = 11 / 16 participants agree with A2**; 18.75% = 3 / 16 participants want a more specific definition (e.g., stating kcal amount / minimum duration of fasting); 12.50% = 2 participants didn't vote
Moderator: Even though we decided for a broad definition of the term "fasting", we would like to have clear definitions for additional terms such as "total and complete fast" and "modified fasting". In this way, "fasting" may serve as an umbrella term, but we also have other, more specific terms for our publications to make it very clear what we mean.
P06: I am fine with the definition A2 BUT in some of the following definitions, kcal amount and durations are highlighted, and I thought that all definitions should be consistent, so if it is decided that we have a general definition of the term "fasting", that definition should either also include these facts or none of the definitions should highlight these facts in order to be consistent.
Moderator: Maybe it would be an option to define "fasting" in a therapeutic sense → To make the definition of "therapeutic fasting" that we already have more specific OR say that "fasting" in a medical context means "xyz".
P06: Of course it depends on the perspective: clinicians want to standardize the approach regardless of the final aim which is of course therapeutic, so we are biased in that sense. What is the objective of this statement paper? Is it a medical / therapeutic / clinical one? If yes, then ALL definitions need to be standardized; if the aim is more conceptual and less therapeutic, then I can agree that specifications in kcal and fasting duration is not so important.
Moderator: The interesting thing is that more and more research is done in the field "in between" from people that come from religious fasting traditions looking at "fasting" now from a health perspective & try to find parameters of these fasts that could also help in therapeutic fasting settings, example: in psychiatric colleague's clinic: Buddhistic traditions are a part of his clinic and he combines fasting and meditations to enhance both interventions. This is the field that is upcoming, and this is why we are sitting here, to see how we can have a talk about these things and understand each other.
P02: Wikipedia reference: "Fasting is the abstention from eating and sometimes drinking. From a purely physiological context, "fasting" may refer to the metabolic status of a person who has not eaten

overnight, or to the metabolic state achieved after complete digestion and absorption of a meal." Wikipedia
Comment: My reference is not Wikipedia in general but anyways [the point I want to make is:] my grandchildren know what fasting means, there is a general definition of fasting. I think we should forget about all that and go to the medical or physiological practice of fasting and then it is "total fast" or "MF" and we need to have clear definitions for these two. But if we are stuck with the term "fasting", which generally everybody knows what it means (= to reduce kcal), but which has a slightly different meaning for everyone [then we will get nowhere]. We should therefore focus on the fasting definitions related to science. We don't have to redefine all the dictionaries but when it comes to medical practice, we should have a clear definition of "fasting" and define what **we** mean by fasting.
Moderator: We will then have a broad definition of "**fasting**" and a definition of "**total /complete fasting**" and "**MF**". A few questions remain regarding "**total /complete fasting**".

1.2 TOTAL FAST

The term **total / complete fast** was included as an additional term in the second survey because some experts had mentioned this term as an alternative to the general term **fasting** or **dry fasting** in the first round of the survey. However, we wanted to define total fasting separately to distinguish it more clearly from similar terms like **fasting**, **water-only fasting,** and and **fluid-only fasting.** In our proposed definition we included the historical term **zero-calorie diet**.
The following definition reached a consensus of **85.85 %** in the **second** survey:

> *The term **total fast**, or **complete fast**, refers to a fasting regimen, where only calorie-free beverages, including water and unsweetened tea, are consumed ad libitum for a certain period of time. Historically, prolonged total fasts were used for the therapy of people with obesity under the term **zero-calorie diet**.*

Two participants suggested that this definition should rather be titled **fasting** or **water-only fasting.** Another two participants again pointed out that the term **total fast** would better fit the definition of **dry fasting**. It was therefore decided to delete the above definition and list the terms **total fasting / complete fasting** under **dry fasting** in the third round of the survey. This was criticized by several participants in the third round (see comments under the term **dry fasting**).

QUESTIONS TO BE DISCUSSED:
- Should the term **total / complete fasting** equal the term **fasting / water-only fasting**? OR Should the term **total / complete fast** be omitted altogether?
- Should the term **total / complete fasting** equal the term **dry fasting**?

Questions:
1. Who agrees with the following: ***Total fast and complete fast*** *are the same thing, meaning no calories consumed during a period of the fast?*

Poll: **93.75% = 15 / 16 participants agree.**

2. Since the terms "**total / complete fasting"** are sometimes used as synonyms for "**fasting**", how should we deal with the wording of the term "**alternate-day fasting (ADF)**": Should the term **ADF** be changed to "alternate-day **total** /**complete fast**", even though ADF is a branded term, or can it remain as it is?

*Question: An option would also be to keep the name ADF but state in the definition that ADF means a day of **total/ complete fast** plus a day of ad libitum eating. Who agrees with that?*
Poll: **75% = 12 / 16 participants agree.**

3. What do you prefer: *Should we always use "**total / complete fast**" or is one term, either "**total**" or "**complete**", more popular / better?* What are your opinions?

P06: I prefer [having] both, with slash in between, but it means the same, if one has to be chosen, then "complete".

P14: There is some differentiating between no food and no water versus just no food.

Moderator to P14's comment: That is true, in Israel they also understand total fast as dry fasting (= no fluids allowed). We also have dry fasting experts in this round. Since total fast equals dry fasting in some cultures, what do you think?

P17: The term "dry fasting" is used in many publications and seems to be clear [= no fluids and food allowed]. The problem might be that some people want to differentiate between "no calories" and "no fluids". So, from a hierarchy of thinking, I would say fasting without calories comes before fasting without fluids. Fasting without calories I would call "complete fasting" and fasting without fluids "dry fasting".

P09: I agree that we should have two different terms for dry fasting and total fasting. There are publications that use "total / complete fast" and those should better not to be confused with "dry fasting".

P18: A distinction should be made between "water-only fasting" and "zero-calorie fasting with beverages" because there can be the danger of patients thinking zero-calorie beverages such as Coca-Cola zero are allowed to be consumed during the fast. So maybe it would be better to not call it "fluid-only fasting" but "water-only fasting" and make it clear that beverages such as tea can be part of that regimen but sodas like Coca-Cola zero shouldn't be included.

P01: We could say "non-processed fluids" / "non ultra-processed fluids"

Moderator: Yes, I also had patients that wanted to drink zero-calorie Coca-Cola while fasting because they thought it was allowed, so maybe we should take this comment into the publication as a note for practitioners OR use the term "non-processed fluids" in the definition of "fluid-only fasting"?

P18.: Yes. And since it is not clear in all cultures, I think it would be important to make a distinction between "**dry fasting**" and "**non-processed liquid-only fasting**".

Moderator: So, we agreed on total / complete fast meaning no intake of calories without any other subtext, but dry fasting would be different.

P17: I have a question regarding "water-only fasting": Are teas problematic in water-only fasting? If so, then we could stay with water-only fasting?

Moderator: There are forms that object teas, there are forms that only use distilled water.

P14: Yes, tea is not the problem, but caffeine would often be restricted / excluded, if someone is on a complete / total fast such as in a Daniel Fast.

P05: I changed my opinion, I now agree with the definition A2 of "fasting", since we decided to have subtype definitions like complete-, total-, water-only-fasting versus MF.

P10: I like the term "hierarchical" that P05 used previously because we are defining a hierarchy of definitions and we should include this word "HIERARCHY" when describing the process of methodology that has been adopted.

Moderator – answer to P10: Great idea. P05 also said she would help with visualizing the publication, maybe the following is sth. we could visualize: How are the terms connected / visualizing the hierarchy of terms so that there is a better overview.

DECISION:

Fasting = umbrella term (includes religious fasting regimens as well as fasting regimens that allow some caloric intake such as the Buchinger fast in Germany)

Total / complete fast = no calories consumed during the fasting period (no extra subtext)
Water-only fasting = complete / total fasts with only water being consumed *(consensus already reached in the survey)*
Fluid-only fasting = non ultra-processed fluids / liquids (allows water, tea, juices without added free sugars)
Dry fasting = Dry Fasting refers to a fasting regimen, during which a voluntary abstinence from all foods and beverages, including water, is practiced for a certain period of time. *(consensus already reached in the survey)*

1.3 MODIFIED FASTING

At the end of the first survey, we asked you if there were any other terms that you considered important to this consensus process. **Modified fasting** was one of the requested terms and was therefore included in the second round of the survey.

The following definition reached a consensus of **75.76%** in the **second** survey:

> *The term **modified fasting** refers to limiting energy intake to 20-25% of energy needs on fasting days.*
> *Modified fasting regimens are often adapted to specific clinical settings and indications, allowing for different complementary or supportive therapeutic interventions.*

However, some participants suggested several changes for this definition. Therefore, it was decided to include the term in the live discussion instead of presenting it again in the third survey.

QUESTIONS TO BE DISCUSSED:

- Should the term **fasting-mimicking diet** be part of the definition?
- Should the definition have a specific **maximum caloric intake**?
- If so, should the maximum caloric intake be a **range (20-25%/20-40%/20-50%)** or an **absolute number** of calories?

Based on the comments in the second survey round, we created two options for discussion / re-evaluation.

A1: The term **modified fasting** refers to limiting energy intake, typically to 20-25% of energy needs on fasting days. Modified fasting regimens are often adapted to specific clinical settings and indications, allowing for different complementary or supportive therapeutic interventions.

A2: The term **modified fasting** refers to limiting energy intake, typically to 20-50% of energy needs on fasting days. Modified fasting regimens are often adapted to specific clinical settings and indications, allowing for different complementary or supportive therapeutic interventions. An optimal composition of modified fasting can be achieved with a fasting-mimicking diet (FMD).

Moderator: We want to define MF (which has been used in publications up until now) BUT it has been decided that the kcal need to be stated as well as duration of minimum fasting and maybe specific foods that are (not) allowed. Maybe we could take the **alternate-day (modified) fasting** definition as an example for **MF** regimens since we already have very clear suggestions for kcal amounts / percentages of caloric intake allowed on the MF days in ADMF. → please read how the discussion continued at 2.2.

2. ALTERNATE-DAY (MODIFIED) FASTING

We actually started the discussion with this term and the question in the red box but as mentioned above, we wanted to keep the order we set in the agenda for this document.

2.1 ALTERNATE-DAY FASTING

The following definition reached a consensus of **82.35 %** in the **first** round of the survey:

Alternate day fasting (ADF) *refers to alternating a day of eating ad libitum and a day of either water-only fasting or a diet very low in calories.*

(Note that "or, in animals, **every-other-day feeding (EOD)"** was removed as it was decided to focus on fasting terms in humans.)

However, some participants requested a distinction between **ADF** and the term **alternate-day modified fasting (ADMF),** which allows some caloric intake on the fasting day. Therefore, in the second survey, we asked you to choose either the ADF definition from above or one that allows only water on the fasting day. In this second round, no clear favorite emerged. Since there were also differing opinions on the definitions of the general terms **fasting** and **modified fasting,** it was decided to include the terms **alternate-day fasting** and **alternate-day modified fasting** in the live discussion as well rather than presenting them again in the third round.

QUESTION TO BE DISCUSSED:

- Should we have two separate definitions for **alternate-day fasting** and **alternate-day modified fasting** or combine the two, keeping the definition of the first round of the survey?

If the definitions for ADF and ADMF should be separate, we propose to choose one of these three definitions of **ADF**:

A1: Alternate-day fasting (ADF) refers to alternating a day of eating *ad libitum* and a day of either water-only fasting or a diet very low in calories.

A2: Alternate day fasting (ADF) refers to alternating a day of eating *ad libitum* and a day of water-only fasting.

A3: Alternate day fasting (ADF) refers to alternating a day of eating *ad libitum* and a fasting day where only water and energy-free beverages are consumed.

Comments regarding the question in the red box:

P04: Yes, we should have 2 definitions (ADF / ADMF), my suggestion is: "zero calorie alternate-day fasting" instead of "alternate-day fasting" because it might confuse people. It means that it is water-only on fast day and *ad libitum* eating on feast /eating day; "modified alternate-day fasting" is the other major term used in the literature for humans, it means people can consume 25-30% of energy needs on fast day usually in lunch or dinner.

P06: I agree, we should have one day of total fasting (= ADF), or when allowing a minimal amount of calories call it "alternate-day modified fasting".

P02: We should have 2 definitions, I agree with P04, but the question is "what is zero, what is considered a day"? Is a day midnight to midnight? I wouldn't put a limit for calories on the ADMF fasting day, somebody who is willing to do 25% less calories already shows an effort to ADMF.

Moderator: This brings us back to what "fasting" actually means, is it a zero-calorie diet is it not? Is it a very low-calorie diet? In some traditions it means limiting food intake to certain foods, etc. in other contexts fasting means abstaining from food and beverages intake. → *After these comments on ADF were made, we returned to the term "fasting" / or rather began to actually discuss the term "fasting" in the breakout rooms, the results of which you have already read above.*

2.2 ALTERNATE-DAY MODIFIED FASTING

It was suggested that the term **alternate-day modified fasting**, which allows some caloric intake on the fasting day, be included as an extra term in the **second** round of the survey to distinguish this modified fasting regimen from **alternate-day fasting.** We asked you to select a maximum number of calories for the modified fasting days, but no consensus was reached.

QUESTIONS TO BE DISCUSSED:

- If we define **alternate-day modified fasting** separately from **alternate-day fasting** what maximum number of calories for the modified fasting day do you suggest?

We propose the following definition for **ADMF** based on the responses of the **alternate-day fasting** experts we asked via email (see their answers below):
Alternate-day modified fasting (ADMF) refers to alternating a day of eating *ad libitum* and a day of eating a low-calorie diet with up to 500 - 600 kcal.

Moderator: Maybe we could take the **alternate-day (modified) fasting** definition as an example for **MF regimens** to define the allowed **kcal-amounts** on the **MF days in general.**
For ADMF we already received proposals for the maximum absolute kcal amounts / ranges in % on the MF days. We need to clarify first: Should we use **absolute kcal amounts or % or range of %**? And then, how high should the caloric threshold be (in absolute amounts or %)?
P06: In my mind there are parallels between "fasting" and "ADF" as well as "MF" and "ADMF". We agreed that "total fast = zero calorie"; ADF = one day ad libitum / no restriction-day alternating with a zero-kcal day. For the general term "fasting", we were quite broad and decided not to define specific restrictions in terms of max kcals per day. [For this reason] I wonder why we should be so strict when defining the threshold of kcal / specifying this threshold in ADMF [when there is no] scientific reason for deciding for a specific threshold?
Moderator: Yes, it might be better not to have a concrete threshold?
P05: I want to speak up for Prof. Varady, who in most of her studies uses a 25% calorie intake on MF days, so she would disagree with a limit of 500-600 kcal and prefer either a **percentage less than 50%** or **no specific limit at all**. The "5:2 diet" allows for 35% kcal on MF days and it would be nice to include all those regimens under MF. But if we set the limit at 35%, what about FMD, should it also be treated as a MF approach? Then it wouldn't be included if we set the limit to 35% because Prof. Longo usually allows around 700-1100 kcal depending on the fasting-mimicking day.
P02: I have been part of some of [these mentioned] studies and P05 said what I wanted to say: Anything below 50% kcal would be ADMF. People understand kcal better than "% of energy requirement".
Moderator: We need to agree on using either kcal or % of energy requirements. In the survey, some experts also said that people's occupation and exercise pattern should be considered when defining energy requirements, but this would make the studies more complicated, since all these details must first be known before patients can be assigned to specific fasting regimens.

P01: To set standards for medical / scientific reports, we should use **% of energy requirements** in the definition of MF. In practical terms, we should have a look at the amount of kcal already used in MF in studies today: in Germany it is usually < 400 kcal (meeting the 25% criterium); But it is a continuum, so we have a problem: In prolonged-periodic-fasting in Germany traditionally 400-500 kcal should not be exceeded but then we have a continuum to the FMD because it gets lower and lower, today some days in the FMD allow 550 kcal, so it is very close [to the upper limit of MF], so to distinguish FMD from MF should we have a cut-off? I have no solution to differentiate between FMD and upper limit of MF.

P08: The main issue besides choosing the % of daily caloric intake for MF, is deciding the upper limit of the NON-RESTRICTED days: using the term "*ad libitum*" is not an appropriate term for human clinical-orientated guidelines /studies. It should be implied that "*ad libitum*" as it has been used primarily in animal studies, is not used for humans because we wouldn't eat 5000 kcal in 4 days → not appropriate term for human guidelines.

Moderator: That is an interesting point! IF and TRE- people usually eat less kcal even if they are told they can eat ad libitum. Yes, we need to give it a thought if we want to use "ad libitum" in our definitions or not!
P18: Since we are talking about *ad libitum* eating alternating with some lower energy intake, MF should be quite below 50% of kcal intake to have an impact on the fasting person, it should be around 25%. 500-600 kcal as a threshold might be good because we are talking about a consecutive day of eating as much as somebody wants, and in practice I have seen that people find a balance, so it is better if the energy intake is lower than 50% on the MF days.
Moderator: We have 3 suggestions regarding the kcal restriction on modified fasting days at the moment: 1. 500-600kcal, 2. percentages of energy requirement ≤ 50% and ≤ 25-30%

P11: I agree with P18: A 50% threshold has nothing to do with fasting it is just a caloric reduction. Fasting is much lower; it has to be at least 25% (= 400-500 kcal).

P13: We should create hypothetical people and plug the information in some of the prediction equations and see what the 25% kcal reduction equal in a kcal-range; example: 25% intake = 300 kcal for a 45 kg female and up to 700 kcal for a 120 kg male → just so someone reading the guidelines doesn't have to do the math themselves.

P05: That sounds like a fantastic idea, I vote to do everything in % of energy requirements because patients burn different amounts of kcal per day, so there is "no one size fits all-kcal-requirement"; Another word of caution: The largest study done on ADF (done by Prof. Varady) used a 25% threshold on MF days, and I suggest to create a definition that is compatible with this largest study, so not going above 25% makes sense. This goes for any type of MF regimen on which large research studies have already been conducted.

Moderator: So we need clarifications on the following to questions (those are also important for the definition of caloric restriction later):

1. Do we want to keep the **absolute kcal amounts** or **% of energy requirements on the MF days**?

Poll: kcal amount: 26,67% = 4 / 15 participants; **% of energy requirements: 60% = 9 / 15 participants**; 20% = 3 /15 participants didn't vote (→ 2 didn't say anything / maybe haven't been on the screen when doing the poll)

P14: Using percentages makes sense, assuming a person knows what their actual intake and therefore their % in kcal is. In practice though a lot of people have no idea how many kcal they consume, so if they are told not to go above 25% of their normal intake, they wouldn't know what it means. Of course if you do a diet diary with the patients you can calculate it but if people are just reading the guidelines without a practitioner then they wouldn't have any reasonable idea of what the % means in kcal, this is why sometimes an absolute number helps people to take action, this might need to be mentioned in the publication.

Moderator: It was suggested before that after the definitions, we give practical examples of e.g., a 120 kg male and a 45 kg woman and how many kcal that would amount to, or do you still believe it would be better to just have the kcal amount in the definition?

P14: Yes, those two examples certainly help, but the point that P05 made earlier needs to be considered: A very large person has a much higher total energy expenditure than a small person but you can give a few examples for people to wrap their heads around and hopefully they will talk to a practitioner that will help them determining their kcal intake. I was just concerned about the people out there that are trying to figure things out alone and will not know how to approach the percentages.

Moderator: So, in your opinion, *could we **keep the kcal percentages in the definitions**, but give examples so people have an idea of what the % range corresponds to in absolute kcals?*

→ **Collective answer: Yes**

P09: I am comfortable with both, I don't find a difference, since the other day is *ad libitum* eating anyways.

P18: I agree with P09.

P13: It might be good to put a small table in with incremental changes in body mass and age, so that people don't have only the two extremes [super skinny vs obese] and they will have a better idea of how to apply it to themselves when reading the guidelines (so it will not just be useful for clinicians).

Moderator:

2. What should the **percentage of kcal be on the MF days** in general: 25% or 50% of energy requirements?
3. Should all MF regimens have a threshold of ≤ 25%?

Poll on 3.: **80% = 12 / 15 participants**; 20% = 3 / 15 participants didn't raise hands

P05: I didn't raise my hand because there are MF studies using thresholds of up to 35% (e.g. [in studies of] "5:2 diets"), so if we want to harmonize definitions for MF regimens, we might have to allow up to 35% and then FMD could also be subsumed under MF (hierarchy of fasting definitions: FMD, 5:2, ADF)

P01: It is really difficult [to decide the percentage]; For "5:2 diets" [35 %] is appropriate and it really allows for an umbrella term but for the FMD-diet team we don't know what is the attitude because they coined the term FMD and besides the caloric amount they also created some specific macronutrient proportions, so if we say 35% [is the threshold], we don't know, if the team by Prof. Longo would still agree to get included with the term MF.

Prof. Longo's comment from the survey on the presented definition of MF: ***25% of calories from the wrong source, let's say proteins could completely reverse many of the effects of fasting****, so unless the term* ***fasting mimicking diet*** *is also included, I'm against this term.*

→ *P08:* Any source of macronutrients can induce glycolysis either protein or carbs so in a pathophysiological basis I don't understand the background on this skepticism.

Moderator: If we still stay with the hierarchy, this would not be a problem anymore because then the FMD can have a more specific and more defined fasting regimen definition with exact macronutrient percentages.

P06: I understand Prof. Longo's point and agree but so far, we have avoided discussions on macronutrient compositions of the fasting regimens. I would also suggest doing the same for the definition of MF. Then, we can decide whether FMD is one specific object which merits specific definitions in terms of duration or whether it's about kcal per day / % of energy or macronutrients but for MF definition I would suggest avoiding going into details of macronutrients.

P12: As far as I understood, one has to reduce sugar and protein to activate repair genes so we should find a solution for this subject.

Moderator: The following question remains: *Should we go with 25% or 35% as a threshold for the caloric restriction on MF terms?* Since studies on the "5:2 diet" may be excluded if we decide for 25%, we have a problem and because we don't have any consent from the other experts that aren't here today, *perhaps we should bring this up for discussion in the next round of the survey?*

Collective answer: Yes

MODIFIED FASTING:

TO BE DISCUSSED IN THE 4TH SURVEY: Threshold of kcal intake on MF days: up to 25% or up to 35%?

MODIFIED FASTING REGIMENS:

WHAT IS THE PERCENTAGE OF THE ENERGY REQUIREMENT IN KCAL ON MF-DAYS?

One participant will prepare an example table for the next survey round for the other experts to know, how it would look like in the final publication.

6. CALORIC RESTRICTION

The following definition of **caloric restriction** reached a consensus of **88.24 %** in the **second** round of the survey.

> ***Caloric restriction (CR)*** *describes a reduction in energy intake below the total amount of calories that would be needed to maintain a person's current body weight, without causing malnutrition. CR may also be used to achieve a healthy body weight over time.*

However, no consensus was reached on the range of reduction in daily caloric intake.

Additionally, it was suggested that the term **continuous energy restriction** (CER) / **daily energy restriction** (DER) be subsumed under the term **caloric restriction.** Thus, the following sentence was added to the definition above and accepted during the third survey round:

*If **CR** is done daily, it can also be referred to as **continuous energy restriction (CER)** or **daily energy restriction (DER).***

QUESTIONS TO BE DISCUSSED:

- Should a **specific amount / range of reduction** in daily caloric intake be specified in the definition of **caloric restriction**?
- If yes, what amount / range should be specified?

Moderator: Would you stick to 25 or 35% [= same threshold as in MF] or do you think caloric restriction should have a much broader range OR we should not have any limits at all?

P06: I agree with the definition that reached 88% in the second survey:
Caloric restriction (CR) describes a reduction* in energy intake below the total ~~caloric intake~~ amount of calories that would be needed to maintain a ~~healthy~~ person's current body weight, without causing malnutrition. CR may also be used to achieve a healthy body weight over time. *Note: amount of reduction is not specified in this definition.
Comment: this broader definition of CR is better and in line with the broader definition for the term fasting.

Moderator: This definition has no suggested reduction in kcal or % of energy intake.

P06 and P11: yes, [we think that's fine].

P09: I prefer definition **A3** from the third survey:

Caloric restriction (CR) describes a reduction in energy intake below the total amount of calories that would be needed to maintain a person's current body weight, without causing malnutrition. The degree of caloric restriction should be decided individually depending on the person's age, sex, current body weight, occupation, goal & planned duration of CR. If **CR** is done daily, it can also be referred to as **continuous energy restriction (CER)** or **daily energy restriction (DER).**

Comment: This is a very good and balanced definition, because calorie restriction should be individualized!

P06: I agree with P09. A3 is broader and more precise but this [the first added and underlined sentence in blue in the definition] also applies to fasting regimens because they should also be individualized for therapeutic purposes, so if we decide to specify the individualization in the definition of CR, then it might also need to be added in a similar statement to the definition of fasting.

Moderator: Yes, we should probably include [the part of the individualization] in the definition of therapeutic fasting. We will include it in the 4th survey or include it in the background text as a comment.

P10: I want to make a comment on what P06 said "that fasting should be individualized", that is true if we consider the medical / therapeutic fasting, but since there is also religious fasting it doesn't make sense.

P06: I agree, this is why this "individualized sentence" should NOT be added to the broad definition of fasting but to the therapeutic fasting definition.

THERAPEUTIC FASTING

Moderator: We can include the sentence on individualization either as a comment or in the definition itself – we will see how we will offer the definition in the 4th round of the survey.

Moderator: **Back to the definition of caloric restriction**: Should we stick with the definition of the second survey that already reached consensus or vote on one of the three CR definitions from the third survey? (2 min break – Poll afterwards)

Definition of A3**:**

Caloric restriction (CR) describes a reduction in energy intake below the total amount of calories that would be needed to maintain a person's current body weight, without causing malnutrition. The degree of caloric restriction should be decided individually depending on the person's age, sex, current body weight, occupation, goal & planned duration of CR. If **CR** is done daily, it can also be referred to as **continuous energy restriction (CER)** or **daily energy restriction (DER).**

Who is in favor of A3?

Poll: **86,67% = 13 / 15 participants;** 13.34% = 2 / 15 participants didn't vote

Experts that didn't vote - reasoning:

P11: The first blue sentence was added and I would prefer a version without this sentence (=A1 see below) because for me A1 is enough.

P05: I feel similar to P11: A3 is a little wordy but I don't feel strongly about it, so me not voting for A3 is not a sign of disagreement per se, just a preference for an elegant and shorter definition.

Definition of A1:

Caloric restriction (CR) describes a reduction* in energy intake below the total amount of calories that would be needed to maintain a person's current body weight, without causing malnutrition. ~~CR may also be used to achieve a healthy body weight over time.~~ If **CR** is done daily, it can also be referred to as **continuous energy restriction (CER)** or **daily energy restriction (DER).**

*Note: amount of reduction is not specified in this definition.

Who is in favor of A1?

Poll: 53,35% = 8 / 15 participants;

Revote for A3: Who is in favor of A3 now? 46.67% = 7 / 15 participants

CALORIC RESTRICTION:

Moderator: Since it is almost **50:50 for A1 and A3**, we will offer both definitions in the 4th survey round for re-evaluation.

5. INTERMITTENT ENERGY RESTRICTION - INTERMITTENT FASTING - TIME-RESTRICTED EATING

5.1 INTERMITTENT ENERGY RESTRICTION

At the end of the first survey, we asked you if there were any other terms that you considered important to this consensus process. **Intermittent energy restriction** was one of these requested terms and was therefore included in the second round of the survey.

The following definition reached a consensus of **81.26 %** in the **second** survey:

> ***Intermittent Energy Restriction (IER)*** *includes periods of caloric restriction alternating with periods of ad libitum eating. As such, IER includes fasting regimens like intermittent fasting (IF) and time-restricted eating (TRE).*

However, some participants pointed out that **TRE** should not be part of the definition of **IER**. Therefore, we offered an additional definition in the **third** round:

> ***Intermittent Energy Restriction (IER)*** *includes periods of caloric restriction alternating with periods of ad libitum energy intake.*

In this third round, no clear favorite emerged. Therefore, it was decided to include the term in the live discussion.

QUESTION TO BE DISCUSSED:

- Should **Intermittent Fasting** and **Time-Restricted Eating** be classified under **Intermittent Energy Restriction**?

P05: I want to debate on how the term IER is used and to what degree IER means the same as IF – this might be a huge can of worms, but it would be important to be addressed.
Moderator: Can you expand on that point? How should we introduce this in the survey?
P05: I have heard that some people think that IF and IER are **not** the same thing and that they don't overlap at all and they might say that IF is a water-only fasting for some number of hours in duration and that it doesn't include any sort of MF approaches, those same people will also put MF under IER, they would say any sort of periodic MF approaches are IER; but I tend to take a more inclusive approach and say that **IER is A FORM OF IF**!
Moderator: I would have thought that IF is a sub-category of IER not the other way around.
P05: It is really interesting, I wish Prof. Heilbronn was on this call because she did bunch of studies that involve IF but not a decrease in caloric intake so it is technically not IER per se cause it all evens out, the patients are maintaining their weight, meaning that there are ways that you can achieve periods of extended fasting without reducing caloric intake and TRE would be another classic example of that.
Moderator: Although overnight for a certain period of hours you reduce the calorie intake as such, but if you take the 24 hours you have the same amount of calories.
P05: Yes, it can be nuanced, and it really depends on your perspective, and you can argue in many different points.
Moderator: I see, would you be willing to prepare a suggestion for the next written survey on the point of IER?
P05: Sure, I propose suggestions from opposing viewpoints, so the experts have a range to vote on.
Moderator: It would be best to have 2 different definitions since we have had bad experiences with 3 options (we usually don't reach a consensus) but if it is not possible and there are 3 then we will start with 3, and take the 2 that have more votes and offer them in the following round.

INTERMITTENT ENERGY RESTRICTION
P05 will propose definitions for IER for the next survey round.

5.2 INTERMITTENT FASTING

The following definition of **intermittent fasting** reached a consensus of **88.24 %** already in the **first** round of the survey.

> ***Intermittent fasting (IF)*** *refers to repetitive fasting periods of up to 48 h. IF includes fasting regimens of 1 day per week (6:1), 2 separate or consecutive days per week (5:2), alternate day fasting (ADF) and time-restricted eating (TRE).*

However, some participants voted to exclude **TRE** from this definition. Therefore, we offered an additional definition in the second round:

> ***Intermittent fasting (IF)*** *refers to repetitive fasting periods lasting up to 48 h each. IF includes fasting regimens of 1 fasting day per week ~~(6:1),~~ 2 separate or consecutive fasting days per week ~~(5:2)~~ and alternate-day fasting (ADF). ~~and time-restricted eating (TRE).~~*

In this second round, no clear favorite emerged, hence we proposed the following definition in the **third** round that reached a consensus of **86.67 %.**

> ***Intermittent fasting (IF)*** *refers to repetitive fasting periods lasting up to 48 h each. IF includes fasting regimens of 1 fasting day per week, 2 separate or consecutive fasting days per week, alternate-day fasting (ADF), and time-restricted eating (TRE).*

A few participants still voted to exclude TRE from the definition.

QUESTION TO BE DISCUSSED:
- Should **Time-Restricted Eating** be considered a sub-category of **Intermittent Fasting**?

5.3 TIME-RESTRICTED EATING

The following definition of **time-restricted eating** reached a consensus of **91.18 %** already in the **first** round of the survey.

> ***Time-restricted eating (TRE)*** *is a dietary regimen in which food intake is restricted to a specific period of time (usually 4 to 10 hours) during the day, resulting in a daily fasting window of <u>14 to 20 hours</u>. There is no explicit limit on energy intake during eating or feeding hours.*
> (Note that *"or, when referring to animals,* ***time-restricted feeding (TRF),"*** was removed as it was decided to focus on fasting terms in humans.)

However, a few participants wished to expand the fasting window and to include the **one-meal a day diet (OMAD)** under the definition of **TRE**. Therefore, we offered additional definitions to choose from. In the past two rounds, <u>no clear favorite emerged</u>.

QUESTIONS TO BE DISCUSSED:
- ❖ How long should the fasting window be for **TRE**?
- ❖ Should **OMAD** be included in the definition of **TRE**?

<u>Moderator:</u> What is the hierarchy with IF / TRE? Should TRE be included in IF or not?

<u>P13:</u> In the third survey the following definition reached a consensus of 86%:

A3: Intermittent fasting (IF) refers to repetitive fasting periods lasting up to 48 h each. IF includes fasting regimens of 1 fasting day per week, 2 separate or consecutive fasting days per week, alternate-day fasting (ADF), and time-restricted eating (TRE).

Comment: Why are we discussing the definition if we already reached consensus?

<u>Moderator:</u> Because two people disagreed with including TRE under IF.

<u>P05:</u> it is a very hot topic "what is IER, what is IF"; TRE in the US can be practiced with and without reducing energy intake – so when I give talks, I don´t define TRE as a form of **IER**. Under IER I define all MF regimens that are periodic in nature (AD(M)F, FMD, 5:2 diet) – I would argue that all of those approaches are IF-approaches; but back to P06' point that we should discuss the minimal duration of fasting: [For TRE] we do have a consensus on 14 hours as a minimum duration of fasting, right? Because then the question is resolved → most TRE-approaches are at least 14 hours, so then we could shorten the discussion.

<u>P05:</u> And since we agreed that TRE is fasting practiced for at least 14 hours, then we should agree that TRE should be counted as IF.
<u>Moderator:</u> Does everyone agree on the term TRE being part of the hierarchy of IF and IER?
Collective answer: Yes

TIME-RESTRICTED EATING

<u>Results from the 3 survey rounds on TRE:</u>

In the first round we reached >90% consensus on the following definition:

Time-restricted eating (TRE) or, when referring to animals, **time-restricted feeding (TRF)**, is a dietary regimen in which food intake is restricted to a specific period of time (usually 4 to 10 hours) during the day, resulting in a daily fasting window of <u>14 to 20 hours</u>. There is no explicit limit on energy intake during eating or feeding hours.

→ In the last two rounds however, we didn't reach consensus anymore (we offered 3 definitions in the second round and 2 definitions in the third round). Since there was a consensus on the **minimum fasting hours being 14 hours** in round 1, we stick to that and leave the upper limit open in the definition.

Fourth Questionnaire

Defining Fasting: Finding Common Ground Using the Delphi Method - Round 4

Please read the following information thoroughly before starting the second questionnaire.

As a reminder:

We modified the fasting terms and definitions according to your suggestions. In the definitions you will see crossed out words in red and newly added words underlined in blue to make the modifications visible. We also provide you with single or summarized comments of the panel experts in anonymized form, as well as the distribution of votes from the last round, to help you decide. However, in order to make this survey as compact as possible, not all comments are listed. (We are happy to send you **a document with all comments on request**).

Guide for the colors & italics in the comments

- ❖ Comments that occur more than once are highlighted in orange / light blue / purple
- ❖ Text in italics: alternative definitions for terms
- ❖ Purple background: alternative names for described definitions

---

Terms on which consensus has been reached in the last survey(s) but which need to be re-evaluated due to comments by participants

**Specific Fasting Regimens**	
**FASTING-MIMICKING DIET (FMD)**	**FASTING-MIMICKING DIET (FMD) –** In the second survey we asked you to indicate whether you consider the inclusion of the term **Fasting-Mimicking Diet** important for this consensus process. Although no consensus was reached, we proposed to include the term FMD because of the growing body of research on this diet and offered the following definition:<br>A **Fasting-Mimicking Diet (FMD)** specifies any diet that is specifically composed to induce the metabolic effects of fasting while allowing for a potentially higher caloric intake. It usually refers to a plant-based, calorie restricted diet with a maximum of 1000 kcal per day with solid food components that lasts 3 to 5 days and is followed periodically (e.g. once a month). **FMDs** are usually free of refined sugars and starch and low in protein.<br>**Distribution of votes:**<br>**►70.37 % of the panel experts voted in favour of this definition.**<br>► **3.70 %** of the panel experts voted neutral.<br>►**14.82 %** of the panel experts selected "disagree / strongly disagree".<br>►**11.11 %** of the panel experts voted that this definition is irrelevant.<br>Although consensus was reached for this definition, several changes were proposed.<br>Experts' comments:<br>*AGREE:*<br>• I would delete the words "free of refined sugars and starch and" so that the last sentence reads: "FMDs are usually low in protein."<br>• I propose a modification of this definition as follows: *"Fasting-Mimicking Diet (FMD) specifies any diet that is specifically composed to induce the metabolic effects of fasting* ***, such as a reduction of blood glucose and growth factors, while allowing for a potentially higher caloric intake than fasting****. It usually refers to a plant-based, calorie restricted diet with a maximum of 1000 kcal per day that lasts 3 to 5 days, and which is followed periodically (e.g. every 2, 3 or 4 weeks, also depending on daily calorie content). FMDs are usually free of refined sugars and starch and low in protein content. "*<br>*NEUTRAL:*<br>• I suggest adding that FMD as a term and as a diet was originally developed by a for-profit company.<br>*DISAGREE:*<br>• a very low protein low calorie diet with a maximum of 1000 kcal/day...<br>*STRONGLY DISAGREE:*

- can't say It *usually refers to a plant-based, calorie restricted diet with a maximum of 1000 kcal per day with solid food components that lasts 3 to 5 days and is followed periodically (e.g. once a month). FMDs are usually free of refined sugars and starch and low in protein*. à This is one commercial version, **there could be others**
- FMD is just another method of caloric restriction, because with a daily intake of up to 1000 Kcal you cannot induce the same metabolic effects of traditional fasting which requires far less calories.

THIS DEFINITION IS IRRELEVANT:

- I cannot see that you considered FMD as industrial products without limitations eg as to content of minerals, amino acids, vitamins and so forth. Free of refined sugars and vegan origin is too weak to associate it to fasting with natural products.

We submitted the comments to the leading expert on FMD and, after consultation with him, decided to modify the definition according to his input (see below). Please note that we have deleted the comment about „periodic practice" as we feel this is inconsistent with our proposed definition of **periodic fasting**.

To what extend do you agree or disagree with this modified definition?

A **Fasting-Mimicking Diet (FMD)** specifies any diet that is specifically composed to induce the metabolic effects of fasting while allowing for a potentially higher caloric intake, including solid foods. It usually refers to a plant-based, calorie restricted diet with a maximum of 1400 kcal per day ~~with solid food components~~ that lasts 3 to 7 days ~~and is followed periodically (e.g. once a month)~~. FMDs are usually relatively low in refined sugars and starch, low in protein and high in plant-based fats.

Note: It is recommended to follow the FMD once a month to every four months.

The expert's reasoning:

- We are using 1400 kcal for Alzheimer's and elderly patients. That's the whole point: get as much fasting response as possible without endangering the life or health of the patient
- It does not mean you get a full response but based on the fmd composition you can still get a very strong fasting response. Don't forget that someone with 10 pounds of fat has a 50,000 kcal reserve or so, yet they enter a full fasting response when placed on water only.

Answer	n	%
Strongly Agree (AO01)	12	36.36%
Agree (AO02)	14	42.42%
Neutral (AO03)	0	0.00%
Disagree (AO04)	2	6.06%
Strongly Disagree (AO05)	3	9.09%
This definition is irrelevant and should be excluded from the consensus process (AO06)	2	6.06%
Due to my specialization, I am not familiar with this term / this method and prefer not to vote on it (AO07)	0	0.00%

STRONGLY AGREE: further thoughts

P05: I strongly believe that we should defer to Valter Longo on how he wants to define FMDs. So I agree with his proposed definition above.

AGREE: further thoughts

P20: But IMO there is really not enough research on FMD by enough separate groups to define exactly what they are in terms of length, timing/ kcal composition.

P36: The recommendations should be personalized as the FMD may be inappropriate for certain subject (anorexia person with BMI 17) or someone who is 90 years old. Also the frequency and type of FMD should depend on pre-clinical and clinical trials performed on the use of FMDs against a particular disease or condition

P17: I also miss the **'industrial' aspect which means any form of FMD I know is a manufactured product** (this should be part of definition) **in contrast to natural alimentation as is used for 'conventional' fasting**.

Also, if a person realizes any advantage after practicing FMD for 7 days, what will keep him from continuing esp as it is claimed to be safe? So 3 to 7 days cannot be more than a recommendation.

I did not realize that a high content of plant based fats is considered to be essential, but i am not practicing FMD.

DISAGREE: suggested modifications / alternative definition

P34: it should be "plant-derived protein" to avoid increases in IGF1

P32: I think maximum of 1000 cal and having and is followed periodically (e.g. once a month makes this more accurate

STRONGLY DISAGREE: suggested modifications / alternative definition

P06:	I disagree with this definition for two main reasons:<br>a) the **threshold of 1400 Kcal per day is definitely too high** **for such a regimen**. Indeed, this threshold is close to a normocaloric diet for several people, including people with low height/BMI. In addition, this amount of calorie is unlikely to cause the positive metabolic effects that are expected by a FMD, such as a reduction of blood glucose, growth factors and lipid levels. For instance, in the study by Valdemarin f et al (Cancers), in which a FMD regimen or max 1000 Kcal/die was administered to cancer patients, the metabolic effects were much milder than those obtained with a more severe FMD regimen (Vernieri et al. Cancer Discov 2022). I suggest max **800-1000 Kcal** per day in this definition.<br>b) it is stated that a FMD should "induce the metabolic effects of fasting". However, according to the definition of fasting that was accepted by the majority of us, fasting is the abstinence from some or all foods. This definition does not necessarily imply a reduction in calorie intake, nor of specific macronutrients. For instance, totally avoiding meat but consuming a high amount of carbohydrates or sugars can be still defined "fasting" according to this definition. Being this definition generic and including different types of dietary styles/regimens, no unique metabolic effects can be expected. Therefore, I would modify the definition of FMD as a regimen that is able to "**induce the metabolic effects of <u>complete</u> fasting**", rather than "fasting"
P11:	How do we know whether we really achieve the same metabolic effects of fasting like autophagy, ketosis, hormonal changes, reduction of proinflammatory cytokines and so on with a daily intake of 1000 or even 1400 Kcal also containing many carbohydrates ? Do we have valid scientific data proving this statement?
P33:	This should be a general term. There are likely to be many different versions of this i.e low carb not low protein and of different lengths.<br>The definition currently proposed SHOULD BE REMOVEDIT IS REFERRING TO ONE COMMERCIAL PRODUCT and not a class of dieting<br>*Fasting-Mimicking Diet (FMD) specifies any diet that is specifically composed to induce the metabolic effects of fasting while allowing for a potentially higher caloric intake*.
**This definition is irrelevant and should be excluded – comments:**	
P15:	Who is "the leading expert" on FMD? Did they disclose any financial conflicts of interest?
**<u>RELIGIOUS FASTING</u>**	**RELIGIOUS FASTING –** In the third survey, we offered the definition with the majority of votes from the previous round with the additional synonyms of **dry fasting** (= **total / complete fasting**) because some dry fasting experts mentioned in the second round that these terms can be used interchangeably:<br>**Religious fasting** refers to any fasting regimen that is undertaken as part of a religious practice. Religious fasting thus involves practices such as: **dry fasting (= total / complete fasting)** on specific days of the year up to 25 hours at a time (e.g. Jewish tradition, The Church of Jesus Christ of Latter-day Saints); intermittent dry fasting (e.g. Ramadan fasting, Bahá'í fasting); time-restricted eating (e.g. Buddhism); and diets restricting certain foods (e.g. Christian orthodox traditions, Daniel fast) if more broadly defined. Typically, religious fasting also includes spiritual activities to improve cognitive function and well-being.<br>**Distribution of votes:**<br>► **79.31 % of the panel experts voted in favour of this definition.**<br>► **3.45 %** of the panel experts voted neutral.<br>► **17.24 %** of the panel experts selected "disagree / strongly disagree".<br>Although consensus was reached for this definition, several changes were proposed.<br>Experts' comments:<br>*AGREE:*<br>• *Religious fasting thus involves practices such as: dry fasting on specific days of the year* ***up to 72 hours (130racticed orthodox tradition), 25 hours at a time (e.g. Jewish tradition, The Church of Jesus Christ of Latter-day Saints***<br>*DISAGREE:*<br>• don't think we need to describe the spiritual elements in a nutrition definition<br>• don't see why the last sentence should be part of the definition; we are defining food intake NOT other religious practices. Otherwise you need to start including things like special dress associated with the fasting, prayers, etc. It doesn't end. Let's stick to just food/liquid intake.<br>• *ALTERNATIVE DEFINITION: Religious fasting refers to any fasting regimen that is undertaken as part of a religious practice.* I prefer short and concise definitions. Why add examples?<br>More than two experts noted that dry fasting should not be equated with total / complete fasting in the third round. This was also raised in the discussion and participants agreed to use the terms **total / complete fast** for a different definition. For this reason, the additional synonyms of **dry fasting** will not be included in the final definition of **religious fasting**.<br>Based on the comments received, we decided to adapt the definition of religious fasting as follows. To what extend you agree or disagree with this definition?<br>**Religious fasting** refers to any fasting regimen that is undertaken as part of a religious practice.

Note: Religious fasting thus involves practices such as: dry fasting on specific days of the year up to 25 hours at a time (e.g. Jewish tradition, The Church of Jesus Christ of Latter-day Saints); intermittent dry fasting (e.g. Ramadan fasting, Bahá'í fasting); time-restricted eating (e.g. Buddhism); and diets restricting certain foods (e.g. Christian orthodox traditions, Daniel fast) if more broadly defined. Typically, religious fasting ~~also~~ includes spiritual activities to improve cognitive function and well-being.

Answer	n	%	% w/o A007
Strongly Agree (AO01)	11	**33.33%**	**34,38%**
Agree (AO02)	19	**57.58%**	**59,38%**
Neutral (AO03)	1	3.03%	3,13%
Disagree (AO04)	0	0.00%	0,00%
Strongly Disagree (AO05)	0	0.00%	0,00%
This definition is irrelevant and should be excluded from the consensus process (AO06)	1	3.03%	3,13%
Due to my specialization, I am not familiar with this term / this method and prefer not to vote on it (AO07)	1	3.03%	

AGREE: further thoughts

P14: Final sentence needs to include some aspect of spiritually, as this is really the whole point of religious fasting. I would consider: *Typically, religious fasting also includes spiritual activities to* ***improve one's relationship with God, in addition to*** *cognitive function and well-being.*

P13: I wonder if a slight rewording of the last sentence to: "*Typically, religious fasting also includes spiritual activities* ***aimed at improving cognitive function and well-being***." Would make this **sound more neutral** (I.e., not confirming nor denying any potential cognitive or well-being benefits, which is outside the scope of our work).

P18: In Christian Orthodox tradition, a 60 hour dry fasting practiced once a year.

"Statheropoulos M, Agapiou A, Georgiadou A: Analysis of expired air of fasting male monks at Mount Athos. J Chromatogr B Analyt Technol Biomed Life Sci 2006;832:274-279."

P09: In 131racticed orthodox tradition, a 60-hours dry fasting is 131racticed once a year[1]

[1]Statheropoulos M, Agapiou A, Georgiadou A: Analysis of expired air of fasting male monks at Mount Athos. J Chromatogr B Analyt Technol Bio med Life Sci 2006;832:274–279.

P05: I would delete the sentence "*Typically, religious fasting also includes spiritual activities to improve cognitive function and well-being*." Otherwise, it looks good to me.

NEUTRAL: suggested modifications / alternative definition

P33: this definition is fine and not v informative when discussing biological effects of different feeding patterns. However important to acknowledge the spiritual / social context

This definition is irrelevant and should be excluded from the consensus process.

P36: If we are participating in a scientific survey, then "religious fasting" should not be included since it does not mean anything and it could refer to nearly any type of fasting whether it is considered fasting or not based on the definitions set here

(If these definitions are based on science then religious fasting is irrelevant since even within a religion there may be many different ways to do a particular type of fasting. Thus, if fasting is defined, then religious fasting is simply any form of fasting done for religious purposes. To define "religious fasting" which is undefinable, makes this process not very scientific and would undermine its purpose but also the participation by scientists)

FX-MAYR-THERAPY

FX-MAYR THERAPY – The following definition of FX-Mayr Therapy reached a consensus of **77.27%** in the **second** survey:

FX-Mayr-Therapy or **FX-Mayr Cure** refers to a 3-phase fasting regimen containing elements of water-only fasting, a very low-calorie diet with a training of "proper chewing", in order to help individuals (re-)gain their sense of satiety and an easily digestible diet towards the end of the treatment. The dietary intervention is accompanied by manual treatments focusing on the abdominal region.

Although consensus was reached for this definition, an expert on **FX-Mayr therapy** suggested a few more changes to the definition to bring it up to date.

To what extent you agree or disagree with the modified definition?

FX-Mayr-Therapy or **FX-Mayr Cure** refers to a ~~3-phase fasting~~ regimen containing elements of water-only fasting, a very low-calorie diet with a training of "proper chewing", in order to help individuals (re-)gain their sense of satiety and an easily digestible diet towards the end of the treatment. The dietary intervention is accompanied by bowel cleansing procedures and manual treatments focusing on the abdominal region.

	Answer	n	%	% w/o A007
	Strongly Agree (AO01)	7	21.21%	**31,82%**
	Agree (AO02)	15	45.45%	**68,18%**
	Neutral (AO03)	0	0.00%	
	Disagree (AO04)	0	0.00%	
	Strongly Disagree (AO05)	0	0.00%	
	This definition is irrelevant and should be excluded from the consensus process (AO06)	0	0.00%	
	Due to my specialization, I am not familiar with this term / this method and prefer not to vote on it (AO07)	11	33.33%	
**STRONGLY AGREE - comments**				
P17:	... easily digestible diet generally with individualized components...			
**This definition is irrelevant and should be excluded from the consensus process.**				
P36:	this is a dangerous direction, particularly if it includes the word "cure". We have no way here to assess whether this is a therapy and much less a cure. The process would take months and the effort of lots of scientists and physicians who would have to determine if this particular method has in fact therapeutic effects			

Definitions that were discussed in the live online discussion, but which need to be re-evaluated in this fourth round:

Terms concerning dietary and caloric restriction	
**CALORIC RESTRICTION (CR)**	**CALORIC RESTRICTION (CR)**:<br>The following three definitions for **caloric restriction** were offered in the third survey:<br>Note on all three definitions: Total caloric intake here refers to the amount of calories required to maintain a person's "healthy" BMI – depending on their height and age.<br>**A1: Caloric restriction (CR)** describes a reduction* in energy intake below the total amount of calories that would be needed to maintain a person's current body weight, without causing malnutrition. If **CR** is done daily, it can also be referred to as **continuous energy restriction (CER)** or **daily energy restriction (DER).** *Note: amount of reduction is not specified in this definition.<br>**A2: Caloric restriction (CR)** describes a ≥ 10%* reduction in energy intake below the intake amount of calories that would be needed to maintain a person's current body weight, without causing malnutrition. If **CR** is done daily, it can also be referred to as **continuous energy restriction (CER)** or **daily energy restriction (DER).** *Note: we have chosen "≥ 10%" to combine the range options previously offered.<br>**A3: Caloric restriction (CR)** describes a reduction in energy intake below the total amount of calories that would be needed to maintain a person's current body weight, without causing malnutrition. The degree of caloric restriction should be decided individually depending on the person's age, sex, current body weight, occupation, goal & planned duration of CR. If **CR** is done daily, it can also be referred to as **continuous energy restriction (CER)** or **daily energy restriction (DER).**<br>**Distribution of votes:**<br>A1: ▶ **33.33 %** of the panel experts voted in favour of this definition.<br>A2: ▶ **13.33 %** of the panel experts voted in favour of this definition.<br>A3: ▶ **53.33 %** of the panel experts voted in favour of this definition.<br>As no consensus was reached, we talked about this term in the discussion. The participants decided to vote again on definitions A1 and A3 from the third round (see above).<br>**Distribution of votes in the discussion:**<br>A1: ▶ **53.33 %** of the panel experts voted in favour of this definition. (= 8/15 participants)<br>A3: ▶ **46.66 %** of the panel experts voted in favour of this definition. (= 7/15 participants)<br>As no consensus was reached in the discussion, these two definitions will be presented again in this round of the survey. Please indicate which one you prefer:<br>**A1: Caloric restriction (CR)** describes a reduction in energy intake below the total amount of calories that would be needed to maintain a person's current body weight, without causing malnutrition. If **CR** is done daily, it can also be referred to as **continuous energy restriction (CER)** or **daily energy restriction (DER).**<br>**A3: Caloric restriction (CR)** describes a reduction in energy intake below the total amount of calories that would be needed to maintain a person's current body weight, without causing

	malnutrition. The degree of caloric restriction should be decided individually depending on the person's age, sex, body composition, activity level, occupation, goal & planned duration of CR. If **CR** is done daily, it can also be referred to as **continuous energy restriction (CER)** or **daily energy restriction (DER).**

Answer	n	%
I agree with A1 (AO01)	9	27.27%
I agree with A3 (AO02)	24	72.73%
Neither of the above (AO03)	0	0.00%
This definition is irrelevant and should be excluded from the consensus process (AO04)	0	0.00%
Due to my specialisation, I am not familiar with this term / this method and prefer not to vote on it (AO05)	0	0.00%

I agree with A1

P13:	I think the portion of A3 in blue (stating that the degree of caloric restriction should be individualized) could be included as additional explanation in the manuscript without being part of the definition itself.
P05:	I still prefer the >= 10% description. Perhaps we could compromise and add a phrase "*Typically, CR involves a >=10% reduction in energy intake.*"
P33:	although a2 is strictly correct<br>in practice this doesn't often happen as hard i.e all pts with obesity are placed on 800 kcal total diet replacement diets
**TOTAL / COMPLETE FAST**	**TOTAL / COMPLETE FAST –** This term was talked about in the online discussion. Participants voted to include this term in the consensus process for use in studies where no calories are consumed during the fasting period. Inclusion seemed necessary because it was decided to use a broad definition for the term **fasting**. Dry fasting experts present in the online discussion supported a distinction between **total / complete fasting** and **dry fasting**.<br>To what extend do you agree or disagree with this definition?<br>The term **total fast**, or **complete fast**, refers to a fasting regimen, where no calories are consumed for a certain period of time.<br>Note: Total / complete fasting can be equated with water-only fasting.

Answer	n	%
Strongly Agree (AO01)	13	39.39%
Agree (AO02)	18	54.55%
Neutral (AO03)	0	0.00%
Disagree (AO04)	2	6.06%
Strongly Disagree (AO05)	0	0.00%
This definition is irrelevant and should be excluded from the consensus process (AO06)	0	0.00%
Due to my specialization, I am not familiar with this term / this method and prefer not to vote on it (AO07)	0	0.00%

STRONGLY AGREE: further thoughts

P17:	water, resp. tea only fasting

AGREE: further thoughts

P05:	I suggest rewording the definition more succinctly as: "*Total or complete fasting, **also known as water-only fasting**, refers to a fasting regimen where no calories are consumed for a certain period of time*." We still need to agree on the minimum number of hours that someone needs to be fasting for in order for something to constitute fasting.

DISAGREE: suggested modifications / alternative definition

P06:	I suggest to be a bit more flexible, and to consider the possibility **to include a minimum of caloric consumption (e.g., max 100 Kcal per day)**. In addition, a minimal duration of this regimen should be specified. As it is now, the definition is **too generic**. I suggest the following definition: "*The term total fast, or complete fast, refers to a fasting regimen, where a maximum of 100 Kcal/day is consumed for at least 24 consecutive hours*.
P37:	The definition should explicitly say **water is allowed.**
**FLUID-ONLY FASTING**	**FLUID-ONLY FASTING** – In the second round we offered two definitions for this term. Neither definition reached consensus.<br>In the second round, we also offered a definition for the term **gruel fasting**, as this is a traditional **therapeutic fasting regimen** practiced in Germany. Since neither consensus (**agreement votes: 50.00 %**), nor exclusion (**exclusion votes: 42.31 %)** was reached for this definition, we suggest to simply include the term gruel fasting with a note in the definition of **fluid-only fasting**.

	In the discussion, it was decided that the definition for **fluid-only fasting** should only allow for “non ultra-processed fluids / liquids” such as water, tea, & juices with no added sugars.<br><br>Please note the following:<br>• *Regarding the reasoning of fluid-only fasting*: Drinking liquids such as vegetable broth, gruel (from oats, flaxseed) and juices is permitted in the tradition of fluid-only fasting in Germany and has its background in supporting the fasting person physiologically and psychologically to abstain from food.<br>• *Regarding coffee and honey*: We removed coffee and honey from the definition. Both can be consumed during fluid-only fasting but are not an essential part of this regimen. This will be mentioned in the results section of the publication.<br>To what extend do you agree or disagree with the following definition?<br>**Fluid-only fasting** refers to a fasting regimen, whereby only beverages are consumed for a certain period of time. Water and unsweetened herbal tea may be consumed *ad libitum*. Clear vegetable broth, vegetable and/or fruit juices can be consumed up to a total of 500 kcal per day*. Ultra-processed fluids should not be consumed.<br>*based on the Expert Panel Update of the 2002 Consensus Guidelines for Fasting Therapy from 2013.<br>Note: This definition includes traditional fasting regimens that use various broths, gruel, or decoctions, such as the traditional German gruel fasting.

Answer	n	%	% w/o A007
Strongly Agree (AO01)	10	30.30%	31,25%
Agree (AO02)	15	45.45%	46,88%
Neutral (AO03)	2	6.06%	6,25%
Disagree (AO04)	3	9.09%	9,38%
Strongly Disagree (AO05)	0	0.00%	0,00%
This definition is irrelevant and should be excluded from the consensus process (AO06)	2	6.06%	6,25%
Due to my specialization, I am not familiar with this term / this method and prefer not to vote on it (AO07)	1	3.03%	

NEUTRAL: suggested modifications / alternative definition	
P33:	not sure you need the ultra processed
P37:	N/A
**DISAGREE: suggested modifications / alternative definition**	
P23:	this definition captures both fasting and modified fasting regimens. Therefore, I would prefer to define this as *Fluid-only fasting refers to a fasting regimen, whereby **no solid food calories and only zero calorie beverages** are consumed.*
P35:	If you are consuming up to 500 calories a day you are feeding and not fasting. This is a modified feeding program and calling it fasting is misleading.
P20:	Consumption of fruit juices will completely negate any benefit of ‘fasting’ and should be removed.

**MODIFIED FASTING**	**MODIFIED FASTING –** the following definition reached a consensus of **75.76%** in the second survey: The term **modified fasting** refers to limiting energy intake to 20-25% of energy needs on fasting days. Modified fasting regimens are often adapted to specific clinical settings and indications, allowing for different complementary or supportive therapeutic interventions. However, some participants suggested several changes for this definition that were addressed in the discussion. It was decided to **define modified fasting in the broadest sense** (similar to the definition of fasting) to include all types of modified fasting diets (such as FMD, diets with 2 separate or consecutive fasting days per week, ADMF). We decided to adapt the definition of **modified fasting** as follows: Please indicate to what extend you agree or disagree with this definition: The term **modified fasting** refers to limiting energy intake, typically up to (…)* % of energy needs on fasting days. Modified fasting regimens encompass alternate-day modified fasting, fasting practiced on 2 separate or consecutive days per week and the fasting-mimicking diet. These regimens are often adapted to specific clinical settings and indications, allowing for different complementary or supportive therapeutic interventions. *Note that "(…)" will be replaced by the threshold value selected below.

Answer	n	%	% w/o A007
Strongly Agree (AO01)	6	15.62%	18,75%
Agree (AO02)	20	62.50%	62,50%
Neutral (AO03)	1	3.12%	3,13%
Disagree (AO04)	3	9.38%	9,38%
Strongly Disagree (AO05)	1	3.12%	3,13%
This definition is irrelevant and should be excluded from the consensus process (AO06)	1	3.12%	3,13%
Due to my specialization, I am not familiar with this term / this method and prefer not to vote on it (AO07)	1	3.12%	

AGREE: further thoughts

P05:	I prefer shorter and simpler definitions. I recommend **deleting the words**: "*These regimens are often adapted to specific clinical settings and indications, allowing for different complementary or supportive therapeutic interventions.*" It's not an important enough idea to be part of the definition. The rest of the definition looks great.
Brandhorst:	[…] typically up to **75%** of energy needs […]
P01:	I´m **unsure how to categorize Buchinger and Mayr Fasting here**. They have about 200 up to 500kcal but are not bound to the macronutrient definition of FMD. Should we then include them as "*specific traditional medical methods as Buchinger- and Mayr-type fasting also belong ...* If we have a threshold like 25% then it might be no more comply with the FMD

NEUTRAL: suggested modifications / alternative definition

P02:	Would put up to **50%** of energy needs

DISAGREE: suggested modifications / alternative definition

P32:	Should **not include FMD**
P33:	this should be **intermittent** modified fasting refers to limiting energy intake. *Modified fasting regimens encompass alternate-day modified fasting, fasting practiced on a number of separate or consecutive days per week and fasting-mimicking die**ts***.(likely to be more than one!) i.e ***intermittent modified fasting*** *refers to limiting energy intake. Modified fasting regimens encompass alternate-day modified fasting, fasting practiced on a number of separate or consecutive days per week and fasting-mimicking diets.(* **likely to be more than one**!)
P20:	I think that eating whenever you like during modified fasting instead makes it an IER diet. If low carbohydrate foods, or prolonged consecutive fasting periods, then modified-fasting is appropriate.

STRONGLY DISAGREE: suggested modifications / alternative definition

P36:	**if fmd is included then the 35% calorie limit is not appropriate**

This definition is irrelevant and should be excluded from the consensus process

P25:	to me this is just being on a diet and **doesn't warrant its own terminology**

**MODIFIED FASTING**	Please choose which **threshold value for the allowed percentage of energy** requirements (= kcal intake) on modified fasting days you prefer:<br>• Maximum energy intake of 25% of energy requirements<br>• Maximum energy intake of 35% of energy requirements<br>One participant suggested that a table be added to the definition showing the absolute kcal amount to which the percentage of energy requirements may correspond. You can see the example table in the next box.

Answer	n	%	% w/o A007
A1: Maximum energy intake of 25% of energy requirements (AO01)	19	57.58%	61,29%
A2: Maximum energy intake of 35% of energy requirements (AO02)	6	18.18%	19,35%
Neither of the above (AO03)	3	9.09%	9,68%
This definition is irrelevant and should be excluded from the consensus process (AO04)	3	9.09%	9,68%
Due to my specialization, I am not familiar with this term / this method and prefer not to vote on it (AO05)	2	6.06%	

Neither of the above – comments:

P23:	I would say that the max number of calories of a **FMD (1400)** should be the max number of calories of a modified fasting regimen, **since FMDs are included in the definition**
P02:	Up **to 50%** like has been done in many research RCTs including ours
P36:	**if fmd is included then the 35% calorie limit is not appropriate**

This details is irrelevant– comments:

P33:	I think we can specify- this seems pointless and will be context specific

MODIFIED FASTING

MODIFIED FASTING – One participant suggested that we supplement the definition with a table showing what absolute kcal amount the percentage of energy requirement can correspond to, to give readers of our guidelines an idea of what kcal amounts (depending on age, body composition, and activity level) can be consumed. This participant kindly created such a sample table, which we have attached to the invitation email. If you are unable to open it, please let us know.

For a better understanding of the table below, please read the expert's notes:

- The table includes a **range of body masses (from 40 to 180 kg, in increments of 10 kg)** and a **range of activity factors (from 1.0 to 2.4)**. Both of these ranges are quite broad to encompass the vast majority of possibilities.
- Since height is required in the equation but exerts a fairly small effect on the estimates, height values corresponding to the 50th percentile for females and males provided by the WHO are used. While this is technically for young adults (19 years old), using the **50th percentile** values **at age 19** is reasonable for the purposes of this demonstration. These values were **163.2 cm for females and 176.5 cm for males**.
- Three versions of the table were created, **using ages 30, 50, and 70**.
- **Both sexes** are included for each body mass, activity factor, and age value.
- The **first three sheets show kcal values representing 25% of estimated weight-maintenance needs**. For reference, the **last three sheets show the kcal values for weight maintenance needs** (I.e., the predicted resting energy expenditure times the activity factor specified).

ALTERNATE-DAY FASTING (ADF)

ALTERNATE-DAY FASTING – In the **second** survey we offered two definitions for the term alternate-day fasting. Neither definition reached consensus. This term was therefore discussed in the online discussion.

In the discussion, it was decided to create two different definitions for alternate-day fasting and alternate-day modified fasting.

To what extend do you agree or disagree with this definition?

Alternate day fasting (ADF) refers to alternating a day of eating *ad libitum* and **a day of water-only fasting**.

Answer	n	%
Strongly Agree (AO01)	13	39.39%
Agree (AO02)	18	54.55%
Neutral (AO03)	0	0.00%
Disagree (AO04)	2	6.06%
Strongly Disagree (AO05)	0	0.00%
This definition is irrelevant and should be excluded from the consensus process (AO06)	0	0.00%

Answer	n	%
Due to my specialization, I am not familiar with this term / this method and prefer not to vote on it (AO07)	0	0.00%

DISAGREE: suggested modifications / alternative definition

P08:	Please avoid ad libitum terminology
P11:	In general people **don't perform water only** on fasting days, but **prefer fluid-only fasting**

ALTERNATE-DAY MODIFIED FASTING

ALTERNATE-DAY MODIFIED FASTING – In the second survey round, we asked you to select an energy equivalent for the allowed caloric intake on the **modified fasting days** in **alternate-day modified fasting (ADMF).** Since no consensus was reached on this issue, we included it in the discussion. It was decided to align the definition for **ADMF** with the general definition of **modified fasting**.

Please indicate which of the two definitions of ADMF you prefer:

A1: Alternate-day modified fasting (ADMF) refers to alternating a day of eating *ad libitum* and a day of modified fasting.
A2: Alternate-day modified fasting (ADMF) refers to alternating a day of eating ad libitum and a day of modified fasting with limited caloric intake at lunch or dinner.

Answer	n	%	% w/o A007
I agree with A1 (AO01)	20	60.61%	62.50%
I agree with A2 (AO02)	9	27.27%	28.13%
Neither of the above (I disagree with the given definitions) (AO03)	2	6.25%	6.25%
This definition is irrelevant and should be excluded from the consensus process (AO04)	1	3.12%	3.13%
Due to my specialization, I am not familiar with this term / this method and prefer not to vote on it (AO05)	1	3.12%	

I agree with A1 – comments:

P32:	**There is a Hoddy/Varady paper that showed it didn't matter when the meal was consumed.**
P02:	And modified fasting is up to 50% of energy needs

I agree with A2 – comments:

P20:	As above, eg if you drank 3 fruit juices throughout a modified fasting day for 500kcal, you are in no way fasting.

Neither of the above – comments:

P34:	modified fasting, is not fasting
P36:	alternate day fasting could include both the modified and not modified version

This definition is irrelevant and should be excluded from the consensus process

P25:	again, it's just like being on a diet on alternate days

Intermittent Fasting Regimens

INTERMITTENT ENERGY RESTRICTION (IER)

INTERMITTENT ENERGY RESTRICTION – In the third round, we offered two options:

A1 (wording from the second survey): **Intermittent Energy Restriction (IER)** includes periods of caloric restriction alternating with periods of *ad libitum* eating. As such, IER includes fasting regimens like intermittent fasting (IF) and time-restricted eating (TRE).

A2: Intermittent Energy Restriction (IER) includes periods of caloric restriction alternating with periods of ad libitum energy intake.

Distribution of votes:

A1: ▶**56.67 %** of the panel experts voted in favour of this definition.
A2: ▶**43.33 %** of the panel experts voted in favour of this definition.

Expert comments:

A2:

- I disagree with considering IER, IF and TRE as equivalent terms. **IF is a more extreme version of IER**, and in this respect it **can be incorpotared within IER.** However, **they cannot be considered as equivalent terms**, and a threshold of allowed calorie intake should be fixed to differentiate these two terms. IF and TRE can be considered as equivalent, provided that a minimum duration of fasting is specified for both approaches
- I think time restricted feeding should have its own definition to distinguish it from IER
- in the term 'TRE' there is not the term 'intermittent'
- I appreciate how straightforward A2 is, which is why it received my vote. However, this definition may leave it up to the reader **if they conceptualize intermittent fasting and time-restricted eating as programs that meet this definition (rather than explicitly stating that they do meet the definition, as in A1**). This could be fine, but I am not sure if that is consistent with the **purpose of the document**, which I think is **to reduce some of the ambiguity and confusion** regarding these terms.

	As **no consensus** was reached, we briefly talked about this term in the discussion. One participant offered to prepare a definition for the fourth survey.<br>To what extend do you agree or disagree with this definition=<br>**Intermittent Energy Restriction (IER)** includes periods of modified fasting alternating with periods of *ad libitum* energy intake.<br>Note: IER is a subcategory of intermittent fasting (IF) and includes regimens such as alternate-day modified fasting (ADMF), fasting on 2 separate or consecutive days per week, weeks of caloric restriction followed by weeks of eating in energy balance, and the fasting-mimicking diet.

Answer	n	%	% w/o A007
Strongly Agree (AO01)	9	27.27%	**29,03%**
Agree (AO02)	14	42.42%	**45,16%**
Neutral (AO03)	0	0.00%	0,00%
Disagree (AO04)	5	15.15%	16,13%
Strongly Disagree (AO05)	2	6.06%	6,45%
This definition is irrelevant and should be excluded from the consensus process (AO06)	1	3.03%	3.23%
Due to my specialization, I am not familiar with this term / this method and prefer not to vote on it (AO07)	2	6.06%	

**AGREE: further thoughts**	
P05:	I was the author of that definition. However**, I would change the words "fasting on 2 separate or consecutive days per week" to "5:2 diet"**.
**DISAGREE: suggested modifications / alternative definition**	
P13:	I think the definition of IER should be **modified to use "caloric restriction" rather than "modified fasting"**. For example: "*Intermittent Energy Restriction (IER) includes* ***periods of caloric restriction*** *alternating with periods of ad libitum energy intake*." If "modified fasting" is used, it would mean that an eating program would only qualify as IER if a large energy deficit is used (down to the maximal allowable % intake in whichever definition of modified fasting is approved). In contrast to this, I think many IER programs use smaller energy deficits (I.e., higher energy intake than would be allowed by modified fasting but less than weight maintenance energy needs). This is my reasoning for thinking "caloric restriction" would be more appropriate than "modified fasting" in the definition.<br>Even if that change wasn't made, I feel like the note may need to be revised**. I was surprised to see IER presented as a subcategory of IF within the note because the previous definition presented this relationship the opposite way (I.e., IF was a subcategory of IER**). My view is that IER is a broader concept (alternating ad libitum or weight maintenance calorie intake with periods of caloric restriction, as explained above).
P15:	**IER should include either fasting or modified fasting**
P34:	...a hard to reach agreement if we do include a modified fasting.........
P25:	remove ADM
P36:	if it **is intermittent energy restriction then it should refer to intermittent calorie restriction and not modified fasting**
**STRONGLY DISAGREE: suggested modifications / alternative definition**	
P33:	think the group has got v confused! We need a term for intermittent diets . The **term Intermittent Energy Restriction is an overarching term for modified fasting etc and not a sub group of this.** Suggest the modified fasting term above includes intermittent than this should be covered i.e ***intermittent modified fasting*** *refers to limiting energy intake. Modified fasting regimens encompass alternate-day modified fasting, fasting practiced on a number of separate or consecutive days per week and fasting-mimicking diets.(* **likely to be more than one**!)
P20:	**IF and TRE are a subcategory of IER, not the other way around**. All of these diets are IER – but if you do not have low carb/zero calories for a prolonged period in a day then you are not doing IF or TRE. **Of note, I agree IF is different from TRE (due to mechanism of action as noted on first page) and it shouldn't be placed under 'IF' but under 'IER'.**
**TIME-RESTRICTED EATING**	**TIME-RESTRICTED EATING –** In the third round we asked you which of the following two definitions of time-restricted eating you prefer:<br>**A1: Time-restricted eating (TRE)** is a dietary regimen in which food intake and the consumption of caloric beverages is restricted to a specific period of time (usually 1 to 10 hours) during the day, resulting in a daily fasting window of at least 14 hours. There is no explicit limit on energy intake during eating hours.*<br>*Note: Low frequency meal patterns such as the "**one meal a day diet (OMAD)**" can be regarded as part of TRE according to this definition.<br>**A2: Time-restricted eating (TRE)** is a dietary regimen in which food intake and the consumption of caloric beverages is restricted to a specific period of time (usually 4 to 10 hours) during the day,

resulting in a daily fasting window of 14 to 20 hours. There is no explicit limit on energy intake during eating hours.

Distribution of votes:

A1: ► **53.33 %** of the panel experts voted in favour of this definition.
A2: ► **43.33 %** of the panel experts voted in favour of this definition.
► **3.33 %** of the panel experts voted "neither of the above".

Experts' comments:

- *AGREE WITH A1:* As indicated by the note attached to the asterisk (for A1), inclusion of OMAD as part of TRE necessitates that the eating period could be as short as ~1 hour. I think this definition is preferable as **it is broader and encompasses virtually all TRE-style programs**, which I think **should be the goal of the definition**.
- *AGREE WITH A2:* what about the requirement to begin eating at least one hour after arising, and end at least 3 hours before going to sleep? Without that you don't have the full benefit of improving the circadian rhythm.
- *NEITHER OF THE ABOVE:* I suggest TRF if > fasting period to the average i.e > 12 hours

As no consensus was reached on the duration of the **fasting and eating windows in TRE**, we talked about this term in the discussion. The participants decided that it would be easiest to define only the minimum fasting duration without specifying the fasting or eating window. In this way, the **one-meal-a-day diet (OMAD)** can also be considered part of TRE.

We therefore decided to adjust the definition of TRE with the majority of votes (= A1) from the third round of the survey**.** To what extend do you agree or disagree with this definition?

Time-restricted eating (TRE) is a dietary regimen in which food intake and the consumption of caloric beverages is restricted to a specific period of time ~~(usually 1 to 10 hours)~~ during the day, resulting in a daily fasting window of at least 14 hours. There is no explicit limit on energy intake during eating hours.

Answer	n	%	% w/o A007
Strongly Agree (AO01)	16	48.48%	50,00%
Agree (AO02)	12	36.36%	37.50%
Neutral (AO03)	1	3.03%	3,13%
Disagree (AO04)	1	3.03%	3.13%
Strongly Disagree (AO05)	2	6.06%	6.25%
This definition is irrelevant and should be excluded from the consensus process (AO06)	0	0.00%	0.00%
Due to my specialization, I am not familiar with this term / this method and prefer not to vote on it (AO07)	1	3.03%	

AGREE: further thoughts

P25: would add: "*Ideally the period of time during which food is consumed should begin at least **one hour after arising and end at least 3 hours before going to sleep.** Non-caloric beverages can be consumed at any time.*"

P20: I prefer A2 but am ok with this.

NEUTRAL: suggested modifications / alternative definition

P13: Personally, I would vote to **remove the portion about the duration** and simply state:

Time-restricted eating (TRE) is a dietary regimen in which food intake and the consumption of caloric beverages is restricted to a specific period of time during the day ~~resulting in a daily fasting window of at least 14 hours~~. There is no explicit limit on energy intake during eating hours.

DISAGREE: suggested modifications / alternative definition

P33: *Time-restricted eating (TRE) is a dietary regimen in which food intake and the consumption of caloric beverages is restricted to a specific period of time **(usually 1 to 10 hours)** during the day ~~resulting in a daily fasting window of at least 14 hours~~, There is no explicit limit on energy intake during eating hours.*

STRONGLY DISAGREE: suggested modifications / alternative definition

P36: The **most important TRE is a 11-12 hour TRE.** This definition would exclude the most popular TREs and probably the safest

P37: It is **critical that it is a consistent eating window**. I provide the following definition:

*Time-restricted eating (TRE) is a dietary regimen that requires all energy intake to be consumed in a consistent daily **eating window of 6-10 hours** during the individuals habitual waking hours. Water, and occasionally other zero-calorie beverages, are allowed outside the eating window. There is now explicit limit on energy intake.*

[fasting window: 14-18h]

Note: **The main factors that differentiate TRE from IF are (1) TRE does not require caloric restriction, and (2) TRE does require a consistent daily eating window as it is developed in reference to circadian rhythms.**

Specific Fasting Regimens	
**THERAPEUTIC / MEDICAL FASTING**	**THERAPEUTIC / MEDICAL FASTING –** As no consensus was reached on this term in the second survey, but also no modifications were suggested, we talked about this term in the discussion: It was decided to allow a broad and brief definition of the term **fasting**, but to include the aspect of individualization of the fasting program in the definition of **therapeutic fasting**.<br>To what extend do you agree or disagree with this definition?<br>**Therapeutic fasting** refers to any fasting regimen that is applied as a therapeutic intervention. The **fasting regimen** should be decided individually depending on the person's age, sex, body composition, activity level, occupation, goal & planned duration of **fasting**.<br>**Medically supervised fasting** refers to any fasting regimen that is applied as a therapeutic intervention by a trained physician or similar credentialed healthcare provider.

Answer	n	%	% w/o A007
Strongly Agree (AO01)	11	33.33%	**34.38%**
Agree (AO02)	18	54.55%	**56.25%**
Neutral (AO03)	1	3.03%	3.13%
Disagree (AO04)	0	0.00%	0.00%
Strongly Disagree (AO05)	1	3.03%	3.13%
This definition is irrelevant and should be excluded from the consensus process (AO06)	1	3.03%	3.13%
Due to my specialization, I am not familiar with this term / this method and prefer not to vote on it (AO07)	1	3.03%	

AGREE: further thoughts

P18:	*Medically supervised fasting refers to any fasting regimen that is applied as a therapeutic intervention by a trained physician ~~or similar credentialed healthcare provider.~~*<br>The health care providers that are not trained physicians do not have the background to accommodate fasting patients with pre-existing health issues (e.g. diabetes mellitus, renal disease etc.)
P09:	*"Medically supervised fasting refers to any fasting regimen that is applied as a therapeutic intervention by a trained physician-~~or similar credentialed healthcare provider.~~"*<br>The health provider, who are not physician, do not have the background to supervise fasting patients, having various diseases.
P05:	I don't think the wording "*Therapeutic fasting interventions are individually tailored to a person's age, sex, body composition, physical activity level, occupation, goal & planned duration of fasting*" is needed. I would **delete it.**

STRONGLY DISAGREE: suggested modifications / alternative definition

P25:	I actually object to the term "therapeutic." To me it has too many connotations associated with poorly documented science.<br>In my mind, **fasting is done either for religious or health reasons**. If you're looking to differentiate those two, then I would say so. In other words, I would define religious fasting on the one hand and **health-related fasting** as the other definition. You could use the definition exactly as you have stated as long as you **change the terminology from "therapeutic" to "health-related".** It's not your definition that I am strongly opposed to; it's the classification.<br>The other reason I **object to the term therapeutic is that there is no guarantee it will in fact be therapeutic**. In fact it could even turn out to be detrimental (for example if a physician without adequate training in diabetes prescribed "therapeutic fasting" and the patient became hypoglycemic and could even die from it). Using a term like health-related doesn't imply any outcome.

Terms on which consensus has not yet been reached and which were not part of the CC:

**SHORT-TERM FASTING (STF)**	**SHORT-TERM FASTING** – In the second survey, we asked you to indicate what duration you consider appropriate for the term short-term fasting.

Based on the lack of consensus and the comments received, we propose the following definition:

Short-term fasting (STF) refers to a fluid-only fasting regimen with a duration of 1-3 days.

Answer	n	%	% w/o A007
Strongly Agree (AO01)	10	30.30%	31.25%
Agree (AO02)	16	48.48%	50.00%
Neutral (AO03)	2	6.06%	6.25%
Disagree (AO04)	4	12.12%	12.50%
Strongly Disagree (AO05)	0	0.00%	0.00%
This definition is irrelevant and should be excluded from the consensus process (AO06)	0	0.00%	0.00%
Due to my specialization, I am not familiar with this term / this method and prefer not to vote on it (AO07)	1	3.03%	

AGREE: further thoughts

P20: Calorie free fluid

P17: rather 1-7 days

NEUTRAL: suggested modifications / alternative definition

P34: no calories, water only

P02: Would limit to 1 or 2 days

DISAGREE: suggested modifications / alternative definition

P06: Short-term fasting (STF) **recalls the definition of fasting**, which was very generic and did not necessarily imply a reduction in calorie intake. Therefore, I would replace this definition with two different definitions:

*1. **Short-term fluid-only fasting** refers to a fluid-only fasting regimen with a duration of 1-3 days.*

*2. Short term **complete** fasting refers to a **complete** fasting regimen with a duration of 1-3 days.*

P22: should be 1-2 days

P33: should say short term fluid only fast to fit will all agreed definitions

P36: I would limit to 2 days, since day 3 signals a longer fasting which involves ketogenesis

PROLONGED FASTING (PF) / LONG-TERM FASTING:

LONG-TERM FASTING –In the second survey, we asked you to indicate what duration you consider appropriate for the term long-term fasting.

Based on the lack of consensus, we propose the following definition:

Prolonged fasting (PF), also called **long-term fasting (LTF)**, refers to any fluid-only fasting regimen lasting ≥ 4 consecutive days.

Answer	n	%	% w/o A007
Strongly Agree (AO01)	10	30.30%	31.25%
Agree (AO02)	16	48.48%	50.00%
Neutral (AO03)	1	3.03%	3.13%
Disagree (AO04)	5	15.15%	15.63%
Strongly Disagree (AO05)	0	0.00%	0.00%
This definition is irrelevant and should be excluded from the consensus process (AO06)	0	0.00%	0.00%
Due to my specialization, I am not familiar with this term / this method and prefer not to vote on it (AO07)	1	3.03%	

AGREE: further thoughts

P34: as long as the “fluid” isn’t caloric

P05: I’m fine with either a 3, 4, or 5-day minimum definition to distinguish between short-term vs. long-term fasting. I don’t feel strongly on this point. In order to achieve consensus, you can count my vote towards what the majority votes for.

P20: Calorie free fluid

P17: more than 7 days

NEUTRAL: suggested modifications / alternative definition

P02: Would have it at least 3 days

DISAGREE: suggested modifications / alternative definition

P06: Long-term fasting (LTF) **recalls the definition of fasting**, which was very generic and did not necessarily imply a reduction in calorie intake. Therefore, I would replace this definition with two different definitions:

1. Long-term **fluid-only** fasting refers to a fluid-only fasting regimen with a duration of ≥ 4 consecutive days.

	2. Long-term complete fasting refers to a complete fasting regimen with a duration of ≥ 4 consecutive days.
P30:	The term “long-term fasting” should be used for a time span of at least **10-14 days.**
P33:	should be prolonged fluid only fasting
P01:	"**fluid only" should not be worded**. For example, Mayr and gruel fasting are not fully fluid fasting but belong to the prolonged fasting methods
P36:	I would go with 3 days or longer. see above

**PERIODIC FASTING (PF)**	**PERIODIC FASTING –** In the **second** survey we offered two definitions for the term **periodic fasting.**

As no clear favorite emerged, we offer the definition with the majority of votes from the **second** round again.Please also take note of the definitions of “periodic” from the Cambridge, Oxford and Merriam Webster`s dictionaries.

“PERIODIC” according to dictionaries:

	Cambridge	Merriam-Webster	Oxford Dictionary of English
Adjec-tive	happening repeatedly over a period of time;<br>*American Cambridge:*<br>(=repeated event) SCIENCE – happening repeatedly in a fixed pattern;<br>(=time) happening repeatedly although not necessarily frequently;<br>PHYSICS – A pattern or other event is periodic if it repeats at periods of time that are equal	occurring or recurring at regular intervals; occurring repeatedly from time to time;<br>consisting of or containing a series of repeated stages, processes, or digits: CYCLIC	Appearing or occurring at intervals

To what extend do you agree or disagree with this definition?

Periodic fasting (PF) refers to any fasting regimen that is repeated at regular intervals (periods), such as every day, every week, or every several months.

Note: According to this definition, periodic fasting would include intermittent fasting regimens.

Answer	n	%	% w/o A007
Strongly Agree (AO01)	11	33.33%	34.38%
Agree (AO02)	15	45.45%	46.88%
Neutral (AO03)	1	3.03%	3.13%
Disagree (AO04)	5	15.15%	15.63%
Strongly Disagree (AO05)	0	0.00%	0.00%
This definition is irrelevant and should be excluded from the consensus process (AO06)	0	0.00%	0.00%
Due to my specialization, I am not familiar with this term / this method and prefer not to vote on it (AO07)	1	3.03%	

NEUTRAL: suggested modifications / alternative definition

P02:	Take out “such as every day”

DISAGREE: suggested modifications / alternative definition

P32:	I utilize periodic fasting as more of a **monthly basis of feeding** and fasting rather than day to day. I would not say that ADF or TRE are periodic fasting but that something like the fasting mimicking diet as periodic fasting. I thought Longo came up with Periodic fasting term **to separate FMD to IF**.
P23:	*Periodic fasting (PF) refers to any fasting regimen that* ***lasts at least 4 days and is*** *repeated at regular intervals (periods).*
P33:	this term is not useful<br>should have an episodic term to describe something which happens less regularly. I have always used for something which happens for a few months every few months and not a daily weekly thing i.e low cal diets for 3 months and 3 months off and then repeat as with the tempo study etc
P20:	This doesn’t differentiate from intermittent fasting. PF reflects Merriam Webster to be more cyclical ie 4 days per month.
P36:	Periodic Fasting should refer to **any form of prolonged fasting whether is at regular intervals or not**, excluding all of the frequent intermittent fasting interventions. For example, periodic fasting could include any fasting/FMD of 3 days or longer done less than once/week or once/2 weeks, but which could also occur **once a year without any particular interval**

In the last survey, consensus was reached on one term

This page is for your information only.

**INTERMITTENT DRY FASTING:**	**INTERMITTENT DRY FASTING** – As no consensus was reached in the second round, we offered the following definition for **intermittent dry fasting** proposed by one of the experts in the third round:<br>**Intermittent dry fasting (IDF)** refers to intermittent fasting regimens that involve abstaining from food and fluid intake during fasting hours. Most commonly, they range from 9 to 20 hours.<br>**Distribution of votes:**<br>► **85.19 %** of the panel experts voted in favour of this definition.<br>► **0.00 %** of the panel experts voted neutral.<br>► **7.41 %** of the panel experts selected "disagree / strongly disagree".<br>► **7.41 %** of the panel experts voted that this definition is irrelevant.<br>With the reached consensus of **85.19 %,** the definition will be included in the publication as is.

In the live online discussion, consensus was reached on the following terms

This page is for your information only. Please note that the following are terms that already reached a consensus in one of the last survey rounds but had to be re-evaluated due to the participants' comments.

**General terms concerning fasting**	
**FASTING**	**FASTING –** In the online discussion we asked the participants the following questions:<br>• How should the term **fasting** be defined? – Should it equal complete abstention from ALL foods and calorie-containing beverages?<br>• What about the fasting regimens that do not require zero-calorie intake per se, but have been called fasting in the past? (e.g. religious fasting, fluid-only fasting, therapeutic fasting)<br>Some of the participants' opinions:<br>• **"fasting" = zero intake of calories; "modified fasting" = low intake of calories; have a third term (as a subtype of fasting) = dry fasting;**<br>**reasoning: there are cultural differences based on countries; problem could be degree of confusion that is created when someone says fasting and by definition we allow zero calorie intake AND a certain amount of calories, e.g. 25% of their caloric intake; the public as well as practitioners might get confused with what they (patients) are doing / not doing;**<br>• The definition should include: **maximum amount of kcal of day –** proposal: max. amount of 200(-500) kcal on fasting day & **minimum amount of duration of fasting** – proposal: minimum duration of 20 hrs; There could be two terms: "total fasting" = zero calories and "fasting" = allowing for some kcal (threshold should be discussed)<br>• the term "fasting" used to be used in Germany also for zero-calorie-diets BUT the aim was then to not propogate zero-calorie diets to avoid protein catabolism; for this reason modified fasting was introduced; in general is logical to say "fasting" = zero kcal, "modified fasting" = some kcal allowed<br>• when we talk about "fasting" we need to remember that "fasting" is an old word. In the history of fasting various forms have been employed, and these forms have to be included in the definition of "fasting". Zero-kcal diet was never a part of fasting except for in the last decades; 2500 years ago there were no zero kcal diets or zero-kcal beverages, they didn't exist<br>• From a methodological perspective: if we want to define "fasting" – since there are different kinds of practices – the common features and characteristics must be found and listed, so the definition should be somewhat broad. I also agree with the definition A2. Based on this definition, we can then articulate the different types of fasting.<br>We then ran a poll on the following definition of **fasting** that already reached a consensus of **84.85 % in the second survey:**<br>**A2: Fasting** refers to a voluntary abstinence from some or all foods or foods and beverages for preventive, therapeutic, religious, cultural, or other reasons.<br>**Distribution of votes:**<br>►**68.75 % (=11/16 participants)** of the discussion participants voted in favour of definition A2.<br>►**18.75 % (=3/16 participants)** of the discussion participants voted for a more specific definition.

	(e.g., stating kcal amount / minimum duration of fasting).<br>►**9.09 % (= 2/16 participants)** of the discussion participants didn't vote.<br><br>It has been decided to use a broad definition of **fasting** that includes ALL types of fasting. Terms such as **total / complete fasting** and **modified fasting** are defined to make it clear in publications which "subtype of fasting" is being studied.
**DRY FASTING (DF)**	**DRY FASTING –** definition from the **second** survey:<br><br>**Dry Fasting** refers to a fasting regimen, during which a voluntary abstinence from all foods and beverages, including water, is practiced for a certain period of time.<br><br>The above definition reached a consensus of **87.50%** in the second round. However, some dry fasting experts commented that the terms **total / complete fasting** are equivalent to **dry fasting**. For this reason, we included the terms total/complete fasting in the definition of dry fasting in the third round as follows:<br><br>**Dry Fasting**, also called **total fasting / complete fasting**, refers to a fasting regimen, during which a voluntary abstinence from all foods and beverages, including water, is practiced for a certain period of time.<br><br>Although a consensus of **74.08%** was reached for this revised definition in the third round, several changes were proposed.<br>Experts' comments from the third survey:<br>*DISAGREE*<br>• I don't like the addendum in blue, because complete or total fasting to me seems absence of food and drinks, but not from water<br>• Commonly, in these times, The term total is used for zero diet (but with water or tea intake) If we choose the above proposed definition a lot of published studies would be misinterpreted<br>*STRONGLY DISAGREE:*<br>• Dry fasting should not be referred to as total/complete fasting as that will create confusion on the well-established practice of referring to water-only fasting as complete or total fasting.<br>• I strongly disagree with considering "dry fasting" as equivalent to "total/complete fasting". Indeed, while these types of fasting could be similar in terms of calorie intake restriction, there are huge physiological and therapeutic differences between these two approaches. In particular, while "total/complete fasting" can be safe, well tolerated and, potentially, therapeutically effective even when prolonged (i.e., when lasting for 2 or more days), "dry fasting", which also implies total abstinence from the intake of beverages, including water, is potentially harmful, in that it can cause hypotension, dehydration, damage to the kidneys, blood electrolyte imbalance and other severe side effects.<br>• *Dry Fasting refers to a fasting regimen, during which a voluntary abstinence from all foods and beverages, including water, is practiced for a certain period of time.*<br>The comments and proposed changes were discussed in the online discussion. Participants decided that **dry fasting** should not be synonymous with **total / complete fasting**. Therefore, the terms total / complete fasting will be used for a different definition and thus won't be included as synonyms in the final definition of dry fasting. The final definition of dry fasting will be the one from the second survey (see above), which reached a consensus of **87.50%.**
**INTERMITTENT FASTING (IF)**	**INTERMITTENT FASTING** – definition in the third survey:<br><br>**Intermittent fasting (IF)** refers to repetitive fasting periods lasting up to 48 h each. IF includes fasting regimens of 1 fasting day per week, 2 separate or consecutive fasting days per week, alternate-day fasting (ADF), and time-restricted eating (TRE).<br><br>**Distribution of votes:**<br><br>► **86.67 %** of the panel experts voted in favour of this definition.<br>► **13.33 %** of the panel experts selected "disagree / strongly disagree".<br>Although the above definition reached a consensus of **86.67 %**, changes were proposed.<br>Experts' comments:<br>• I don't like the addendum in blue, because complete or total fasting to me seems absence of food and drinks, but not from water<br>• *AGREE*: I disagree with implications of the statement: TRE has a different mechanism of action (restoring circadian rhythms) than IF (body uses ketones for energy) and should therefore not be lumped together. (1) TRE has multiple mechanisms, not just restoring circadian rhythms (too narrow). (2) This statement assumes that the most important feature of fasting is the production of ketones. History, IF has not been defined by the production of ketones. Historically, definitions have focused on the length of the fasting period, not the underlying mechanisms (which are still being understood).<br>• *DISAGREE*:would have preferred to keep them separate BUT if you do combine them, then at least give a brief description of TRE in the definition "(limiting food intake to 6-12 hours per 24 hour period)"

	• *DISAGREE: Intermittent fasting refers to repetitive fasting periods applied **on a per week basis**, alternated with periods of ad libitum food intake. / Intermittent fasting (IF) refers to repetitive fasting periods. IF includes diurnal fasting regimens of 1 fasting day **per week**, 2 separate or consecutive fasting days **per week**, successive- or alternate-day fasting.*<br>• STRONGLY DISAGREE: we need to describe the different patterns of the diet time restricted feeding is very different to intermittent fasting and should have its own definition.<br>Based on the comments in the last survey, we wanted to know from participants in the online discussion whether **time-restricted eating** should now be considered a subcategory of **intermittent fasting**. The participants felt that this should be the case because **time-restricted eating** is **repetitive** in nature and has a **minimum fasting duration of 14 hours** per day (intermittent fasting includes fasting regimens with a repetitive fasting period of up to 48 hours). Therefore, the above definition of intermittent fasting is included in the publication without changes.

Fifth Questionnaire

Defining Fasting: Finding Common Ground Using the Delphi Method - Round 5

Please read the following information thoroughly before starting the fourth questionnaire.

Since this is the final round, please indicate only those changes that seem central to you. We will not be able to include these changes in the final definitions because we cannot ask the panel for feedback again, but they will be included in the discussion section of the joint publication.

As a reminder:
We modified the fasting terms and definitions according to your suggestions. In the definitions you will see crossed out words in red and newly added words underlined in blue to make the modifications visible. We also provide you with single or summarized comments of the panel experts in anonymized form, as well as the distribution of votes from the last round, to help you decide. However, in order to make this survey as compact as possible, not all comments are listed. (We are happy to send you **a document with all comments on request**).

Evaluation rules

We would like to inform you that it was necessary to add a final evaluation rule (see rule 11 below).

11. If no consensus is reached for a term by the end of the fifth round, the last version that reached consensus will be used. If there was no previous consensus, this result will be described in the publication.

Guide for the colors & italics in the comments

- Comments that occur more than once are highlighted in orange / light blue / purple
- Text in italics: alternative definitions for terms
- Purple background: alternative names for described definitions
- Terms highlighted in green: reached an agreement ≥ 70 and were accepted as is
- Terms highlighted in dark green: didn't reach an agreement in this round but in a previous round

---

Conflict of interest and demographics	
COI	For the upcoming joint publication, we would like to obtain more information to describe the panel.<br>Please disclose all relationships or interests that could inappropriately influence or bias your work.<br>**Financial interests:** These may include but are not limited to membership, employment, consultancies, stocks/shares ownership; honoraria; grants or other funding; paid expert testimony and patent-licensing arrangements.<br>**Non-financial interests:** These may include but are not limited to personal relationships or competing interests directly or indirectly tied to this research, or professional interests or personal beliefs that may influence your research.

**Age**	Average: 53.26
**Gender**	How do you identify?<br>• Woman<br>• Man<br>• Genderqueer / Non-binary<br>• Other:<br>**Distribution of votes:**<br>Woman: ▶ **34.21 % (13 participants)**<br>Man: ▶ **63.16 % (24 participants)**<br>Genderqueer / Non-Binary: ▶ **2.63 % (1 participant)**
**Field of study / subject**	In what field of study or subject did you obtain your highest qualification? (you can check more than one box)<br>• Nutritional Sciences ▶ **26.32% (10 participants)**<br>• Clinical Nutrition ▶ **18.42% (7 participants)**<br>• Dietetics and Nutrition ▶ **15.79% (6 participants)**<br>• Medicine ▶ **50.00% (19 participants)**<br>• Biology ▶ **15.79% (6 participants)**<br>• Molecular Biology ▶ **5.26% (2 participant)**<br>• Molecular Medicine ▶ **5.26% (2 participant)**<br>• Health Sciences ▶ **15.79% (6 participants)**<br>• Other: ▶ **47.37% (18 participants)**<br>Other: **Nutrutional Epidemiology, Physiology, Physiological chemistry, Medical Oncology, Theology, Endocrinology, Circadian Biology, Exercise Physiology, Genetic Epidemiology, Kinesiology, Genetic Epidemiology, DC/DO, Neuroscience / Chronobiology, Physics**
**Continent of origin**	• Asia ▶ **5.26% (2 participant)**<br>• Australia ▶ **5.26% (1 participants)**<br>• Europe ▶ **52.63% (20 participants)**<br>• North America ▶ **34.21% (13 participants)**<br>• South America ▶ **0.00% (0 participants)**<br>• Africa ▶ **2.63% (1 participants)**
**Continent of employ-ment**	• Asia ▶ **5.26% (1 participants)**<br>• Australia ▶ **2.63% (1 participants)**<br>• Europe ▶ **42.11% (16 participants)**<br>• North America ▶ **47.37% (18 participants)**<br>• South America ▶ **0.00% (0 participants)**<br>• Africa ▶ **2.63% (1 participants)**
**Researcher / Clinician (scientist)**	Are you a researcher, clinician scientist or clinician?<br>• **Researcher ▶ 50.00% (19 participants)**<br>• **Clinician scientist ▶ 26.32% (10 participants)**<br>• **Clinician ▶ 21.05% (8 participants)**<br>• **Other ▶ 2.63% (1 participant) = Assistant Professor**
**Main focus of work**	Is fasting the main focus of your work?<br>• Yes ▶ **55.26% (21 participants)**<br>• No ▶ **44.74% (17 participants)**
**Years involved in fasting research**	• 1-5 years ▶ **2.63% (1 participants)**<br>• 5-10 years ▶ **26.32% (10 participants)**<br>• 10-15 years ▶ **21.05% (8 participants)**<br>• 15 or more years ▶ **47.37% (18 participants)**<br>• Other: 38 years ▶ **2.63% (1 participant)**

Additional information in the form of "Comments"	
Comments	When additional information about a definition was important but did not seem relevant to the understanding of the term, we added a "Note" to the definition. We decided to change the wording from "Notes" to "Comments".<br>An example of such an additional "comment" is found in the definition of **caloric restriction**: consensus was reached on the following definition, but it was mentioned that the sentence highlighted in blue doesn't need to be part of the definition itself, but could rather be included in the publication as an additional explanation of the term:<br>**Caloric restriction (CR)** describes a reduction in energy intake below the total amount of calories that would be needed to maintain a person's current body weight, without causing malnutrition. The degree of caloric restriction should be decided individually depending on the person's age, sex, body composition, physical activity level, occupation, goal & planned duration of CR. If CR is done daily, it can also be referred to as continuous energy restriction (CER) or daily energy restriction (DER).<br>*Proposal:*<br>**Caloric restriction (CR)** describes a reduction in energy intake below the total amount of calories that would be needed to maintain a person's current body weight, without causing malnutrition. If CR is done daily, it can also be referred to as continuous energy restriction (CER) or daily energy restriction (DER).<br>Comment: The degree of caloric restriction should be decided individually depending on the person's age, sex, body composition, physical activity level, occupation, goal & planned duration of CR.<br>Do you agree wit TEM h adding additional information in the form of "comments"? If you would like to suggest another term for it or have another solution, please let us know in the comment field.<br>• Yes, I agree<br>• No, I disagree<br>• I have another solution / would like to propose a different term<br>**Distribution of votes:**<br>A1: ▶ **93.10 %** of the panel experts voted "Yes, I agree".<br>A2: ▶ **3.45 %** of the panel experts voted "No, I disagree".<br>A3: ▶ **3.45 %** of the panel experts voted "I have another solution / would like to propose a different term".
**No, I disagree**	
P33:	Don't think its needs anything adding. I WOULD JUST KEEP THE SIMPLE STATEMENT *Caloric restriction (CR) describes a reduction in energy intake below the total amount of calories that would be needed to maintain a person's current body weight, without causing malnutrition.* energy requirements are estimated as stated but can be measured more accurately. Don't think we want to get into this. Also, as soon as you energy restrict your energy requirements reduce by around 10%, again too complex to get in to. Do not agree with the statement that the duration of CR impacts on its severity. The degree of CR will depend on what you are trying to achieve and the population.Since we are saying not causing malnutrition, will need to say something about meeting minimal nutritional requirements for protein, micronutrients, essential fatty acids and carbohydrate
**I have another solution:**	
P06:	I would propose a small integration to the proposed definition to exclude from the definition of calorie restriction any changes in the intake of liquids/fluids that could result in a reduction in patient body weight not because of reduced calorie intake, but as a result of dehydration: *Caloric restriction (CR) describes a reduction in energy intake below the total amount of calories that would be needed to maintain a person's current body weight, without causing malnutrition **and dehydration**. If CR is done daily, it can also be referred to as continuous energy restriction (CER) or daily energy restriction (DER).*

Terms on which consensus has been reached in the last survey, but which need to be re-evaluated based on comments from participants

Terms concerning dietary and caloric restriction	
TOTAL / COMPLETE FAST	**TOTAL / COMPLETE FAST –** In the **fourth** survey, we offered the following definition:<br>The term **total fast**, or **complete fast**, refers to a fasting regimen, where no calories are consumed for a certain period of time.<br>Note: Total / complete fasting can be equated with water-only fasting.<br>**Distribution of votes:**

	▶ **93.94 % of the panel experts voted in favour of this definition.**<br>▶ **0.00 %** of the panel experts voted neutral.<br>▶ **6.06 %** of the panel experts selected “disagree / strongly disagree”.<br>▶ **0.00 %** of the panel experts voted that this definition is irrelevant.<br><br>Although consensus was reached for this definition, it was proposed to specify the **minimum duration** of this regimen. The suggested minimum duration was **24 hours**. We have discussed this in our team and feel that specifying the duration isn't necessary to describe the "quality" of this type of fast, which consists of not consuming calories. For reference, here is the already **accepted** definition of **water-only fasting**: **Water-only fasting** refers to a fasting regimen, where only water is consumed for a certain period of time.<br><br>Please select either the initial definition A1 or definition A2 with the duration:<br><br>**A1** (initial wording)**:** The term **total fast**, or **complete fast**, refers to a fasting regimen, where no calories are consumed for a certain period of time.<br>Comment: Total / complete fasting can be equated with water-only fasting.<br><br>**A2:** The term **total fast**, or **complete fast**, refers to a fasting regimen, where no calories are consumed for ~~a certain period of time~~ a minimum of 24 hours.<br>Comment: Total / complete fasting can be equated with water-only fasting.<br><br>**Distribution of votes:**<br><br>A1: ▶ **44.83 %** of the panel experts voted in favour of this definition.<br>A2: ▶ **55.17 %** of the panel experts voted in favour of this definition.<br>**A3:** ▶ **0.00 %** of the panel experts voted “neither of the above”.<br><br>**→ Since no consensus was reached in round 5, the last version that reached consensus is used.**<br>**→ round 4 (see above)**
**A1: initial wording**	
P35:	A more **complete and traditional definition would be the *absence of all substances, except pure water for a certain period of time*.**
**FLUID-ONLY FASTING**	**FLUID-ONLY FASTING** – In the **fourth** round we offered the following definition:<br><br>**Fluid-only fasting** refers to a fasting regimen, whereby only beverages are consumed for a certain period of time. Water and unsweetened herbal tea may be consumed *ad libitum*. Clear vegetable broth, vegetable and/or fruit juices can be consumed up to a total of 500 kcal per day*. Ultra-processed fluids should not be consumed.<br><br>*based on the 2013 updated version of the 2002 Consensus Guidelines for Fasting Therapy.<br><br>Note: This definition includes traditional fasting regimens that use various broths, gruel, or decoctions, such as the traditional German gruel fasting.<br><br>**Distribution of votes:**<br><br>▶ **78.13 % of the panel experts voted in favour of this definition.**<br>▶ **6.25 %** of the panel experts voted neutral.<br>▶ **9.38 %** of the panel experts selected “disagree / strongly disagree”.<br>▶ **6.25 %** of the panel experts voted that this definition is irrelevant.<br><br>Although consensus was reached for this definition, changes were proposed. Comments of participants:<br><br>**DISAGREE:**<br><br>• this definition captures both fasting and modified fasting regimens. Therefore, I would prefer to define this as: *Fluid-only fasting refers to a fasting regimen, whereby no solid food calories and only zero calorie beverages are consumed.*<br>• If you are consuming up to 500 calories a day you are feeding and not fasting. This is a modified feeding program and calling it fasting is misleading.<br>• Consumption of fruit juices will completely negate any benefit of ‘fasting’ and should be removed.<br><br>We want to explain once again, why the consumption of "liquid calories" e.g., in the form of **fruit juices**, is allowed in the German tradition of fluid-only fasting:<br><br>**Drinking liquids helps to:**<br><br>• support the fasting person physiologically and psychologically to abstain from food<br>• prevent protein breakdown and muscle wasting<br>Larger amounts of fruit juice would, of course, negate the benefits of fasting. Therefore, in the evaluated studies of the German Buchinger fasting program, traditionally only small amounts of fruit/vegetable juice are allowed. The **total caloric intake does not exceed 500 kcal**, and it has been **repeatedly shown that the metabolic switch of fasting is achieved with this regimen.**

	Based on the comments received and the above information, we have adapted the definition as follows. Please select either the initial definition A1 or the adapted definition A2:<br>**A1** (initial wording)**: Fluid-only fasting** refers to a fasting regimen, whereby only beverages are consumed for a certain period of time. Water and unsweetened herbal tea may be consumed *ad libitum*. Clear vegetable broth, vegetable and/or fruit juices can be consumed up to a total of 500 kcal per day*. Ultra-processed fluids should not be consumed.<br>* based on the 2013 updated version of the 2002 Consensus Guidelines for Fasting Therapy.<br>Comment: This definition includes traditional fasting regimens that use various broths, gruel, or decoctions, such as the traditional German gruel fasting.<br><br>**A2: Fluid-only fasting** refers to a modified fasting regimen, whereby only beverages are consumed for a certain period of time. Water and unsweetened herbal tea may be consumed *ad libitum*. Clear vegetable broth, vegetable and/or fruit juices can be consumed up to a total of 500 kcal per day*. Ultra-processed fluids should not be consumed.<br>*based on the Expert Panel Update of the 2002 Consensus Guidelines for Fasting Therapy from 2013.<br>Comment: This fasting regimen includes traditional fasting regimens that use various broths, gruel, or decoctions, such as the traditional German gruel fasting.<br>**Distribution of votes:**<br>**A1** (initial wording): ▶**25.00 %** of the panel experts voted in favour of this definition.<br>**A2** (…modified fasting regimen): ▶**71.43 %** of the panel experts voted in favour of this definition.<br>**A3:** ▶ **3.57 %** of the panel experts voted "neither of the above".
**A1: initial wording**	
P13:	To me, it is **hard to definitively call fluid-only fasting true fasting or modified fasting because it depends on which fluids are consumed**. If only **water and non-caloric beverages are consumed, this would be true fasting**. However, if **broths and juices providing calories are consumed, this would be modified fasting**. Because of this, I think sticking with the broader wording (A1) is more appropriate. However, perhaps the **comment** could **clarify that fluid-only fasting could be considered true fasting or modified fasting depending on whether the beverages being ingested contain calories**.
**A2: refers to a modified fasting regimen**	
P05:	I don't feel strongly here. You can count my vote under whichever definition receives the most votes. I don't want to prevent a consensus from being reached.
P33:	This is a very specific type of fasting - so would say **one type of fluid only fasting as other groups could have a no calorie version**. Therefore if included in the definitions would include under a **heading of specific types of fasting diets not as a generic type of diet**
**Neither of the above**	
P20:	**Am still a little uncomfortable with this but I am not familiar with this work, so would be ok if you modify me to 5th.**<br>I am guessing this is many consecutive days of fasting (I can see patients doing IF and thinking they can drink 2 glasses of apple juice at lunch and dinner, stimulating glucose and insulin pathways, and knocking down benefits, mind you I think about this for all IF diets and any carbs - it is mitigating pathways - but you will still get benefits of IER and weight loss).
P29:	**I suggest replacing "a certain period of time" with "at least 24 h".**
**Intermittent Fasting Regimens**	
**TIME-RESTRICTED EATING**	**TIME-RESTRICTED EATING –** In the **fourth** round, we offered the following definition:<br>**Time-restricted eating (TRE)** is a dietary regimen in which food intake and the consumption of caloric beverages is restricted to a specific period of time during the day, resulting in a daily fasting window of at least 14 hours. There is no explicit limit on energy intake during eating hours.<br>Distribution of votes:<br>▶**87.50 % of the panel experts voted in favour of this definition.**<br>▶ **3.13 %** of the panel experts voted neutral.<br>▶ **9.38 %** of the panel experts selected "disagree / strongly disagree".<br>▶ **0.00 %** of the panel experts voted that this definition is irrelevant.<br>Although consensus was reached for this definition, it was proposed that the hours of the **fasting window** be adjusted to **12 hours**. Please select either the initial definition A1 or the adapted definition A2:<br>**A1** (initial wording): **Time-restricted eating (TRE)** is a dietary regimen in which food intake and the consumption of caloric beverages is restricted to a specific period of time during the day, resulting

	in a **daily** fasting window of at least 14 hours. There is no explicit limit on energy intake during eating hours.<br>**A2: Time-restricted eating (TRE)** is a dietary regimen in which food intake and the consumption of caloric beverages is restricted to a specific period of time during the day, resulting in a **daily** fasting window of at least **12** hours. There is no explicit limit on energy intake during eating hours.<br>**Distribution of votes:**<br>A1: ▶**62.07 %** of the panel experts voted in favour of this definition.<br>A2: ▶**31.03 %** of the panel experts voted in favour of this definition.<br>**A3:** ▶ **0.00 %** of the panel experts voted "neither of the above".<br>**Since no consensus was reached in round 5, the last version that reached consensus is used. → round 4 (see above)**
**A1: initial wording**	
P05:	**I thought we already agreed on a 14-hour definition and we already voted on the time limit.**
**A2: fasting window of at least 12 hours**	
P13:	For some individuals with a prolonged habitual eating window, such as 16 hours of eating per 24-hour period, a 12-hour **eating window would still represent a meaningful restriction**. As such, I think a minimum of 12 hours is more inclusive of various TRE protocols.
Manoogian:	*Time-restricted eating (TRE) is a dietary regimen in which all calorie intake is restricted to a consistent period of time during the 24-hour day, resulting in a daily fasting window of at* ***12-18 hours****. There is no explicit limit on energy intake during eating hours.*<br>***Comment: TRE typically only allows water outside of the designated eating window, but in some cases, non-caloric beverages such as tea or black coffee are permitted. The time of the fasting window should be customized to an individual based on their schedule and sleep time.***
**Neither of the above**	
P33:	suggest just state *Time-restricted eating (TRE) is a dietary regimen in which food intake and the consumption of caloric beverages is restricted to a specific period of time during the day*- no need to specify as each report would state the window advised
P29:	*Time-restricted eating (TRE) is a dietary regimen in which all calorie intake is restricted* ***to a consistent*** *period* ***of time during the 24-hour day****, resulting in a daily fasting window of* ***12-18 hours****. There is no explicit limit on energy intake during eating hours.*<br>***Comment: TRE allows water consumption outside of the designated eating window, but in some cases, non-caloric beverages such as unsweetened tea or black coffee are permitted. The time of the fasting window should be customized to an individual based on their schedule and sleep time.***
**INTERMITTENT ENERGY RESTRICTION (IER)**	**INTERMITTENT ENERGY RESTRICTION** – In the **second** AND **fourth** round, consensus was reached for this term with opposing wording.<br>**ROUND 2:**<br>Definition: **Intermittent Energy Restriction (IER)** includes periods of caloric restriction alternating with periods of *ad libitum* eating. As such, IER includes fasting regimens like intermittent fasting (IF) and time-restricted eating (TRE).<br>**Distribution of votes:**<br>▶**81.26 % of the panel experts voted in favour of this definition.**<br>▶ 0.00 % of the panel experts voted neutral.<br>▶15.63 % of the panel experts selected "disagree / strongly disagree".<br>▶ 3.13 % of the panel experts voted that this definition is irrelevant.<br>**ROUND 4:**<br>Definition: **Intermittent Energy Restriction (IER)** includes periods of modified fasting alternating with periods of ad libitum energy intake.<br>Note: IER is a subcategory of intermittent fasting (IF) and includes regimens such as alternate-day modified fasting (ADMF), fasting on 2 separate or consecutive days per week, weeks of caloric restriction followed by weeks of eating in energy balance, and the fasting-mimicking diet.<br>**Distribution of votes:**<br>▶**74.19 % of the panel experts voted in favour of this definition.**<br>▶ 0.00 % of the panel experts voted neutral.<br>▶20.58 % of the panel experts selected "disagree / strongly disagree".<br>▶ 3.23 % of the panel experts voted that this definition is irrelevant.<br>Although consensus was reached for this definition in the last round, changes have been proposed that are similar to the wording of the definition in the second round<br>Comments of participants in round 4:<br>**DISAGREE:**

- I think the definition of IER should be modified to use "caloric restriction" rather than "modified fasting". For example: "I*ntermittent Energy Restriction (IER) includes periods of caloric restriction alternating with periods of ad libitum energy intake.*" If "modified fasting" is used, it would mean that an eating program would only qualify as IER if a large energy deficit is used (down to the maximal allowable % intake in whichever definition of modified fasting is approved). In contrast to this, I think many IER programs use smaller energy deficits (I.e., higher energy intake than would be allowed by modified fasting but less than weight maintenance energy needs). This is my reasoning for thinking "caloric restriction" would be more appropriate than "modified fasting" in the definition. Even if that change wasn't made, I feel like the note may need to be revised. I was surprised to see IER presented as a subcategory of IF within the note because the previous definition presented this relationship the opposite way (I.e., IF was a subcategory of IER). My view is that IER is a broader concept (alternating ad libitum or weight maintenance calorie intake with periods of caloric restriction, as explained above).
- …a hard to reach agreement if we do include a modified fasting……..
- if it is intermittent energy restriction then it should refer to intermittent calorie restriction and not modified fasting

STRONGLY DISAGREE:

- I think the group has got v confused! We need a term for intermittent diets . The term Intermittent Energy Restriction is an overarching term for modified fasting etc and not a sub group of this. Suggest the modified fasting term above includes intermittent than this should be covered i.e intermittent modified fasting refers to limiting energy intake. Modified fasting regimens encompass alternate-day modified fasting, fasting practiced on a number of separate or consecutive days per week and asting-mimicking diets.(likely to be more than one!)
- IF and TRE are a subcategory of IER, not the other way around. All of these diets are IER – but if you do not have low carb/zero calories for a prolonged period in a day then you are not doing IF or TRE. Of note, I agree IF is different from TRE (due to mechanism of action as noted on first page) and it shouldn't be placed under 'IF' but under 'IER'.

Based on the comments received, we are offering an adapted version of the definition from the **second** round as well as the definition from the **last** round for reevaluation.

Below is an illustration to help you understand the connections between the terms IER, IF, and TRE, depending on which definition you choose for IER. (If your browser does not allow you to view the illustration, you can also find it attached to the email with the link to this survey.) Please select either the adapted definition A1 from the second round or definition A2 from the last round.

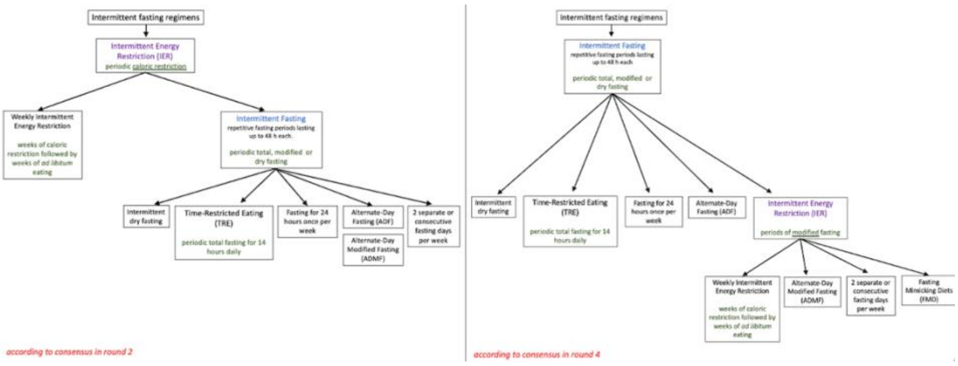

A1 (similar to round 2): Intermittent Energy Restriction (IER) or intermittent caloric restriction, includes periods of caloric restriction alternating with periods of *ad libitum* eating.

Comment: IER~~ is a subcategory of intermittent fasting (IF) and ~~includes all intermittent fasting regimens~~, fasting-mimicking diets~~* and weeks of caloric restriction followed by weeks of *ad libitum* eating.

*With a duration of 3 to 7 days, FMDs should, in our opinion, be placed among the continuous and not the intermittent fasting regimens.

A2 (wording from round 4): **Intermittent Energy Restriction (IER)** includes periods of modified fasting alternating with periods of *ad libitum* energy intake.

Comment: IER is a subcategory of intermittent fasting (IF) and includes regimens such as alternate-day modified fasting (ADMF), fasting on 2 separate or consecutive days per week, weeks of caloric restriction followed by weeks of eating in energy balance, and the fasting-mimicking diet.

Distribution of votes:

A1: ▶**46.43 %** of the panel experts voted in favour of this definition.
A2: ▶**39.29 %** of the panel experts voted in favour of this definition.
A3: ▶**14.29 %** of the panel experts voted "neither of the above".

→ No consensus was reached in the fifth round. According to the rules we would have to abide by the last consensus reached (see round 4).

Nevertheless, we decided to ratifiy the definition that reached consensus in **round 2** instead, for the following reasons:

- this definition is consistent with the accepted definition for intermittent fasting
- this definition received more agreement (81.26%) than the definition of round 4 (74.19%)

We are aware that some participants on this expert panel disagree with the final definitions for IER, IF and TRE. We would like to remark that some participants consistently noted they would not classify TRE under IF because TRE is based on chronobiological considerations. According to these participants, all (other) intermittent fasting regimens are based on calorie restriction, whereas TRE theoretically does not require calories to be reduced in the eating window. This dissent will be discussed in the publication. In our opinion, there are quite a number of aspects for which there are not yet sufficient data to make clear statements. Therefore, we would like to point out that these definitions can certainly be revised in the future and will as such be discussed in the publication

A1: wording similar to round 2

P13: I just think the organization from round 2 makes more sense and is **more consistent with how these programs are frequently defined and organized in the literature.**

A2: wording from the last round

Mannogian: **Time-restricted feeding is not a form of intermittent fasting. I strongly disagree with this definition**. TRE is unique from IF in that is 1. Has a consistent fasting window. 2. Does not require energy restriction, and 3. Has to be practiced daily. I see how these are noted in the definition from round 4, but I feel it is confusing to consider it a form of IF at all.

Neither of the above

P05: I would define continuous energy restriction as the "reducing energy intake on every day." Based on this, I would **define intermittent energy restriction as an approach where energy intake is restricted on some days but not restricted on other days.** Thus, I think a better definition is as follows:

Intermittent Energy Restriction (IER) includes day(s) of fasting or modified fasting interspersed with day(s) of ad libitum energy intake.

Comment: IER is a subcategory of intermittent fasting (IF) and includes regimens such as alternate-day fasting (ADF), alternate-day modified fasting (ADMF), the 5:2 diet, weekly intermittent energy restriction, and the fasting-mimicking diet. ***It does not include time-restricted eating (TRE).***

P20: **Please modulate to 2-3 separate or consecutive days fasting days per week. We typically run 3 fasting days per week, which is not technically ADF.**

I also think you could make everyone happy by the umbrella term 'intermittent energy restriction' which splits into IF, ADF, TRE, and PF.(75, 76)

P33: *IER is a subcategory of intermittent fasting (IF) and includes regimens such as alternate-day modified fasting (ADMF), fasting on 2 separate or consecutive days per week,*

Is the group defining intermittent as = < 48 hours weeks of caloric restriction. Then it cant include weeks of CR & weeks eating in energy balance,

Suggest take the fMD out as this is a specific type of diet

P29: I strongly disagree with including TRE with intermittent fasting. TRE is the only dietary regimen that does not explicitly prescribe calorie reduction on any day, while all other IFs do. **TRE is also based on the science of circadian rhythm, while all other here are based on calorie restriction**. This implies the mechanisms, practice and potential outcomes of TRE are and will be different (some may be shared) with other forms of fasting. For example, TRE participants often report better sleep, while ADF and 5:2 do not. **Lumping TRE with other IFs is dangerous for public adoption, because any adverse effect found in other forms of IFs will be perceived as an adverse effect that applies to all forms of IFs including TRE.**

Continuous Fasting Regimens

SHORT-TERM FASTING (STF)

SHORT-TERM FASTING – In the **fourth** survey, we offered the following definition:

Short-term fasting (STF) refers to a fluid-only fasting regimen with a duration of 1-3 days.

Distribution of votes:

▶ **81.25 % of the panel experts voted in favour of this definition.**
▶ **6.25 %** of the panel experts voted neutral.
▶ **12.50 %** of the panel experts selected "disagree / strongly disagree".
▶ **0.00 %** of the panel experts voted that this definition is irrelevant.

Although consensus was reached for this definition, we received several comments on the duration and type of fasting regimens included. Based on these comments, we have adjusted the above definition (see A3 and A4). We also had a discussion in our team about the duration of STF and would like to propose an additional option (see A2) for the following reasons:

	• The panel has already agreed that **intermittent fasting** is defined as repetitive fasting periods lasting **up to 48 hours**.<br>• We believe that one-day fasts do not make much sense from a clinical perspective.<br>To bring this definition in line with the already accepted definition of intermittent fasting (= fasting regimens lasting up to 48 hours (= 2 days)), we would propose STF to be defined as fasting regimens with a duration of **2-3 days**. Prolonged fasting would then be any fasting regimen with a duration of 3 days or more.<br>Please select your preferred definition.<br>**A1** (initial wording**): Short-term fasting (STF)** refers to a fluid-only fasting regimen with a duration of 1-3 days.<br>**A2 (our proposal): Short-term fasting (STF)** refers to total and modified fasting regimens with a duration of **2-3 days.**<br>**A3: Short-term fasting (STF)** refers to total and modified fasting regimens with a duration of **1-2 days.**<br>**A4: Short-term fasting (STF)** refers to total and modified fasting regimens with a duration of 1-3 days.<br>**Distribution of votes:**<br>A1(initial wording 1-3 days): ▶ **17.24 %** of the panel experts voted in favour of this definition.<br>A2 (our proposal 2-3 days): ▶ **48.28 %** of the panel experts voted in favour of this definition.<br>A3 (1-2 days): ▶ **6.90 %** of the panel experts voted in favour of this definition.<br>A4 (1-3 days): ▶ **27.59 %** of the panel experts voted in favour of this definition.<br>A5: ▶ **0.00 %** of the panel experts voted "neither of the above".
**A2: 2-3 days (our proposal)**	
P13:	I completely agree that adjacent fasting definitions should not overlap in duration (I.e., the duration of intermittent fasting programs should not overlap with short-term fasting), as this could produce much confusion.
P33:	would need to say either **short term total fast or short term modified fast**
**PROLONGED FASTING (PF) / LONG-TERM FASTING:**	**LONG-TERM FASTING** – In the **fourth** survey, we offered the following definition:<br>**Prolonged fasting (PF)**, also called **long-term fasting (LTF)**, refers to any fluid-only fasting regimen lasting ≥ 4 consecutive days.<br>Distribution of votes:<br>▶ **81.25 % of the panel experts voted in favour of this definition.**<br>▶ **3.13 %** of the panel experts voted neutral.<br>▶ **15.63 %** of the panel experts selected "disagree / strongly disagree".<br>▶ **0.00 %** of the panel experts voted that this definition is irrelevant.<br>Although consensus was reached for this definition, we received several comments regarding the **duration** and **type** of fasting regimen. Based on these comments, we adapted the definition above. Please select your preferred definition.<br>**A1** (initial wording): **Prolonged fasting (PF),** also called **long-term fasting (LTF)**, refers to any fluid-only fasting regimen lasting ≥ 4 consecutive days.<br>**A2: Prolonged fasting (PF),** also called **long-term fasting (LTF)**, refers to ~~fluid-only~~ total and modified fasting regimens with a duration of ≥ 4 consecutive days.<br>**A3: Prolonged fasting (PF),** also called **long-term fasting (LTF)**, refers to ~~fluid-only~~ total and modified fasting regimens with a duration of ≥ 3 consecutive days.<br>**Distribution of votes:**<br>**A1** (initial wording ≥ 4 days): ▶ **27.59 %** of the panel experts voted in favour of this definition.<br>**A2** (changes and ≥ 4 days): ▶ **51.72 %** of the panel experts voted in favour of this definition.<br>**A3** (≥ 3 days): ▶ **20.69 %** of the panel experts voted in favour of this definition.<br>**A4:** ▶ **0.00 %** of the panel experts voted "neither of the above".
**PERIODIC FASTING (PF)**	**PERIODIC FASTING –** In the **fourth** survey we offered the following definition for the term **periodic fasting.**<br>**Periodic fasting (PF)** refers to any fasting regimen that is repeated at regular intervals (periods), such as every day, every week, or every several months.<br>Note: According to this definition, periodic fasting would include intermittent fasting regimens.<br>Distribution of votes:<br>▶ **81.26 % of the panel experts voted in favour of this definition.**<br>▶ **3.13 %** of the panel experts voted neutral.

	▶ **15.63 %** of the panel experts selected "disagree / strongly disagree".<br>▶ **0.00 %** of the panel experts voted that this definition is irrelevant.<br>Although consensus was reached for this definition, several changes were proposed. Based on these comments, we adapted the definition above. Please select either the initial definition A1 or the adapted definition A2:<br>**A1** (initial wording)**: Periodic fasting (PF)** refers to any fasting regimen that is repeated at regular intervals (periods), such as every day, every week, or every several months. Comment: According to this definition, periodic fasting would include intermittent fasting regimens.<br>**A2: Periodic fasting (PF)** refers to ~~any~~ prolonged fasting regimens that are repeated at ~~is repeated at regular~~ intervals (periods)~~, such as every day, every week, or every several months~~.<br>**Distribution of votes:**<br>A1: ▶ **53.57 %** of the panel experts voted in favour of this definition.<br>A2: ▶ **46.43 %** of the panel experts voted in favour of this definition.<br>A3: ▶ **0.00 %** of the panel experts voted "neither of the above".
**A1: initial wording**	
P33:	dont think need to specify the definition
**Specific Fasting Regimens**	
**FASTING-MIMICKING DIET (FMD)**	**FASTING-MIMICKING DIET (FMD) –** In the **fourth** survey we offered the following definition:<br>A **Fasting-Mimicking Diet (FMD)** specifies any diet that is specifically composed to induce the metabolic effects of fasting while allowing for a potentially higher caloric intake, including solid foods. It usually refers to a plant-based, calorie restricted diet with a maximum of 1400 kcal per day that lasts 3 to 7 days. FMDs are usually relatively low in refined sugars and starch, low in protein and high in plant-based fats.<br>Note: It is recommended to follow the FMD once a month to every four months.<br>**Distribution of votes:**<br>▶ **78.78 % of the panel experts voted in favour of this definition.**<br>▶ **0.00 %** of the panel experts voted neutral.<br>▶ **15.15 %** of the panel experts selected "disagree / strongly disagree".<br>▶ **6.06 %** of the panel experts voted that this definition is irrelevant.<br>Although consensus was reached for this definition, changes were proposed. Comments of participants:<br>**AGREE:**<br>• IMO there is really not enough research on FMD by enough separate groups to define exactly what they are in terms of length, timing/ kcal composition.<br>**DISAGREE:**<br>• I think maximum of 1000 cal and having and is followed periodically (e.g. once a month makes this more accurate<br>**STRONGLY DISAGREE:**<br>• I disagree with this definition for two main reasons: a) the threshold of 1400 Kcal per day is definitely too high for such a regimen. Indeed, this threshold is close to a normocaloric diet for several people, including people with low height/BMI. In addition, this amount of calorie is unlikely to cause the positive metabolic effects that are expected by a FMD, such as a reduction of blood glucose, growth factors and lipid levels. For instance, in the study by Valdemarin f et al (Cancers), in which a FMD regimen or max 1000 Kcal/die was administered to cancer patients, the metabolic effects were much milder than those obtained with a more severe FMD regimen (Vernieri et al. Cancer Discov 2022). I suggest max 800-1000 Kcal per day in this definition b) it is stated that a FMD should "induce the metabolic effects of fasting". However, according to the definition of fasting that was accepted by the majority of us, fasting is the abstinence from some or all foods. This definition does not necessarily imply a reduction in calorie intake, nor of specific macronutrients. For instance, totally avoiding meat but consuming a high amount of carbohydrates or sugars can be still defined "fasting" according to this definition. Being this definition generic and including different types of dietary styles/regimens, no unique metabolic effects can be expected. Therefore, I would modify the definition of FMD as a regimen that is able to "induce the metabolic effects of complete fasting", rather than "fasting"<br>• How do we know whether we really achieve the same metabolic effects of fasting like autophagy, ketosis, hormonal changes, reduction of proinflammatory cytokines and so on with a daily intake of 1000 or even 1400 Kcal also containing many carbohydrates ? Do we have valid scientific data proving this statement?<br>• This should be a general term. There are likely to be many different versions of this i.e low carb not low protein and of different lengths.

The definition currently proposed SHOULD BE REMOVED. IT IS REFERRING TO ONE COMMERCIAL PRODUCT and not a class of dieting

THIS DEFINITION IS IRRELEVANT:

- Who is "the leading expert" on FMD? Did they disclose any financial conflicts of interest?

Based on the comments received, we have adapted the definition as follows. Please select either the initial definition A1 or the adapted definition A2:

A1 (initial wording)**:** A **Fasting-Mimicking Diet (FMD)** specifies any diet that is specifically composed to induce the metabolic effects of fasting while allowing for a potentially higher caloric intake, including solid foods. It usually refers to a plant-based, calorie restricted diet with a maximum of 1400 kcal per day that lasts 3 to 7 days. FMDs are usually relatively low in refined sugars and starch, low in protein and high in plant-based fats.

Comment: It is recommended to follow the FMD once a month to every four months.

A2: A **Fasting-Mimicking Diet (FMD)** specifies any diet that is specifically composed to induce the metabolic effects of fasting while allowing for a potentially higher caloric intake, including solid foods. It usually refers to a plant-based, calorie restricted diet with a maximum of approximately 1000 kcal per day that lasts 3 to 7 days. FMDs are usually relatively low in refined sugars and starch, low in protein and high in plant-based fats.

~~Note: It is recommended to follow the FMD once a month to every four months.~~ Comment: The exact amount of calories, macronutrient composition, duration and frequency of use needs to be decided individually. FMD meals can consist of packaged products or be freshly prepared.

Distribution of votes:

A1: ▶**10.71 %** of the panel experts voted in favour of this definition.
A2: ▶75.00 % of the panel experts voted in favour of this definition.
A3: ▶ **7.14 %** of the panel experts voted "neither of the above".
A4: ▶ **7.14 %** of the panel experts voted that this definition is irrelevant.

A2: with a maximum of 1000 kcal

P06: I believe that definition A1 is wrong.

How is it possible to hypothesize that FMD has "the metabolic effects of fasting" with a daily calorie intake of up to 1400 KCal (which approximates the normal calorie intake for several people)? I believe that this is highly unlikely and non-physiological, and may generate confusion. I personally believe **that even the 1000 KCal threshold is too high to recapitulate "the metabolic effects of fasting" with an FMD regimen**. I would propose **up to 700 KCal/day. But of course not 1400 KCal/day!**

P05: I prefer to **go with whatever definition Valter Longo prefers.**

Neither of the above

P33: *A Fasting-Mimicking Diet (FMD) specifies any diet that is specifically* composed to induce the metabolic effects of fasting while allowing for intake of some calories and nutrients,. Probably a good idea to define the nutrition of a FMD to induce a fasting effect which the group needs to agree on.

perhaps maximum of approximately 1000 kcal per day that lasts 3 to 7 days. It could be liquid or solid food not just solid

P29: Not that I disagree with these definition, **FMD is trademarked by L-Nutra and it is typically unusual or may be illegal to alter the definition of a Trademark. So, please check with trademark laws.**

RELIGIOUS FASTING

RELIGIOUS FASTING – In the fourth survey we offered the following definition:

Religious fasting refers to any fasting regimen that is undertaken as part of a religious practice.

Note: Religious fasting thus involves practices such as: dry fasting on specific days of the year up to 25 hours at a time (e.g. Jewish tradition, The Church of Jesus Christ of Latter-day Saints); intermittent dry fasting (e.g. Ramadan fasting, Bahá'í fasting); time-restricted eating (e.g. Buddhism); and diets restricting certain foods (e.g. Christian orthodox traditions, Daniel fast) if more broadly defined. Typically, religious fasting includes spiritual activities to improve cognitive function and well-being.

Distribution of votes:

▶**93.76 % of the panel experts voted in favour of this definition.**
▶ **3.13 %** of the panel experts voted neutral.
▶ **0.00 %** of the panel experts selected "disagree / strongly disagree".
▶ **3.13 %** of the panel experts voted that this definition is irrelevant.

Although consensus was reached for this definition, an addition to the definition was proposed (see marked in blue). Based on the comments received, we have adapted the definition as follows. Please select either the initial definition A1 or the adapted definition A2:

	**A1** (initial definition): **Religious fasting** refers to any fasting regimen that is undertaken as part of a religious practice.<br>Comment: Religious fasting thus involves practices such as: dry fasting on specific days of the year up to 25 hours at a time (e.g. Jewish tradition, The Church of Jesus Christ of Latter-day Saints); intermittent dry fasting (e.g. Ramadan fasting, Bahá'í fasting); time-restricted eating (e.g. Buddhism); and diets restricting certain foods (e.g. Christian orthodox traditions, Daniel fast) if more broadly defined. Typically, religious fasting includes spiritual activities to improve cognitive function and well-being.<br>**A2**: **Religious fasting** refers to any fasting regimen that is undertaken as part of a religious practice.<br>Comment: Religious fasting thus involves practices such as: dry fasting on specific days of the year up to 25 hours (e.g. Jewish tradition, The Church of Jesus Christ of Latter-day Saints) or up to 60 hours at a time (e.g. Christian orthodox tradition); intermittent dry fasting (e.g. Ramadan fasting, Bahá'í fasting); time-restricted eating (e.g. Buddhism); and diets restricting certain foods (e.g. Christian orthodox traditions, Daniel fast) if more broadly defined. Typically, religious fasting includes spiritual activities aimed at improving cognitive function and well-being.<br>**Distribution of votes:**<br>**A1**: ►**29.63 %** of the panel experts voted in favour of this definition.<br>**A2**: ►**66.67 %** of the panel experts voted in favour of this definition.<br>**A3:** ► **3.70 %** of the panel experts voted "neither of the above".
**A2: modified comment**	
P05:	I don't feel strongly on this definition. You can count my vote towards whichever option receives the most votes.
**Neither of the above**	
P14:	Religious fasts are "**aimed at growing the relationship between the individual and God**" and not about cognitive function and well-being. That really needs to be included for this definition to be relevant and accurate. For example, Muslims don't fast during Ramadan to improve cognitive function. They do so **to engage in focused prayer for a period of time.** Same is true for Christians and other groups. It's the **prayer and spiritual growth that are important, with little to do with health benefits**.

Terms on which consensus has not yet been reached

Specific Fasting Regimens	
**Addition to THERAPEUTIC / MEDICAL FASTING**	**THERAPEUTIC / MEDICAL FASTING –** In the **fourth** round, we offered the following definition which received consensus:<br>**Therapeutic fasting** refers to any fasting regimen that is applied as a therapeutic intervention. Therapeutic fasting interventions are individually tailored to a person's age, sex, body composition, physical activity level, occupation, goal & planned duration of fasting.<br>**Medically supervised fasting** refers to any fasting regimen that is applied as a therapeutic intervention by a trained physician or similar credentialed healthcare provider.<br>In the context of these terms, we would like to propose an additional definition for the term **preventive fasting**:<br>**Preventive fasting** refers to any fasting regimen that is applied as a preventive intervention.<br>To what extend do you agree or disagree with this modified definition?<br>**Distribution of votes:**<br>►**82.14 %** of the panel experts voted in favour of this definition.<br>► **0.00 %** of the panel experts voted neutral.<br>► **3.45 %** of the panel experts selected "disagree / strongly disagree".<br>►**14.29 %** of the panel experts voted that this definition is irrelevant.
**AGREE: further thoughts**	
P30:	... that is applied in order to prevent any life style-related diseases
**DISAGREE: suggested modifications**	
P20:	I do not get it: Most if not all the fasting regimes are preventive
**This definition is irrelevant**	
P33:	this sounds very speculative as no high quality data of what it can prevent !

P29:	Does not say prevention of what? Almost everyone has some physical, or emotional condition that can modify under certain forms of fasting. Besides, this definition is irrelevant.

General terms concerning fasting

**MODIFIED FASTING**	**MODIFIED FASTING –** In the **fourth** round we offered the following definition:<br>The term **modified fasting** refers to limiting energy intake, typically up to (…)* % of energy needs on fasting days. Modified fasting regimens encompass alternate-day modified fasting, fasting practiced on 2 separate or consecutive days per week and the fasting-mimicking diet. These regimens are often adapted to specific clinical settings and indications, allowing for different complementary or supportive therapeutic interventions.<br>* We asked you to choose which threshold value for the allowed percentage of energy requirements (= kcal intake) on modified fasting days you prefer:<br>• Maximum energy intake of 25% of energy requirements<br>• Maximum energy intake of 35% of energy requirements<br><br>**Distribution of votes for the definition itself:**<br>▶ **81.25 % of the panel experts voted in favour of this definition.**<br>▶ **3.13 %** of the panel experts voted neutral.<br>▶ **12.51 %** of the panel experts selected "disagree / strongly disagree".<br>▶ **3.13 %** of the panel experts voted that this definition is irrelevant.<br><br>**Distribution of votes for the threshold:**<br>▶ **61.29 %** of the panel experts voted in favour of 25% of energy requirements.<br>▶ **19.35 %** of the panel experts voted in favour of 35% of energy requirements.<br>▶ **9.68 %** of the panel experts selected "neither of the above".<br>▶ **9.68 %** of the panel experts voted that this detail is irrelevant.<br><br>Although consensus was reached for the definition itself, no consensus was reached on the threshold value.<br>Comments of participants:<br>**DEFINITION - DISAGREE:**<br>• Should not include FMD<br>• this should be intermittent modified fasting refers to limiting energy intake.<br>• I think that eating whenever you like during modified fasting instead makes it an IER diet. If low carbohydrate foods, or prolonged consecutive fasting periods, then modified-fasting is appropriate.<br>**THRESHOLD:**<br>• I would say that the max number of calories of a FMD (1400) should be the max number of calories of a modified fasting regimen, since FMDs are included in the definition<br>• if fmd is included then the 35% calorie limit is not appropriate<br>• […] typically up to **75%** of energy needs […]<br>• Would put up to **50%** of energy needs<br><br>In the online live discussion, it was decided to use an umbrella term such as **modified fasting** to encompass **all fasting regimens that allow some caloric intake** during the **"fasting" period**. The thresholds discussed for this definition were 25% and 35%. However, it was noted in the last round that these percentages are too low to include FMDs with up to ≈ 1000 kcal. On the other hand, choosing a higher threshold would make the definition of modified fasting too similar to the definition of caloric restriction. We therefore prepared a **definition of modified fasting that excludes FMDs**. Please vote again on the slightly adapted definition with the **threshold of 25%**, which received the majority of votes in the last round:<br>The term **modified fasting** refers to limiting energy intake to typically up to 25% of energy needs on modified fasting days.<br>Comment: Modified fasting regimens can be adapted to specific clinical settings and indications, allowing for different complementary or supportive therapeutic interventions. Examples of modified fasting regimens are alternate-day modified fasting, fasting practiced on 2 separate or consecutive days per week and fluid-only fasting ~~and the fasting-mimicking diet~~.*<br>To what extend do you agree or disagree with this modified definition?<br>**Distribution of votes:**

	▶ 86.20 % **of the panel experts voted in favour of this definition.**<br>▶ **0.00 %** of the panel experts voted neutral.<br>▶ **10.34 %** of the panel experts selected "disagree / strongly disagree".<br>▶ **3.45 %** of the panel experts voted that this definition is irrelevant.
**STRONGLY AGREE: further thoughts**	
P06:	While I totally agree with the definition of "modified fasting" proposed here, I totally disagree with the above-reported statement: "In the online live discussion, it was decided to use an umbrella term such as modified fasting to encompass all fasting regimens that allow some caloric intake during the "fasting" period. "<br>Indeed, based on the definition on which we agreed during the online live discussion, "fasting" generally refers to the restriction of any food or beverage, regardless of a specific restriction in calorie intake. Therefore, "**modified fasting", as proposed here, is a subset of all possible fasting regimens, which is characterized by a significant restriction of calorie intake** (up to 25% of energy needs), and **not by the fact that it allows "some calorie intake"** (almost all fasting regimens, with the exception of water only fasting, allow some calorie intake). I believe that this is a crucial conceptual difference.
**AGREE: further thoughts**	
P23:	I would omit the last words of the definition ("on modified fasting days"), since this is self-explanatory
**DISAGREE: suggested modifications / alternative definition**	
P05:	**No consensus was reached on the threshold of 25%, so I don't believe that we should publish 25% as the threshold in the manuscript. That violates the rules of this process.**<br>I believe modified fasting **should include the fasting-mimicking diet and should entail up to 35% of normal daily intake.**
P20:	**2-3 separate days.**
P33:	dont think you can specify the degree of calorie restriction
**This definition is irrelevant**	
P29:	Too many definitions can make it too confusing.
**Intermittent Fasting Regimens**	
**ALTERNATE-DAY MODIFIED FASTING**	**ALTERNATE-DAY MODIFIED FASTING** – In the **fourth** survey, we offered the following two definitions:<br>**A1: Alternate-day modified fasting (ADMF)** refers to alternating a day of eating *ad libitum* and a day of modified fasting.<br>**A2: Alternate-day modified fasting (ADMF)** refers to alternating a day of eating ad libitum and a day of modified fasting with limited caloric intake at lunch or dinner.<br>**Distribution of votes:**<br>▶ **62.50 %** of the panel experts voted in favour of definition A1.<br>▶ **28.13 %** of the panel experts voted in favour of definition A2.<br>▶ **6.25 %** of the panel experts selected "neither of the above".<br>▶ **3.13 %** of the panel experts voted that this definition is irrelevant.<br>Hoddy et al. (https://pubmed.ncbi.nlm.nih.gov/25251676/) discovered that eating the fasting meal at lunchtime led to similar results in weight loss and cardioprotection as eating it at dinner or as smaller meals throughout the day. The authors note that this meal flexibility may improve tolerability and long-term adherence to ADMF protocols. Since it doesn't seem to matter when meals are consumed, we decided to omit definition A2 and present only the **shorter** definition A1 for reevaluation. (No changes were proposed.)<br>**Alternate-day modified fasting (ADMF)** refers to alternating a day of eating ad libitum and a day of modified fasting.<br>To what extend do you agree or disagree with this definition?<br>**Distribution of votes:**<br>▶ 96.43 % **of the panel experts voted in favour of this definition.**<br>▶ **0.00 %** of the panel experts voted neutral.<br>▶ **0.00 %** of the panel experts selected "disagree / strongly disagree".<br>▶ **3.57 %** of the panel experts voted that this definition is irrelevant.
**This definition is irrelevant**	
P29:	Too many definitions can make it too confusing.
